# Synthesis of *Streptococcus pneumoniae* serotype 9V oligosaccharide antigens

**DOI:** 10.3762/bjoc.16.140

**Published:** 2020-07-15

**Authors:** Sharavathi G Parameswarappa, Claney L Pereira, Peter H Seeberger

**Affiliations:** 1 Max Planck Institute of Colloids and Interfaces, Biomolecular Systems Department, Am Mühlenberg 1, 14476 Potsdam, Germany; 2 Vaxxilon Deutschland GmbH, Magnusstraße 11, 12489 Berlin, Germany; 3 Freie Universität Berlin, Institute for Chemistry and Biochemistry, Arnimallee 22, 14195 Berlin, Germany

**Keywords:** antigen, carbohydrate chemistry, oligosaccharide, *Streptococcus pneumoniae*, vaccines

## Abstract

*Streptococcus pneumoniae* (SP) bacteria cause serious invasive diseases. SP bacteria are covered by a capsular polysaccharide (CPS) that is a virulence factor and the basis for SP polysaccharide and glycoconjugate vaccines. The serotype 9V is part of the currently marketed conjugate vaccine and contains an acetate modification. To better understand the importance of glycan modifications in general and acetylation in particular, defined oligosaccharide antigens are needed for serological and immunological studies. Here, we demonstrate a convergent [2 + 3] synthetic strategy to prepare the pentasaccharide repeating unit of 9V with and without an acetate group at the C-6 position of mannosamine.

## Introduction

*Streptococcus pneumoniae* (SP), a Gram-positive bacterium colonizes the nasopharynx [1] and causes invasive diseases such as meningitis, otitis media, and pneumonia [2]. Invasive pneumococcal diseases cause high mortality and morbidity in children, the elderly and in immunocompromised individuals particularly in developing countries [3]. With increasing antimicrobial resistance to antibiotics, vaccines are becoming even more important to control these pathogens. Despite the availability of multivalent polysaccharide and glycoconjugate vaccines such as Pneumovax, Prevnar^®^ 13, and Synflorix, pneumonococcal diseases are still of growing concern due to an increase in non-vaccine serotypes and the cost of implementing these expensive vaccines in national immunization programs [4–6].

CPS is an important bacterial virulence factor and is critical for the interaction with the host as it helps the bacteria to escape the host immune response [1,7]. These polysaccharides of SP consist of repeating units (RU) that range from di- to heptasaccharides that may be branched and/or charged [8]. Modifications such as O-acetylation, phosphorylation, and sulfation further increase CPS complexity. Many bacterial polysaccharides are O-acetylated [9–10]. Especially SP serotypes, such as 9 (A, V) and 18C differ in O-acetylation. Two O-acetylated serotypes (9V and 18C) are part of the commercial vaccine Prevnar^®^ 13. The traditional CPS isolation approach produces varying length CPS with different degrees of acetylation. An acetate loss during isolation, purification, or protein conjugation leads to structurally altered CPS. Vaccines based on synthetic carbohydrate antigens [11–15] such as the first commercially available semisynthetic glycoconjugate vaccine Quimi Hib^®^ against *H. influenzae* [16] and *Shigella flexneri* pentadecasaccharide that passed phase-I clinical trials [17–18] incorporate defined oligosaccharides.

**Figure 1:**
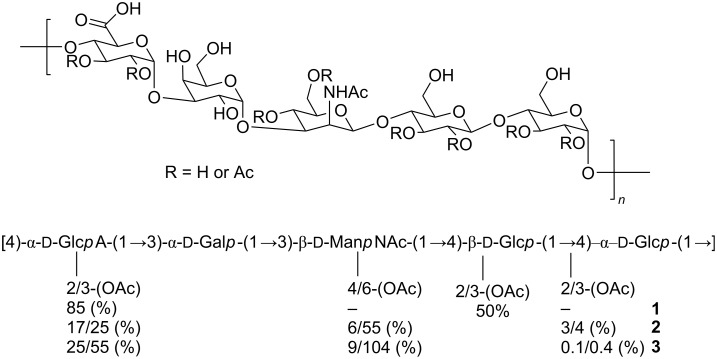
*Streptococcus pneumoniae* 9V repeating unit. The numbers refer to the version concerning the structure that was revised multiple times over the past forty years regarding the degree of acetylation.

SP 9 contains four capsular types (9A, 9L, 9N and 9V) whereby the most prevalent, 9V (57%) affects young children and 9N (34%) infects adults [19]. CPS of 9V and 9A serotypes differ only in the degree of acetylation of the same pentasaccharide RU. The positions and degree of RU acetylation has been revised several times since the initial structure **1** was proposed in 1981 (**2** and **3** in Figure 1) [20–22].

Antibodies raised against the natural 9V polysaccharide recognize the natural and the de-O-acetylated form of 9V, but only the antiserum that recognizes O-deacetylated 9V CPS showed opsonophagocytic activity suggesting that O-acetylation was not essential for a protective antibody response [23]. Another study revealed that rabbit 9V antiserum showed decreasing binding to O-deacetylated 9V PS compared to the acetylated version whereas mAb against the 9V polysaccharide bound O-acetylated and de-acetylated 9V PS about the same and showed opsonophagocytic activity, and passively protected young mice against SP9V challenge [24]. With the role of acetylation still not understood, synthetic SP9V oligosaccharide antigens are key to study the role of O-acetylation in protection and to confirm the structure of the natural RU. To date, only partial syntheses of the SP9A/V backbone have been reported but none have addressed the acetylation issue [25–27]. Oscarson et al. reported the synthesis of the natural pentasaccharide repeating unit as the methyl ester with no acetylation. Since our previous studies involving ST3 [12] and ST8 [13] indicated glucuronic acid to be an important epitope for immunogenicity, we embarked on the syntheses of 9A/9V but using a frameshift sequence of the natural pentasaccharide RU with glucuronic acid at the non-reducing end.

## Results and Discussion

**Scheme 1:**
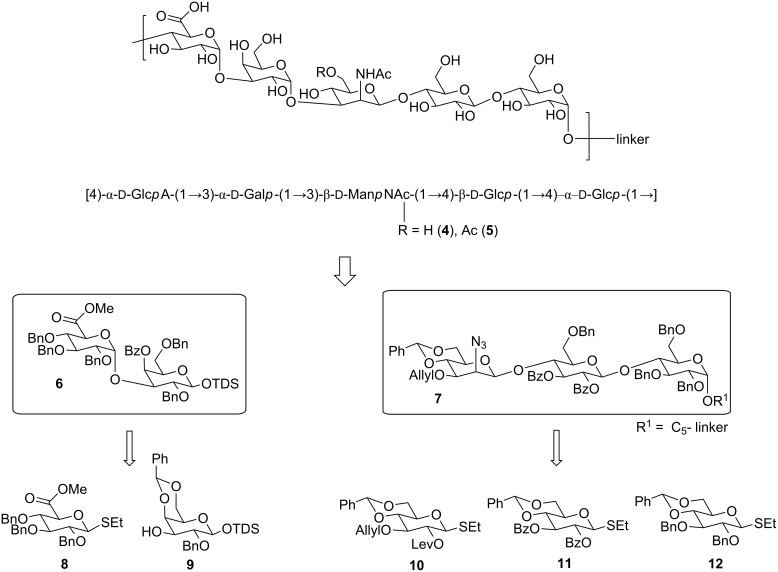
Retrosynthesis of *Streptococcus pneumoniae* 9V deacetylated (**4**) and acetylated (**5**) repeating units.

The SP 9V linear pentasaccharide RU **1** contains ᴅ-glucuronic acid (ᴅ-Glc*p*A), ᴅ-galactose (ᴅ-Gal*p*), 2-acetamido-2-deoxy-ᴅ-mannose (ᴅ-Man*p*NAc), and ᴅ-glucose (ᴅ-Glc*p*) units. Several *cis* glycosidic linkages, including a β-mannosidic linkage, have to be installed stereoselectively while taking provision to install the C-6 *O*-acetate in Man*p*NAc. The synthetic approach (Scheme 1) relies on a late stage [2 + 3] α-glycosylation between disaccharide **6** and trisaccharide **7** to obtain the fully protected pentasaccharide RU. The di- and trisaccharide will be prepared from five differentially protected building blocks (**8**–**12**) that will ensure the desired stereochemical outcome during the glycosylations.

**Scheme 2:**
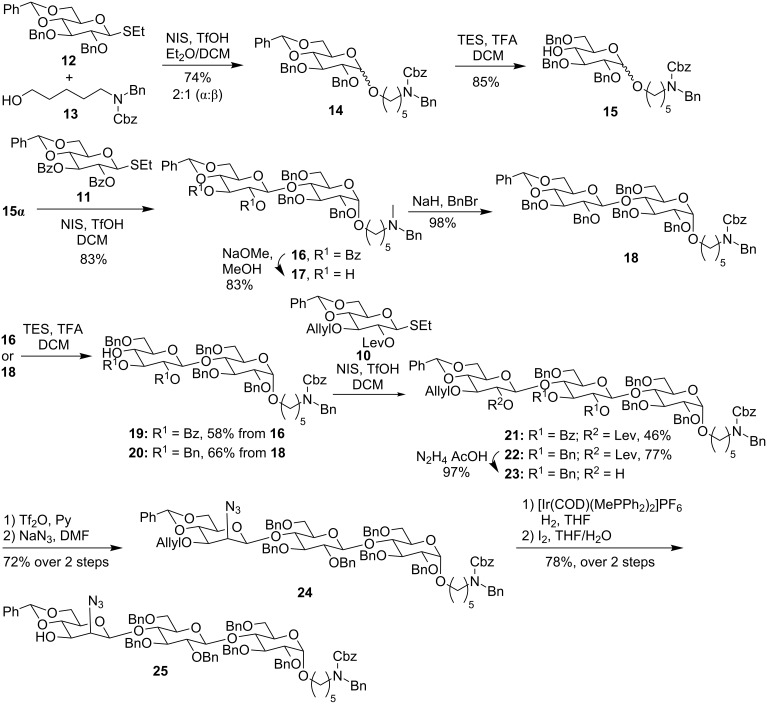
Synthesis of trisaccharide acceptor **25**.

The synthesis of trisaccharide **25** commenced with the union of glucose thioglycoside **12** with C_5_-linker alcohol **13** to yield the corresponding reducing-end monosaccharide **14** equipped with the linker in 70% yield as a mixture of anomers (α:β = 2:1) (Scheme 2). The reductive opening of the benzylidene protecting group in **14** enabled the separation of anomers and furnished acceptor **15α** [28], that was reacted with thioglucoside **11** to yield exclusively the β-disaccharide **16** (Scheme 2). A ring-opening reaction followed by subsequent glycosylation of **19** with orthogonally protected thioglucoside **10** gave trisaccharide **21** in moderate yield. To improve the yield, the nucleophilicity of the disaccharide acceptor **19** (Scheme 2) was altered by replacing the benzoate esters in **16** by a benzyl ether leading to compound **17**. The latter then was converted into the more reactive acceptor **20** via a ring-opening reaction. Glycosylation of **20** with thioglucoside **10** resulted in the desired trisaccharide **22** in almost twice the yield when compared to trisaccharide **21** derived from acceptor **19**. To circumvent the challenging β-mannosylation, the mannosamine unit was installed via the C-2 inversion of glucose at the trisaccharide stage. For that purpose, the C-2 levulinate ester in compound **22** was removed and the resulting secondary alcohol **23** was converted to the azide via a two-step process of triflation and azide substitution to produce the desired trisaccharide **24** [29]. Removal of the allyl group using iridium-catalyzed isomerization and subsequent treatment with iodine in the presence of water yielded trisaccharide acceptor **25** for the late stage [2 + 3] glycosylation.

**Scheme 3:**
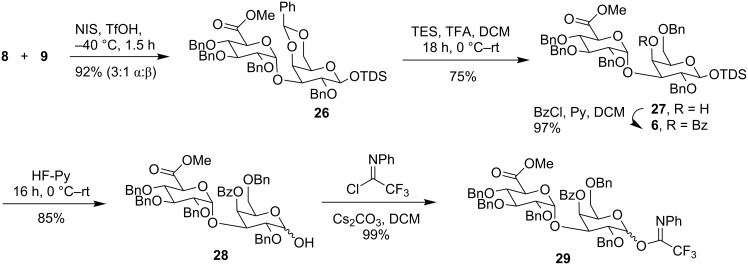
Synthesis of disaccharide **29**.

The synthesis of disaccharide donor **29** started with the glycosylation of glucuronic acid building block **8** with galactose acceptor **9** to obtain disaccharide **26** in 92% yield with 3:1 (α:β) selectivity (Scheme 3). To improve the selectivity for the late stage [2 + 3] glycosylation, disaccharide **26** was modified into compound **6** via benzylidene ring opening followed by benzoylation of the free C-4 secondary hydroxy group in **27**. The disaccharide imidate **29** was synthesized from **6** by anomeric desilylation to afford **28** that was converted to the corresponding imidate in an excellent yield.

**Scheme 4:**
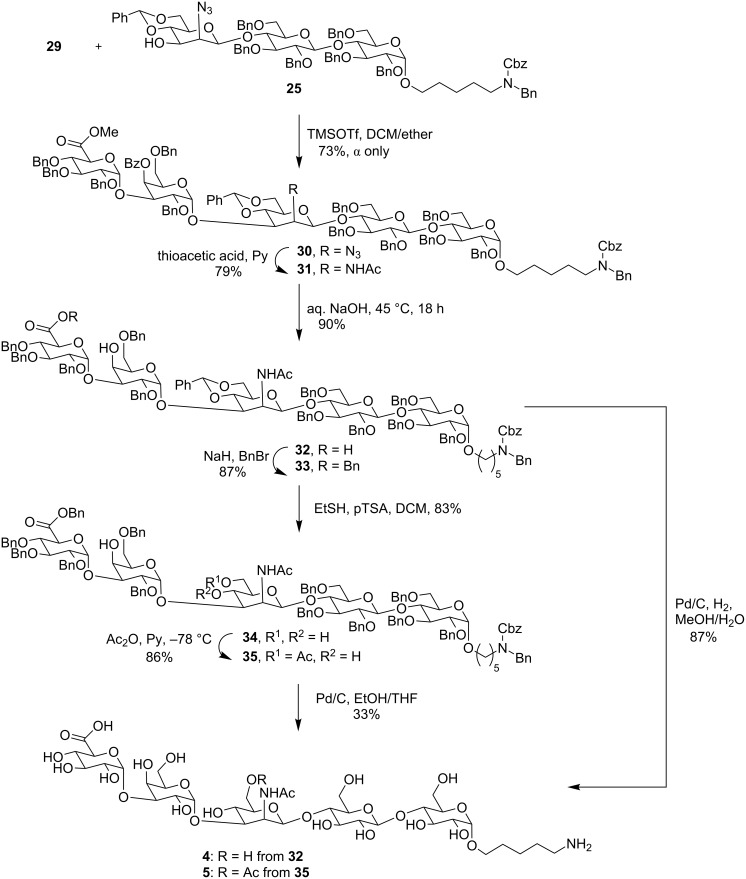
Synthesis of the pentasaccharide repeating unit oligosaccharide antigens without C-6 *O*-acetate (**4**) and with C-6 *O*-acetate (**5**) on Man*p*NAc.

The final [2 + 3] glycosylation of **25** with **29** furnished exclusively the α-anomer of the pentasaccharide **30** in 73% yield (Scheme 4). The subsequent conversion of the azide to acetamide was achieved in one step using thioacetic acid to afford the protected pentasaccharide **31**. The final transformation included the removal of all methyl and benzoate esters under basic conditions to obtain the partially protected pentasaccharide **32** that was hydrogenated using Pd/C and hydrogen to afford pentasaccharide **4** without the acetate group at the C-6 position of the mannosamine.

Pentasaccharide **5**, containing a C-6 *O*-acetate on mannosamine was obtained from **33**, that in turn was a product of the benzylation of **32**. Cleavage of the benzylidene group in **33** yielded **34** that was selectively acetylated at the primary alcohol at low temperature to obtain **35** [30]. The subsequent removal of the benzyl groups using hydrogenation with Pd/C in an EtOH/THF mixture at room temperature for 48 h afforded the desired O-acetylated pentasaccharide **5**. The stereochemistry of each anomeric center during the respective glycosylation was confirmed using the ^1^H coupling constant and using coupled HSQC for the *J*_C1,H1_ coupling (see Supporting Information File 1). The final frame shift 9A and 9V pentasaccharides were compared to the natural reported repeating unit of the CPS. The C6 acetylation of ManNAc was confirmed by comparison of the NMR spectra between the acetylated (9V, compound **5**) and deacetylated (9A compound **4**) synthetic single RU. This data was further confirmed by the work of Moon Nahm et. al that showed the NMR of natural 9V matching with respect to ManNAc C6 acetylation (the observed chemical shift for the methylene H6 were 4.47 and 4.28 ppm, respectively, and for methyl at 2.17 ppm). The ^1^H NMR and HSQC spectra of **5** show the presence of methylene H6 peaks at 4.49 and 4.31 ppm, which in the case of **4**, are present between 3.86–3.94 ppm. Also the new acetyl peak of **5** is seen at 2.18 ppm (Supporting Information File 1).

## Conclusion

Two SP serotype 9V pentasaccharide antigens with and without an acetate group at the C-6 position of Man*p*NAc were synthesized using a convergent [2 + 3] glycosylation strategy. The antigens we prepared will be employed in serological studies using glycan arrays and immunological studies in vivo to probe the significance of the acetate group for immunogenicity and antigenicity.

## Supporting Information

File 1 Experimental procedures.

File 2 NMR Spectra.
