# Supplementary material for: Synthesis of Streptococcus pneumoniae serotype 9V oligosaccharide antigens
Source: Beilstein J Org Chem. 2020 Jul 15;16:1693–9. doi: 10.3762/bjoc.16.140 (PMC7372248; doi:10.3762/bjoc.16.140)
Supplement: File 1 — Experimental procedures. [file Beilstein_J_Org_Chem-16-1693-s001.pdf]

BEILSTEIN JOURNAL OF ORGANIC CHEMISTRY

Supporting Information

for

Synthesis of *Streptococcus pneumoniae* serotype 9V oligosaccharide antigens

Sharavathi G. Parameswarappa, Claney L. Pereira and Peter H. Seeberger

Beilstein J. Org. Chem. **2020,** *16,* 1693–1699. doi:10.3762/bjoc.16.140

Experimental procedures

License and Terms: This is a supporting information file under the terms of the Creative Commons Attribution License (http://creativecommons.org/licenses/by/4.0). Please note that the reuse, redistribution and reproduction in particular requires that the authors and source are credited.
The license is subject to the *Beilstein Journal of Organic Chemistry* terms and conditions: (https://www.beilstein-journals.org/bjoc)

1. General experimental information

Commercial grade solvents were used unless stated otherwise. Dry solvents were obtained from a Waters Dry Solvent System. Solvents for chromatography were distilled prior to use. Sensitive reactions were carried out in heat-dried glassware and under an argon atmosphere. Molecular sieves (4 Å) were activated (dried) in a microwave prior for glycosylation reactions. Analytical thin layer chromatography (TLC) was performed on Kieselgel 60 F254 glass plates precoated with silica gel of 0.25 mm thickness. Spots were visualized by staining with sugar stain (1 mL of 3-methoxyphenol in 1 L of ethanol and 30 mL sulfuric acid) or with Hanessian's stain (5% (w/v) ammonium molybdate, 1% (w/v) cerium(II) sulfate and 10% (v/v) sulfuric acid in water). Silica column chromatography was performed on Fluka Kieselgel 60 (230–400 mesh).

^{1}H, ^{13}C and two-dimensional NMR spectra were measured with Varian 400-MR and/or 600-MR spectrometers. Chemical shifts (δ) are reported in parts per million (ppm) relative to the respective residual solvent peaks ($CDCl_3$: δ 7.26 in ^{1}H and 77.16 in ^{13}C NMR; CD_3OD: δ 3.31 in ^{1}H and 49.0 in ^{13}C NMR; $(CD_3)_2CO$: δ 2.05 in ^{1}H and 29.84 in ^{13}C NMR; D_2O: δ 4.79 in ^{1}H NMR). The following abbreviations were used to indicate peak multiplicities: *s* singlet; *d* doublet; *dd* doublet of doublets; *t* triplet; *dt* doublet of triplets; *q* quartet; *m* multiplet. Coupling constants (*J*) are reported in hertz (Hz). Optical rotation (OR) measurements were carried out with a Schmidt & Haensch UniPol L1000 polarimeter at λ = 589 nm and a concentration (*c*) expressed in g/100 mL in the solvent noted in parentheses. High resolution mass spectrometry (HRMS) was performed at the Free University Berlin, Mass Spectrometry Core Facility, with an Agilent 6210 ESI-TOF mass spectrometer. Infrared (IR) spectra were measured with a Perkin Elmer 100 FTIR spectrometer.

Abbreviations: RM: reaction mixture, rt: room temperature, MW: microwave

2. Experimental procedures

Thexyl 2-*O*-benzyl-4,6-*O*-benzylidene-β-D-galactopyranoside (9)

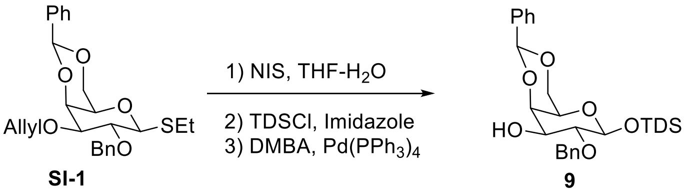

To a solution of thioglycoside donor **SI-1** (1.1 g, 2.485 mmol) [1] in a mixture of THF/water (10 mL:10 mL), NIS (0.615 g, 2.75 mmol) was added. The RM was stirred at rt for 30 min, quenched with aq. $Na_2S_2O_3$ (15 mL, 10% solution), and then extracted with DCM (20 mL × 3). The combined organics were washed with saturated $NaHCO_3$ solution (10 mL), brine (10 mL), dried over Na_2SO_4, filtered, and evaporated in vacuum to obtain a white colored liquid. The crude product was dissolved in DCM (25 mL) and cooled to 0 ˚C. After addition of imidazole (0.338 g, 4.97 mmol) and TDSCl (0.74 mL, 3.73 mmol), the RM was stirred at rt for 14 h. The RM was diluted with DCM (20 mL) and water (20 mL), and the layers were separated. The organic layer was washed with brine (10 mL), dried over Na_2SO_4, filtered, and concentrated to obtain an oil which was purified by silica gel flash column chromatography using 20% EtOAc in hexanes as eluent to obtain the product as white solid (1.1 g, 82%).

The allyl substrate (1.0 g, 1.849 mmol) and *N*,*N*'-dimethylbarbituric acid (DMBA, 0.722 g, 4.62 mmol) were taken in MeOH/THF (15 mL:10 mL) and heated to 45 ˚C for 2 min, degasified, and filled with argon thrice, followed by the addition of 5 mol % $Pd(PPh_3)_4$ (0.107 g, 0.92 mmol), and then degasified and filled with argon (twice). The heating of the yellow colored solution at 50 °C was continued for 16 h. The RM was then cooled to rt and diluted with EtOAc (25 mL), washed with $NaHCO_3$ solution (10 mL), brine (5 mL), dried over Na_2SO_4, filtered, and concentrated in vacuum. Purification by silica gel column chromatography using 40–50% EtOAc in hexanes yielded **9** as brown oil (0.76 g, 82%). ^{1}H NMR (400 MHz, $CDCl_3$) δ 7.65 – 7.10 (m, 10H), 5.54 (s, 1H), 4.99 (d, *J* = 11.2 Hz, 1H), 4.72 (d, *J* = 11.3 Hz, 1H), 4.68 (d, *J* = 7.3 Hz, 1H), 4.26 (dd, *J* = 12.3, 1.5 Hz, 1H), 4.19 (dd, *J* = 3.9, 1.1 Hz, 1H), 4.05 (dd, *J* = 12.3, 1.9 Hz, 1H), 3.80 – 3.68 (m, 1H), 3.55 (dd, *J* = 9.6, 7.4 Hz, 1H), 3.42 – 3.37 (m, 1H), 2.41 (d, *J* = 7.3 Hz, 1H), 1.78–1.68 (m, 1H), 0.92 (dd, *J* = 6.8, 1.8 Hz, 12H), 0.25 (s, 3H), – 0.21 (s, 3H). ^{13}C NMR (101 MHz, $CDCl_3$) δ 138.8, 138.0, 129.3, 128.5, 128.4, 128.0, 127.8, 126.7, 101.5, 98.2, 81.4, 75.7, 75.0, 72.6, 69.5, 66.6, 34.1, 25.1, 20.4, 20.2, 18.8, 18.6, -1.6, -2.5.

2,3-Di-*O*-benzyl-4,6-*O*-benzylidene-αβ-D-glucopyranosyl(1→1)-(2-*N*-benzyl-*N*-benzyloxycarbonylamino)pentanol (14)

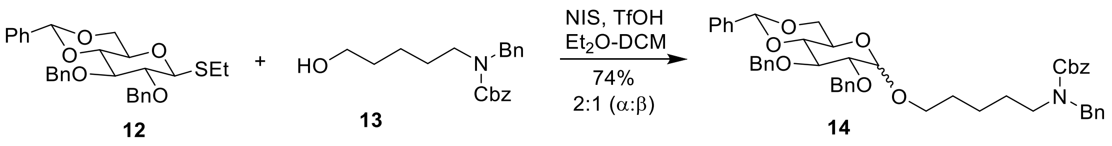

The thioglycoside donor substrate **12** (7.5 g, 15.22 mmol) [2] and C_5 linker amino alcohol acceptor **13** (6.48 g, 19.79 mmol) were dissolved in DCM/ether (25 mL:75 mL), treated with 20 g of dried 4 Å molecular sieves, stirred at rt for 15 min, and then cooled to 0 °C. NIS (3.16 g, 18.27 mmol) and TfOH (0.203 mL, 2.284 mmol) were then added to the RM and stirred at 0 °C for 1 h. The RM was filtered through a celite® bed, washed thoroughly with DCM (25 mL × 4). The filtrate was quenched with 10% $Na_2S_2O_3$ solution (50 mL) and the resulting filtrate was extracted with DCM (25 mL × 3). The organic layer was then washed with brine (10 mL), dried over anhydrous Na_2SO_4, filtered, and concentrated in vacuum to get a pale yellow oily compound, which was purified by silica gel column chromatography using 20–30% EtOAc in hexanes to get the target product **14** as pale yellow transparent gummy liquid as a mixture of α and β-anomers (8.5 g, 74%). $[\alpha]_D^{20}$ = +8.88° (c = 1.0, CH_2Cl_2); IR (thin film, cm^{-1}): νmax : 2931, 2864, 1697, 1087, 1074. HRMS (ESI): calculated for $C_{47}H_{51}NNaO_8$ $[M + Na]^+$, 780.3512, found 780.3535. ^{1}H NMR (400 MHz, $CDCl_3$) δ 7.73 – 6.96 (m, 25H), 5.57 (m, 1H), 5.28 – 5.11 (m, 2H), 4.93 (dd, *J* = 11.3, 4.4 Hz, 1H), 4.84 (dd, *J* = 11.5, 3.5 Hz, 2H), 4.80 – 4.66 (m, 1.4H), 4.52- 4.48 (m, 2.6H), 4.35 (dd, *J* = 10.4, 5.0 Hz, 0.4H), 4.26 ((dd, *J* = 10.1, 4.7 Hz, 0.6H), 4.05 (t, *J* = 9.3 Hz, 1H), 3.94 – 3.13 (m, 8H), 1.75 – 1.46 (m, 4H), 1.37- 1.27 (m, 2H). ^{13}C NMR (101 MHz, $CDCl_3$) δ 156.8, 156.3, 138.9, 138.6, 138.5, 138.4, 138.0, 137.5, 137.4, 137.0, 129.9, 129.1 (2C), 129.0, 128.7, 128.5 (2C), 128.4, 128.3, 128.1 (2C), 128.0, 127.8, 127.7 (2C), 127.4, 126.1, 104.2, 101.3, 101.2, 98.1, 82.4, 82.2, 81.6, 81.0, 79.5, 78.7, 75.5, 75.2, 73.6, 73.0, 70.4, 69.2, 68.9, 68.4, 67.3, 66.1, 62.5, 50.7, 50.4, 47.3, 46.3, 29.6, 29.3, 28.1, 27.7, 23.54, 23.5.

2,3,6-Tri-*O*-benzyl-α-D-glucopyranosyl(1→1)-(5-*N*-benzyl-*N*-benzyloxycarbonylamino)pentanol (15)

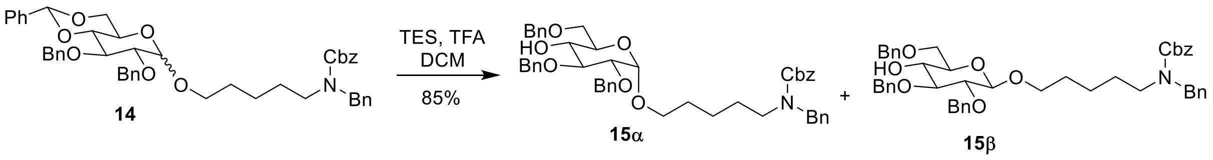

Analogous as described in [3]. Benzylidene substrate **14** (14.0 g, 18.47 mmol, mixture of α and β-anomers) was taken in DCM (300 mL) under argon and stirred in the presence of activated 4 Å molecular sieves for 10 min before cooling to 0 °C. After the addition of triethylsilane (23.60 mL, 148.0 mmol) followed by TFA (8.54 mL, 111.0 mmol) dropwise, the RM was stirred at rt for 16 h before quenching with water (100 mL). The aqueous phase was extracted with DCM (30 mL × 3). The combined organics were washed thoroughly with water (20 mL × 3), brine (20 mL), dried over anhydrous Na_2SO_4, filtered, and evaporated in vacuum to get a colorless oil which was purified by silica gel column chromatography using 20–25% EtOAc in hexanes to get **15** (12.0 g, 85%) as colorless oil [(*α*/*β* = 1.75:1, **15** *α*-anomer (7.64 g), **15** *β*-anomer (4.4 g))]. *α*-anomer: $[\alpha]_D^{20}$ = +39.17° (c = 0.5, CH_2Cl_2); IR (thin film, cm^{-1}): νmax : 3461, 2922, 1698, 1058. HRMS (ESI): calculated for $C_{47}H_{53}NNaO_8$ $[M + Na]^+$, 782.3669, found 782.3664. ^1H NMR (400 MHz, $CDCl_3$) δ 7.51 – 7.04 (m, 25H), 5.17 (d, *J* = 16.9 Hz, 2H), 5.00 (d, *J* = 11.4 Hz, 1H), 4.80 – 4.42 (m, 8H), 3.93 – 3.04 (m, 10H), 2.54 – 2.38 (m, 1H), 1.70 – 1.43 (m, 4H), 1.39 – 1.13 (m, 2H). ^{13}C NMR (101 MHz, $CDCl_3$) δ 156.8, 156.3, 139.0, 138.3, 138.2, 138.0, 137.0, 136.8, 128.7, 128.6, 128.5, 128.4, 128.1, 128.0, 127.96, 127.7, 127.4, 127.3, 97.0, 81.7, 79.9, 75.5, 73.7, 73.0, 71.0, 70.2, 69.6, 68.1, 67.3, 50.6, 50.4, 47.4, 46.3, 29.2, 28.1, 27.7, 23.8, 23.6. *β*-anomer: ^1H NMR (400 MHz, $CDCl_3$) δ 7.57 – 6.81 (m, 25H), 5.16 (d, *J* = 10.3 Hz, 2H), 5.02 – 4.79 (m, 2H), 4.78 – 4.66 (m, 2H), 4.64 – 4.30 (m, 5H), 4.03 – 3.32 (m, 8H), 3.29 – 3.09 (m, 2H), 2.53 (d, *J* = 2.1 Hz, 1H), 1.72 – 1.45 (m, 4H), 1.39 – 1.27 (m, 2H). ^{13}C NMR (101 MHz, $CDCl_3$) δ 138.7, 138.5, 138.0, 129.2, 128.7, 128.6, 128.5, 128.4, 128.1 (2C), 128.0, 127.9, 127.8, 127.3, 125.4, 103.8, 84.1, 81.8, 75.4, 74.8, 74.1, 73.8, 71.8, 70.5, 70.0, 67.3, 50.6, 50.3, 47.3, 46.3, 29.6, 28.1, 27.7, 23.5.

2,3-Di-*O*-benzoyl-4,6-*O*-benzylidene-β-D-glucopyranosyl-(1→4)-2,3,6-tri-*O*-benzyl-α-D-glucopyranosyl-(1→1)-(5-*N*-benzyl-*N*-benzyloxycarbonylamino)pentanol (16)

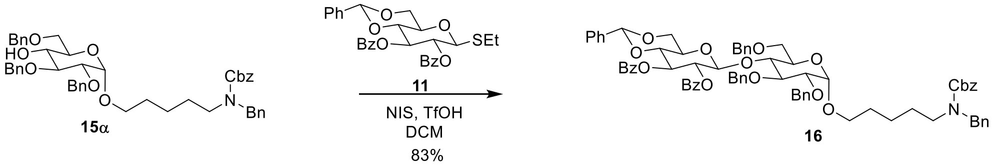

The acceptor substrate **15α** (6.0 g, 7.9 mmol) and thioglycoside donor substrate **11** (5.06 g, 9.71 mmol) [2] were taken in DCM (150 mL), and treated with 10 g of MW-dried 4 Å molecular sieves, stirred at rt for 15 min and then cooled to −10 °C. NIS (2.13 g, 9.47 mmol) and TfOH (0.105 mL, 1.184 mmol) were then added to the RM and stirred at −10 °C to 0 °C for 3 h. The RM was filtered through a celite® bed and washed thoroughly with DCM (25 mL × 3). The filtrate was quenched with 10% $Na_2S_2O_3$ solution (50 mL) and the aqueous phase was extracted with DCM (25 mL × 3). The combined organic layer was then washed with $NaHCO_3$ solution (100 mL), brine (50 mL), dried over anhydrous Na_2SO_4, filtered, and concentrated in vacuum to get a pale yellow oily compound, which was purified by silica gel column chromatography using 25–100% EtOAc in hexanes to get **16** (8 g, 83%) as colorless transparent gummy liquid. $[\alpha]_D^{20}$ = +22.36° (c = 1.00, CH_2Cl_2); IR (thin film, cm^{-1}): νmax: 2868, 1733, 1699, 1270, 1094. HRMS (ESI): calculated for $C_{74}H_{75}NNaO_{15}$ $[M + Na]^+$, 1240.5034, found 1240.5048. ^1H NMR (400 MHz, $CDCl_3$) δ 7.98 – 7.79 (m, 4H), 7.55 – 7.14 (m, 36H), 5.56 (t, *J* = 9.6 Hz, 1H), 5.45 (s, 1H), 5.39 (dd, *J* = 9.5, 8.0 Hz, 1H), 5.18 (d, *J* = 7.1 Hz, 2H), 4.91 (dd, *J* = 40.7, 10.9 Hz, 2H), 4.78 – 4.55 (m, 5H), 4.48 (d, *J* = 7.2 Hz, 2H), 4.29 (d, *J* = 12.1 Hz, 1H), 4.22 (dd, *J* = 10.5, 4.9 Hz, 1H), 3.99 – 3.90 (m, 1H), 3.81 (dt, *J* = 22.2, 9.4 Hz, 2H), 3.63 (dd, *J* = 10.7, 2.8 Hz, 1H), 3.59 – 3.12 (m, 9H), 1.65 – 1.42 (m, 4H), 1.31 – 1.12 (m, 2H). ^{13}C NMR (101 MHz, $CDCl_3$) δ 165.6, 165.1, 156.8, 156.2, 139.5, 138.5, 138.0, 137.8, 137.0, 133.4, 133.2, 129.9, 129.8, 129.5, 129.2 (2C), 128.9, 128.7, 128.6, 128.5, 128.4 (2C), 128.3 (2C), 128.0 (2C), 127.8, 127.6, 127.4, 126.3, 101.5, 100.9, 97.0, 80.0, 79.3, 79.0, 75.4, 73.7, 73.3, 73.0, 72.3, 69.7, 68.7, 68.1, 67.6, 67.3, 66.4, 50.6, 50.4, 47.2, 46.3, 29.0, 28.0, 27.6, 23.4.

4,6-*O*-Benzylidene-β-D-glucopyranosyl-(1→4)-2,3,6-tri-*O*-benzyl-α-D-glucopyranosyl-(1→1)-(5-*N*-benzyl-*N*-benzyloxycarbonylamino)pentanol (17)

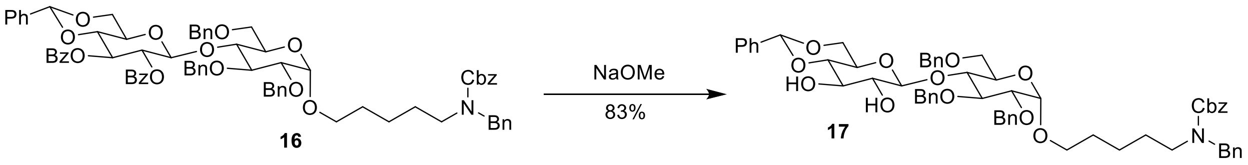

Substrate **16** (4.0 g, 3.28 mmol) was taken in methanol/THF (40 mL:40 mL) at rt, treated with methanolic NaOMe (0.5 M solution, 16.42 mL, 8.21 mmol), and stirred at rt for 18 h. The RM was evaporated in vacuum and the residue was taken in DCM and water (100 mL each). After extraction with DCM (50 mL × 3), the combined organics were dried over Na_2SO_4, filtered, and evaporated in vacuum. Purification by silica gel column chromatography using 45–55% EtOAc in hexanes afforded colorless gummy disaccharide **17** (2.75 g, 83%). $[\alpha]_D^{20}$ = +48.05° (c = 1.00, CH_2Cl_2); IR (thin film, cm^{-1}): νmax : 2955, 2866, 1749, 1091. HRMS (ESI): calculated for $C_{60}H_{67}NNaO_{13}$ $[M + Na]^+$, 1032.4510, found 1032.4505. ^1H NMR (400 MHz, $CDCl_3$) δ 7.58 – 7.04 (m, 30H), 5.44 (s, 1H), 5.17 (d, *J* = 14.6 Hz, 2H), 4.97 – 4.81 (m, 2H), 4.79 – 4.64 (m, 3H), 4.63 – 4.41 (m, 5H), 4.07 – 3.87 (m, 5H), 3.78 (dd, *J* = 18.1, 9.6 Hz, 1H), 3.69 – 3.18 (m, 10H), 3.10 (td, *J* = 9.8, 5.0 Hz, 1H), 2.64 (d, *J* = 16.9 Hz, 1H), 1.66 – 1.46 (m, 4H), 1.43 – 1.24 (m, 2H). ^{13}C NMR (101 MHz, $CDCl_3$) δ 157.0, 156.4, 139.3, 138.4, 137.9, 137.6, 137.2, 129.4, 128.7, 128.6, 128.5, 128.5, 128.3, 128.2, 128.1, 128.0, 127.5, 127.3, 126.5, 103.6, 101.9, 97.0, 80.8, 80.5, 79.7, 77.6, 75.6, 75.4, 73.8, 73.5, 73.2, 69.8, 68.8, 68.5, 68.2, 67.7, 67.4, 66.4, 50.5, 50.2, 47.2, 46.0, 29.2, 28.0, 27.5, 23.8.

2,3-Di-*O*-benzyl-4,6-*O*-benzylidene-β-D-glucopyranosyl-(1→4)-2,3,6-tri-*O*-benzyl-α-D-glucopyranosyl-(1→1)-(5-*N*-benzyl-*N*-benzyloxycarbonylamino)pentanol (18)

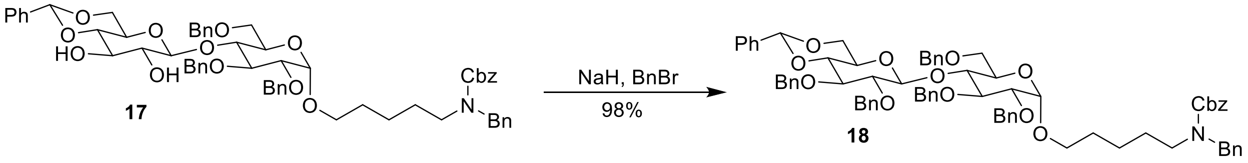

Diol substrate **17** (5.8 g, 5.74 mmol.) was taken in dry DMF (60 mL) at 0 °C, treated with NaH (60 wt %, 0.92 g, 22.97 mmol), and stirred at 0 °C for 35 min. Then, BnBr (2.049 mL, 17.22 mmol) was added to the RM at 0 °C. The RM was warmed to rt and further stirred for 24 h. The reaction was then quenched with water (150 mL) and extracted with EtOAc (100 mL × 3). The combined organics were washed thoroughly with water (50 mL × 3), brine (20 mL), dried over anhydrous Na_2SO_4, filtered, and evaporated in vacuum to get a gummy oil. Purification by silica gel column chromatography using 25–40% EtOAc in hexanes afforded product **18** as white oil after evaporation (6.7g, 98%). $[\alpha]_D^{20}$ = +22.28° (c = 1.00, CH_2Cl_2); IR (thin film, cm^{-1}): νmax : 2866, 1698, 1086. HRMS (ESI): calculated for $C_{74}H_{79}NNaO_{13}$ $[M + Na]^+$, 1212.5449, found 1212.5449. ^{1}H NMR (400 MHz, $CDCl_3$) δ 7.65 – 7.01 (m, 40H), 5.51 (s, 1H), 5.19 (d, *J* = 9.0 Hz, 2H), 4.92 (t, *J* = 11.4 Hz, 2H), 4.85 – 4.66 (m, 6H), 4.66 – 4.54 (m, 2H), 4.50 (d, *J* = 9.7 Hz, 2H), 4.39 (d, *J* = 7.8 Hz, 1H), 4.31 (d, *J* = 12.0 Hz, 1H), 4.20 (dd, *J* = 10.5, 5.0 Hz, 1H), 4.01 – 3.91 (m, 1H), 3.91 – 3.75 (m, 2H), 3.73 – 2.96 (m, 12H), 1.73 – 1.45 (m, 4H), 1.42 – 1.17 (m, 2H). ^{13}C NMR (101 MHz, $CDCl_3$) δ 156.8, 156.3, 139.5, 138.6 (2C), 138.5, 138.0, 137.6, 137.0, 129.1, 128.7, 128.6, 128.5, 128.4 (2C), 128.3, 128.2 (2C), 128.1, 128.0, 127.9 (2C), 127.7 (2C), 127.4, 127.3, 127.1, 126.2, 103.0, 101.2, 97.1, 82.6, 81.8, 81.1, 80.3, 79.2, 77.1, 75.5 (2C), 75.0, 73.5, 73.4, 70.1, 69.0, 68.1, 67.8, 67.3, 65.8, 50.6, 50.3, 47.3, 46.3, 29.2, 28.1, 27.7, 23.6.

2,3-Di-*O*-benzoyl-6-*O*-benzyl-β-D-glucopyranosyl-(1→4)-2,3,6-tri-*O*-benzyl-α-D-glucopyranosyl-(1→1)-(5-*N*-benzyl-*N*-benzyloxycarbonylamino)pentanol (19)

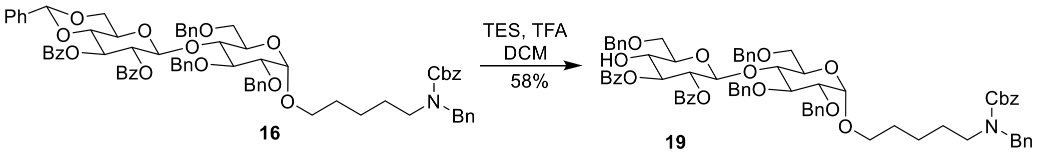

Analogous as described in [3]. Benzylidene substrate **16** (0.6 g, 0.49 mmol) was dissolved in DCM (15 mL) and stirred with activated 4 Å molecular sieves for 10 min before cooling to 0 °C. Triethylsilane (0.63 mL, 3.94 mmol) was added, followed by TFA (0.23 mL, 2.95 mmol) dropwise, and the RM was stirred at rt for 16 h before quenching with water. The aqueous layer was extracted with DCM (20 mL × 3), the combined organics were washed thoroughly with water (20 mL × 3), brine (20 mL), dried over anhydrous Na_2SO_4, filtered, and evaporated in vacuum to get a colorless oil which was purified by silica gel column chromatography using 30–100% EtOAc in hexanes to get **19** as colorless oil (0.35 g, 58%). $[\alpha]_D^{20}$ = +52.46° (c = 0.5, CH_2Cl_2); IR (thin film, cm^{-1}): νmax: 3452, 2938, 2867, 1732, 1698, 1273, 1094. HRMS (ESI): calculated for $C_{74}H_{77}NNaO_{15}$ $[M + Na]^+$, 1242,5191, found 1242.5232. ^{1}H NMR (400 MHz, $CDCl_3$) δ 8.01 – 7.90 (m, 2H), 7.87 – 7.76 (m, 2H), 7.58 – 7.10 (m, 36H), 5.39 – 5.22 (m, 2H), 5.16 (br s, 2H), 4.99 (d, *J* = 11.3 Hz, 1H), 4.83 (d, *J* = 11.2 Hz, 1H), 4.77 – 4.52 (m, 5H), 4.51 – 4.37 (m, 4H), 4.27 (d, *J* = 12.1 Hz, 1H), 3.96 – 3.76 (m, 3H), 3.67 – 3.09 (m, 12H), 1.53 (d, *J* = 39.4 Hz, 4H), 1.30 – 1.06 (m, 2H). ^{13}C NMR (101 MHz, $CDCl_3$) δ 166.9, 165.2, 139.8, 138.6, 137.9, 137.7, 133.4 (2C), 130.0, 129.8, 129.4 (2C), 128.8, 128.7, 128.6 (2C), 128.5 (3C), 128.4, 128.3, 128.2, 128.1, 128.0 (3C), 127.8, 127.79, 127.5, 127.2, 100.5, 97.0, 80.2, 79.4, 77.4, 76.4, 75.2, 73.9, 73.7, 73.4, 72.2 (2C), 70.9, 69.7, 67.9, 67.3, 50.6, 47.3, 46.3, 29.1, 23.4.

2,3,6-Tri-*O*-benzyl-β-D-glucopyranosyl-(1→4)-2,3,6-tri-*O*-benzyl-α-D-glucopyranosyl-(1→1)-(5-*N*-benzyl-*N*-benzyloxycarbonylamino)pentanol (20)

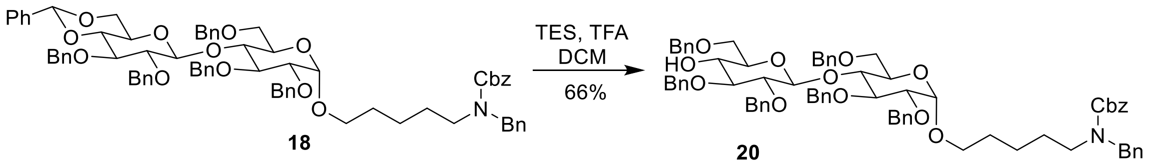

Analogous as described in [3]. Benzylidene enzylidene substrate **18** (6.5 g, 5.46 mmol) was dissolved in DCM (25 mL) and stirred with activated 4 Å molecular sieves (20 g) for 10 min before cooling to 0 °C. Triethylsilane (6.98 mL, 43.7 mmol) was added, followed by TFA (2.52 mL, 32.8 mmol) dropwise, and the RM was stirred at rt for 16 h before quenching with water. The aqueous layer was extracted with DCM (30 mL × 3), the combined organics were washed thoroughly with water (20 mL × 3), brine (20 mL), dried over anhydrous Na_2SO_4, filtered, and evaporated in vacuum to get a colorless oil which was purified by silica gel column chromatography using 35–40% EtOAc in hexanes to obtain as **20** colorless oil (4.3 g, 66%). $[\alpha]_D^{20}$ = +29.71° (c = 1.00, CH_2Cl_2); IR (thin film, cm^{-1}): νmax : 3467, 2867, 1698, 1067. HRMS (ESI): calculated for $C_{74}H_{81}NNaO_{13}$ $[M + Na]^+$, 1214.5606, found 1214.5615. ^1H NMR (400 MHz, $CDCl_3$) δ 7.66 – 6.89 (m, 40H), 5.19 (d, *J* = 7.9 Hz, 2H), 4.99 (d, *J* = 11.0 Hz, 1H), 4.92 – 4.66 (m, 7H), 4.61 (d, *J* = 12.1 Hz, 2H), 4.54 – 4.44 (m, 3H), 4.43 – 4.31 (m, 3H), 4.02 – 3.91 (m, 1H), 3.90 – 3.79 (m, 2H), 3.73 – 3.40 (m, 7H), 3.42 – 3.13 (m, 6H), 2.95 (d, *J* = 1.8 Hz, 1H), 1.76 – 1.50 (m, 4H), 1.41 – 1.17 (m, 2H). ^{13}C NMR (101 MHz, $CDCl_3$) δ 156.8, 156.2, 139.7, 138.9, 138.6, 138.0, 136.8, 128.6 (2C), 128.5, 128.4, 128.3, 128.2, 128.1, 128.0 (2C), 127.9 (2C), 127.8 (2C), 127.7, 127.6, 127.4, 127.3, 127.2, 102.6, 97.1, 84.3, 82.1, 80.4, 79.2, 76.8, 75.3, 74.9, 73.8, 73.7, 73.4, 73.1, 71.4, 70.1, 67.9, 67.3, 50.6, 50.3, 47.2, 46.3, 29.2, 28.1, 27.7, 23.6.

3-*O*-Allyl-4,6-*O*-benzylidene-2-*O*-levulinoyl-β-D-glucopyranosyl-(1→4)-2,3-di-*O*-benzoyl-6-benzyl-β-D-glucopyranosyl-(1→4)-2,3,6-tri-*O*-benzyl-α-D-glucopyranosyl-(1→1)-(5-*N*-benzyl-*N*-benzyloxycarbonylamino)pentanol (21)

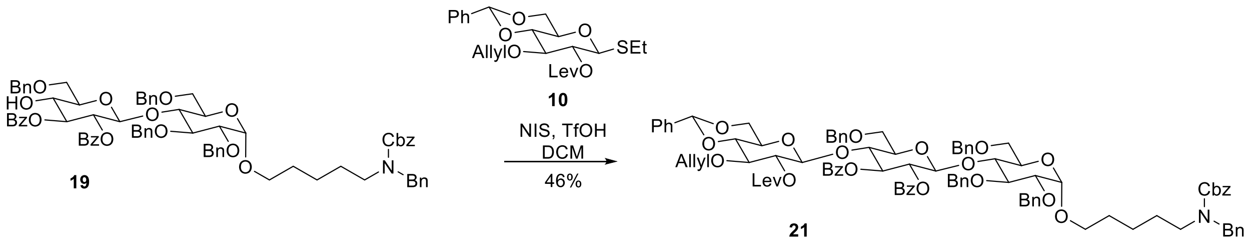

The acceptor substrate **19** (0.25 g, 0.21 mmol) and thioglycoside donor substrate **10** (0.28 g, 0.62 mmol) [4] were taken in DCM (15 mL), treated with 1 g of dried 4 Å molecular sieves, stirred at rt for 15 min, and then cooled to −10 °C. NIS (0.13 g, 0.594 mmol) and TfOH (0.02 mL, 0.21 mmol) were then added to the RM and stirred at −10 °C to 0 °C for 3 h. The RM was filtered through a celite® bed, washed thoroughly with DCM (25 mL × 3). The filtrate was quenched with 10% $Na_2S_2O_3$ solution (50 mL). The aqueous layer was extracted with DCM (10 mL × 3). The combined organic layer was then washed with $NaHCO_3$ solution (50 mL), brine (50 mL), dried over anhydrous Na_2SO_4, filtered, and concentrated in vacuum to obtain a pale yellow oil, which was purified by silica gel column chromatography using 25–100% EtOAc in hexanes to obtain **21** as colorless gummy liquid (0.15 g, 46%) which was contaminated with hydrolyzed donor impurity. ^{1}H NMR (400 MHz, $CDCl_3$) δ 8.08 – 7.90 (m, 2H), 7.87 – 7.73 (m, 2H), 7.60 – 7.09 (m, 41H), 5.82 (ddt, *J* = 17.3, 10.6, 5.4 Hz, 1H), 5.39 – 5.26 (m, 2H), 5.25 – 5.01 (m, 6H), 4.85 (dd, *J* = 9.0, 7.9 Hz, 1H), 4.78 (d, *J* = 11.7 Hz, 1H), 4.71 (dd, *J* = 12.2, 4.5 Hz, 2H), 4.65 (br s, 1H), 4.60 – 4.44 (m, 5H), 4.37 (d, *J* = 8.0 Hz, 1H), 4.33 – 4.20 (m, 3H), 4.12 – 3.99 (m, 2H), 3.89 (dt, *J* = 24.6, 9.0 Hz, 2H), 3.79 – 3.66 (m, 2H), 3.63 (dd, *J* = 10.4, 2.9 Hz, 1H), 3.54 (dd, *J* = 10.6, 4.9 Hz, 1H), 3.50 – 3.42 (m, 4H), 3.40 – 3.08 (m, 6H), 3.02 – 2.90 (m, 1H), 2.87 – 2.63 (m, 2H), 2.57 – 2.42 (m, 3H), 2.18 (s, 3H), 1.62 – 1.41 (m, 4H), 1.38 – 1.06 (m, 2H).

3-*O*-Allyl-4,6-*O*-benzylidene-2-*O*-levulinoyl-β-D-glucopyranosyl-(1→4)-2,3,6-tri-*O*-benzyl-β-D-glucopyranosyl-(1→4)-2,3,6-tri-*O*-benzyl-α-D-glucopyranosyl-(1→1)-(5-*N*-benzyl-*N*-benzyloxycarbonylamino)pentanol (22)

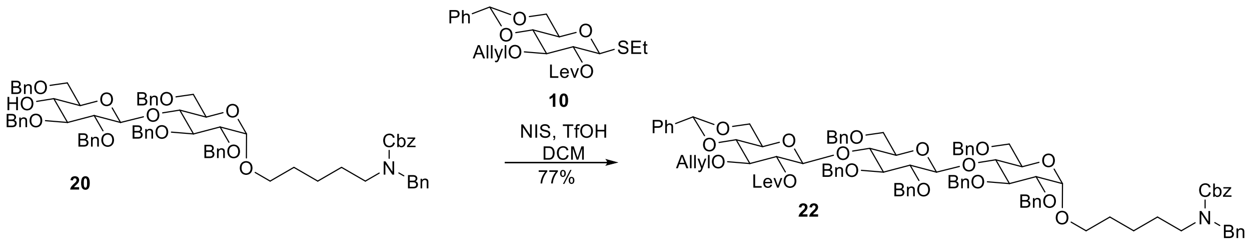

Acceptor **20** (2.0 g, 1.677 mmol) and thioglycoside donor **10** (1.51 g, 3.35 mmol) [4] were taken in DCM (50 mL), treated with 1 g of dried AW 4 Å molecular sieves, stirred at rt for 15 min, and then cooled to −5 °C. NIS (0.604 g, 2.68 mmol) and TfOH (0.03 mL, 0.335 mmol) were then added to the RM at −7 °C to 0 °C, and then stirred at 0 °C for 1 h. The reaction was quenched with 10% $Na_2S_2O_3$ solution, filtered, extracted using DCM (10 mL × 3). The combined organics were then washed with brine (10 mL), dried over Na_2SO_4, and evaporated in vacuum to obtain the crude as brown colored residue. Purification by silica gel column chromatography using 30% EtOAc in hexanes afforded trisaccharide **22** as colorless gummy liquid (2.05 g, 77%). $[\alpha]_D^{20}$ = +18.34° (c = 1.00, CH_2Cl_2); IR (thin film, cm^{-1}): νmax : 2867, 1751,1698, 1092. HRMS (ESI): calculated for $C_{95}H_{105}NNaO_{20}$ $[M + Na]^+$, 1602.7128, found 1602.7038. ^{1}H NMR (400 MHz, $CDCl_3$) δ 7.53 – 7.12 (m, 45H), 5.86 (ddt, *J* = 17.2, 10.7, 5.5 Hz, 1H), 5.44 (s, 1H), 5.31 – 5.05 (m, 5H), 4.99 – 4.82 (m, 2H), 4.81 – 4.57 (m, 8H), 4.55 (d, *J* = 8.0 Hz, 1H), 4.52 – 4.41 (m, 3H), 4.37 – 4.28 (m, 2H), 4.25 (d, *J* = 7.5 Hz, 1H), 4.24 (d, *J* = 12.0 Hz, 1H), 4.17 – 4.03 (m, 2H), 3.92 (ddd, *J* = 10.0, 8.7, 1.6 Hz, 2H), 3.88 – 3.78 (m, 2H), 3.69 (d, *J* = 2.4 Hz, 2H), 3.62 – 3.04 (m, 14H), 2.88 – 2.67 (m, 2H), 2.63 – 2.43 (m, 2H), 2.15 (s, 3H), 1.57 (d, *J* = 21.4 Hz, 4H), 1.40 – 1.25 (m, 2H). ^{13}C NMR (101 MHz, $CDCl_3$) δ 206.0, 171.3, 156.8, 156.2, 139.9, 139.2, 138.8, 138.6, 138.4, 138.0, 137.8, 137.3, 134.9, 129.1, 128.7, 128.6 (3C), 128.5, 128.4, 128.3, 128.2, 128.1 (3C), 128.0, 127.9 (3C), 127.8 (2C), 127.7 (2C), 127.6, 127.4 (2C), 127.2, 127.0, 126.1, 116.7, 102.7, 101.2, 100.6, 97.1, 82.7, 82.0, 81.5, 80.7, 79.0, 78.7, 77.2, 75.4, 75.3, 74.8, 73.7, 73.5, 73.4 (2C), 73.3, 70.1, 68.7, 68.0, 67.6, 67.2, 66.0, 50.6, 50.3, 47.2, 46.3, 37.8, 29.9, 29.2, 28.1, 28.0, 27.7, 23.5.

3-*O*-Allyl-4,6-*O*-benzylidene-β-D-glucopyranosyl-(1→4)-2,3,6-tri-*O*-benzyl-β-D-glucopyranosyl-(1→4)-2,3,6-tri-*O*-benzyl-α-D-glucopyranosyl-(1→1)-(5-*N*-benzyl-*N*-benzyloxycarbonylamino)pentanol (23)

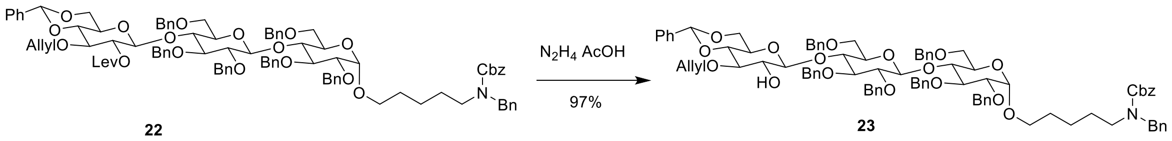

Substrate **22** (3.4 g, 2.151 mmol) was taken in a mixture of DCM/EtOH/toluene (10 mL:10 mL:20 mL) at 0 °C, treated with hydrazine acetate (0.40 g, 4.3 mmol), stirred at 0 °C for 30 min, slowly warmed to rt, and further stirred at rt for 18 h. The reaction was quenched with acetone (1 mL), stirred for 30 min, and concentrated in vacuum to obtain the crude, which was purified by silica gel column chromatography using 20–30% EtOAc in hexanes to yield **23** as colorless gummy liquid (3.1 g, 97%). $[\alpha]_D^{20}$ = +29.62° (c = 1.00, CH_2Cl_2); IR (thin film, cm^{-1}): νmax : 3456, 2867, 1698, 1068. HRMS (ESI): calculated for $C_{90}H_{99}NNaO_{18}$ $[M + Na]^+$, 1504.6760, found 1504.6784. ^1H NMR (400 MHz, $CDCl_3$) δ 7.56 – 7.11 (m, 45H), 5.95 (ddt, *J* = 16.5, 11.1, 5.7 Hz, 1H), 5.45 (s, 1H), 5.31 (dd, *J* = 17.4, 1.9 Hz, 1H), 5.22 – 5.13 (m, 3H), 5.08 (d, *J* = 11.5 Hz, 1H), 4.95 – 4.68 (m, 7H), 4.64 – 4.35 (m, 10H), 4.25 (dd, *J* = 12.8, 5.9 Hz, 1H), 4.06 – 3.83 (m, 5H), 3.79 (dd, *J* = 11.5, 3.4 Hz, 1H), 3.72 – 3.63 (m, 2H), 3.60 – 3.31 (m, 11H), 3.30 – 3.14 (m, 3H), 3.09 (td, *J* = 9.6, 5.0 Hz, 1H), 1.66 – 1.48 (m, 4H), 1.38 – 1.15 (m, 2H). ^{13}C NMR (101 MHz, $CDCl_3$) δ 156.8, 156.2, 139.7, 139.1, 138.6, 138.5, 138.0, 137.8, 137.4, 135.1, 129.1, 129.0, 128.6, 128.6, 128.5, 128.5, 128.4, 128.33, 128.3, 128.1(2C), 128.0, 127.9 (3C), 127.8 (2C), 127.7 (2C), 127.5, 127.3, 127.2, 127.0, 126.1, 117.3, 103.7, 102.7, 101.2, 97.0, 83.7, 82.4, 81.3, 80.5, 80.1, 79.2, 78.1, 76.8, 75.3, 75.2 (2C), 75.0, 74.4, 73.6, 73.5, 73.4, 73.3, 70.1, 68.8, 68.7, 68.1, 67.3, 66.4, 50.6, 50.3, 47.3, 46.3, 29.2, 28.1, 27.7, 23.6.

3-*O*-Allyl-2-azido-4,6-*O*-benzylidene-2-deoxy-β-D-mannopyranosyl-(1→4)-2,3,6-tri-*O*-benzyl-β-D-glucopyranosyl-(1→4)-2,3,6-tri-*O*-benzyl-α-D-glucopyranosyl-(1→1)-(5-*N*-benzyl-*N*-benzyloxycarbonylamino)pentanol (24)

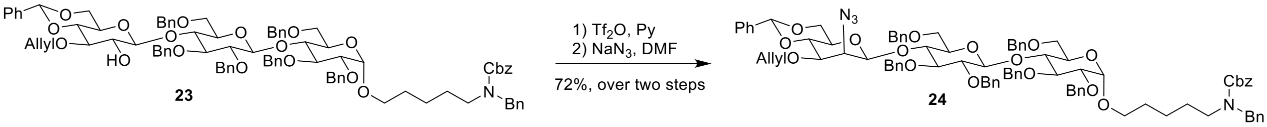

Substrate **23** (1.5 g, 1.012 mmol) was taken in DCM (35 mL) at rt, treated with pyridine (0.409 mL, 5.06 mmol), followed by Tf_2O (0.513 mL, 3.03 mmol), and stirred at rt for 30 min. The reaction was then quenched with water (10 mL) and extracted with DCM (25 mL × 3). The combined organics were washed with aq. $NaHCO_3$ solution (2 mL), brine (3 mL), dried over anhydrous Na_2SO_4, filtered, and concentrated in vacuum to obtain the triflate as brown oil (1.7 g) that was directly subjected to the next step. HRMS (ESI): calculated for $C_{91}H_{98}F_3NNaO_{20}S$ $[M + Na]^+$, 1636.6253, found 1636.6283.

The triflate (1.7 g) was taken in dry DMF (15 mL) and treated with NaN_3 (0.2 g, 3.03 mmol). The RM was heated at 65 °C overnight, cooled to rt, quenched with water (200 mL), and the aqueous layer extracted with EtOAc (50 mL × 5). The combined organics were washed with water (50 × 3 mL), brine (50 mL), dried over anhydrous Na_2SO_4, filtered, and concentrated in vacuum to obtain a brown-colored crude, which was purified by silica gel column chromatography using 30–40% EtOAc in hexanes to obtain product **24** as colorless oil (1.1 g, 72%, over two steps). $[\alpha]_D^{20}$ = +8.46° (c = 1.00, CH_2Cl_2); IR (thin film, cm^{-1}): νmax : 2866, 2108, 1698, 1090. HRMS (ESI): calculated for $C_{90}H_{98}N_4NaO_{17}$ $[M + Na]^+$, 1529.6825, found 1529.6863. ^1H NMR (400 MHz, $CDCl_3$) δ 7.53 – 7.13 (m, 45H), 5.87 (ddt, *J* = 17.4, 10.6, 5.4 Hz, 1H), 5.48 (s, 1H), 5.29 (dd, *J* = 17.2, 1.7 Hz, 1H), 5.25 – 5.16 (m, 3H), 5.11 (d, *J* = 11.5 Hz, 1H), 5.00 (d, *J* = 10.6 Hz, 1H), 4.82 – 4.74 (m, 5H), 4.73 – 4.71 (m, 1H), 4.68 (d, *J* = 1.2 Hz, 1H), 4.62 (dd, *J* = 12.2, 9.4 Hz, 2H), 4.48 (dd, *J* = 14.1, 10.3 Hz, 3H), 4.34 (d, *J* = 10.7 Hz, 1H), 4.33 (d, *J* = 9.1 Hz, 1H), 4.26 (d, *J* = 11.9 Hz, 1H), 4.18 (ddt, *J* = 13.1, 5.2, 1.5 Hz, 1H), 4.09 – 3.77 (m, 8H), 3.70 – 3.16 (m, 14H), 3.04 (td, *J* = 9.7, 4.9 Hz, 1H), 1.71 – 1.48 (m, 4H), 1.42 – 1.22 (m, 2H). ^{13}C NMR (101 MHz, $CDCl_3$) δ 156.8, 156.2, 139.8, 139.1, 138.6 (2C), 138.0 (2C), 137.9, 137.4, 134.4, 129.1, 128.6 (3C), 128.4, 128.3 (3C), 128.1 (2C), 128.0 (2C), 127.9 (2C), 127.8 (2C), 127.7, 127.6 (3C), 127.4, 127.3, 127.1, 126.1, 117.3, 102.7, 101.6, 100.5, 97.1, 83.2, 82.2, 80.6, 79.1, 78.5, 78.2, 77.5, 76.5, 75.4, 75.3, 75.0, 74.0, 73.5 (2C), 73.4, 72.0, 70.1, 69.2, 68.4, 68.1, 67.9, 67.2, 63.7, 50.6, 50.3, 47.3, 46.3, 29.2, 28.1, 27.7, 23.6.

2-Azido-4,6-*O*-benzylidene-2-deoxy-β-D-mannopyranosyl-(1→4)-2,3,6-tri-*O*-benzyl-β-D-glucopyranosyl-(1→4)-2,3,6-tri-*O*-benzyl-α-D-glucopyranosyl-(1→1)-(5-*N*-benzyl-*N*-benzyloxycarbonylamino)pentanol (25)

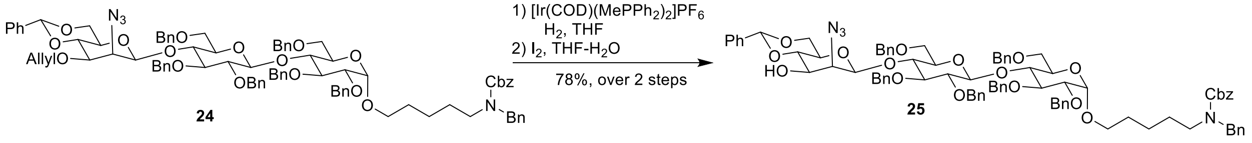

(1,5-Cyclooctadiene)bis(methyldiphenylphosphine)iridium(I) hexafluorophosphate (0.016 g, 0.019 mmol) was taken in THF (5 mL) and bubbled with nitrogen for 2 min at rt. Then, a hydrogen balloon was placed in place of nitrogen and the RM was purged thrice with hydrogen for about 2 min. The color of the reaction mixture changed from red to a clear colorless solution and the RM was further stirred at rt for additional 15 min under hydrogen. The activated catalyst was then added to a stirred solution of **24** (1.4 g, 0.929 mmol) in THF (10 mL) via cannula, and the stirring was continued for 4 h at rt. The reaction was then quenched with aq. $NaHCO_3$ solution (5 mL) and extracted with EtOAc (5 mL × 3). The combined organics were dried over anhydrous Na_2SO_4, filtered, and evaporated in vacuum to obtain the isomerized crude.

The isomerized material was taken in THF/water (15 mL:10 mL) and treated with iodine (0.283 g, 1.114 mmol) at rt. The brown-colored solution was then stirred for 3 h, quenched with 10% solution of $Na_2S_2O_3$ solution, and extracted with EtOAc (50 mL × 2). The combined organics were dried over Na_2SO_4, filtered, and evaporated in vacuum to obtain a brownish-yellow liquid. The crude was then purified by silica gel column chromatography using 30–40% EtOAc in hexanes to obtain **25** as colorless gummy liquid which on drying under vacuum became a fluffy powder (1.06 g, 78%, over two steps). $[\alpha]_D^{20}$ = -1.38° (c = 1.00, CH_2Cl_2); IR (thin film, cm^{-1}): νmax: 2866, 2108, 1698, 1091. HRMS (ESI): calculated for $C_{87}H_{94}N_4NaO_{17}$ $[M + Na]^+$, 1489.6512, found 1489.6528. ^{1}H NMR (600 MHz, $CDCl_3$) δ 7.53–7.10 (m, 45H), 5.44 (s, 1H), 5.18 (d, *J* = 12.2 Hz, 2H), 5.11 (d, *J* = 11.4 Hz, 1H), 5.00 (d, *J* = 10.6 Hz, 1H), 4.83–4.66 (m, 7H), 4.61 (dd, *J* = 17.2, 12.1 Hz, 2H), 4.53–4.42 (m, 3H), 4.35–4.27 (m, 2H), 4.22 (d, *J* = 11.8 Hz, 1H), 4.01 (dd, *J* = 10.5, 4.9 Hz, 1H), 3.95 (t, *J* = 9.4 Hz, 2H), 3.90–3.80 (m, 2H), 3.71 (d, *J* = 3.9 Hz, 1H), 3.66–3.42 (m, 9H), 3.41–3.10 (m, 6H), 2.99 (td, *J* = 9.7, 5.0 Hz, 1H), 2.35 (d, *J* = 5.6 Hz, 1H), 1.69–1.48 (m, 4H), 1.39–1.20 (m, 2H). ^{13}C NMR (151 MHz, $CDCl_3$) δ 156.8, 156.3, 139.9, 139.2, 138.7, 138.6, 138.0 (2C), 137.9, 137.1, 137.0, 136.9, 129.4, 128.7 (2C), 128.6 (2C), 128.5, 128.4 (2C), 128.3 (3C), 128.1(2C), 128.0 (3C), 127.9, 127.8 (4C), 127.6 (2C), 127.5, 127.3, 127.1, 126.4, 102.7, 102.2, 100.7, 97.1, 83.1, 82.2, 80.6, 79.2, 78.5 (2C), 77.2, 75.4, 75.3, 75.1, 74.1, 73.6, 73.5, 73.4, 70.1, 70.0, 69.1, 68.4, 68.1, 67.9, 67.3, 67.0, 64.9, 50.7, 50.4, 47.3, 46.3, 29.2, 28.1, 27.7, 23.6.

Thexyl-(methyl[2,3,4-tri-*O*-benzyl-αβ-D-glucopyranosyl]uronate-(1→3))-2-*O*-benzyl-4,6-*O*-benzylidene-β-D-galactopyranoside (26)

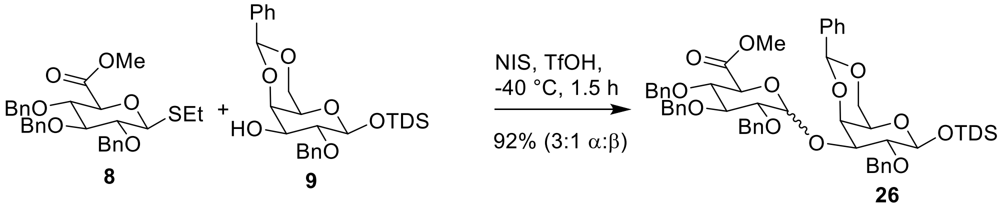

A stirred solution of both thioglycoside donor **8** (2.218 g, 4.24 mmol) [2] and galactose acceptor **9** (1.7 g, 3.4 mmol) in DCM/ether (20 mL:40 mL) was treated with 1 g of AW molecular sieves and stirred at rt for 15 min before being cooled to −40 °C. After the addition of NIS (0.917 g, 4.07 mmol) and TfOH (0.045 mL, 0.509 mmol), the RM was stirred at −40 °C to −20 °C for 30 min, then warmed to 0 °C for 30 min. The reaction was finally quenched with $Na_2S_2O_3$ solution, filtered through a cotton plug, and extracted with DCM (10 mL × 3). The combined organics were then washed with brine (10 mL), dried over anhydrous Na_2SO_4, filtered, and evaporated in vacuum to obtain a brown residue. The crude was purified by silica gel column chromatography using 15–25% EtOAc in hexanes to obtain compound **26** (3.0 g, 92%) [α/β = 3.3:1, *α*-anomer (2.3 g), *β*-anomer (0.7 g)]. *α*-anomer:$[\alpha]_D^{20}$ = +25.29° (c = 1.00, CH_2Cl_2); IR (thin film, cm^{-1}): νmax: 2954, 2868, 1750, 1080, 1061. HRMS (ESI): calculated for $C_{56}H_{68}NaO_{12}Si$ $[M + Na]^+$, 983.4378, found 983.4396. ^1H NMR (400 MHz, $CDCl_3$) δ 7.72–6.85 (m, 25H), 5.53 (s, 1H), 5.23 (d, *J* = 3.6 Hz, 1H), 4.95–4.71 (m, 6H), 4.56–4.43 (m, 4H), 4.35–4.23 (m, 2H), 4.09–4.02 (m, 2H), 3.91–3.79 (m, 2H), 3.73–3.65 (m, 1H), 3.62 (dd, *J* = 9.6, 3.5 Hz, 1H), 3.59 (s, 3H), 3.37 (dt, *J* = 2.7, 1.5 Hz, 1H), 1.78–1.68 (m, 1H), 1.06–0.73 (m, 12H), 0.24 (s, 3H), 0.20 (s, 3H). ^{13}C NMR (101 MHz, $CDCl_3$) δ 170.7, 138.7, 138.6, 138.4, 138.1, 138.0, 129.3, 128.4, 128.4, 128.3, 128.3, 128.2, 128.0, 127.8, 127.7 (2C), 127.4, 126.8, 101.7, 98.3, 92.7, 81.2, 79.9, 79.1, 78.6, 75.9, 75.4, 75.0, 74.8, 72.0, 71.5, 70.4, 69.7, 66.1, 52.5, 34.1, 25.0, 20.4, 20.2, 18.8, 18.6, -1.7, -2.3. *β*-anomer contaminated with donor impurity: ^1H NMR (400 MHz, $CDCl_3$) δ 7.70–7.05 (m, 25H), 5.62 (s, 1H), 5.07 (d, *J* = 7.4 Hz, 1H), 4.99 (t, *J* = 11.1 Hz, 2H), 4.90–4.83 (m, 2H), 4.82–4.75 (m, 2H), 4.73 (d, *J* = 7.3 Hz, 1H), 4.55 (d, *J* = 10.9 Hz, 1H), 4.45 (d, *J* = 10.0 Hz, 1H), 4.29 (dd, *J* = 8.1, 3.8 Hz, 2H), 4.08 (d, *J* = 10.6 Hz, 1H), 3.94 (dd, *J* = 9.8, 3.6 Hz, 1H), 3.89–3.73 (m, 3H), 3.70 (s, 3H), 3.59–3.46 (m, 2H), 3.42 (s, 1H), 1.76 (dt, *J* = 13.7, 6.9 Hz, 1H), 0.95 (d, *J* = 6.7 Hz, 12H), 0.28 (s, 3H), 0.24 (s, 3H). ^{13}C NMR (101 MHz, $CDCl_3$) δ 169.4, 138.5, 138.47, 138.2, 137.8, 128.9, 128.7, 128.6, 128.5 (2C), 128.4, 128.3(2C), 128.2, 128.15, 128.1, 128.0 (2C), 127.9, 127.8, 127.7, 127.6, 126.4, 103.5, 100.9, 98.3, 83.7, 81.2, 80.9, 79.4, 76.1, 75.9, 75.6, 75.2, 74.7, 74.4, 74.2, 69.2, 66.5, 52.5, 34.0, 25.0, 20.4, 20.1, 18.7, 18.6, -1.6, -2.5.

Thexyl-(methyl[2,3,4-tri-*O*-benzyl-α-D-glucopyranosyl]uronate-(1→3))-2,6-di-*O*-benzyl-β-D-galactopyranoside (27)

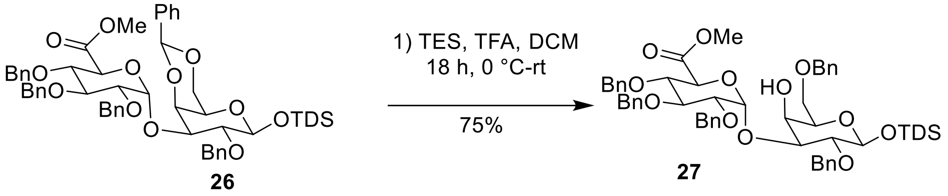

Substrate **26** (1.6 g, 1.67 mmol) was taken in DCM (15 mL) with activated 4 Å molecular sieves for 10 min before cooling to 0 °C. Triethylsilane (2.13 mL, 13.32 mmol) was added, followed by TFA (0.77 mL, 9.99 mmol), and then the RM was stirred at rt for 16 h before quenching with water (20 mL). The RM was filtered through a celite® bed and the aqueous layer extracted with DCM (100 mL × 3). The combined organics were washed thoroughly with sat. $NaHCO_3$ solution (50 mL × 2), water (20 mL × 3), brine (20 mL), dried over anhydrous Na_2SO_4, filtered, and evaporated in vacuum to obtain a colorless gummy solid. The crude product was purified by silica gel column chromatography using 20% EtOAc in hexanes to obtain **27** as colorless oil (1.2 g, 75%). $[\alpha]_D^{20}$ = + 40.48° (c = 1.00, CH_2Cl_2); IR (thin film, cm–1): νmax: 3506, 2953, 2866, 1747, 1070. HRMS (ESI): calculated for $C_{56}H_{70}NaO_{12}Si$ $[M + Na]^+$, 985.4534, found 985.4575. ^{1}H NMR (400 MHz, $CDCl_3$) δ 7.45 – 7.16 (m, 25H), 4.96 (d, *J* = 10.8 Hz, 1H), 4.90 (d, *J* = 1.3 Hz, 2H), 4.86 – 4.71 (m, 4H), 4.69 – 4.59 (m, 4H), 4.56 (d, *J* = 11.0 Hz, 1H), 4.51 (d, *J* = 10.1 Hz, 1H), 3.98 (t, *J* = 9.4 Hz, 1H), 3.89 (d, *J* = 3.1 Hz, 1H), 3.84 (dd, *J* = 9.7, 6.0 Hz, 1H), 3.79 – 3.53 (m, 6H), 3.51 (s, 3H), 3.39 (s, 1H), 1.77 – 1.66 (m, 1H), 1.02 – 0.77 (m, 12H), 0.24 (s, 3H), 0.20 (s, 3H). ^{13}C NMR (101 MHz, $CDCl_3$) δ 170.5, 138.5, 138.3, 138.2 (2C), 137.4, 128.7 (2C), 128.6, 128.5 (3C), 128.4 (2C), 128.3 (2C), 128.0, 127.9, 127.8 (2C), 127.4, 98.5, 94.7, 81.5, 80.3, 78.7, 78.6, 78.3, 75.9, 75.1, 74.5, 73.8, 72.9, 70.5, 69.5, 65.1, 52.3, 33.9, 24.9, 20.3, 20.0, 18.7, 18.5, -1.7, -2.8.

Thexyl-(methyl[2,3,4-tri-*O*-benzyl-α-D-glucopyranosyl]uronate-(1→3))-4-*O*-benzoyl-2,6-di-*O*-benzyl-β-D-galactopyranoside (6)

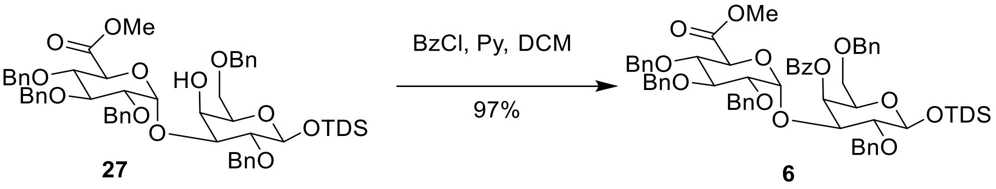

Substrate **27** (1.4 g, 1.45 mmol) was taken in DCM (10 mL) at 0 °C and treated with pyridine (10 mL) and BzCl (0.84 mL, 7.27 mmol). The RM was stirred at rt for 36 h and then concentrated in vacuum to obtain a solid residue that was then taken in DCM (50 mL) and water (50 mL). The solution was acidified with dil. HCl (100 mL), and extracted with DCM (50 mL × 3). The combined organics were washed with saturated $NaHCO_3$ (100 mL), brine (25 mL), dried over anhydrous Na_2SO_4, filtered, concentrated under vacuum, and dried to obtain the crude. Purification by silica gel column chromatography using 20% EtOAc in hexanes afforded **6** as colorless oil (1.5 g, 97%). $[\alpha]_D^{20}$ = +33.01° (c = 1.00, CH_2Cl_2); IR (thin film, cm^{-1}): νmax : 2954, 2867, 1749, 1721, 1095, 1070. HRMS (ESI): calculated for $C_{63}H_{74}NaO_{13}Si$ $[M + Na]^+$, 1089.4796, found 1089.4813. ^1H NMR (400 MHz, $CDCl_3$) δ 8.02 (dd, *J* = 8.3, 1.4 Hz, 2H), 7.68 – 6.97 (m, 28H), 5.83 (d, *J* = 3.2 Hz, 1H), 5.38 (d, *J* = 3.4 Hz, 1H), 4.95 (d, *J* = 10.7 Hz, 1H), 4.87 (d, *J* = 10.7 Hz, 1H), 4.80 (d, *J* = 7.2 Hz, 1H), 4.72 (dd, *J* = 11.0, 3.5 Hz, 2H), 4.67 – 4.40 (m, 7H), 3.98 (dd, *J* = 9.9, 3.3 Hz, 1H), 3.93 (t, *J* = 9.3 Hz, 1H), 3.84 – 3.75 (m, 2H), 3.67 (dd, *J* = 10.1, 9.0 Hz, 1H), 3.60 (s, 3H), 3.60 (s, 3H), 1.79 – 1.69 (m, 1H), 1.00 – 0.85 (m, 12H), 0.25 (s, 3H), 0.21 (s, 3H). ^{13}C NMR (101 MHz, $CDCl_3$) δ 170.5, 166.1, 138.7, 138.5, 138.3, 138.2, 133.2, 130.2, 129.9, 128.5 (2C), 128.4, 128.3 (4C), 128.0, 127.8 (3C), 127.6, 127.4 (2C), 98.7, 93.5, 81.0, 80.0, 78.7, 75.6, 75.4, 75.0, 74.5, 73.8, 72.7, 72.5, 70.3, 68.9, 66.7, 52.4, 34.2, 25.0, 20.5, 20.2, 18.8, 18.6, -1.9, -2.4.

Methyl[2,3,4-tri-*O*-benzyl-α-D-glucopyranosyl]uronate-(1→3)-4-*O*-benzoyl-2,6-di-*O*-benzyl-αβ-D-galactopyranoside (28)

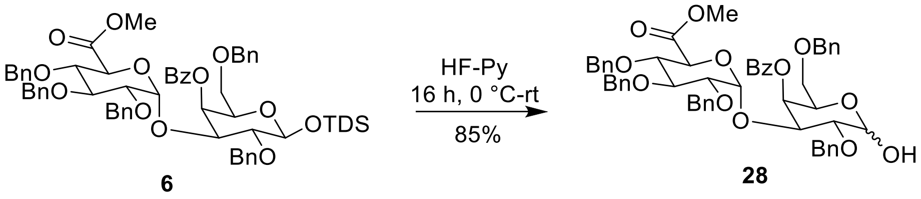

Substrate **6** (1.5 g, 1.405 mmol) was taken in pyridine (15 mL) at 0 °C and treated with 70% HF in pyridine (3.62 mL, 28.1 mmol). The RM was warmed to rt and stirred for 15 h, then quenched with $NaHCO_3$ solution, and extracted with EtOAc (10 mL × 3). The combined organics were dried over Na_2SO_4, filtered, and evaporated in vacuum. The crude product was then purified by silica gel column chromatography using 40–60% EtOAc in hexanes to obtain **28** as clear colorless oil (1.1 g, 85%). $[\alpha]_D^{20}$ = +51.79° (c = 1.00, CH_2Cl_2); IR (thin film, cm^{-1}): νmax : 3426, 2872, 1748, 1721,1097, 1071. HRMS (ESI): calculated for $C_{55}H_{56}NaO_{13}$ $[M + Na]^+$, 947.3619, found 947.3645. ^1H NMR (400 MHz, $CDCl_3$, mixture of α and β anomers) δ 8.04 – 7.92 (m, 2H), 7.64 – 7.08 (m, 28H), 5.84 (d, *J* = 3.2 Hz, 0.7H), 5.79 (d, *J* = 3.2 Hz, 0.3H), 5.43 (d, *J* = 3.5 Hz, 0.7H), 5.38 (d, *J* = 3.2 Hz, 1H), 4.95 – 4.91 (m, 0.7H), 4.88 (d, *J* = 11.6 Hz, 0.7H), 4.81 (d, *J* = 7.7 Hz, 0.3H), 4.79 – 4.54 (m, 6.6H), 4.53 – 4.34 (m, 5H), 4.05 (dd, *J* = 10.0, 3.5 Hz, 0.7H), 4.02 – 3.92 (m, 1.3H), 3.89 – 3.80 (m, 1H), 3.77 – 3.69 (m, 1H), 3.66 (s, 2H), 3.63 – 3.47 (m, 4H). ^{13}C NMR (101 MHz, $CDCl_3$, mixture of α and β anomers) δ 170.5 (2C), 166.1, 166.0, 149.1, 138.6 (2C), 138.4 (2C), 138.1 (2C), 137.9, 137.7, 137.5, 136.8, 133.5, 133.3, 133.2, 130.2, 130.1, 129.8, 129.5, 128.9, 128.6, 128.5 (3C), 128.4 (2C), 128.3 (3C), 128.2, 128.1 (2C), 128.0 (4C), 127.9 (2C), 127.8 (3C), 127.7, 127.6 (2C), 127.5, 127.4, 124.1, 98.0, 93.5 (2C), 91.7, 81.1, 81.0, 79.9, 79.7, 78.7 (2C), 78.5, 75.6 (2C), 75.1, 75.0, 74.9, 74.3, 73.8, 73.6, 72.8, 72.7 (2C), 71.0, 70.4, 70.3, 68.8 (2C), 68.4, 67.7, 66.5, 52.6, 52.4.

Methyl [2,3,4-tri-*O*-benzyl-α-D-glucopyranosyl]uronate-(1→3)-4-*O*-benzoyl-2,6-di-*O*-benzyl-αβ-D-galactopyranosyl trifluoro-(*N*-phenyl)acetimidate (29)

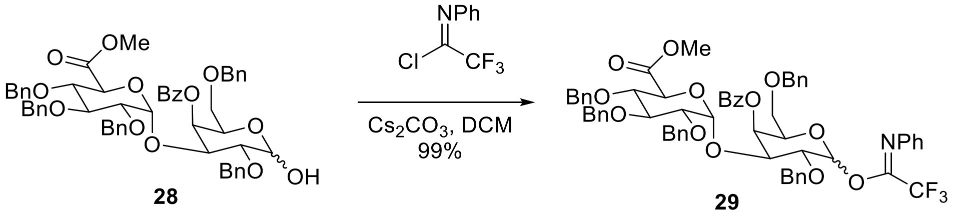

28 **29**

Substrate **28** (0.70 g, 0.76 mmol) was taken in DCM (10 mL), cooled to 0 °C, and treated with (*E*)-2,2,2-trifluoro-*N*-phenylacetimidoyl chloride (0.47 g, 2.27 mmol) and Cs_2CO_3 (0.49 g, 1.51 mmol) and slowly warmed to rt over 1 h. The RM was filtered and the filtrate was evaporated in vacuum to obtain a crude, which was then purified by silica gel column chromatography using 2–10% EtOAc in hexanes with 1% Et_3N to obtain **29** as pale yellowish fluffy powder (0.82 g, 99%). HRMS (ESI): calculated for $C_{63}H_{60}F_3NNaO_{13}$ $[M + Na]^+$, 1118.3914, found 1118.3957. ^{1}H NMR (600 MHz, $CDCl_3$, mixture of α and β anomers) δ 8.04 – 7.87 (m, 2H), 7.66 – 7.01 (m, 33H), 6.79 (d, *J* = 7.8 Hz, 1.3H), 6.73 – 6.56 (m, 0.7H), 5.96 (d, *J* = 3.1 Hz, 0.3H), 5.90 (s, 0.7H), 5.44 (dd, *J* = 8.5, 3.3 Hz, 1H), 4.93 (d, *J* = 10.4 Hz, 0.7H), 4.89 (d, *J* = 12.1 Hz, 0.3H), 4.81 (d, *J* = 10.4 Hz, 0.7H), 4.76 (ddd, *J* = 14.7, 10.9, 4.1 Hz, 2.3H), 4.71 – 4.53 (m, 4.3H), 4.53 – 4.40 (m, 3.7H), 4.37 (br s, 0.3H), 4.20 (d, *J* = 10.3 Hz, 0.3H), 4.13 – 4.01 (m, 1.3H), 3.98 (t, *J* = 9.3 Hz, 0.7H), 3.92 (t, *J* = 9.3 Hz, 0.3H), 3.79 – 3.70 (m, 1H), 3.68 (s, 1H), 3.65 – 3.48 (m, 5H). ^{13}C NMR (151 MHz, $CDCl_3$, mixture of α and β anomers) δ 170.5, 170.3, 165.9, 165.8, 143.7, 143.5, 138.7, 138.6, 138.5, 138.4, 138.2, 138.1, 137.7, 137.6 (2C), 137.3, 133.4, 133.3, 130.2, 130.1, 129.6, 129.5 129.3, 129.0 (2C), 128.9, 128.8 (2C), 128.7, 128.6 (2C), 128.5 (3C), 128.4 (2C), 128.3 (2C), 128.2 (2C), 128.0 (3C), 127.9 (3C), 127.8 (3C), 127.7, 127.6 (3C), 127.5, 124.4, 120.8, 119.4, 93.5, 93.1, 81.1, 81.0, 79.9, 79.6, 79.0, 78.7, 76.9, 76.2, 75.7, 75.6, 75.2, 75.0, 74.4, 73.9, 73.7, 73.6 (2C), 72.8, 72.7, 71.2, 70.5, 70.3, 70.2, 68.3, 68.0, 66.9, 66.1, 52.5, 52.4.

Methyl[2,3,4-tri-*O*-benzyl-α-D-glucopyranosyl]uronate-(1→3)-4-*O*-benzoyl-2,6-di-*O*-benzyl-α-D-galactopyranosyl-(1→3)-2-azido-4,6-*O*-benzylidene-2-deoxy-β-D-mannopyranosyl-(1→4)-2,3,6-tri-*O*-benzyl-β-D-glucopyranosyl-(1→4)-2,3,6-tri-*O*-benzyl-α-D-glucopyranosyl-(1→1)-(5-*N*-benzyl-*N*-benzyloxycarbonylamino)pentanol (30)

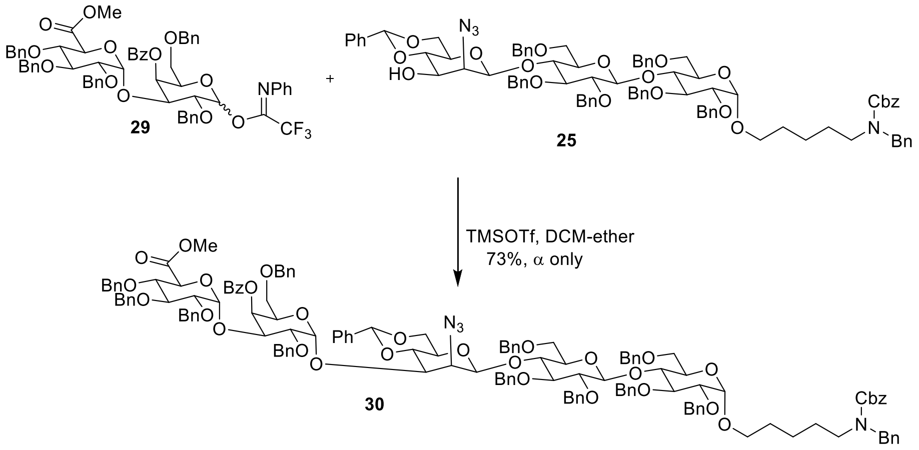

Acceptor **25** (0.2 g, 0.136 mmol) was taken in DCM/ether (2 mL:2 mL) with activated molecular sieves and stirred at rt for 10 min before being cooled to −10 °C. One portion of donor substrate **29** (0.112 g, 0.102 mmol) in DCM (200 µL) was added, followed by TMSOTf (1.231 µL, 6.81 µmol) and the RM was stirred for 1 h at −10 °C. The remaining portion of donor **29** (0.112 g, 0.102 mmol) in DCM (200 µL) was added dropwise over 10 min at −10 °C to −7 °C and the RM was stirred for additional 1 h at −7 °C. The reaction was then quenched with 0.5 mL of Et_3N, stirred for 15 min, filtered through a Celite® bed, washed thoroughly with DCM (5 mL × 10) and the solvents removed under vacuum. The crude product was then purified by silica gel column chromatography using 50% EtOAc in hexanes to obtain **30** as colorless gummy liquid (235 mg, 73%). $[\alpha]_D^{20}$ = +42.40° (c = 1.00, CH_2Cl_2); IR (thin film, cm^{-1}): νmax: 2867, 2107, 1751, 1699, 1092. HRMS (ESI): calculated for $C_{142}H_{148}N_4NaO_{29}$ $[M + Na]^+$, 2396.0127, found 2396.0015. ^{1}H NMR (600 MHz, $CDCl_3$) δ 7.85 (dd, *J* = 8.2, 1.1 Hz, 2H), 7.56–7.01 (m, 73H), 5.76 (d, *J* = 3.0 Hz, 1H), 5.42 (d, *J* = 3.7 Hz, 1H), 5.30 (s, 1H), 5.27 (d, *J* = 3.4 Hz, 1H), 5.17 (d, *J* = 11.6 Hz, 2H), 5.10 (d, *J* = 11.4 Hz, 1H), 5.01 (d, *J* = 10.5 Hz, 1H), 4.79–4.66 (m, 7H), 4.65–4.51 (m, 8H), 4.52–4.29 (m, 11H), 4.19 (dd, *J* = 7.3, 4.7 Hz, 1H), 4.13 (d, *J* = 12.1 Hz, 1H), 4.05–3.73 (m, 10H), 3.65–3.59 (m, 2H), 3.57 (s, 3H), 3.57–3.41 (m, 9H), 3.38 (t, *J* = 9.0 Hz, 1H), 3.36–3.33 (m, 1H), 3.31 (dd, *J* = 9.2, 7.8 Hz, 1H), 3.27–3.13 (m, 2H), 3.10 (dt, *J* = 9.7, 2.7 Hz, 1H), 2.91 (td, *J* = 9.7, 4.9 Hz, 1H), 1.71–1.47 (m, 4H), 1.41–1.21 (m, 2H). ^{13}C NMR (151 MHz, $CDCl_3$) δ 170.6, 166.1,

139.9, 139.2, 138.73, 138.7, 138.5, 138.3, 138.2, 138.1, 137.9, 137.8, 137.4, 133.2, 130.1, 129.8, 129.3, 128.7, 128.6 (3C), 128.4 (2C), 128.3 (2C), 128.2 (2C), 128.1 (2C), 128.0, 127.8, 127.74, 127.7, 127.6 (2C), 127.5 (2C), 127.4, 127.3, 127.2, 127.1, 126.5, 102.9, 102.1, 99.8, 98.0, 97.1, 93.9, 83.2, 82.2, 81.1, 80.6, 80.0, 79.2, 78.7, 78.4 (2C), 77.3, 75.6, 75.5, 75.3, 75.2, 75.1, 74.7, 74.5, 73.8, 73.6, 73.4 (2C), 73.1, 72.6, 70.9, 70.7, 70.4, 70.2, 69.7, 69.4, 68.9, 68.6, 68.1, 67.9, 67.3, 67.1, 64.6, 52.3, 50.7, 50.4, 47.3, 46.3, 29.2, 28.2, 27.7, 23.6.

Methyl[2,3,4-tri-*O*-benzyl-α-D-glucopyranosyl]uronate-(1→3)-4-*O*-benzoyl-2,6-di-*O*-benzyl-α-D-galactopyranosyl-(1→3)-2-acetamido-4,6-*O*-benzylidene-2-deoxy-β-D-mannopyranosyl-(1→4)-2,3,6-tri-*O*-benzyl-β-D-glucopyranosyl-(1→4)-2,3,6-tri-*O*-benzyl-α-D-glucopyranosyl-(1→1)-(5-*N*-benzyl-*N*-benzyloxycarbonylamino)pentanol (31)

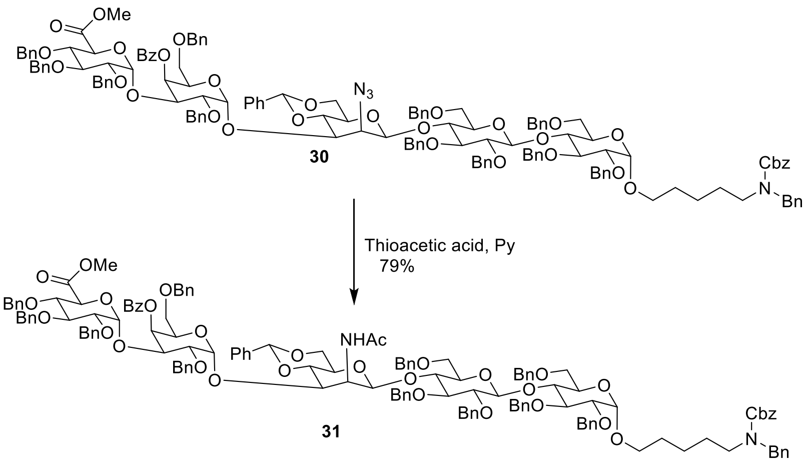

Azide substrate **30** (0.42 g, 0.177 mmol) was taken in dry pyridine (4 mL). After the addition of thioacetic acid (2.5 mL, 34.8 mmol), the RM was stirred at rt for 48 h. The RM was then evaporated in vacuum and purified by silica gel chromatography using 30–35% EtOAc in hexanes to recover azide **30** (200 mg) and 40–60% EtOAc in hexanes to obtain compound **31** (228 mg) as white fluffy solid after drying. The recovered azide starting material (200 mg) was resubjected to the same reaction conditions with thioacetic acid (1.0 mL), pyridine (4 mL) and stirring for 24 h. Additional 1 mL of thioacetic acid and pyridine were added to the RM and stirring was continued for another 48 h. The RM was concentrated in vacuum and purified by silica gel chromatography using 30–35% EtOAc in hexanes to recover azide **30** (100 mg) and 40–60% EtOAc in hexanes to obtain

compound **31** (106 mg) as white fluffy solid after drying. The two batches were combined and dried in vacuum to afford compound **31** (0.33 g, 79%). $[\alpha]_D^{20}$ = +45.00° (c = 1.00, CH_2Cl_2); IR (thin film, cm^{-1}): νmax : 2926, 2868, 1721, 1698, 1092, 1072, 1028. HRMS (ESI): calculated for $C_{144}H_{152}N_2NaO_{30}$ $[M + Na]^+$, 2412.0328, found 2412.0327. 1H NMR (600 MHz, $CDCl_3$) δ 7.81 (dd, *J* = 8.2, 1.2 Hz, 2H), 7.62 – 6.88 (m, 73H), 5.74 (d, *J* = 3.4 Hz, 1H), 5.55 (d, *J* = 9.6 Hz, 1H), 5.43 (d, *J* = 3.6 Hz, 1H), 5.29 (s, 1H), 5.17 (d, *J* = 12.7 Hz, 2H), 5.14 (d, *J* = 3.3 Hz, 1H), 5.12 (d, *J* = 11.4 Hz, 1H), 4.84 – 4.77 (m, 3H), 4.77 – 4.66 (m, 6H), 4.65 – 4.42 (m, 15H), 4.41 – 4.27 (m, 5H), 4.20 (dd, *J* = 10.2, 3.3 Hz, 1H), 4.13 – 4.03 (m, 2H), 4.01 – 3.86 (m, 5H), 3.83 (td, *J* = 9.3, 3.9 Hz, 2H), 3.74 – 3.50 (m, 8H), 3.49 (s, 3H), 3.47 – 3.34 (m, 4H), 3.33 – 3.21 (m, 3H), 3.19 – 3.15 (m, 1H), 3.05 (dt, *J* = 9.7, 2.6 Hz, 1H), 2.89 (td, *J* = 9.8, 4.9 Hz, 1H), 1.86 (s, 3H), 1.65 – 1.48 (m, 4H), 1.37 – 1.28 (m, 2H). ^{13}C NMR (151 MHz, $CDCl_3$) δ 170.6, 170.5, 166.2, 156.8, 156.3, 139.8, 139.3, 138.7 (2C), 138.4 (2C), 138.3, 138.2, 138.1, 138.0, 137.3, 132.9, 130.2, 130.0, 129.5, 128.7, 128.6, 128.5 (2C), 128.4 (3C), 128.3 (3C), 128.1 (3C), 128.0 (2C), 127.9, 127.8 (2C), 127.7 (3C), 127.6 (2C), 127.5 (3C), 127.4, 127.3, 127.2, 127.1, 126.9, 126.5, 102.9, 102.4, 99.1, 97.1, 97.07, 94.5, 83.3, 82.2, 81.0, 80.6, 79.9, 79.8, 79.1, 78.3, 77.0, 75.8, 75.4, 75.3, 75.2, 75.0, 74.7, 74.5, 73.6, 73.5, 73.4, 73.2, 73.0, 72.9, 72.5, 71.8, 70.7, 70.3, 70.21, 70.2, 69.2, 68.84, 68.8, 68.7, 68.1, 68.0, 67.3, 66.7, 52.7, 52.2, 50.7, 50.4, 47.3, 46.3, 29.8, 29.2, 28.1, 27.7, 23.6, 23.2.

2,3,4-Tri-*O*-benzyl-α-D-glucopyranosyluronate-(1→3)-2,6-di-*O*-benzyl-α-D-galactopyranosyl-(1→3)-2-acetamido-4,6-*O*-benzylidene-2-deoxy-β-D-mannopyranosyl-(1→4)-2,3,6-tri-*O*-benzyl-β-D-glucopyranosyl-(1→4)-2,3,6-tri-*O*-benzyl-α-D-glucopyranosyl-(1→1)-(5-*N*-benzyl-*N*-benzyloxycarbonylamino)pentanol (32)

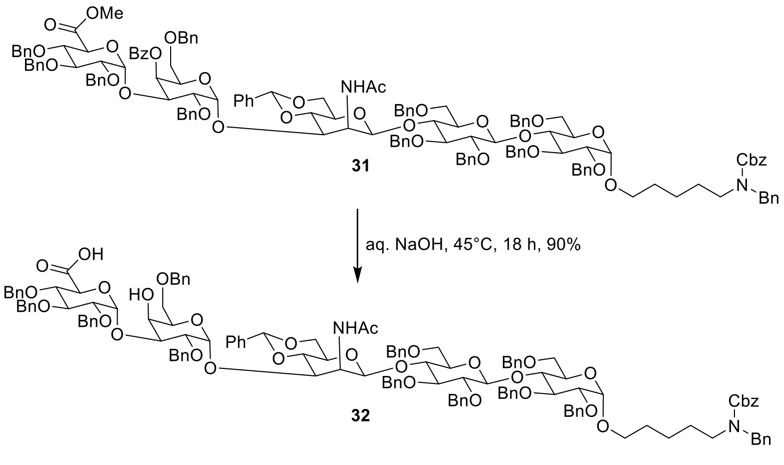

To a solution of substrate **31** (0.14 g, 0.059 mmol) in THF (5 mL), 15% aq. NaOH solution (2 mL) was added. The RM was stirred at 45 °C for 18 h, cooled to rt and evaporated in vacuum. The residue was then diluted with water, acidified with acetic acid and extracted with EtOAc (5 mL × 4). The combined organics were washed with water (5 mL × 2), brine solution (5 mL), dried over anhydrous Na_2SO_4, filtered, and evaporated in vacuum. Purification by silica gel column chromatography using 100% EtOAc–20% acetone in EtOAc provided the white gummy product **32** (0.12 g, 90%). $[\alpha]_D^{20}$ = +42.58° (c = 1.00, acetone); IR (thin film, cm^{-1}): νmax : 3445, 2926, 2868, 1698, 1655, 1454, 1092, 1042. HRMS (ESI): calculated for $C_{136}H_{146}N_2NaO_{29}$ $[M + Na]^+$, 2293.9909, found 2293.9895. ^{1}H NMR (600 MHz, Acetone-d_6) δ 7.68 – 6.87 (m, 70H), 5.57 (brs, 1H), 5.55 (s, 1H), 5.43 (s, 1H), 5.22 – 5.09 (m, 3H), 5.01 (d, *J* = 11.2 Hz, 1H), 4.96 – 4.83 (m, 4H), 4.83 – 4.62 (m, 13H), 4.61 – 4.41 (m, 11H), 4.39 – 4.17 (m, 3H), 4.15 – 4.09 (m, 1H), 4.06 – 4.00 (m, 2H), 3.98 – 3.78 (m, 8H), 3.76 – 3.60 (m, 7H), 3.49 – 3.42 (m, 2H), 3.40 – 3.11 (m, 7H), 2.05 (s, 3H), 1.58 – 1.51 (m, 4H), 1.41 – 1.32 (m, 2H). 13C NMR (151 MHz, Acetone-d_6) δ 171.0, 141.0, 140.7, 140.1, 140.0, 139.9 (2C), 139.8, 139.5, 139.4, 139.1, 138.9, 130.6, 130.5, 129.8, 129.3, 129.2 (3C), 129.1 (3C), 129.0 (3C), 128.9 (2C), 128.8 (3C), 128.7 (4C), 128.6 (2C), 128.5 (3C), 128.4 (2C), 128.3 (2C), 128.2 (3C), 128.1(3C), 127.9 (2C), 127.7, 127.4, 103.3, 102.7, 101.1, 97.5, 96.8, 83.8, 83.1, 82.2, 81.1, 80.5, 80.3, 79.9, 79.0, 77.9, 76.0, 75.6, 75.5 (2C), 75.2, 73.8, 73.7 (2C), 73.6, 73.2, 71.4, 71.2, 69.9, 69.2, 69.0, 68.4, 68.0, 67.5, 67.1, 54.2, 51.0, 50.8, 47.8, 46.9, 29.9, 28.7 (2C), 27.8, 24.2, 23.3.

α-D-Glucopyranosyluronate-(1→3)-α-D-galactopyranosyl-(1→3)-2-acetamido-2-deoxy-β-D-mannopyranosyl-(1→4)-β-D-glucopyranosyl-(1→4)-α-D-glucopyranosyl-(1→1)-(5-amino)pentanol (4)

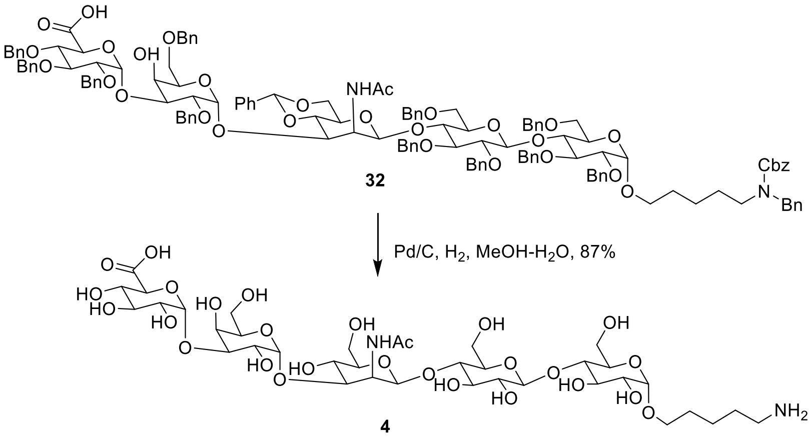

Substrate **32** (35 mg, 0.015 mmol) was taken in MeOH (4 mL) and treated with a suspension of Pd/C (25 mg) in methanol (1 mL) and H_2 using a balloon for 16 h at rt. The RM was then filtered through a PTFE filter, and washed thoroughly with methanol (5 mL), water/methanol (5 mL:5 mL), and water (5 mL). The filtrate was then evaporated in vacuum and dried to obtain **4** as colorless solid (13 mg, 87%). HRMS (ESI): calculated for $C_{37}H_{65}N_2O_{27}$ $[M + H]^+$, 969.3775, found 969.3793. ^{1}H NMR (600 MHz, D_2O) δ 5.34 (d, *J* = 3.9 Hz, 1H, αGal-H1), 5.17 (d, *J* = 3.8 Hz, 1H, αGlcA-H1), 4.92 (d, *J* = 3.9 Hz, 1H, αGlc-H1), 4.91 (d, *J* = 1.6 Hz, 1H, βManNAc-H1), 4.65 (dd, *J* = 4.5, 1.7 Hz, 1H, βManNAc-H2), 4.52 (d, *J* = 8.0 Hz, 1H, βGlc-H1), 4.41 (d, *J* = 10.1 Hz, 1H), 4.32 – 4.15 (m, 2H), 4.09 – 3.53 (m, 25H), 3.49 (dq, *J* = 7.7, 2.7 Hz, 1H), 3.36 (t, *J* = 8.5 Hz, 1H, βGlc-H2), 3.02 (t, *J* = 7.6 Hz, 2H), 2.06 (s, 3H), 1.78 – 1.60 (m, 4H), 1.56 – 1.40 (m, 2H). ^{13}C NMR (151 MHz, D_2O) δ 177.5, 177.1, 104.9 (βGlc-C1), 102.7 (αGal-C1), 101.9 (βManNAc-C1), 100.4(αGlc-C1), 97.8(αGlcA-C1), 81.3, 81.2, 79.4, 78.9, 77.2, 76.9, 76.6, 75.6, 75.1, 74.3 (2C), 74.1, 73.9, 73.7, 73.6, 73.1, 70.5, 69.3, 69.2, 68.4, 64.1, 62.9, 62.7, 62.5, 55.4, 42.0, 30.7, 29.1, 25.1, 24.5.

Benzyl[2,3,4-tri-*O*-benzyl-α-D-glucopyranosyl]uronate-(1→3)-2,6-di-*O*-benzyl-α-D-galactopyranosyl-(1→3)-2-acetamido-4,6-*O*-benzylidene-2-deoxy-β-D-mannopyranosyl-(1→4)-2,3,6-tri-*O*-benzyl-β-D-glucopyranosyl-(1→4)-2,3,6-tri-*O*-benzyl-α-D-glucopyranosyl-(1→1)-(5-*N*-benzyl-*N*-benzyloxycarbonylamino)pentanol (33)

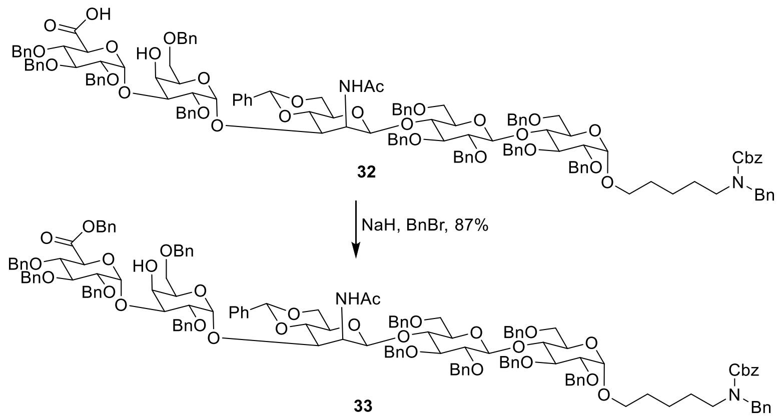

Substrate **32** (0.1 g, 0.044 mmol) was taken in DMF (3 mL) at 0 °C, treated with NaH (8.8 mg, 0.220 mmol) and stirred for 15 min. Then, BnBr (0.016 mL, 0.132 mmol) was added, the RM slowly warmed to rt, and stirred for additional 16 h. The reaction was then quenched with water (10 mL) and extracted with EtOAc (5 mL × 4). The combined organics were washed with water (5 mL × 2), brine (5 mL), dried over anhydrous Na_2SO_4, and evaporated in vacuum. The crude product was purified by silica gel column chromatography using 30–40% EtOAc in hexanes to obtain product **33** as gummy solid (90 mg, 87%). $[\alpha]_D^{20}$ = +33.76° (c = 0.50, CH_2Cl_2); IR (thin film, cm^{-1}): νmax: 2870, 1745, 1686, 1454, 1072, 1041. HRMS (ESI): calculated for $C_{143}H_{152}N_2NaO_{29}$ $[M + Na]^+$, 2384.0378, found 2384.0404. ^1H NMR (400 MHz, $CDCl_3$) δ 7.50–6.84 (m, 75H), 5.46 (d, *J* = 9.6 Hz, 1H), 5.36 (s, 1H), 5.29 (d, *J* = 3.5 Hz, 1H), 5.18 (d, *J* = 7.8 Hz, 2H), 5.14 (d, *J* = 11.3 Hz, 1H), 5.10 (d, *J* = 12.5 Hz, 1H), 5.01 (d, *J* = 12.3 Hz, 1H), 4.90–4.55 (m, 21H), 4.49 (d, *J* = 9.6 Hz, 2H), 4.46–4.33 (m, 4H), 4.32–4.21 (m, 2H), 4.18–4.07 (m, 2H), 4.07–3.79 (m, 9H), 3.83–3.40 (m, 14H), 3.37–3.12 (m, 4H), 3.04 (d, *J* = 9.6 Hz, 1H), 2.89 (td, *J* = 9.8, 5.0 Hz, 1H), 1.81 (s, 3H), 1.70–1.48 (m, 4H), 1.38–1.24 (m, 2H). ^{13}C NMR (101 MHz, $CDCl_3$) δ 170.6, 170.0, 156.8, 156.2, 139.8, 139.2, 138.63, 138.6, 138.3, 138.2, 138.1, 138.0, 137.9, 137.2 (2C), 135.2, 129.6, 128.7 (2C), 128.6 (4C), 128.5 (3C), 128.4 (2C), 128.3 (2C), 128.2, 128.1 (2C), 128.0 (2C), 127.9 (2C), 127.8 (3C), 127.7 (2C), 127.6 (2C), 127.5, 127.4, 127.3, 127.1, 126.9, 126.5, 102.9, 102.5, 99.1, 97.3, 97.1, 94.6, 83.2, 82.2, 81.6, 80.6, 80.2, 79.6, 79.1, 78.4, 77.3, 75.8, 75.7, 75.4, 75.2, 75.16, 74.9, 74.8, 74.5, 74.4, 73.5, 73.45, 73.3, 73.2, 73.1, 72.4, 71.0, 70.6, 70.5, 70.1, 68.8, 68.6, 68.1, 67.9, 67.3, 67.1, 66.8, 66.6, 52.9, 50.6, 50.3, 47.3, 46.3, 29.8, 29.2, 28.1, 27.7, 23.6, 23.4.

Benzyl[2,3,4-tri-*O*-benzyl-α-D-glucopyranosyl]uronate-(1→3)-2,6-di-*O*-benzyl-α-D-galactopyranosyl-(1→3)-2-acetamido-2-deoxy-β-D-mannopyranosyl-(1→4)-2,3,6-tri-*O*-benzyl-β-D-glucopyranosyl-(1→4)-2,3,6-tri-*O*-benzyl-α-D-glucopyranosyl-(1→1)-(5-*N*-benzyl-*N*-benzyloxycarbonylamino)pentanol (34)

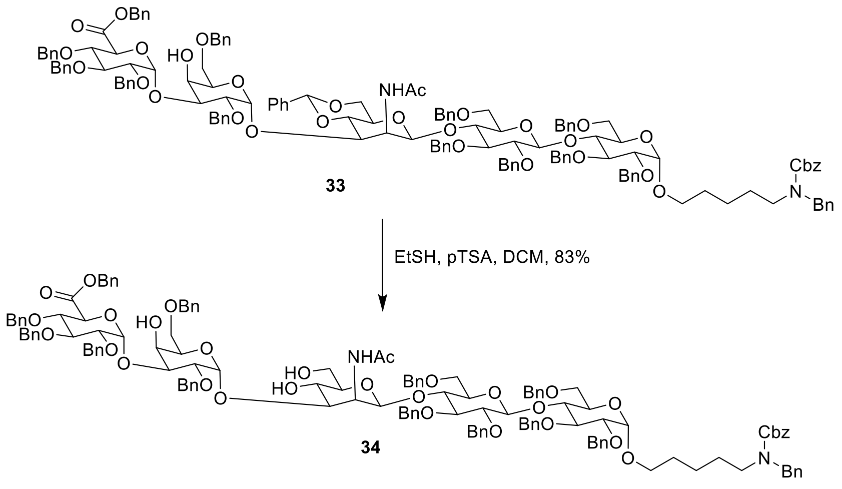

Benzylidene substrate **33** (35 mg, 0.15 mmol) was taken in chloroform (3 mL), pTSA (1 mg) and EtSH (1.096 µL, 0.015 mmol) were added and the RM stirred at rt for 6 h. The reaction was then quenched with triethylamine (1 mL) and concentrated in vacuum. Purification by silica gel column chromatography using 50–60% EtOAc in hexanes provided **34** as colourless oil (28 mg, 83%). $[\alpha]_D^{20}$ = +35.87° (c = 0.50, CH_2Cl_2); IR (thin film, cm^{-1}): νmax : 3441, 2926, 1747, 1698, 1454, 1072, 1043. HRMS (ESI): calculated for $C_{136}H_{148}N_2NaO_{29}$ $[M + Na]^+$, 2296.0065, found 2296.0094. ^1H NMR (400 MHz, $CDCl_3$) δ 7.96 – 6.67 (m, 70H), 5.35 (d, *J* = 9.8 Hz, 1H), 5.24 – 5.06 (m, 5H), 4.91 (d, *J* = 11.0 Hz, 1H), 4.88 – 4.40 (m, 25H), 4.33 (d, *J* = 12.1 Hz, 1H), 4.27 (d, *J* = 7.7 Hz, 1H), 4.18 (d, *J* = 12.2 Hz, 1H), 4.14 (d, *J* = 5.9 Hz, 1H), 4.08 – 3.70 (m, 11H),), 3.68 – 3.48 (m, 9H), 3.45 – 3.15 (m, 8H), 3.06 (ddd, *J* = 16.0, 9.6, 3.3 Hz, 2H), 2.96 (ddd, *J* = 9.3, 5.3, 3.7 Hz, 1H), 1.88 (s, 3H), 1.68 – 1.47 (m, 4H), 1.43 – 1.06 (m, 2H). ^{13}C NMR (101 MHz, $CDCl_3$) δ 170.0, 169.8, 156.8, 156.3, 139.9, 139.2, 138.6, 138.6, 138.4, 138.3, 138.1, 138.0, 137.9, 137.3, 137.0, 135.1, 129.0, 128.98, 128.8, 128.7, 128.68, 128.65, 128.6, 128.57, 128.52, 128.5, 128.43, 128.4, 128.38, 128.35, 128.3, 128.14, 128.1, 128.0, 127.95, 127.9, 127.87, 127.8, 127.74, 127.7, 127.6, 127.4, 127.3, 127.1, 126.7, 102.9, 101.6, 98.8, 97.1, 94.4, 83.3, 83.1, 82.2, 81.5, 80.7, 80.0, 79.0, 78.3, 77.36, 76.2, 75.9, 75.4, 75.3, 75.2, 75.13, 75.1, 75.0, 74.9, 74.4, 74.3, 73.6, 73.53, 73.5, 73.3, 71.2, 70.1, 69.5, 68.6, 68.5, 68.1, 67.8, 67.4, 67.3, 66.1, 62.6, 52.6, 50.6, 50.3, 47.3, 46.3, 29.8, 29.2, 28.1, 27.7, 23.7, 23.6.

Benzyl[2,3,4-tri-*O*-benzyl-α-D-glucopyranosyl]uronate-(1→3)-2,6-di-*O*-benzyl-α-D-galactopyranosyl-(1→3)-2-acetamido-6-*O*-acetyl-2-deoxy-β-D-mannopyranosyl-(1→4)-2,3,6-tri-*O*-benzyl-β-D-glucopyranosyl-(1→4)-2,3,6-tri-*O*-benzyl-α-D-glucopyranosyl-(1→1)-(5-*N*-benzyl-*N*-benzyloxycarbonylamino)pentanol (35)

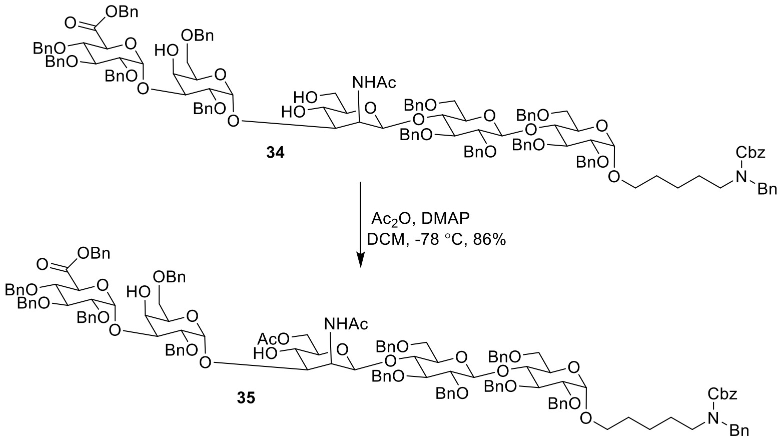

Substrate **34** (25 mg, 10.99 µmol) was taken in DCM (1 mL) and at −78 °C DMAP (6.71 mg, 0.055 mmol) in DCM (0.25 mL) was added, followed by Ac_2O (1.35 µL, 0.014 mmol) in DCM (0.25 mL), and the RM was stirred at −78 °C for 1 h. After warming to rt the reaction was then quenched with water (5 mL) and extracted with $CHCl_3$ (3 mL × 3). The combined organics were dried over Na_2SO_4, filtered, and concentrated in vacuum. Purification by column chromatography on silica gel using 30–50% EtOAc in hexanes afforded product **35** as a colorless oil (22 mg, 86%). $[\alpha]_D^{20}$ = +43.33° (c = 0.50, CH_2Cl_2); IR (thin film, cm^{-1}): νmax: 3441, 2923, 1743, 1698, 1454, 1073, 1042. HRMS (ESI): calculated for $C_{138}H_{150}N_2NaO_{30}$ $[M + Na]^+$, 2338.0171, found 2338.0180. ^1H NMR (600 MHz, $CDCl_3$) δ 7.64–6.95 (m, 70H), 5.37 (d, *J* = 9.8 Hz, 1H), 5.16 (d, *J* = 10.6 Hz, 2H), 5.14–5.05 (m, 3H), 4.95–4.67 (m, 13H), 4.66–4.39 (m, 14H), 4.34 (d, *J* = 12.1 Hz, 1H), 4.31–4.21 (m, 3H), 4.16 (dd, *J* = 12.2, 4.8 Hz, 1H), 4.11 (dd, *J* = 12.2, 2.3 Hz, 1H), 4.05–3.90 (m, 6H), 3.85–3.80 (m, 2H), 3.80–3.68 (m, 3H), 3.67–3.52 (m, 5H), 3.50 (td, *J* = 10.2, 9.6, 3.6 Hz, 2H), 3.48–3.42 (m, 2H), 3.38 (td, *J* = 9.7, 2.0 Hz, 1H), 3.35–3.20 (m, 5H), 3.14 (dd, *J* = 9.5, 4.1 Hz, 1H), 3.08 (dq, *J* = 9.8, 2.4 Hz, 2H), 1.97 (s, 3H), 1.80 (s, 3H), 1.71–1.44 (m, 4H), 1.38–1.16 (m, 2H). ^{13}C NMR (151 MHz, $CDCl_3$) δ 171.2, 170.2, 169.9, 156.9, 156.3, 139.9, 139.4, 138.7, 138.6, 138.5, 138.4, 138.0 (2C), 137.3, 137.2, 135.1, 128.9, 128.8, 128.7 (5C), 128.6 (5C), 128.5 (3C), 128.4 (3C), 128.3 (4C), 128.2, 128.1 (2C), 128.0 (3C), 127.9 (2C), 127.8 (4C), 127.7 (2C), 127.6, 127.5, 127.3, 127.1, 126.9, 102.9, 101.3, 99.3, 97.1, 94.6, 83.5, 82.5, 82.3, 81.6, 80.6, 80.0, 79.2, 78.4, 77.2, 76.3, 76.0, 75.9, 75.3, 75.1, 75.0, 74.9 (2C), 74.7, 74.5, 74.4, 73.9, 73.6, 73.5, 73.4, 73.2, 71.2, 70.2, 69.7, 68.8, 68.5, 68.0, 67.5, 67.4, 67.3, 66.3, 63.4, 52.7, 50.7, 50.4, 47.3, 46.3, 29.8, 29.2, 28.1, 27.7, 23.6 (2), 21.0.

α-D-Glucopyranosyluronate-(1→3)-α-D-galactopyranosyl-(1→3)-2-acetamido-6-*O*-acetyl-2-deoxy-β-D-mannopyranosyl-(1→4)-β-D-glucopyranosyl-(1→4)-α-D-glucopyranosyl-(1→1)-(5-amino)pentanol (5)

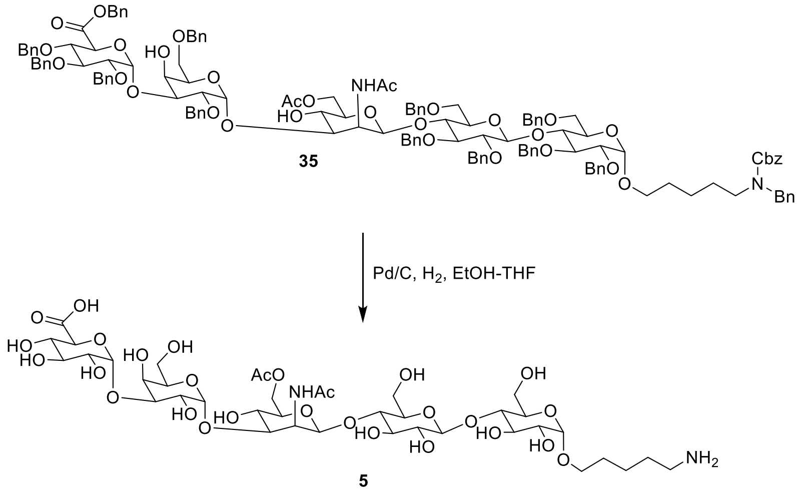

Substrate **35** (38 mg, 0.016 mmol) was taken in THF (1 mL), treated with a suspension of Pd/C (40 mg) in ethanol (2 mL) and stirred under hydrogen pressure using a balloon for 48 h. The RM was then filtered through a PTFE filter and washed thoroughly with ethanol/water. Evaporation of solvent yielded a colorless material. ^{1}H NMR of the crude showed that the reaction was not complete. The crude was resubjected to hydrogenolysis for additional 24 h under the same conditions. The RM was then filtered through a PTFE filter and washed thoroughly with ethanol/water. Evaporation of the solvent yielded a colorless crude product which was then purified using a C_{18}-sepak column with water and ethanol as the eluents. Lyophilization of the water fraction yielded the pentasaccharide RU **5** as a white fluffy solid (5.5 mg, 33%). HRMS (ESI): calculated for $C_{39}H_{66}N_2NaO_{28}$ [M + Na]$^+$, 1033.3700, found 1033.3767. ^{1}H NMR (600 MHz, D_2O) δ 5.33 (d, *J* = 3.9 Hz, 1H, αGal-H1), 5.13 (d, *J* = 3.8 Hz, 1H, αGlcA-H1), 4.92 (d, *J* = 2.1 Hz, 2H, αGlc-H1 and βManNAc-H1), 4.67 (dd, *J* = 4.6, 1.6 Hz, 1H, βManNAc-H2), 4.52 (d, *J* = 8.0 Hz, 1H, βGlc-H1), 4.51 – 4.48 (m, 1H, ManNAc-H6a/b), 4.31 (dd, *J* = 12.2, 6.5 Hz, 1H, ManNAc-H6a/b), 4.23 – 4.19 (m, 2H), 4.17 (t, *J* = 6.3 Hz, 1H), 4.01 (dd, *J* = 9.6, 4.6 Hz, 1H, βManNAc-H3), 3.96 (dd, *J* = 10.4, 3.8 Hz, 1H, αGal-H2), 3.93 – 3.60 (m, 18H), 3.59 – 3.51 (m, 3H), 3.39 – 3.31 (m, 1H, βGlc-H2), 3.02 (t, *J* = 7.6 Hz, 2H), 2.23 – 2.15 (m, 3H), 2.07 (s, 3H), 1.79 – 1.63 (m, 4H), 1.55 – 1.41 (m, 2H). ^{13}C NMR (151 MHz, D_2O) δ 177.7, 176.7, 104.9 (βGlc-C1), 102.8 (αGal-C1), 102.3 (βManNAc-C1), 100.4 (αGlc-C1), 97.3 (αGlcA-C1), 81.9, 81.3, 79.2, 77.0, 76.6, 76.5, 76.3, 75.6, 75.2, 74.8, 74.2, 73.9, 73.8, 73.6, 73.1, 70.5, 69.6, 69.3, 68.1, 66.2, 65.1, 64.1, 62.6, 62.5, 55.3, 42.0, 30.6, 29.1, 25.1, 24.6, 22.9.

3. References

1) Ghosh, S.; Misra, A. K. *J. Carbohydr. Chem.* **2009**, *28*, 447-462.
2) B. Schumann, S. G. Parameswarappa, M. P. Lisboa, N. Kottari, F. Guidetti, C. L. Pereira, P. H. Seeberger, *Angew. Chem. Int. Ed.* **2016**, *55*, 14431-14434.
3) Rathwell, D.; Parameswarappa, S. G.; Govindan, S.;Anish, C; Pereira, C. L.; Seeberger, P. H. *Protein and peptide-free synthetic vaccines against streptococcus pneumoniae type 3* EP 2851092A1 Mar 3, **2015**.
4) Pereira, C. L.; Geissner, A.; Anish, C.; Seeberger, P. H. *Angew. Chem. Int. Ed*. **2015**, *54*, 10016 –10019.
