# Supplementary material for: Synthesis of Streptococcus pneumoniae serotype 9V oligosaccharide antigens
Source: Beilstein J Org Chem. 2020 Jul 15;16:1693–9. doi: 10.3762/bjoc.16.140 (PMC7372248; doi:10.3762/bjoc.16.140)

BEILSTEIN JOURNAL OF ORGANIC CHEMISTRY

Supporting Information

for

Synthesis of *Streptococcus pneumoniae* serotype 9V oligosaccharide antigens

Sharavathi G. Parameswarappa, Claney L. Pereira and Peter H. Seeberger

Beilstein J. Org. Chem. **2020,** *16,* 1693–1699. doi:10.3762/bjoc.16.140

NMR Spectra

License and Terms: This is a supporting information file under the terms of the Creative Commons Attribution License (http://creativecommons.org/licenses/by/4.0). Please note that the reuse, redistribution and reproduction in particular requires that the authors and source are credited.
The license is subject to the *Beilstein Journal of Organic Chemistry* terms and conditions: (https://www.beilstein-journals.org/bjoc)

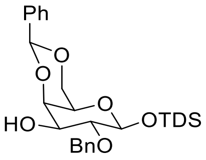

9

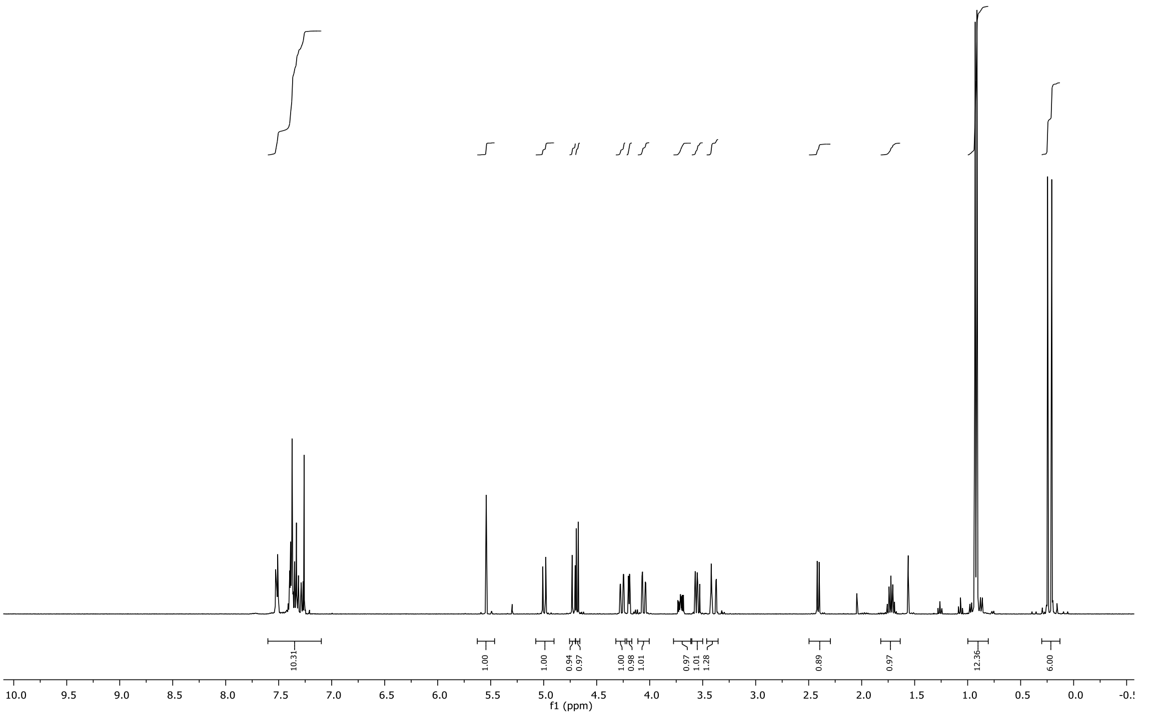

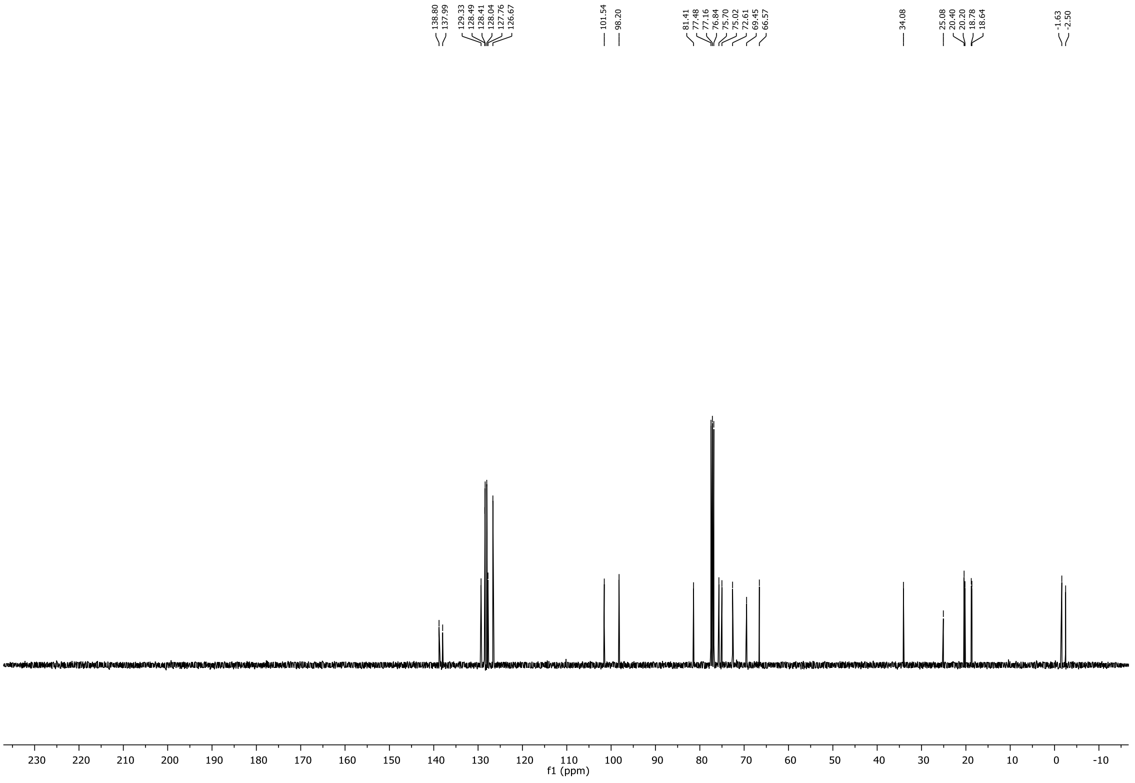

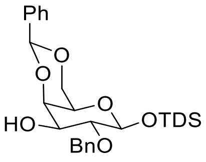

9

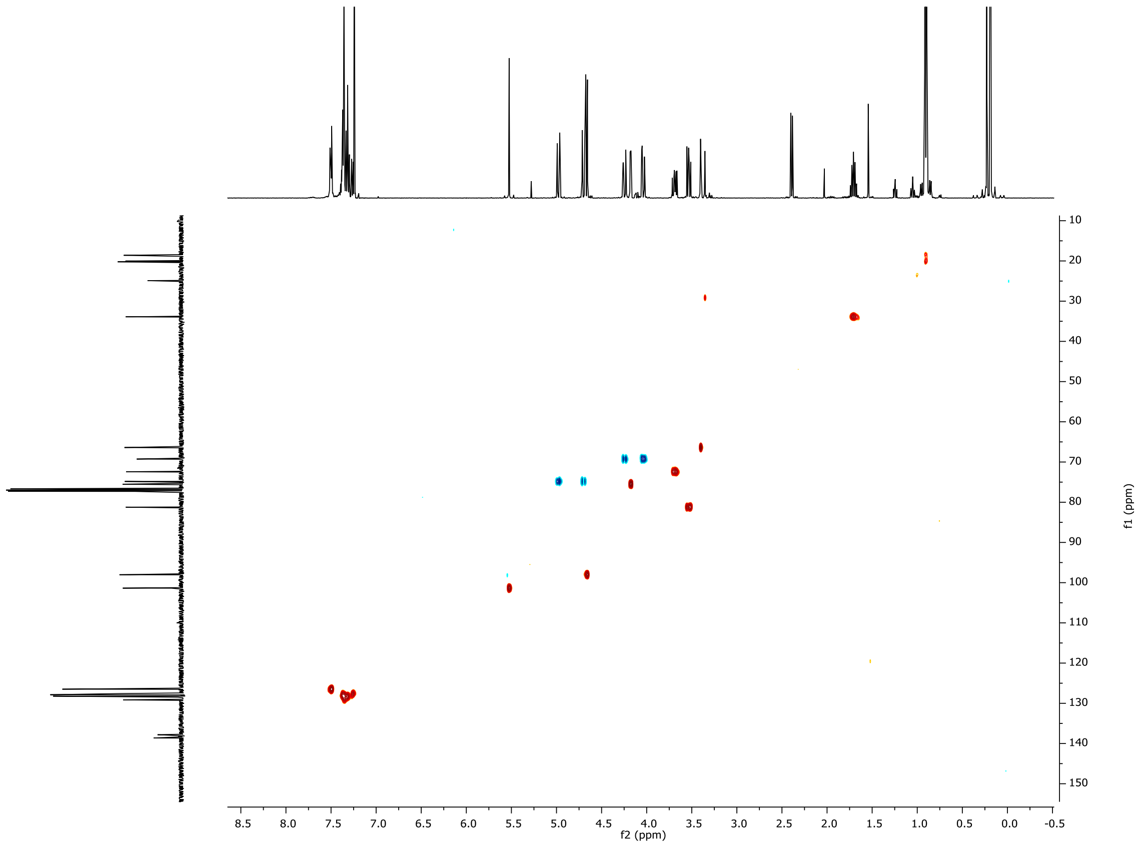

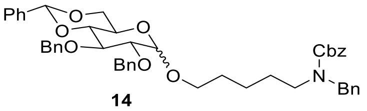

14

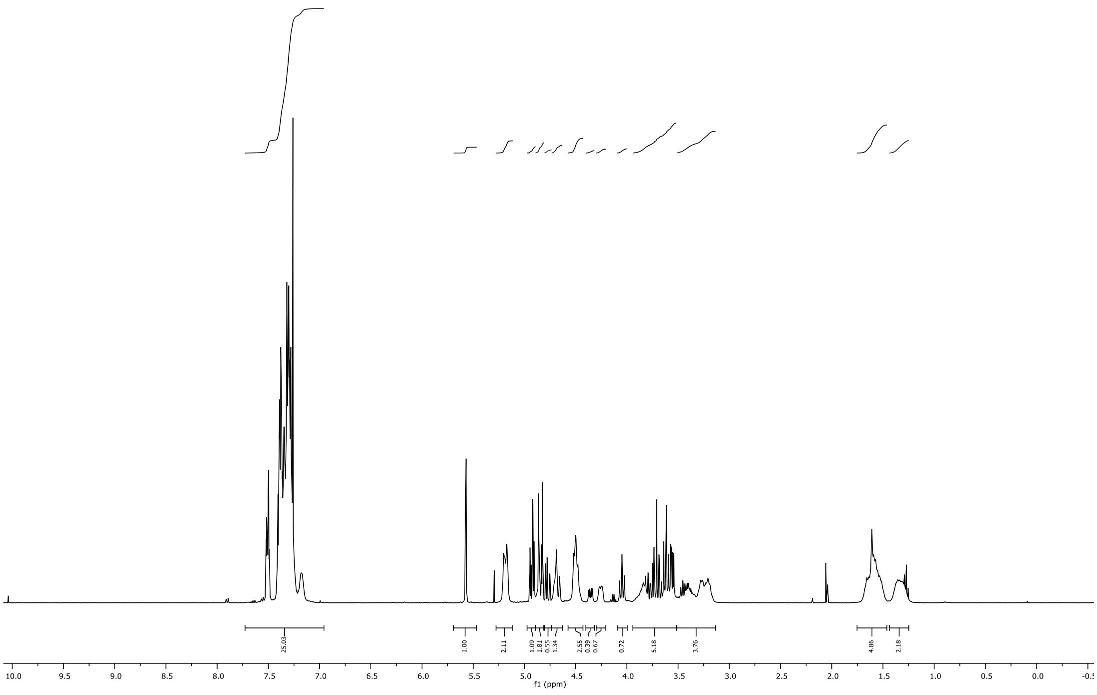

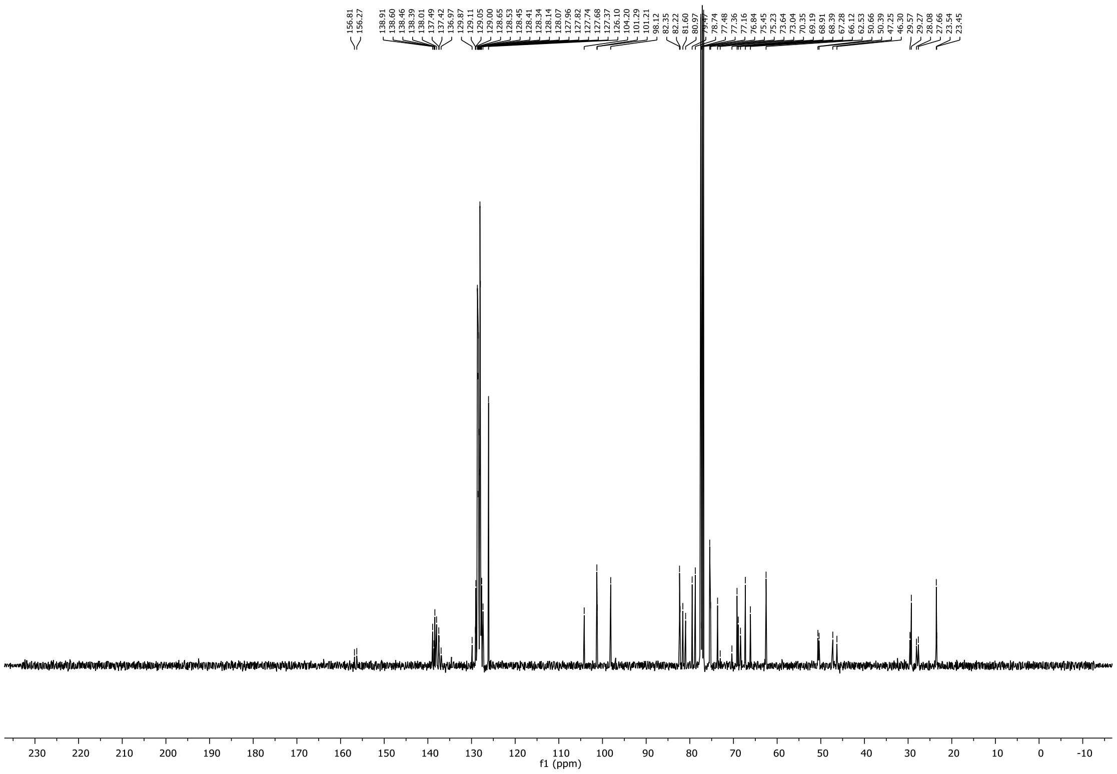

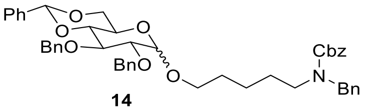

14

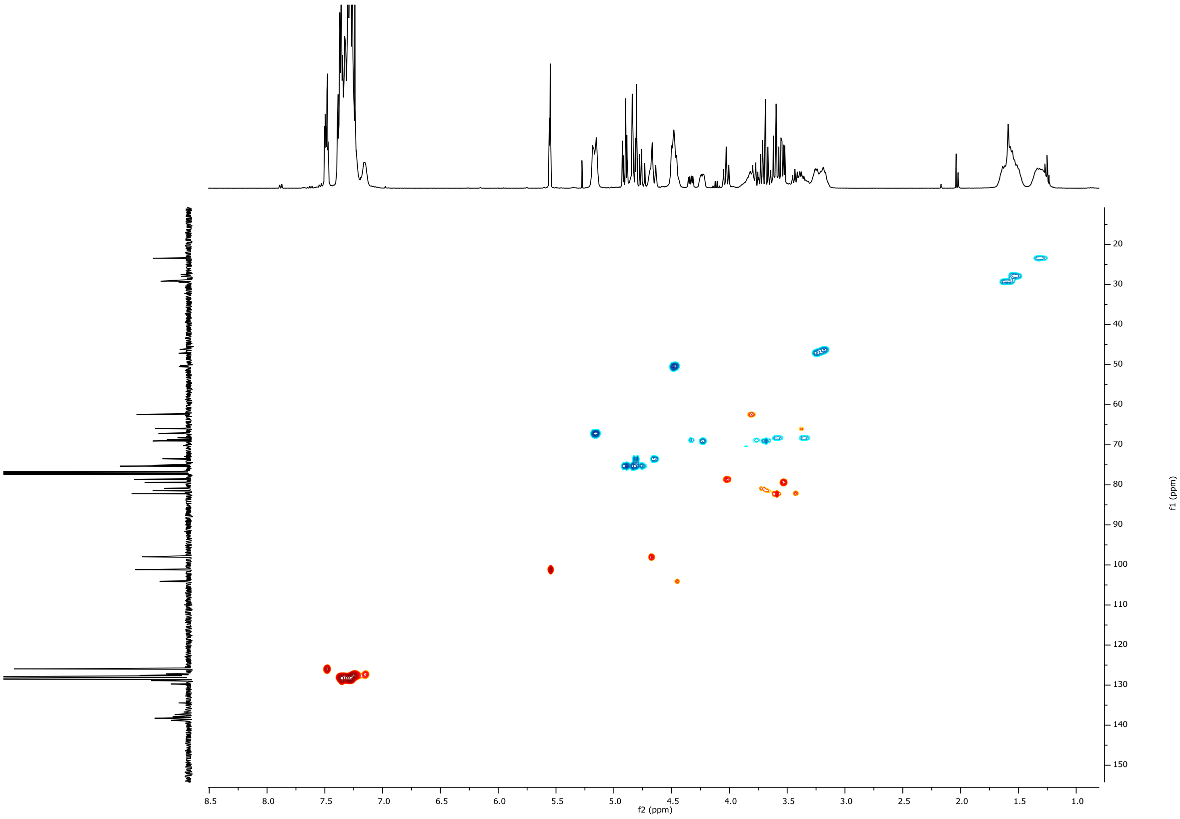

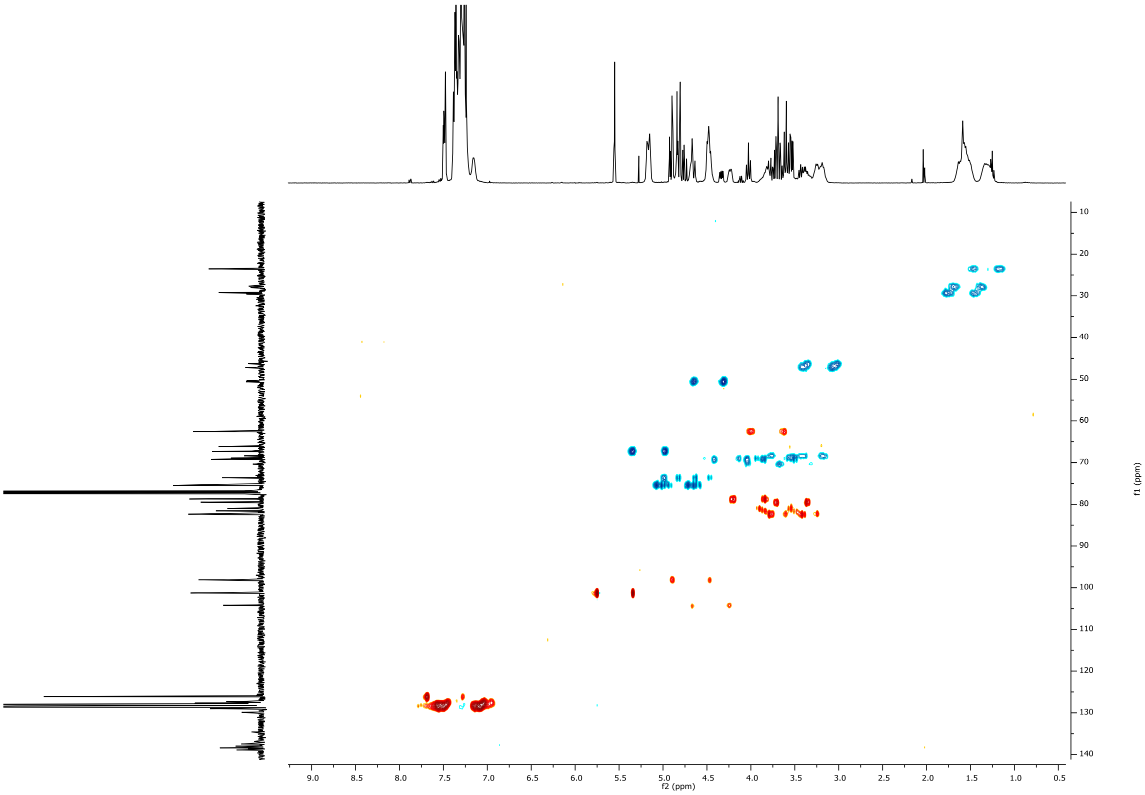

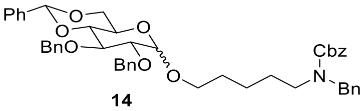
Ph
O
O
O
BnO
BnO
O
Cbz
N
Bn

14

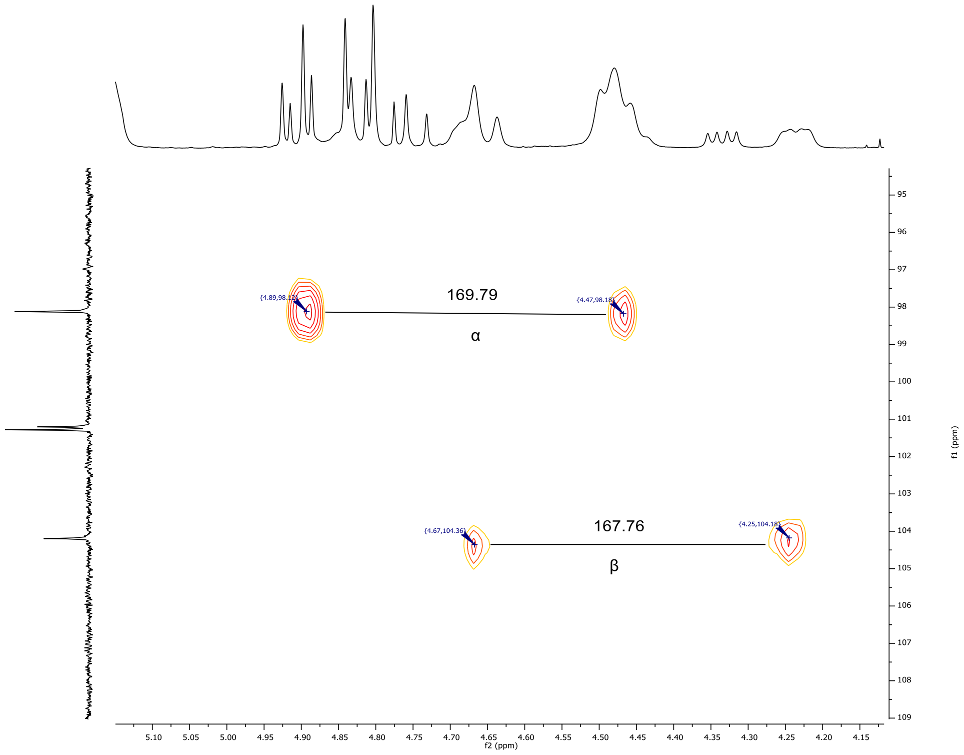
(4.89,98.17)
169.79
(4.47,98.18)
α
(4.67,104.36)
167.76
(4.25,104.18)
β
f1 (ppm)
f2 (ppm)

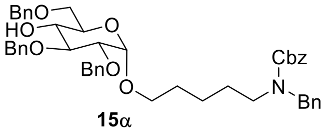
BnO
HO
BnO
BnO
O
Cbz
N
Bn

15α

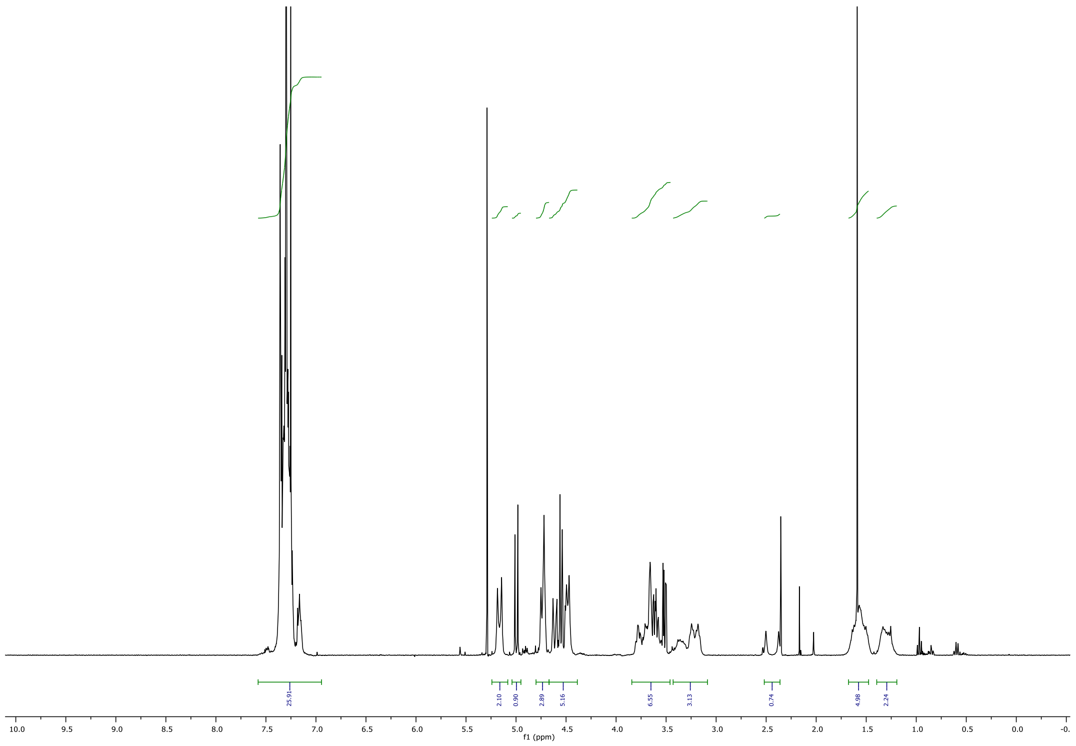
25.91
2.10
0.90
2.89
5.16
6.55
3.13
0.74
4.98
2.24
f1 (ppm)

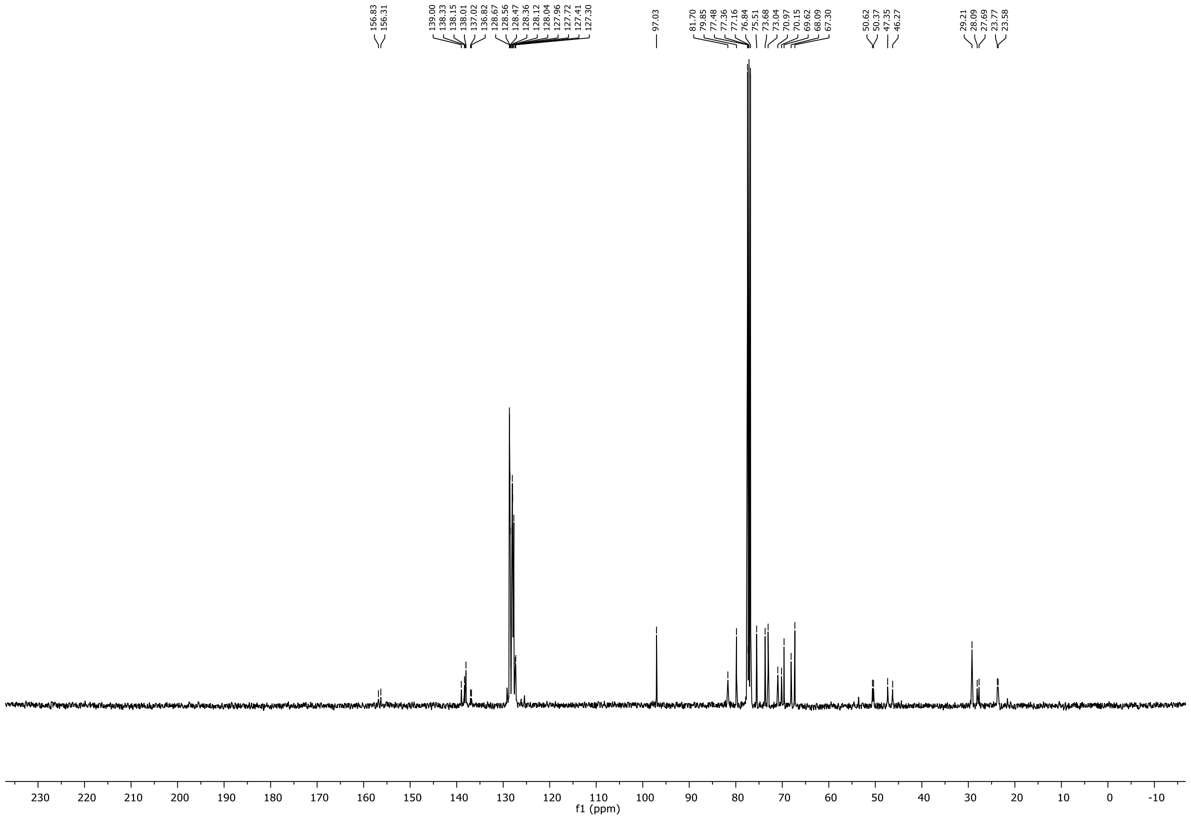
156.83
156.31
139.00
138.33
138.15
138.01
137.02
136.82
128.67
128.56
128.47
128.36
128.12
128.04
127.96
127.72
127.41
127.30
97.03
81.70
79.85
77.48
77.36
77.16
76.84
75.51
73.68
73.04
70.97
70.15
69.62
68.09
67.30
50.62
50.37
47.35
46.27
29.21
28.09
27.69
23.77
23.58
f1 (ppm)

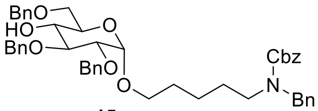

15α

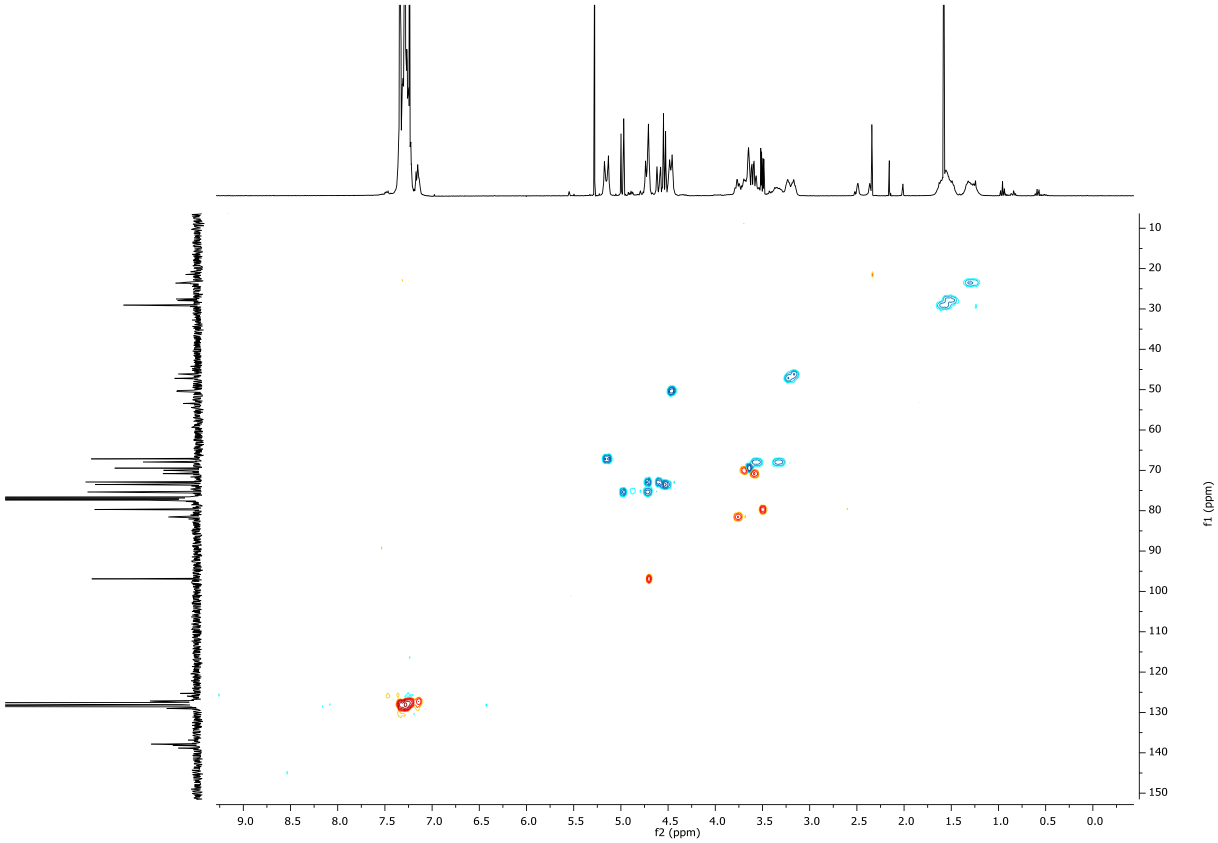

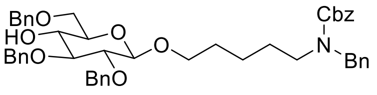

15β

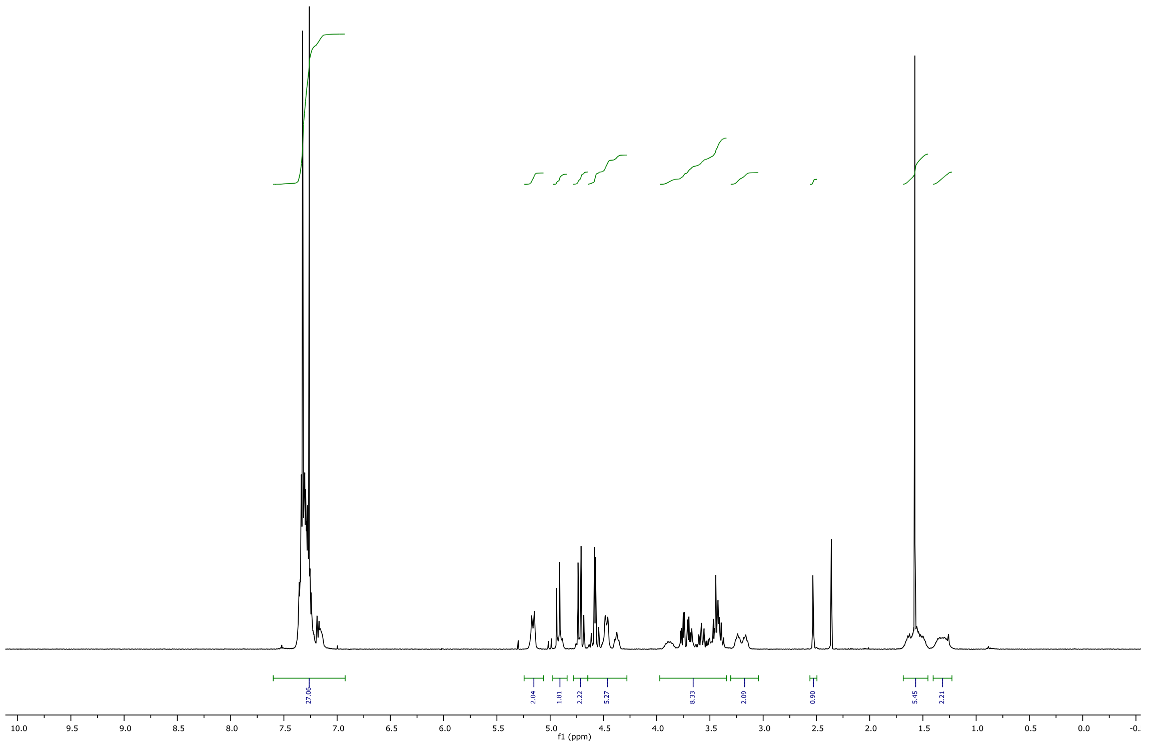

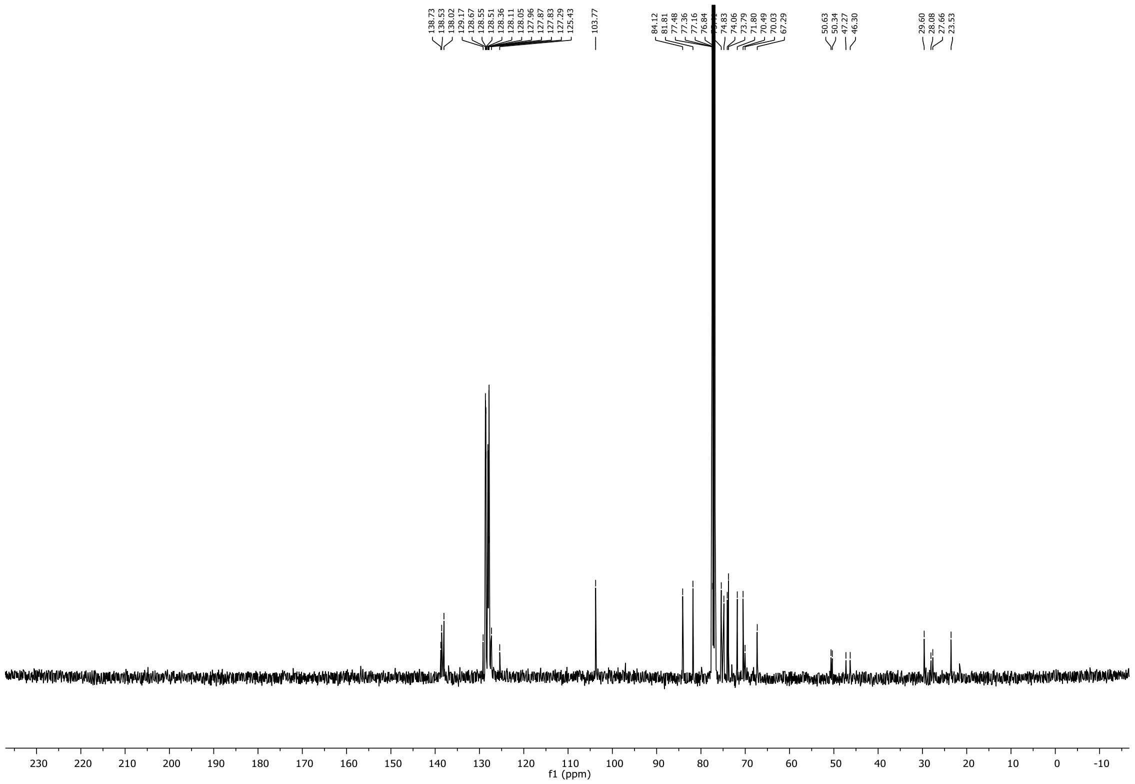

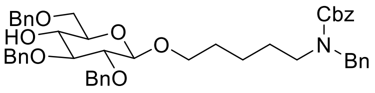

15β

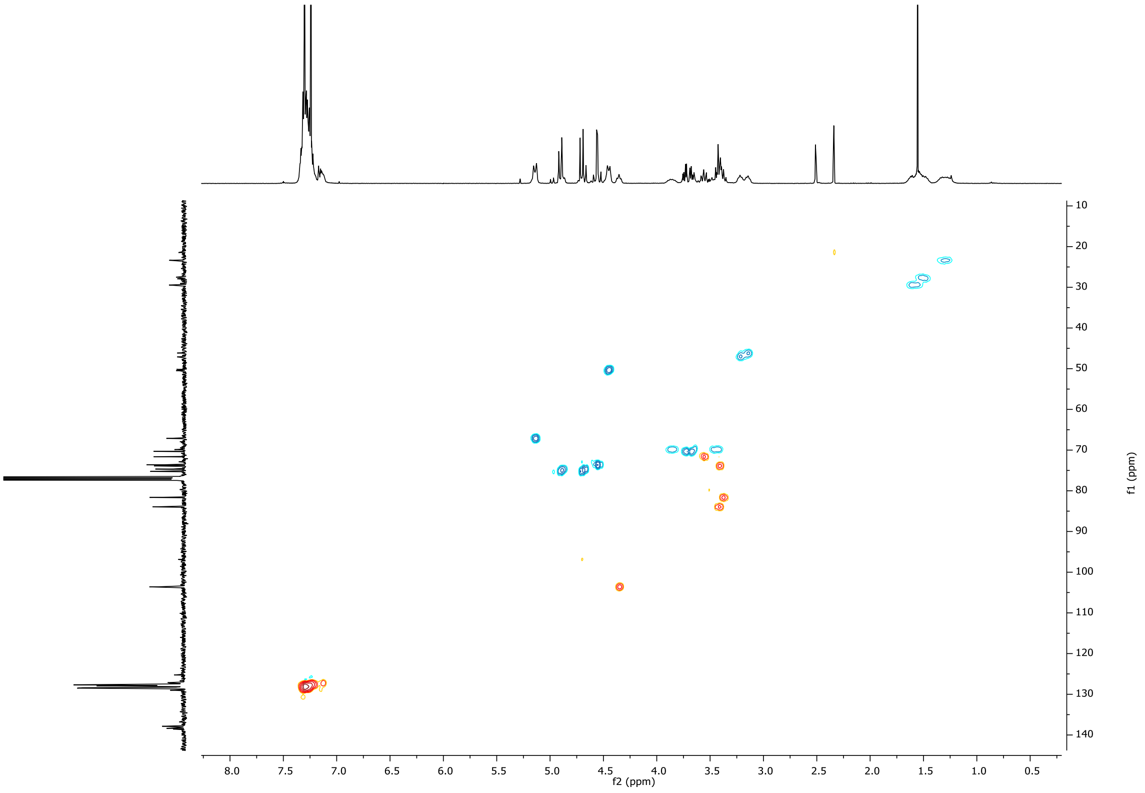

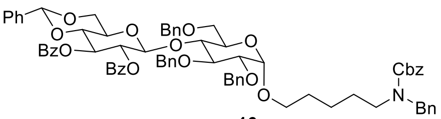
Ph
O
O
BzO
BzO
O
BnO
O
BnO
O
BnO
O
Cbz
N
Bn

16

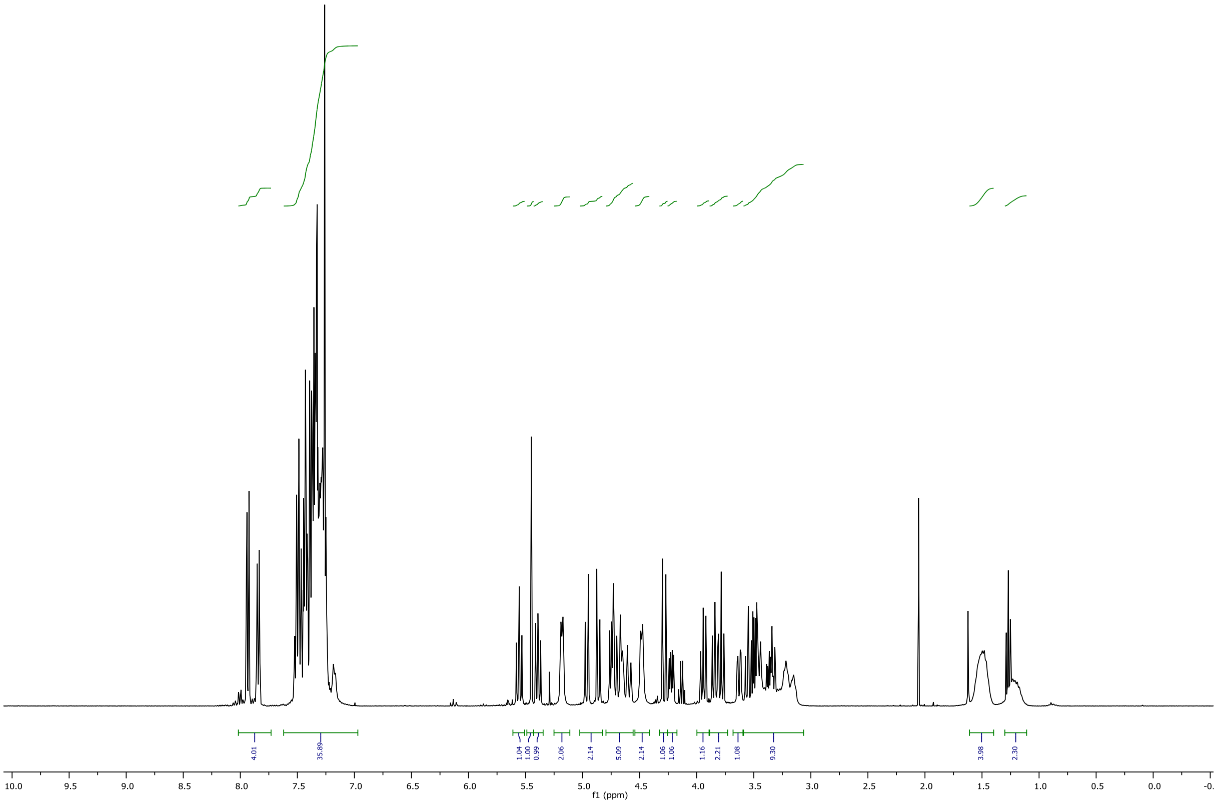
4.01
35.89
1.04
1.00
0.99
2.06
2.14
5.09
2.14
1.06
1.06
1.16
2.21
1.08
9.30
3.98
2.30
f1 (ppm)

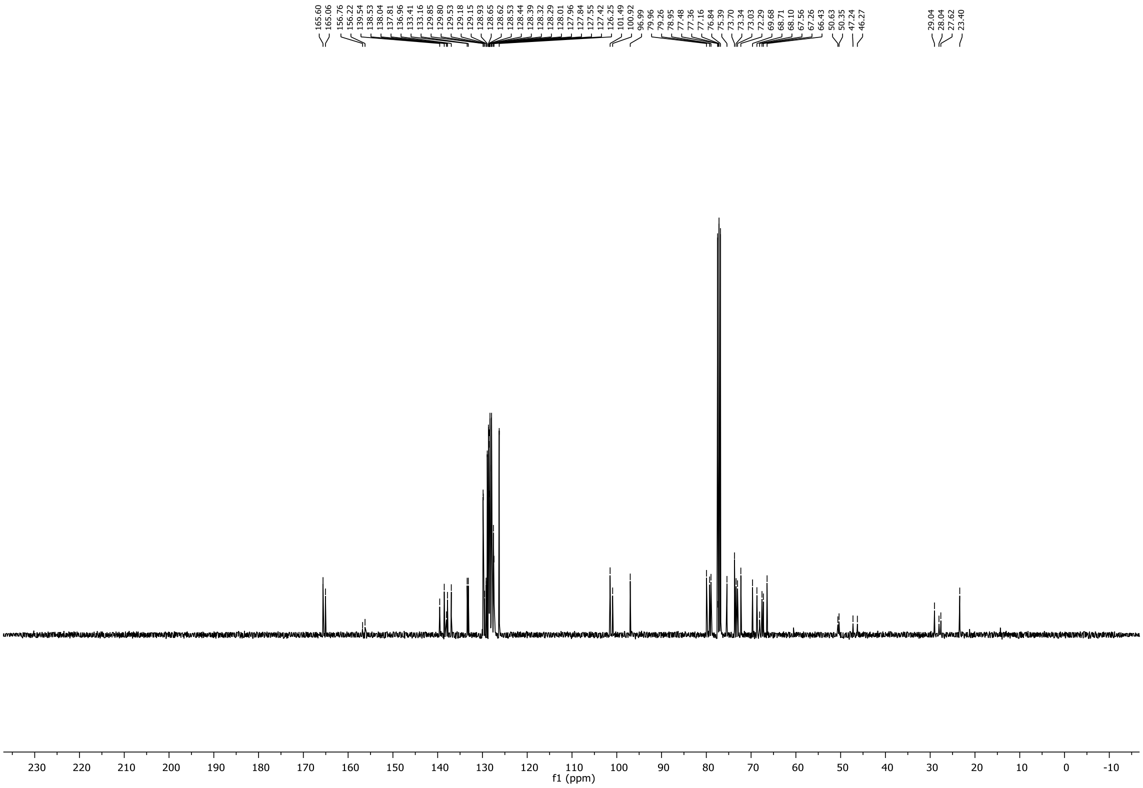
165.60
165.06
156.76
156.22
139.54
138.53
138.04
137.81
136.96
133.41
133.16
129.85
129.80
129.53
129.53
129.18
129.15
128.93
128.65
128.62
128.53
128.44
128.39
128.32
128.29
128.01
127.96
127.84
127.55
127.42
126.25
101.49
100.92
96.99
79.86
79.26
78.95
77.48
77.36
77.16
76.84
75.39
73.70
73.34
73.03
72.29
69.68
68.71
68.10
67.56
67.26
66.43
50.63
50.35
47.24
46.27
29.04
28.04
27.62
23.40
f1 (ppm)

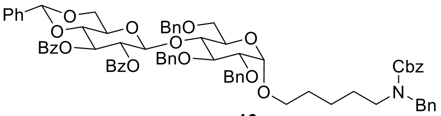
Ph
O
O
BzO
BzO
BnO
O
O
BnO
BnO
O
O
Cbz
N
Bn

16

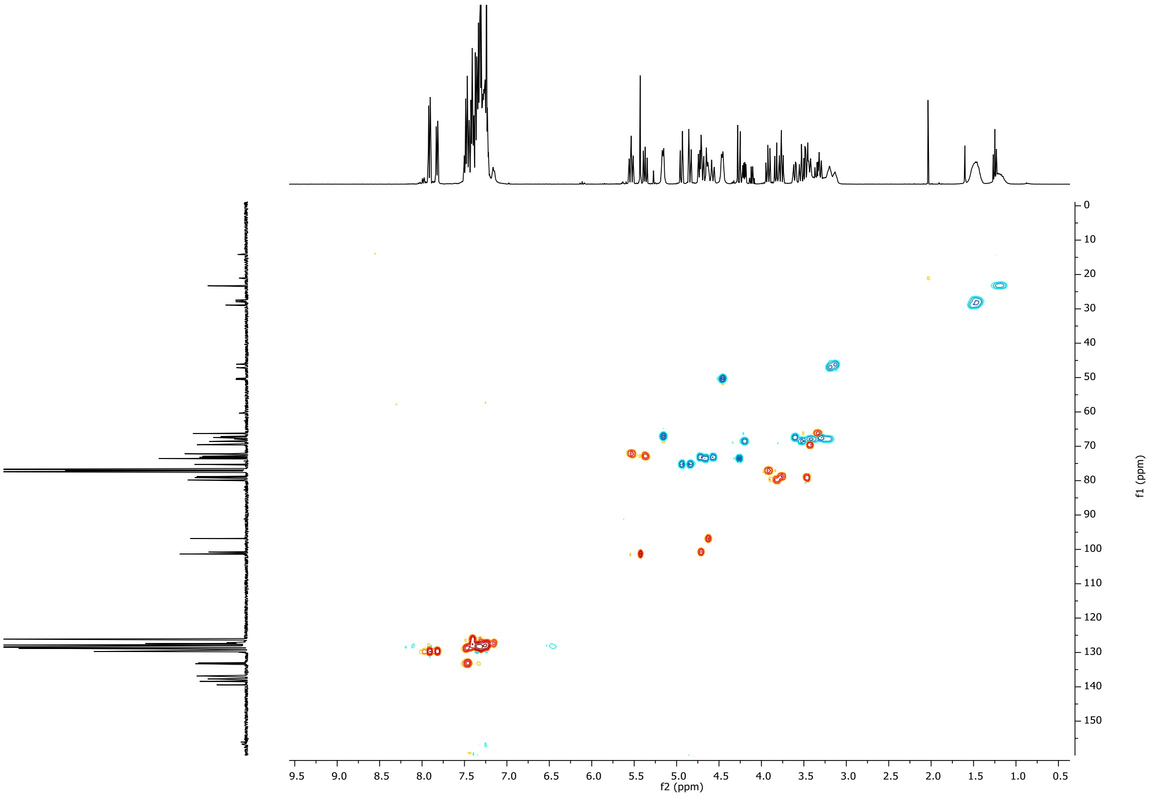
f1 (ppm)
f2 (ppm)
9.5
9.0
8.5
8.0
7.5
7.0
6.5
6.0
5.5
5.0
4.5
4.0
3.5
3.0
2.5
2.0
1.5
1.0
0.5
0
10
20
30
40
50
60
70
80
90
100
110
120
130
140
150

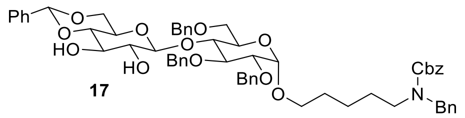
Ph
O
O
HO
HO
BnO
O
BnO
O
BnO
O
Cbz
N
Bn

17

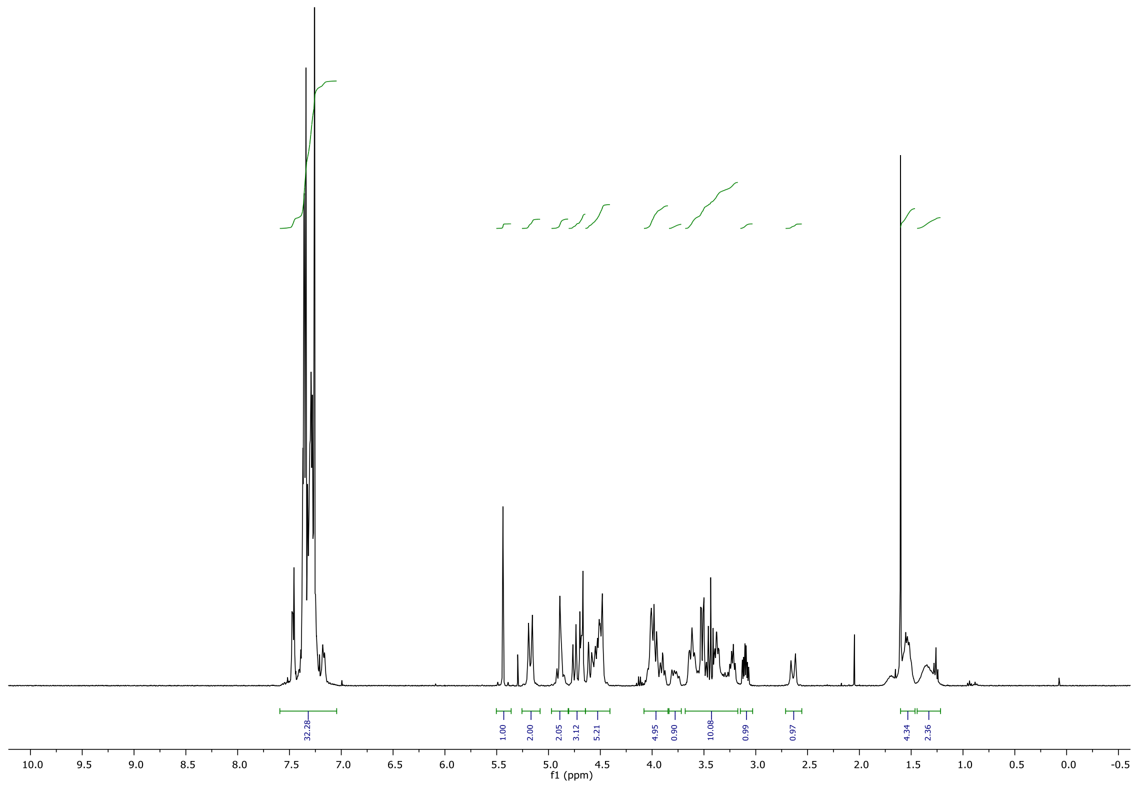
32.28
1.00
2.00
2.05
3.12
5.21
4.95
0.90
10.08
0.99
0.97
4.34
2.36
10.0 9.5 9.0 8.5 8.0 7.5 7.0 6.5 6.0 5.5 5.0 4.5 4.0 3.5 3.0 2.5 2.0 1.5 1.0 0.5 0.0 -0.5
f1 (ppm)

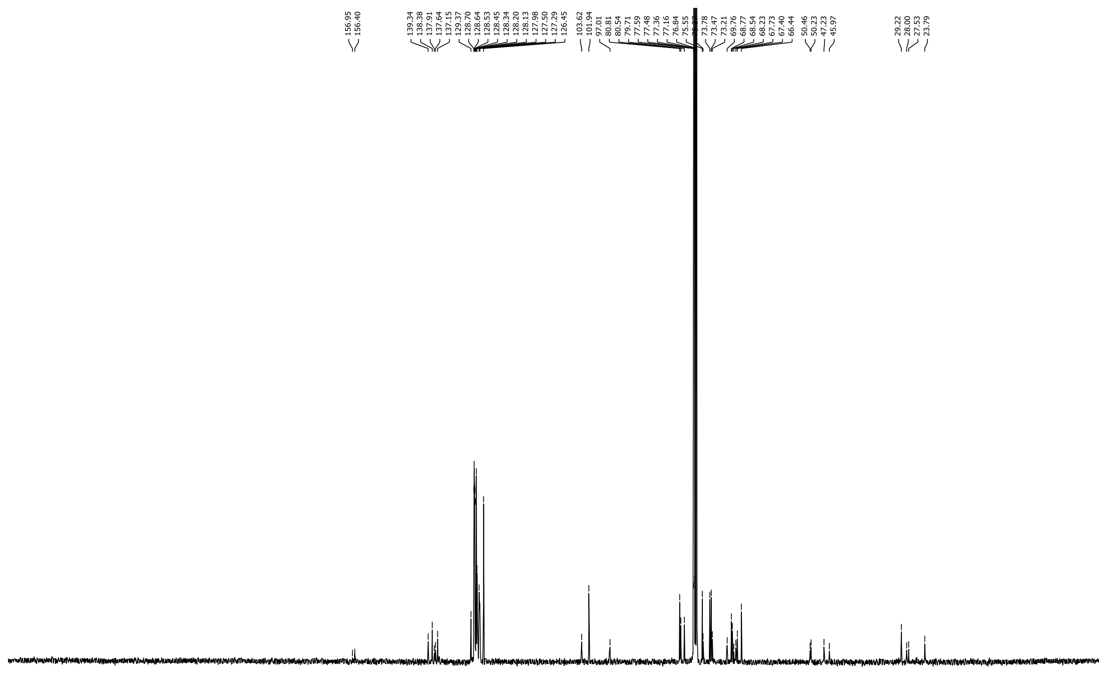
156.95
156.40
139.34
138.38
137.91
137.64
137.15
129.37
128.70
128.64
128.53
128.45
128.34
128.20
128.13
127.98
127.50
127.29
126.45
103.62
101.94
97.01
80.81
80.54
79.71
77.59
77.48
77.36
77.16
76.84
75.55
73.78
73.47
73.21
69.76
68.77
68.54
68.23
67.73
67.40
66.44
50.46
50.23
47.23
45.57
29.22
28.00
27.53
23.79
230 220 210 200 190 180 170 160 150 140 130 120 110 100 90 80 70 60 50 40 30 20 10 0 -10
f1 (ppm)

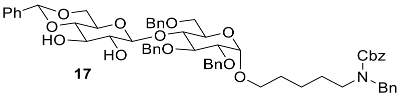

17

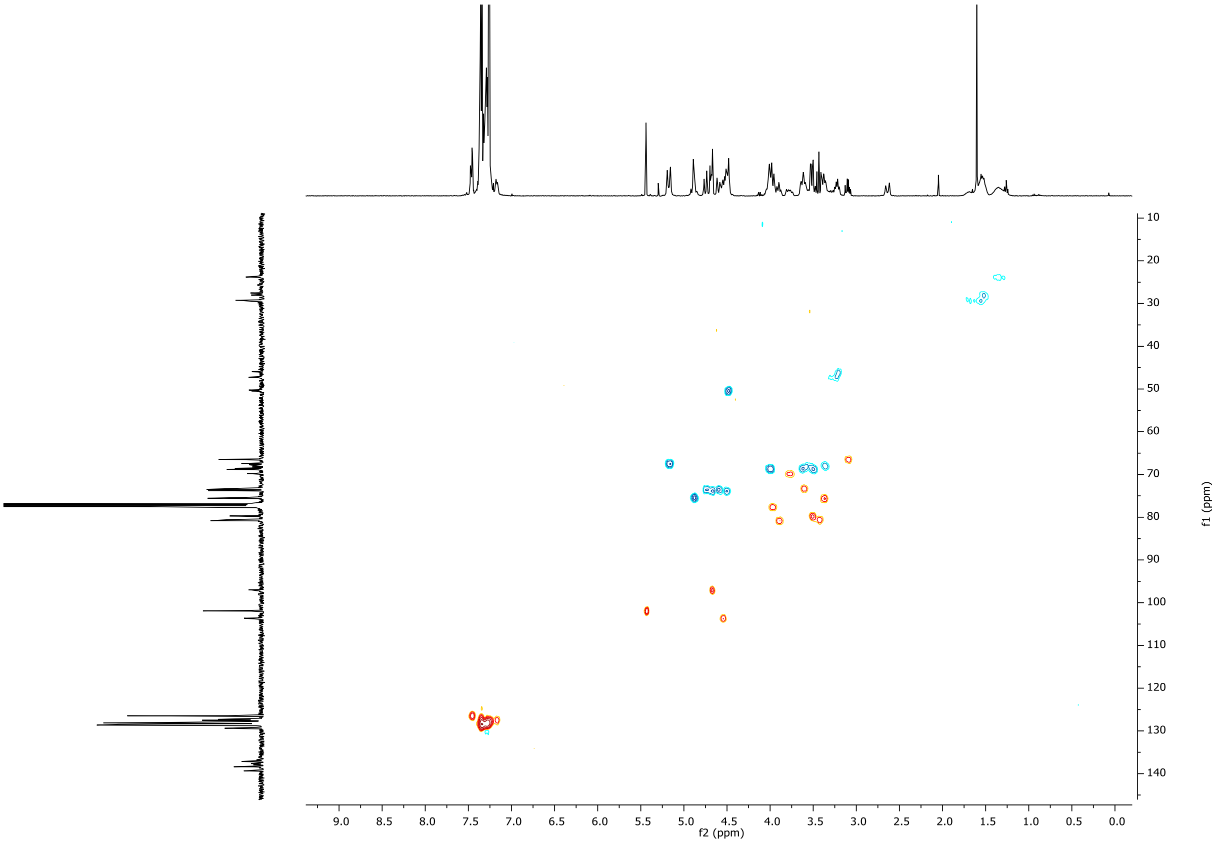

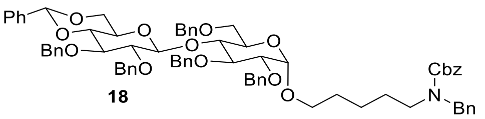
Ph
BnO
BnO
BnO
BnO
BnO
O
Cbz
N
Bn

18

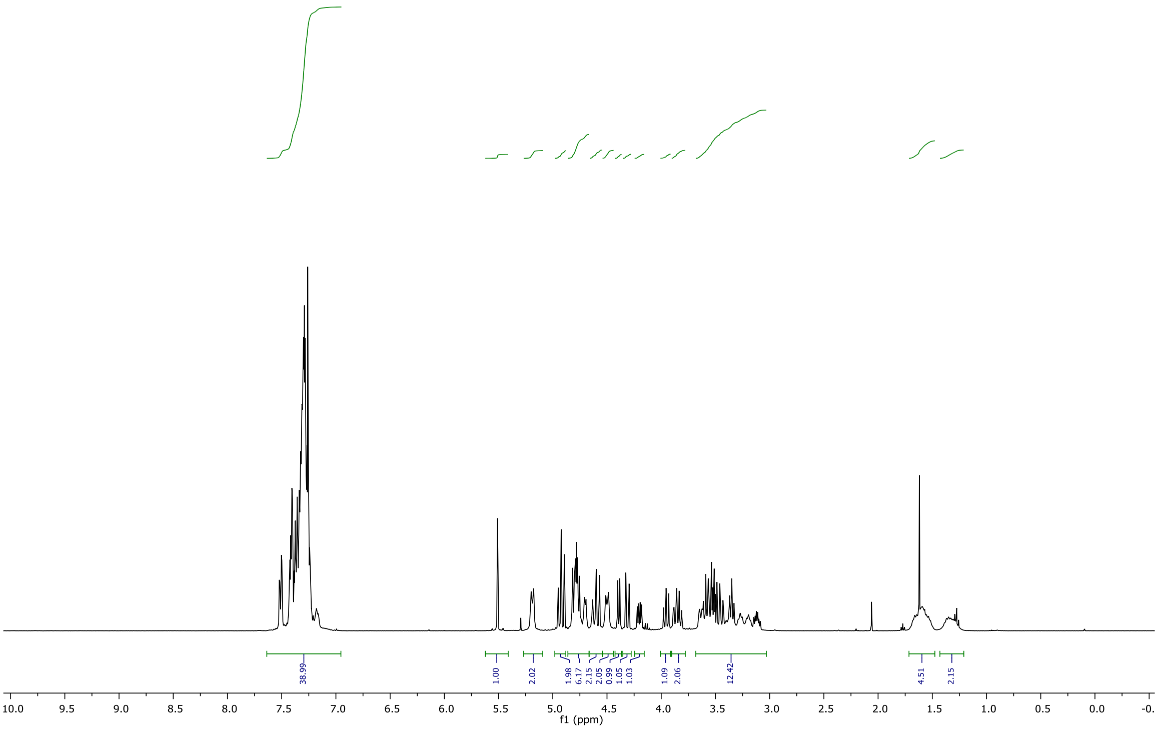
38.99
1.00
2.02
1.98
6.17
2.15
2.05
0.99
1.05
1.03
1.09
2.06
12.42
4.51
2.15
10.0 9.5 9.0 8.5 8.0 7.5 7.0 6.5 6.0 5.5 5.0 4.5 4.0 3.5 3.0 2.5 2.0 1.5 1.0 0.5 0.0 -0.
f1 (ppm)

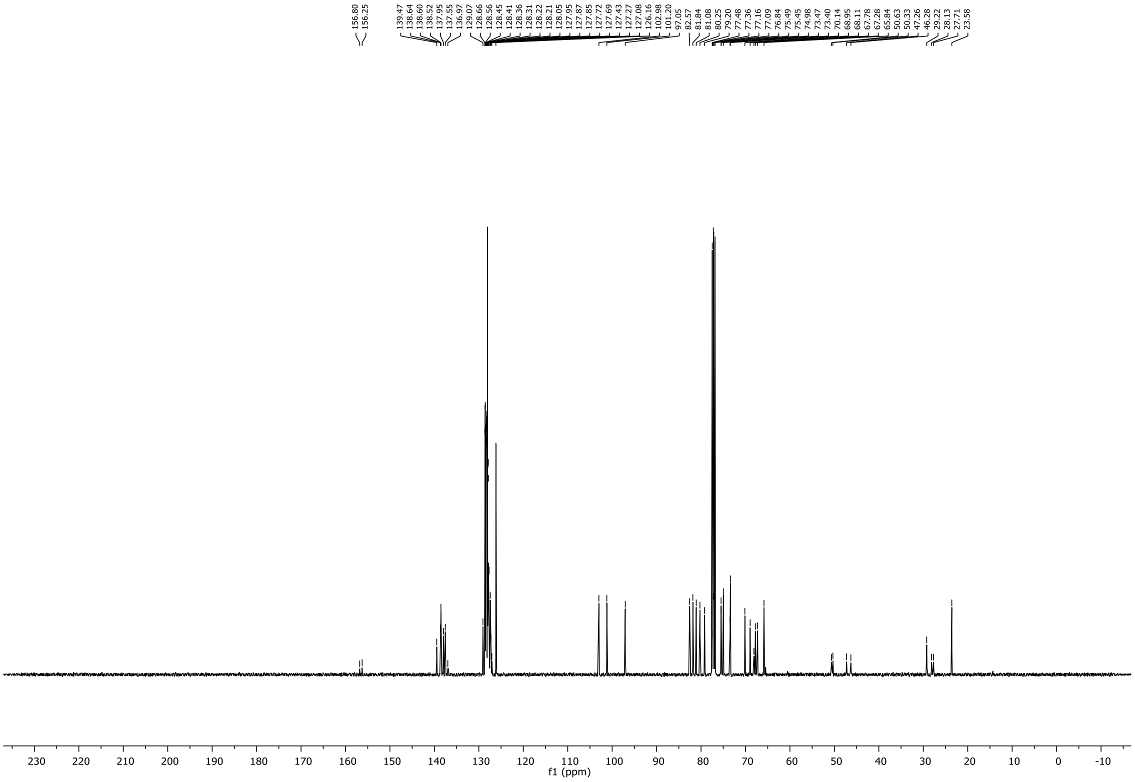
156.80
156.25
139.47
138.64
138.60
138.52
137.95
137.55
136.97
129.07
128.66
128.56
128.45
128.41
128.36
128.31
128.22
128.21
128.05
127.95
127.87
127.85
127.72
127.69
127.43
127.27
127.08
126.16
102.98
101.20
97.05
82.57
81.84
81.08
80.25
79.20
77.48
77.36
77.16
77.09
76.84
75.49
75.45
74.98
73.47
73.40
70.14
68.95
68.11
67.78
67.28
65.84
50.63
50.33
47.26
46.28
29.22
28.13
27.71
23.58
230 220 210 200 190 180 170 160 150 140 130 120 110 100 90 80 70 60 50 40 30 20 10 0 -10
f1 (ppm)

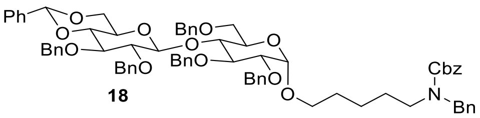

18

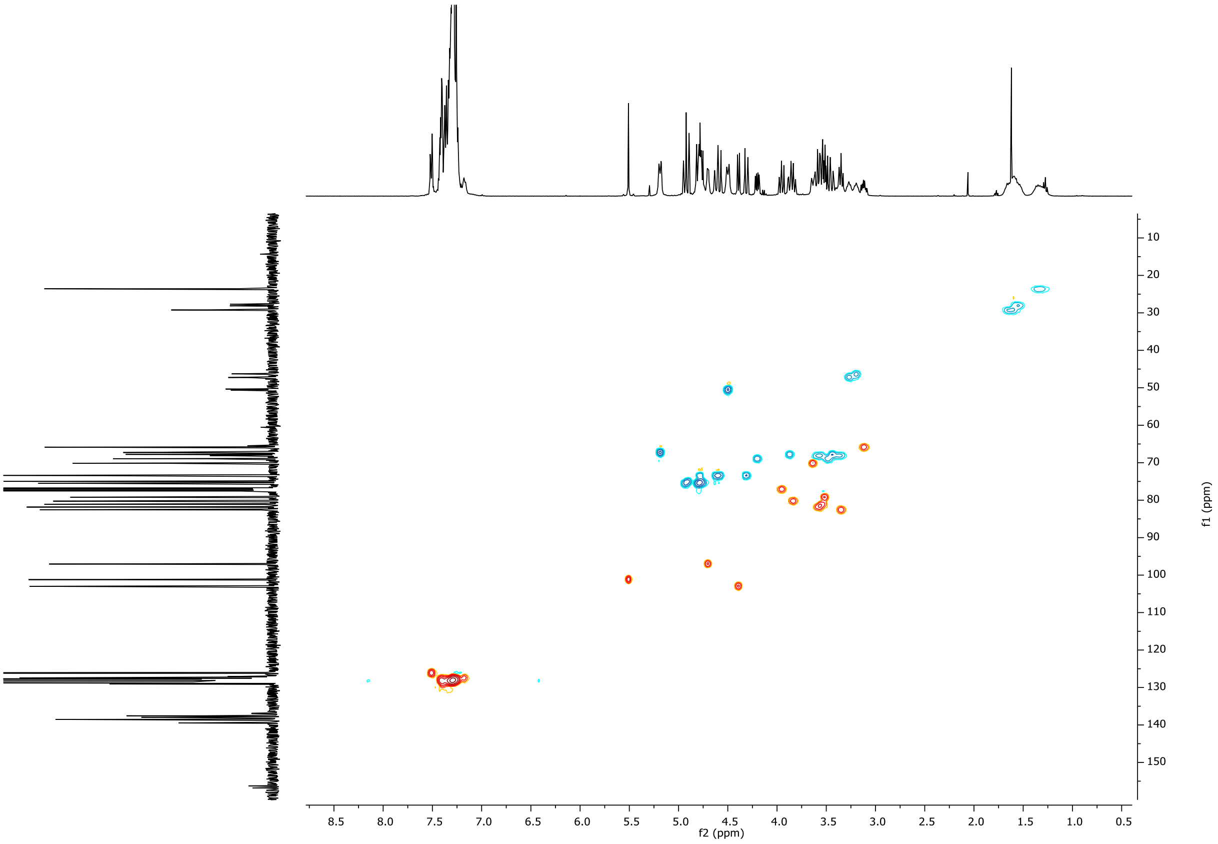

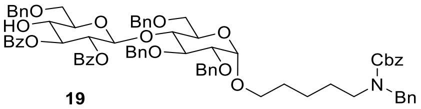
BnO
HO
BzO
BzO
BnO
BnO
BnO
O
Cbz
N
Bn

19

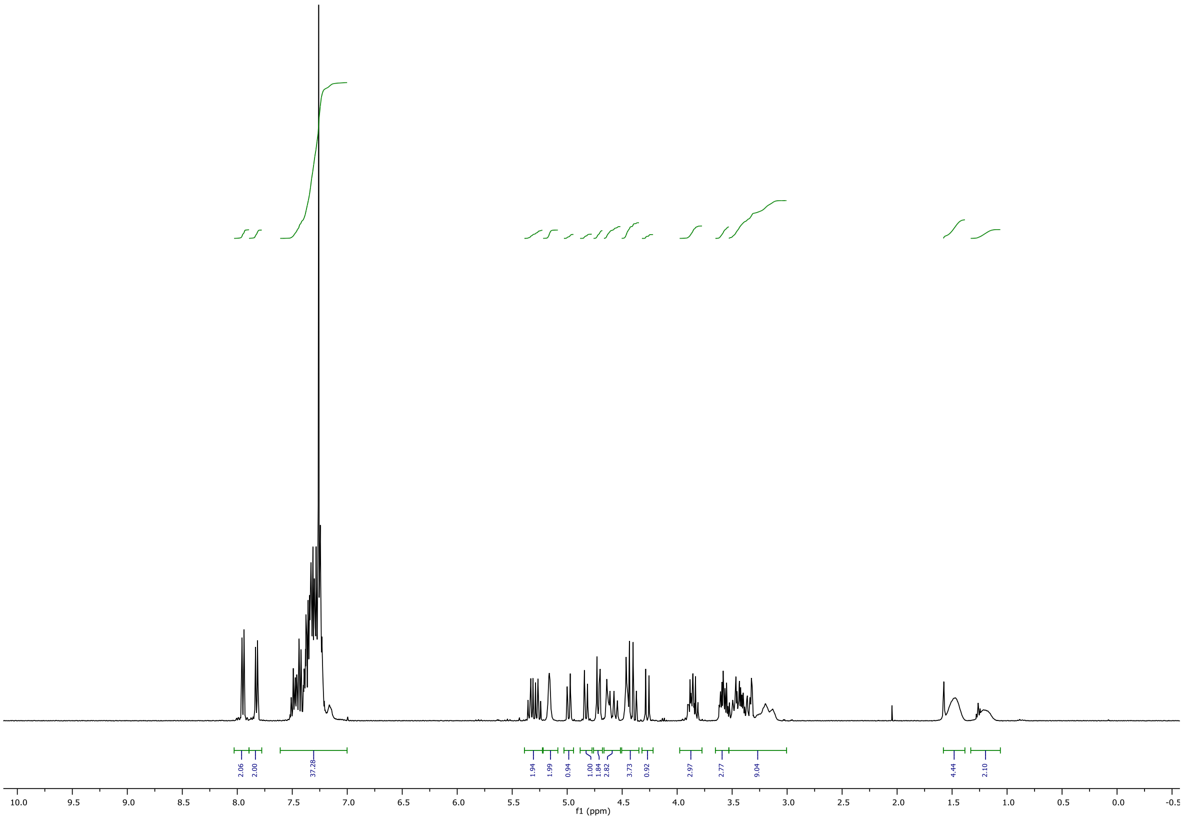
2.06
2.00
37.28
1.94
1.99
0.94
1.00
1.84
2.82
3.73
0.92
2.97
2.77
9.04
4.44
2.10
f1 (ppm)

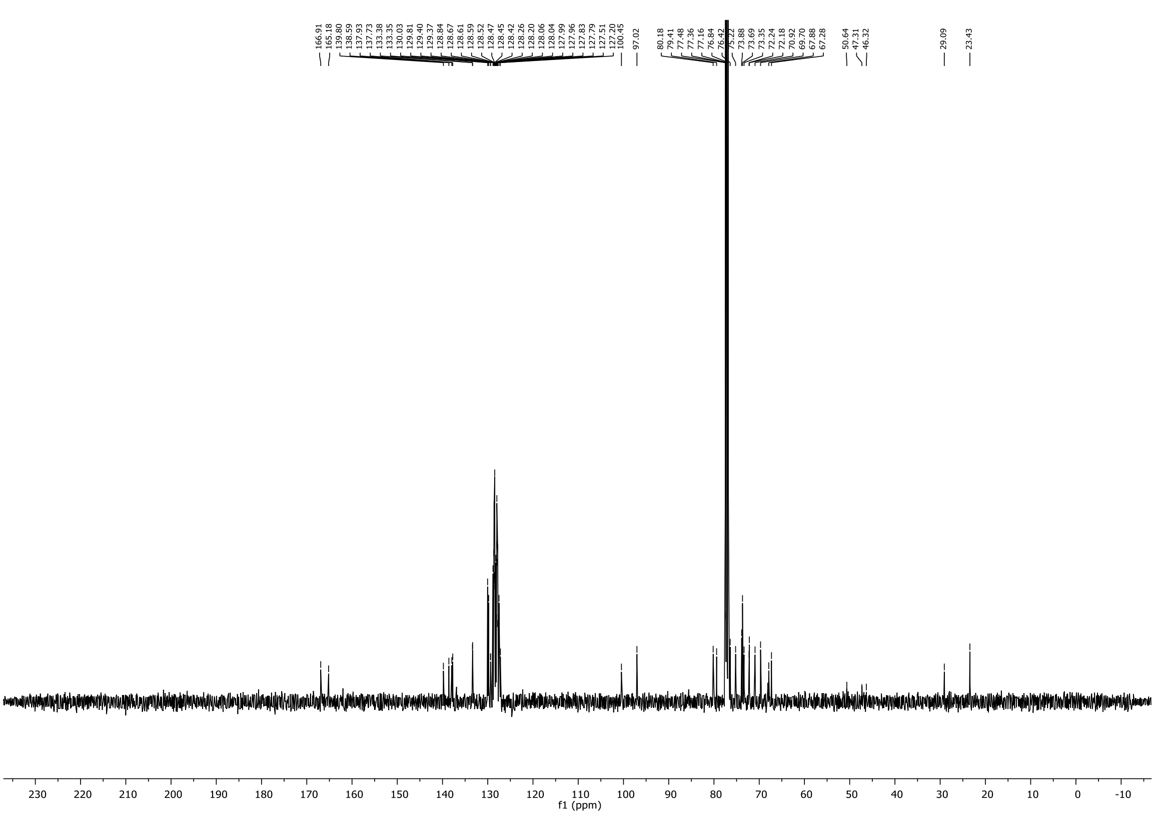
166.91
165.18
139.80
138.59
137.93
137.73
133.38
133.35
130.03
129.81
129.40
129.37
128.84
128.67
128.61
128.59
128.52
128.47
128.45
128.42
128.26
128.20
128.06
128.04
127.99
127.96
127.83
127.79
127.51
127.20
100.45
97.02
80.18
79.41
77.48
77.36
77.16
76.84
73.88
73.69
73.35
72.24
72.18
70.92
69.70
67.88
67.28
50.64
47.31
46.32
29.09
23.43
f1 (ppm)

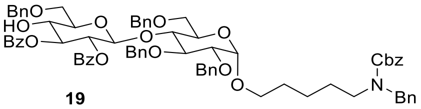

19

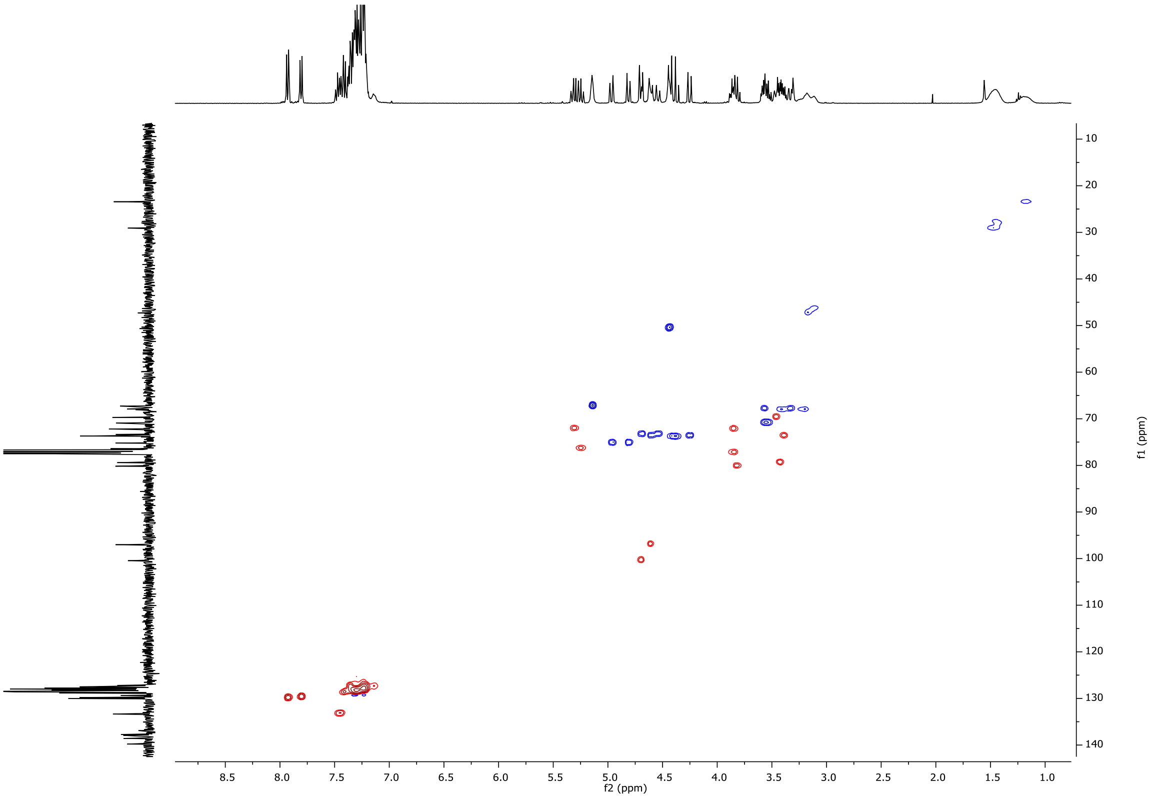

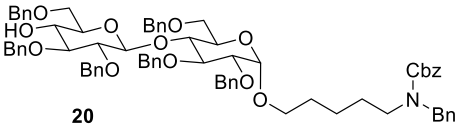

20

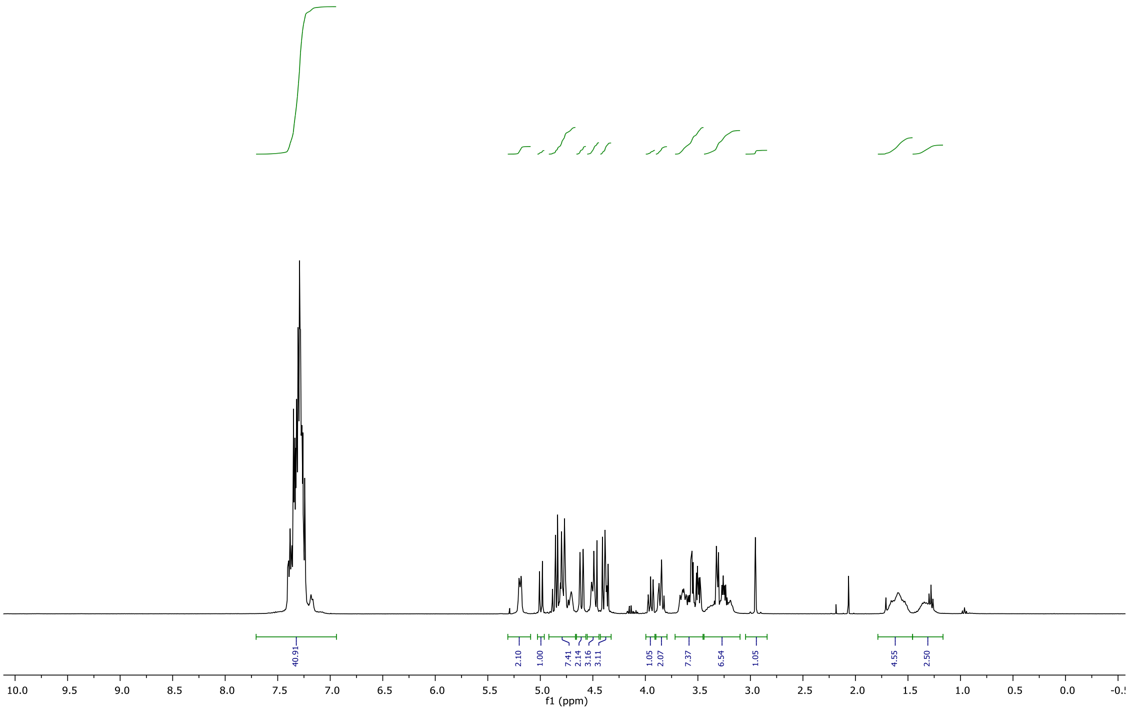

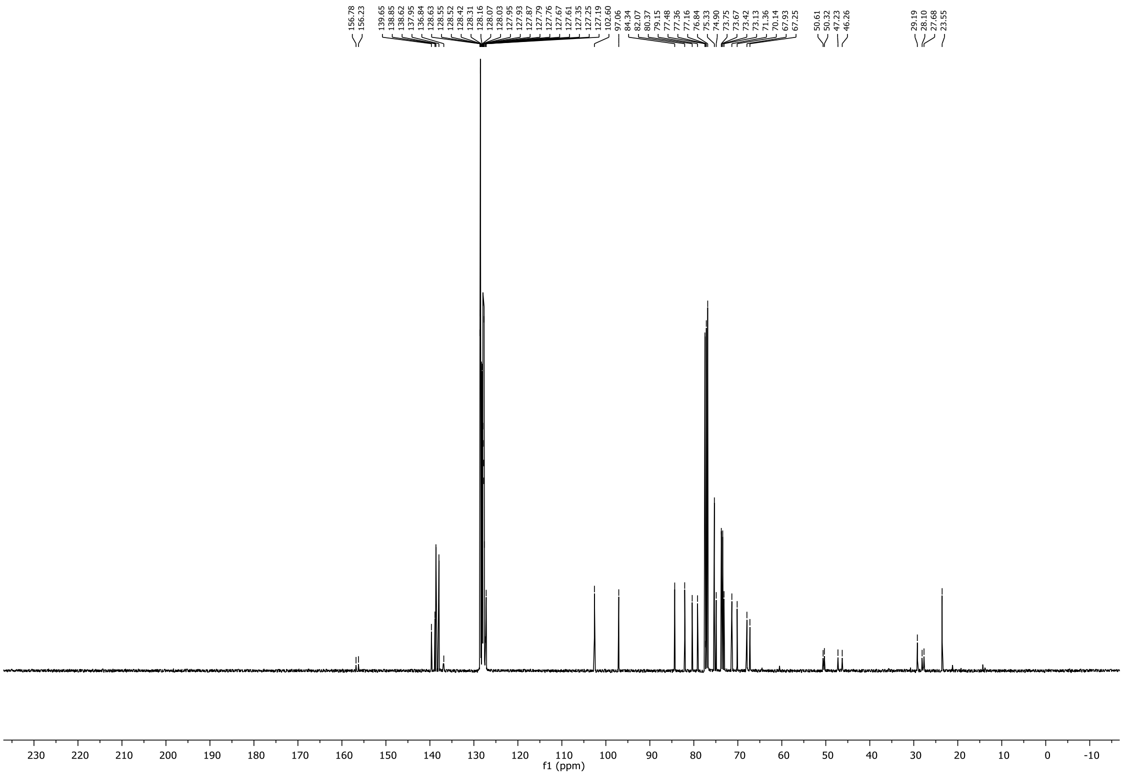

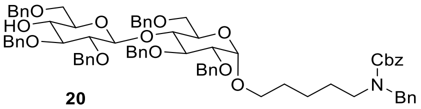
BnO
HO
BnO
BnO
O
BnO
O
BnO
O
BnO
O
Cbz
N
Bn

20

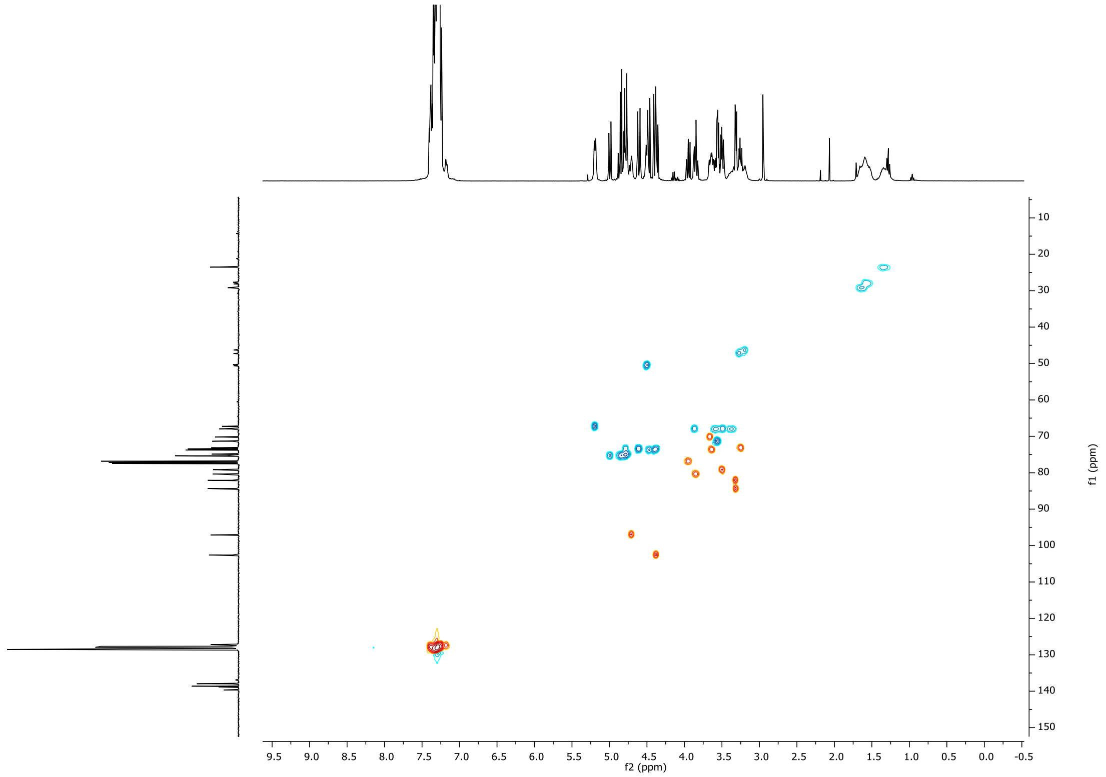
f2 (ppm)
f1 (ppm)

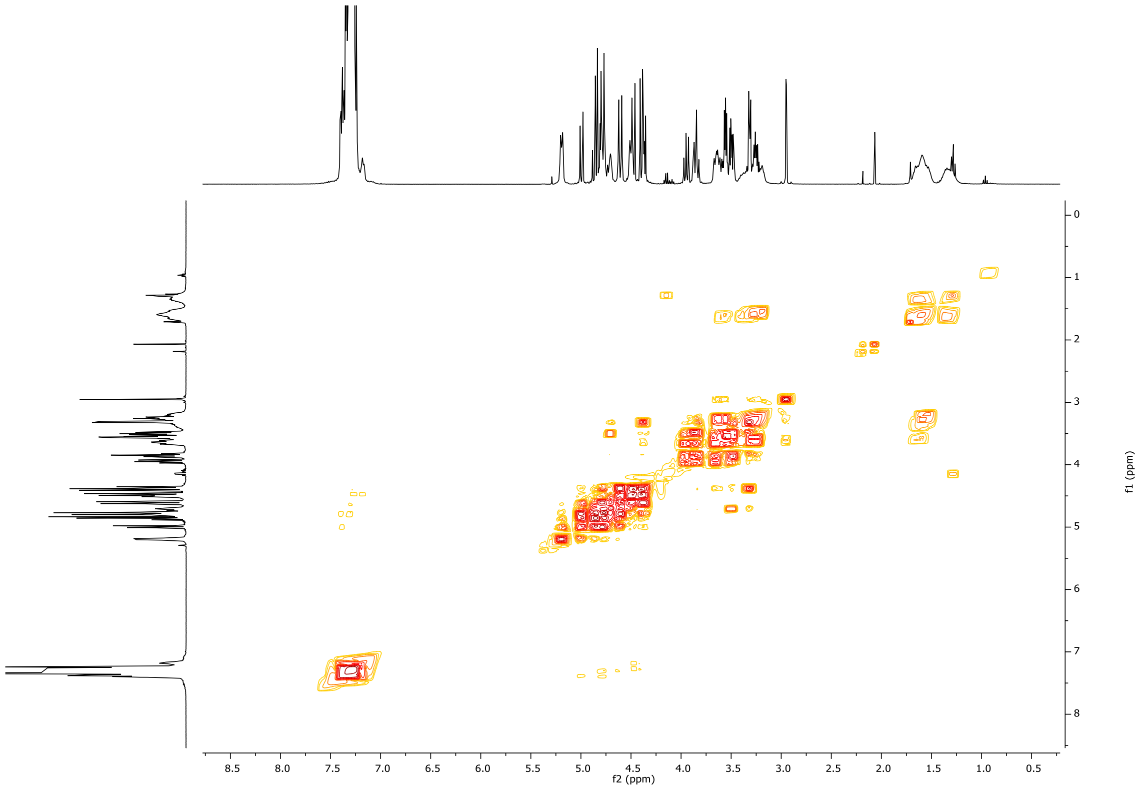
f2 (ppm)
f1 (ppm)

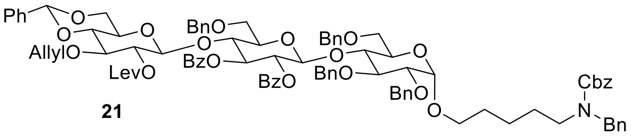

21

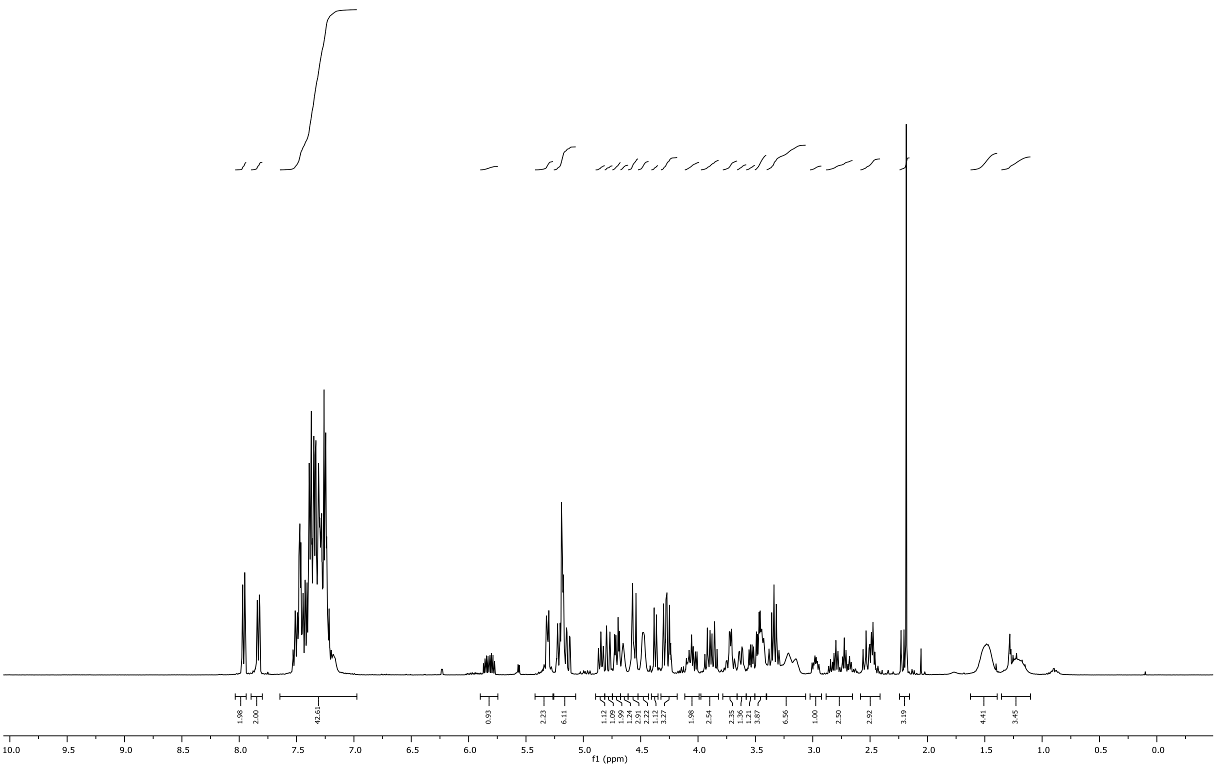

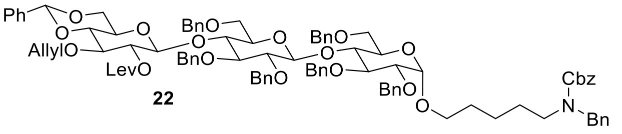
Ph
O
O
O
AllylO
LevO
BnO
O
BnO
O
BnO
BnO
O
BnO
BnO
O
BnO
O
Cbz
N
Bn

22

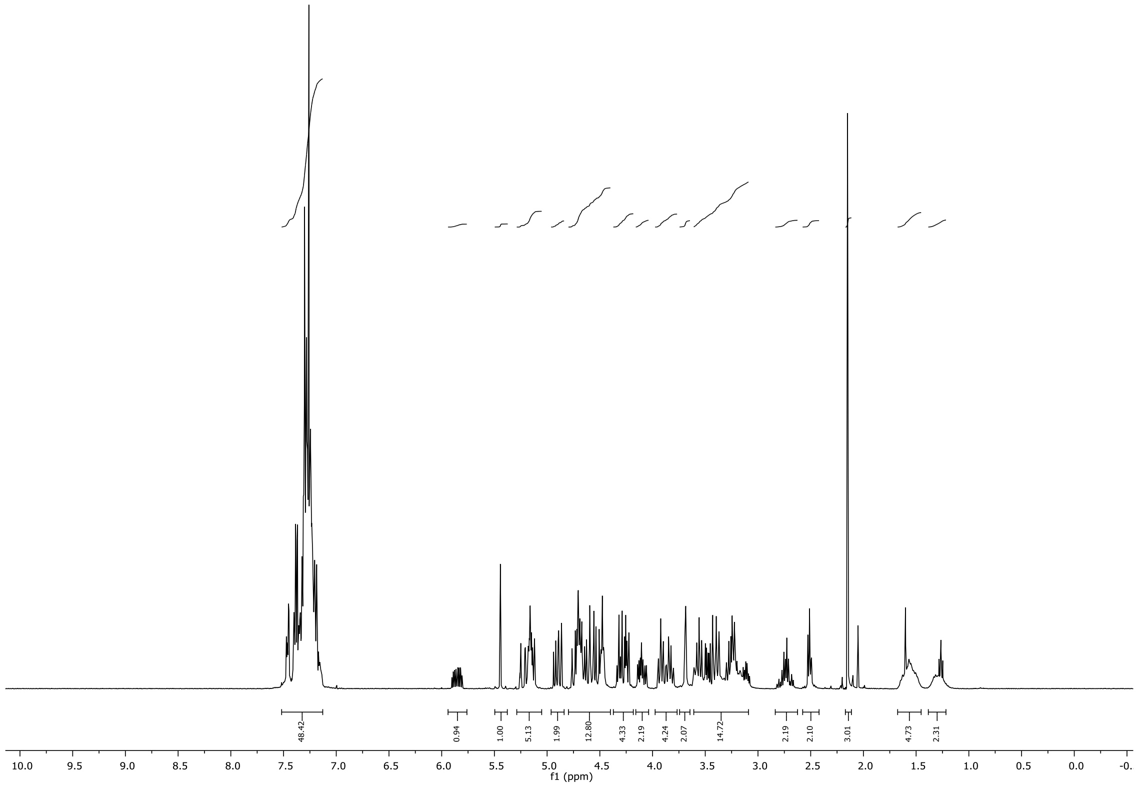
48.42
0.94
1.00
5.13
1.99
12.80
4.33
2.19
4.24
2.07
14.72
2.19
2.10
3.01
4.73
2.31
10.0 9.5 9.0 8.5 8.0 7.5 7.0 6.5 6.0 5.5 5.0 4.5 4.0 3.5 3.0 2.5 2.0 1.5 1.0 0.5 0.0
f1 (ppm)

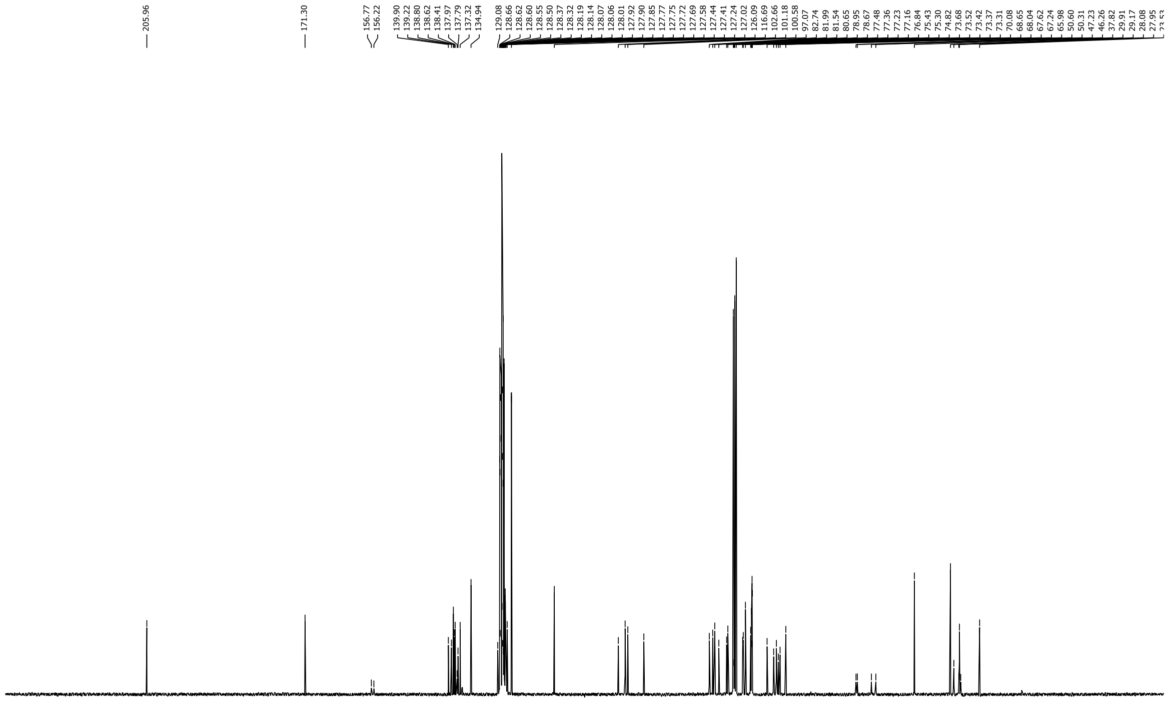
205.96
171.30
156.77
156.22
139.90
139.22
138.80
138.62
138.41
137.97
137.79
137.32
134.94
129.08
128.66
128.62
128.60
128.55
128.50
128.37
128.32
128.19
128.14
128.07
128.06
128.01
127.92
127.90
127.85
127.77
127.75
127.72
127.69
127.58
127.44
127.41
127.24
127.02
126.09
116.69
102.66
101.18
100.58
97.07
82.74
81.99
81.54
80.65
78.95
78.67
77.48
77.36
77.23
77.16
76.84
75.43
75.30
74.82
73.68
73.52
73.42
73.37
73.31
70.08
68.65
68.04
67.62
67.24
65.98
50.60
50.31
47.23
46.26
37.82
29.91
29.17
28.08
27.95
230 220 210 200 190 180 170 160 150 140 130 120 110 100 90 80 70 60 50 40 30 20 10 0 -10
f1 (ppm)

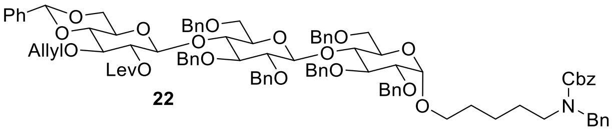
Ph
O
O
AllylO
LevO
O
BnO
O
BnO
BnO
O
BnO
O
BnO
BnO
O
O
Cbz
N
Bn

22

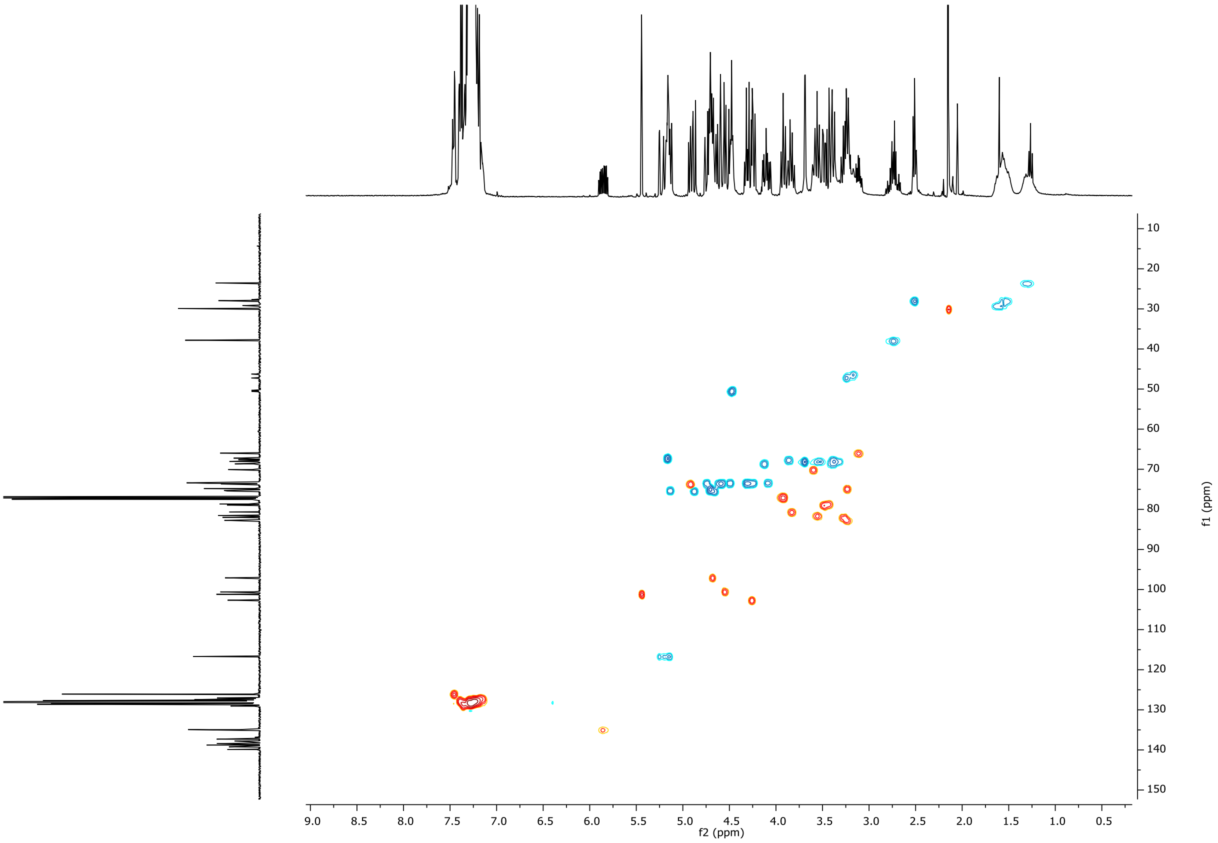
f2 (ppm)
f1 (ppm)

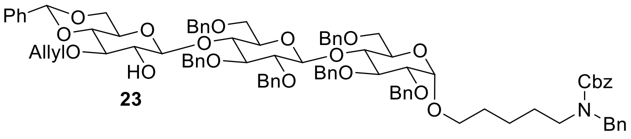

23

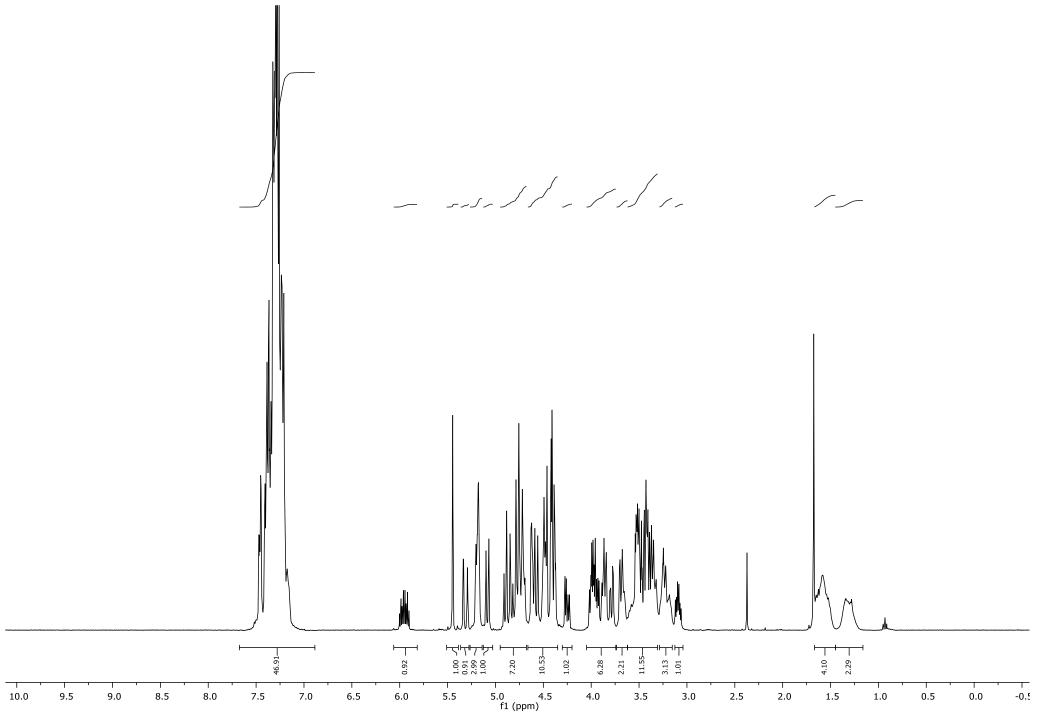

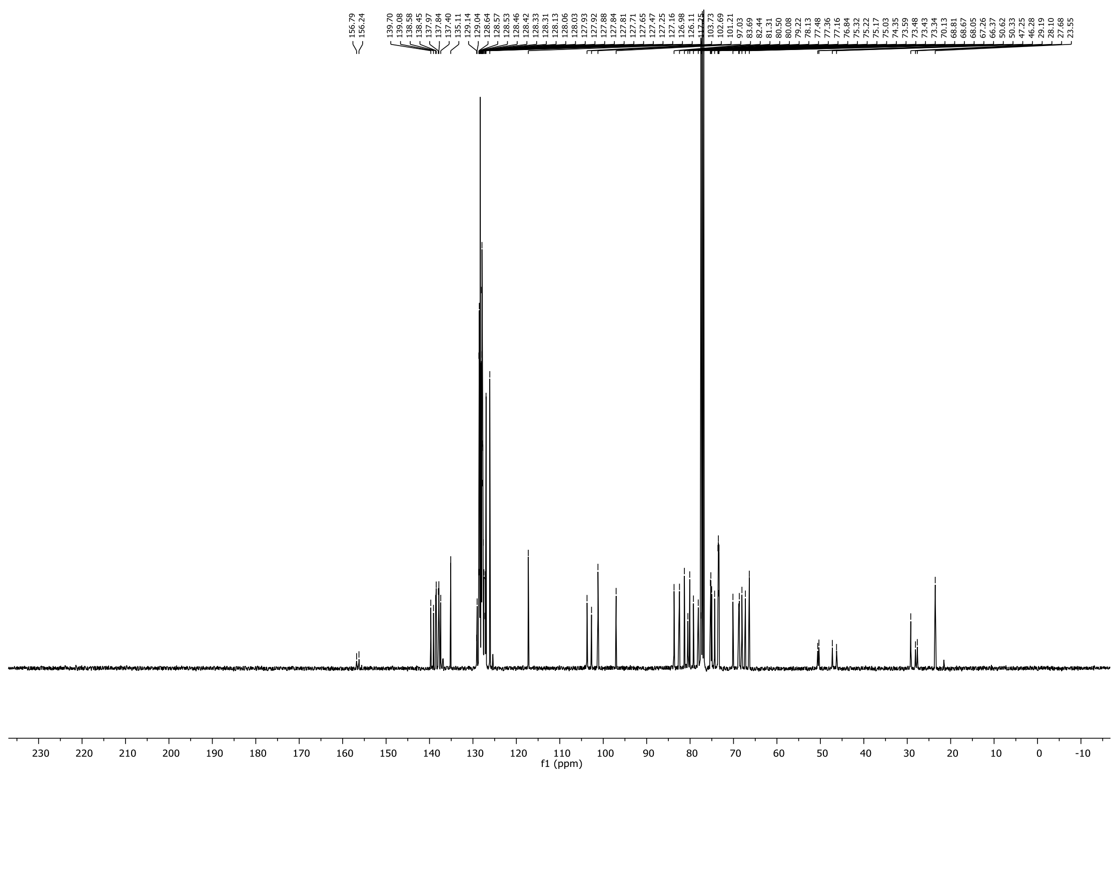

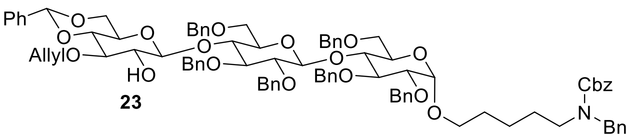
Ph
AllylO
HO
BnO
BnO
BnO
BnO
BnO
BnO
Cbz
N
Bn

23

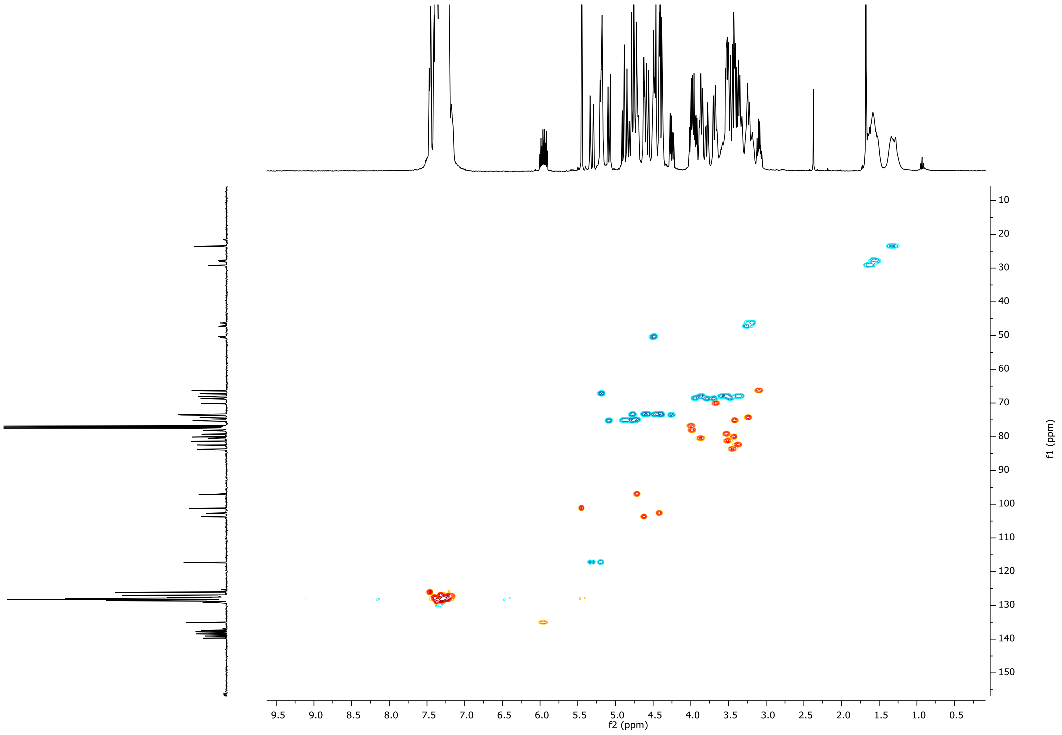
f2 (ppm)
f1 (ppm)

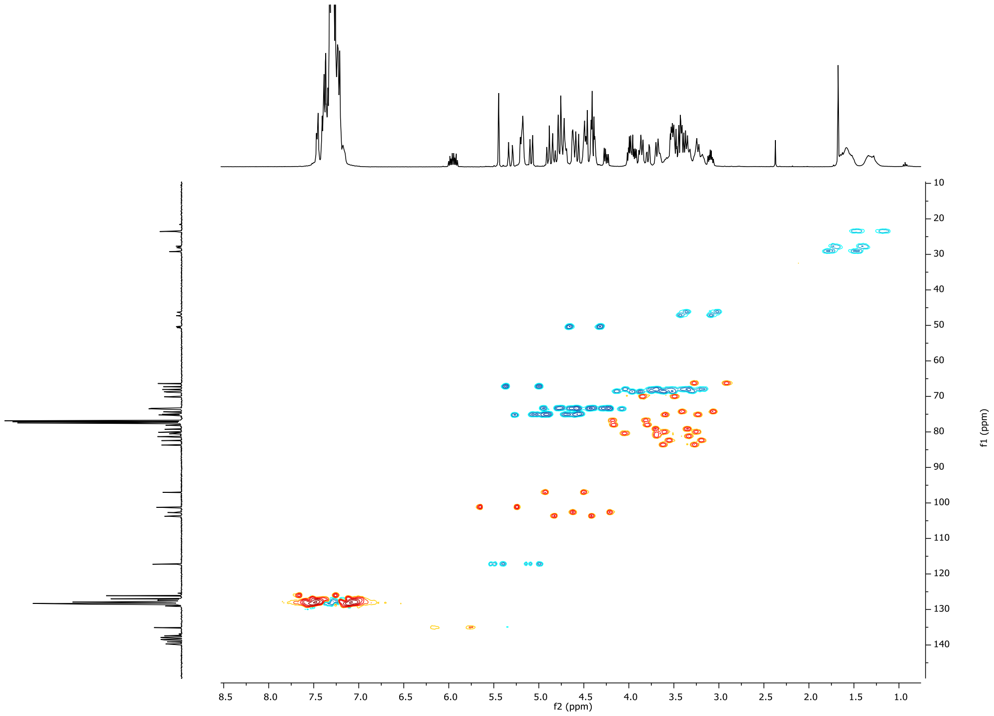
f2 (ppm)
f1 (ppm)

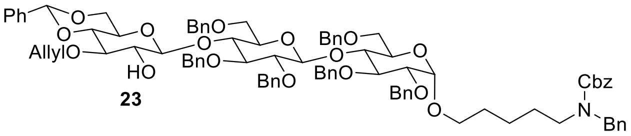

23

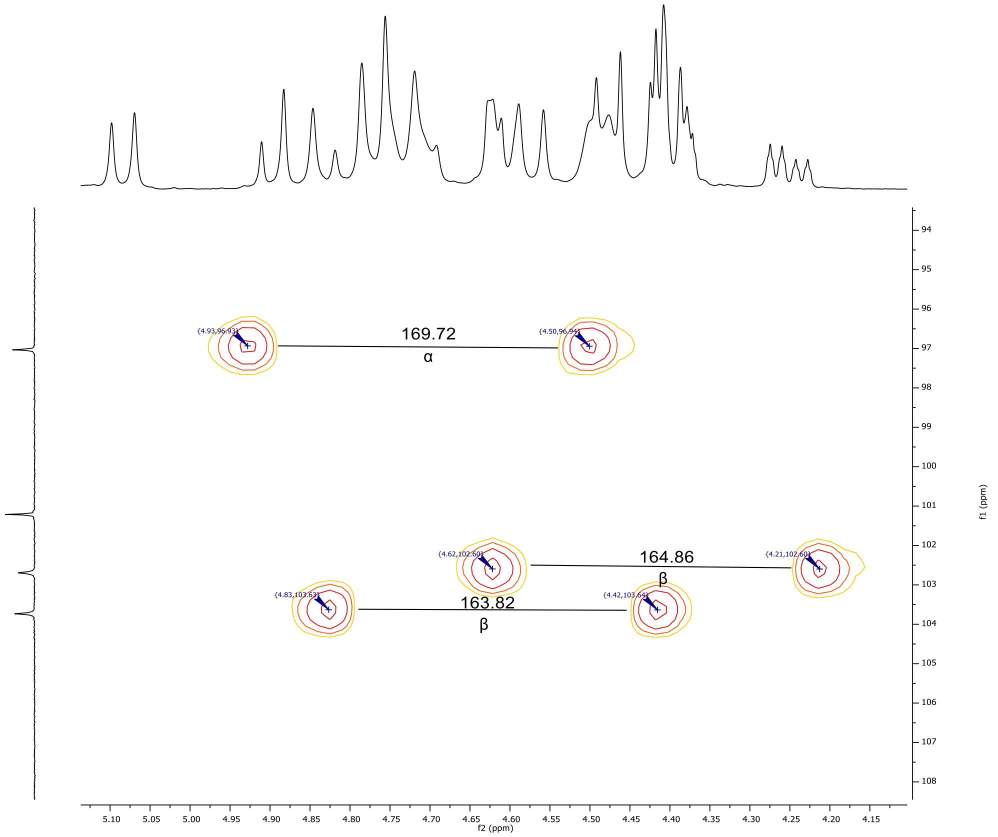

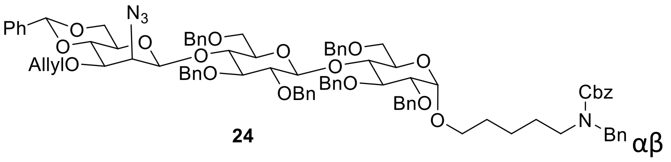

24

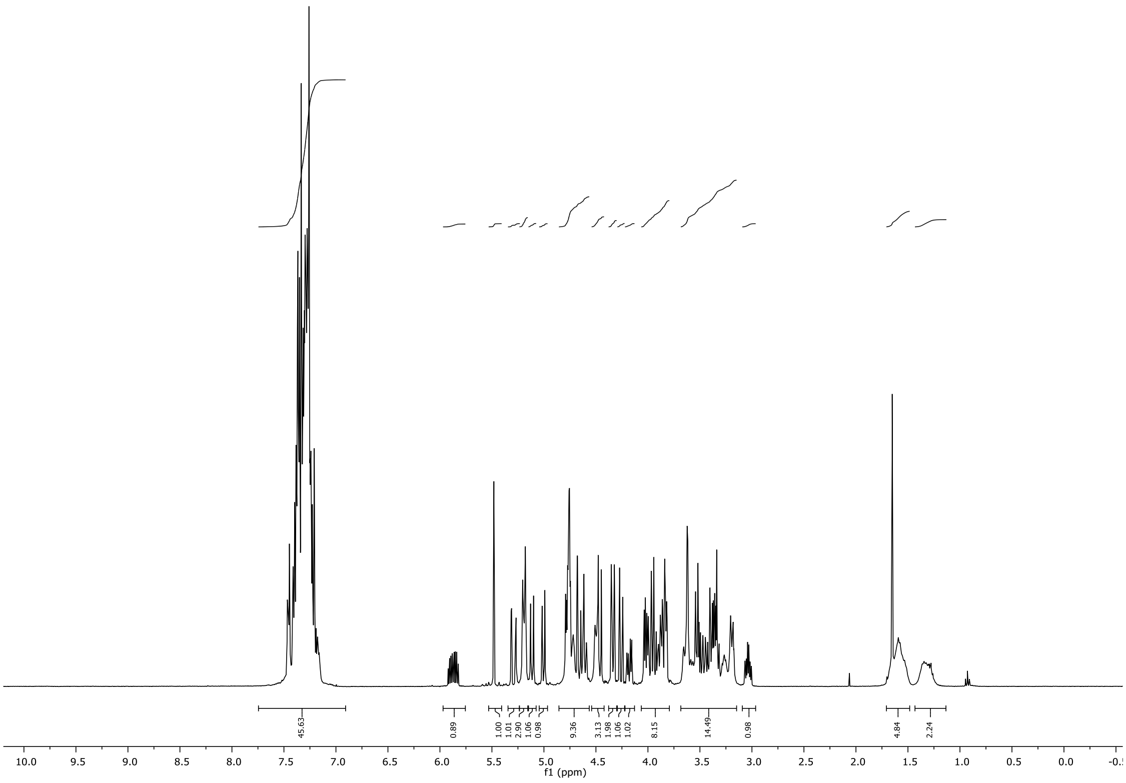

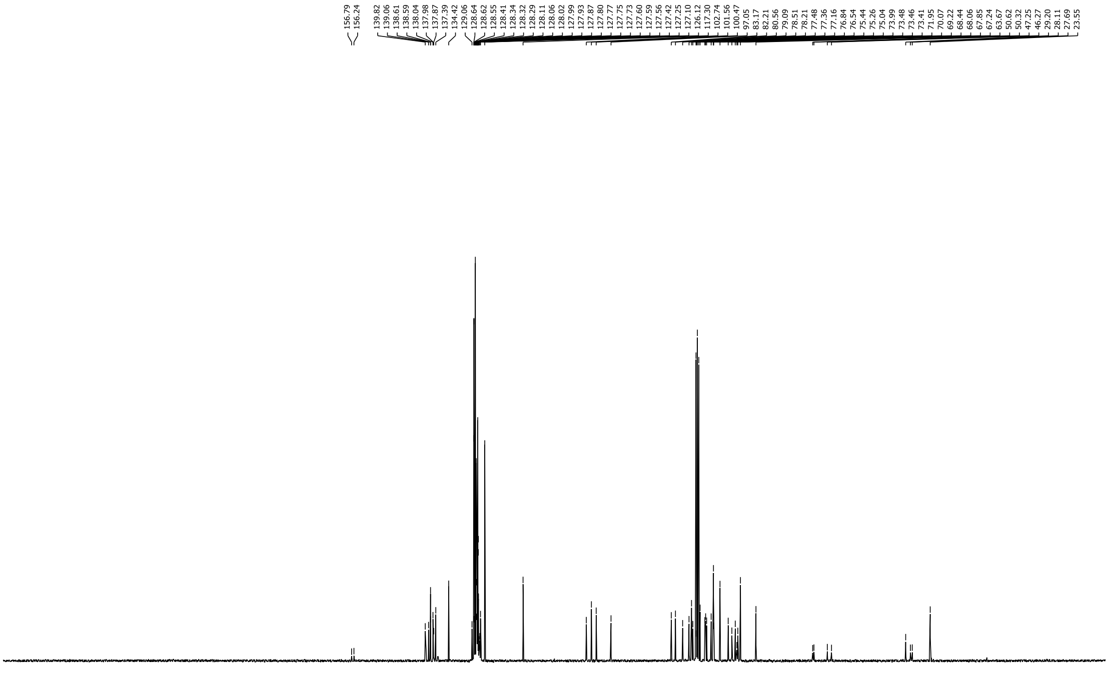

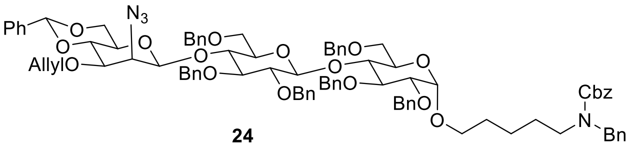
Ph
O
O
N3
O
BnO
O
AllylO
BnO
O
OBn
BnO
O
BnO
O
BnO
O
Cbz
N
Bn

24

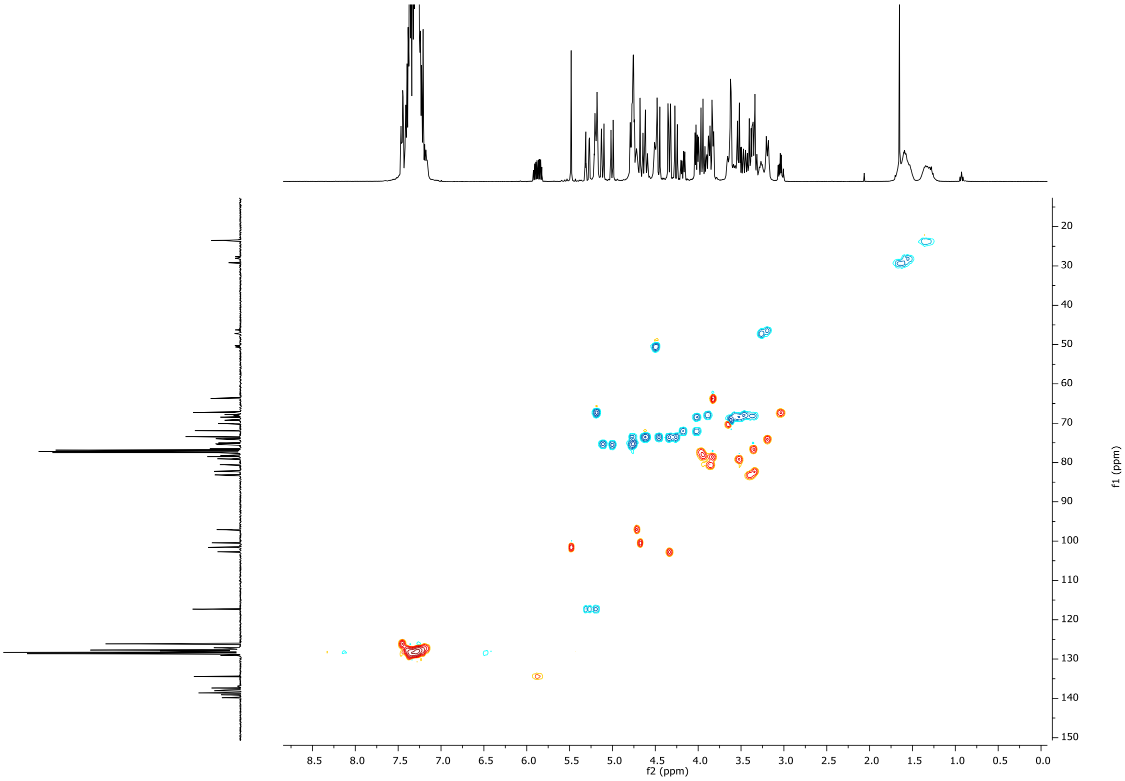
f2 (ppm)
f1 (ppm)

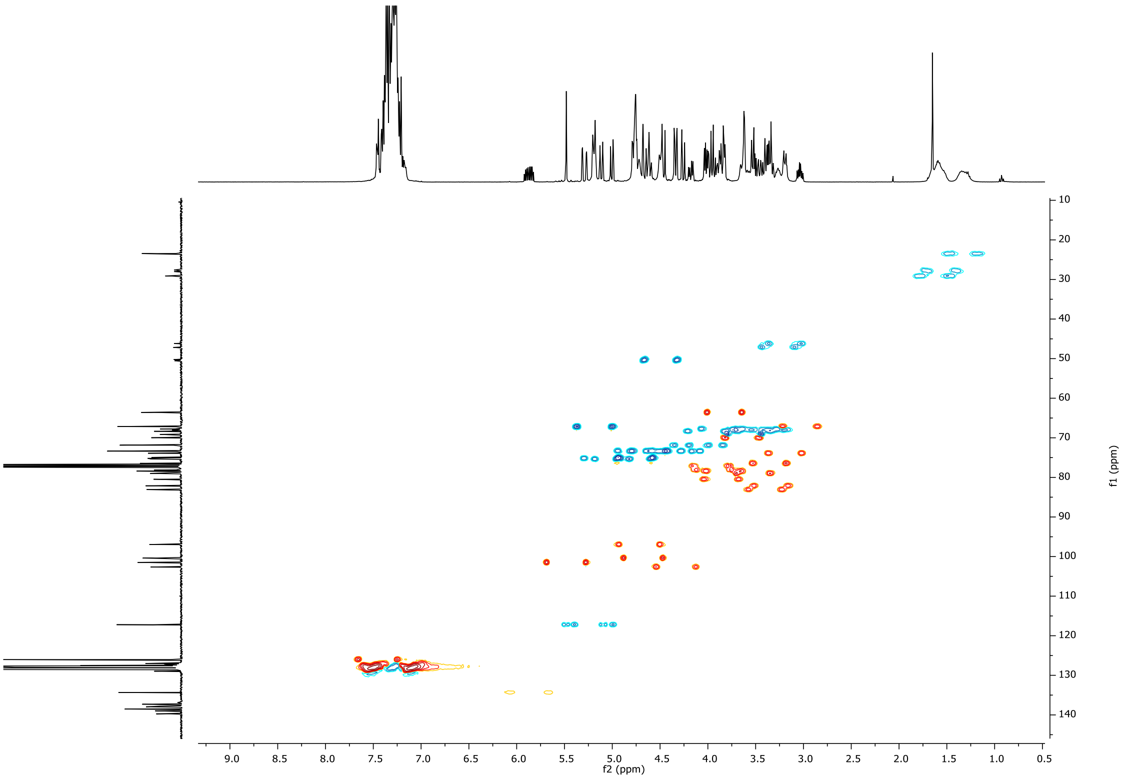
f2 (ppm)
f1 (ppm)

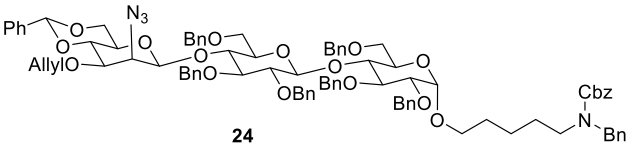
Ph
O
O
N3
O
AllylO
BnO
O
BnO
O
OBn
BnO
O
BnO
O
BnO
O
Cbz
N
Bn

24

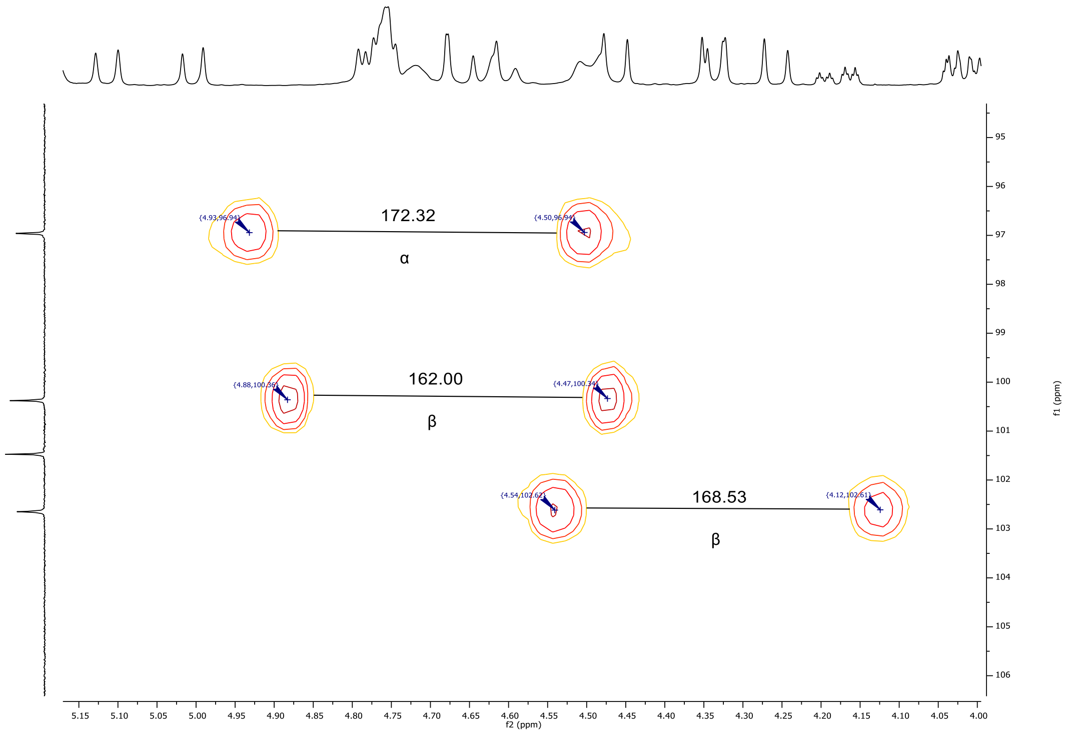
172.32
α
162.00
β
168.53
β
f2 (ppm)
f1 (ppm)

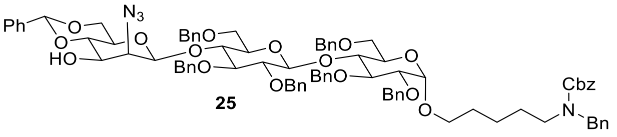
Ph
O
O
HO
N3
O
BnO
O
BnO
O
OBn
BnO
O
BnO
O
BnO
O
Cbz
N
Bn

25

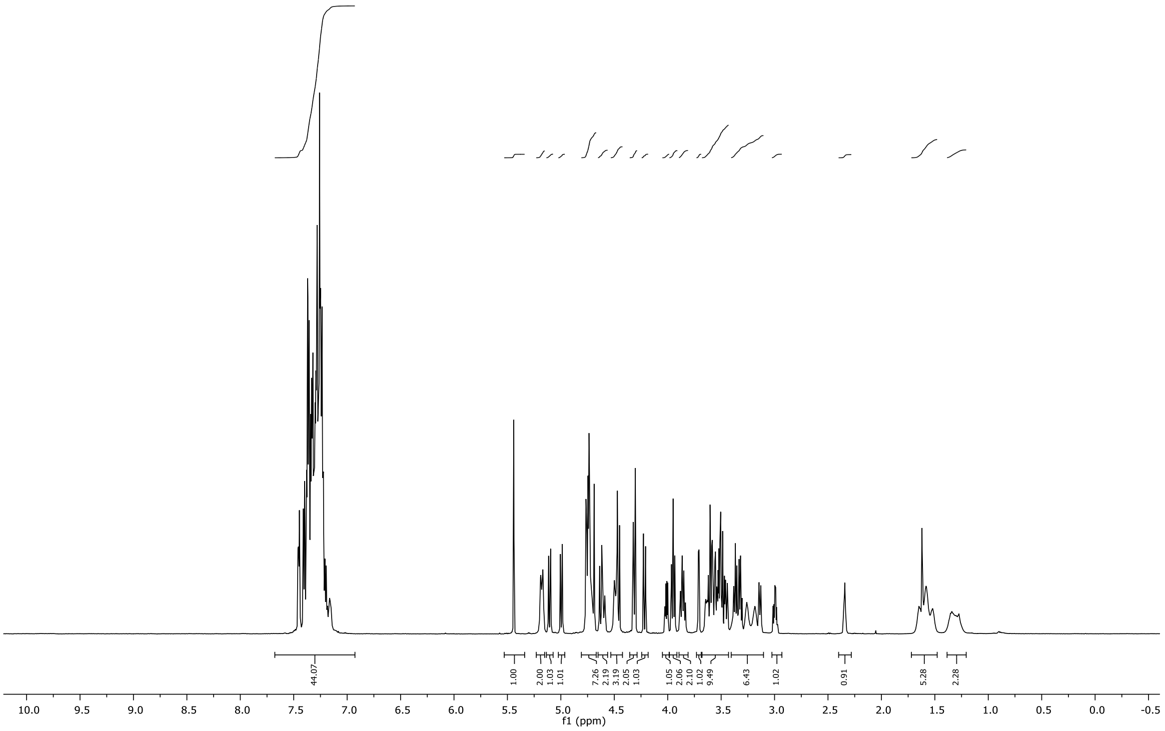
44.07
1.00
2.00
1.03
1.01
7.26
2.19
3.19
2.05
1.03
1.05
2.06
2.10
1.02
9.49
6.43
1.02
0.91
5.28
2.28
10.0 9.5 9.0 8.5 8.0 7.5 7.0 6.5 6.0 5.5 5.0 4.5 4.0 3.5 3.0 2.5 2.0 1.5 1.0 0.5 0.0 -0.5
f1 (ppm)

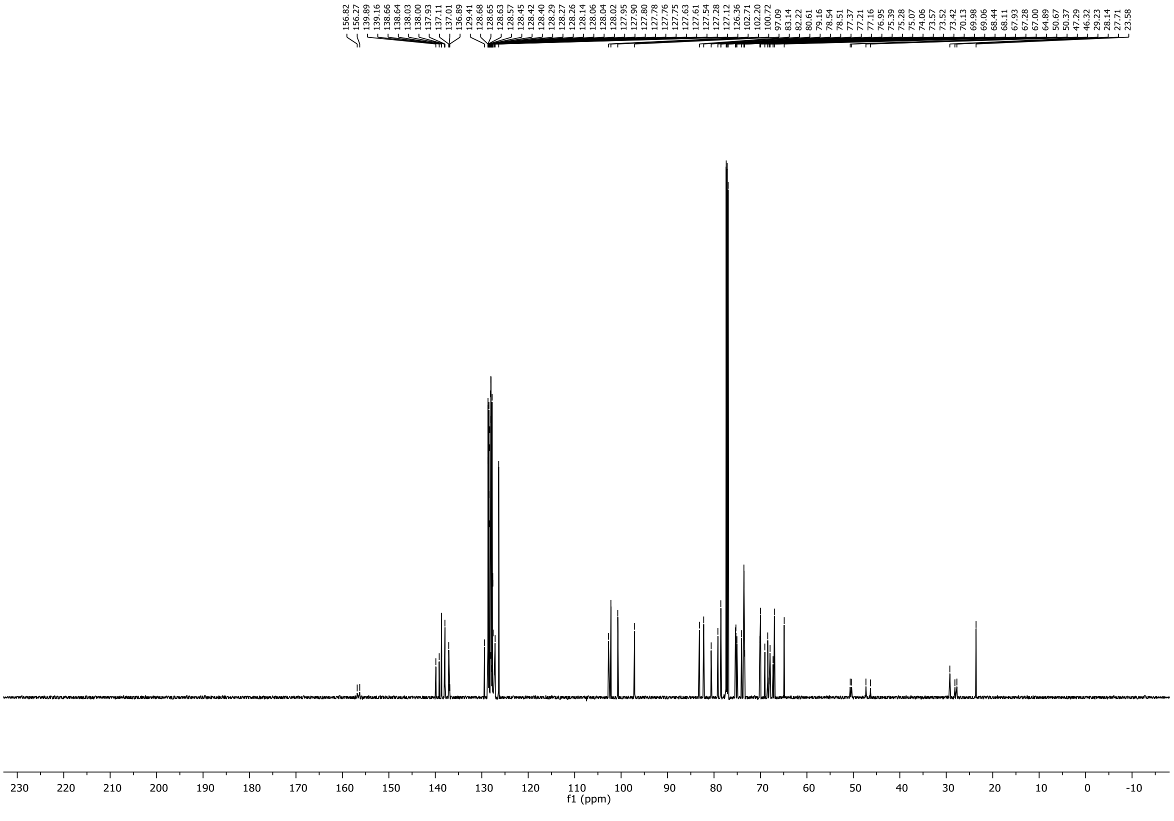
156.82
156.27
139.89
139.16
138.66
138.64
138.03
138.00
137.93
137.11
137.01
136.89
129.41
128.68
128.65
128.63
128.57
128.45
128.42
128.40
128.29
128.27
128.26
128.14
128.06
128.04
128.02
127.95
127.90
127.80
127.78
127.76
127.75
127.63
127.61
127.54
127.28
127.12
126.36
102.71
102.20
100.72
97.09
83.14
82.22
80.61
79.16
78.54
78.51
77.37
77.21
77.16
76.95
75.39
75.28
75.07
74.06
73.57
73.52
73.42
70.13
69.98
69.06
68.44
68.11
67.93
67.28
67.00
64.89
50.67
50.37
47.29
46.32
29.23
28.14
27.71
23.58
230 220 210 200 190 180 170 160 150 140 130 120 110 100 90 80 70 60 50 40 30 20 10 0 -10
f1 (ppm)

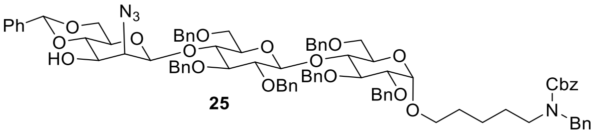

25

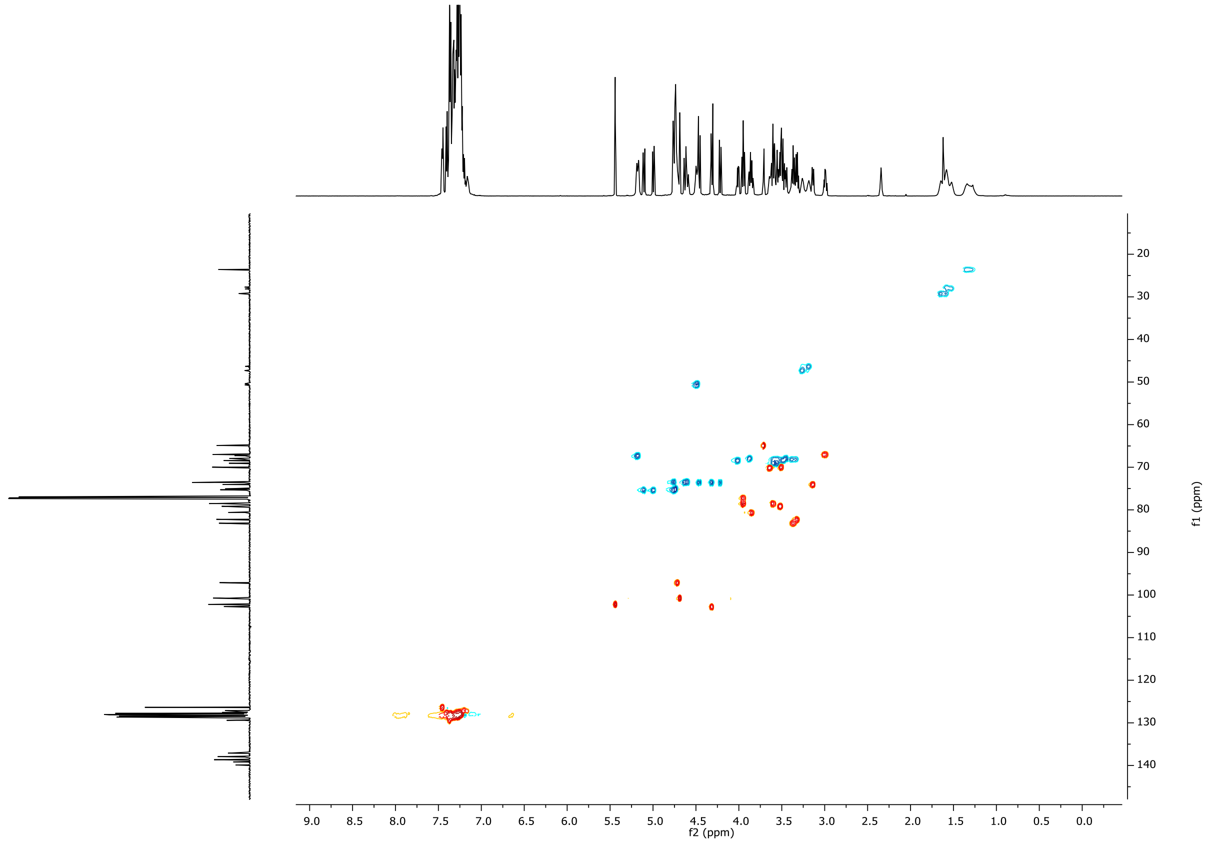

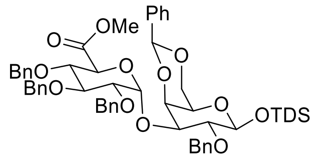
OMe
Ph
BnO
BnO
BnO
OTDS
BnO

26α

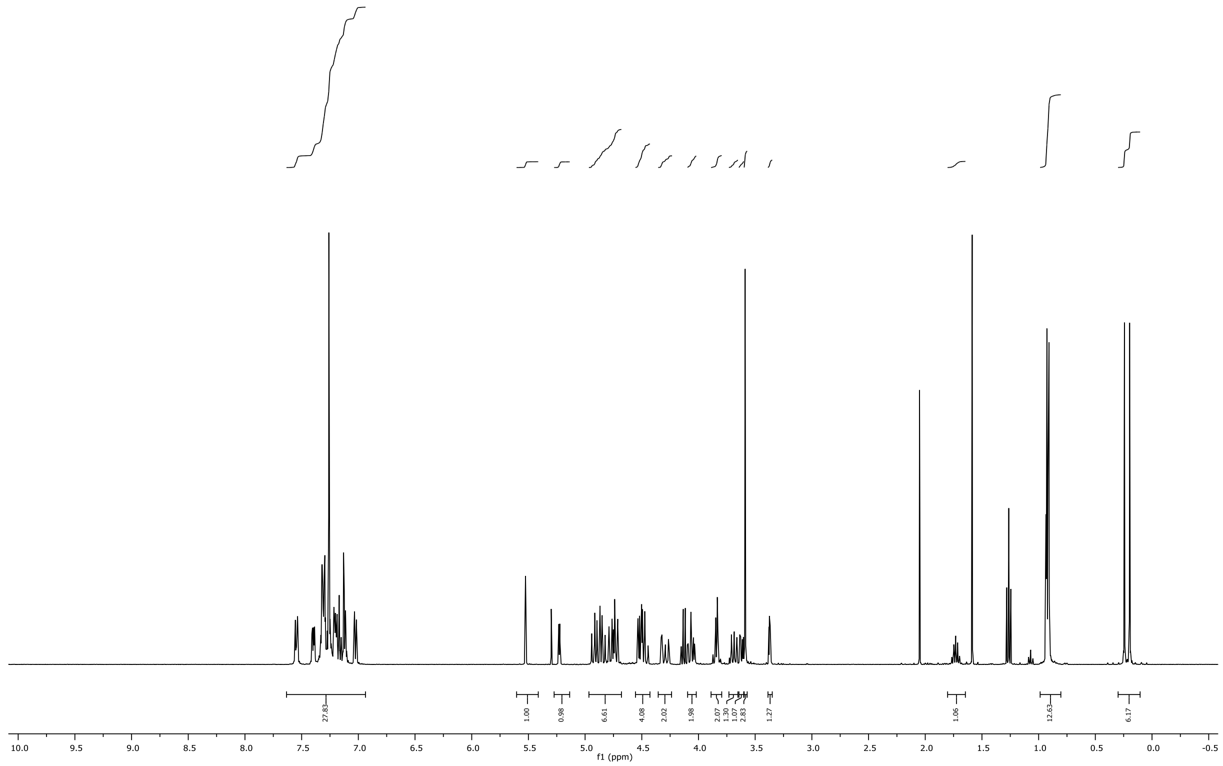
f1 (ppm)

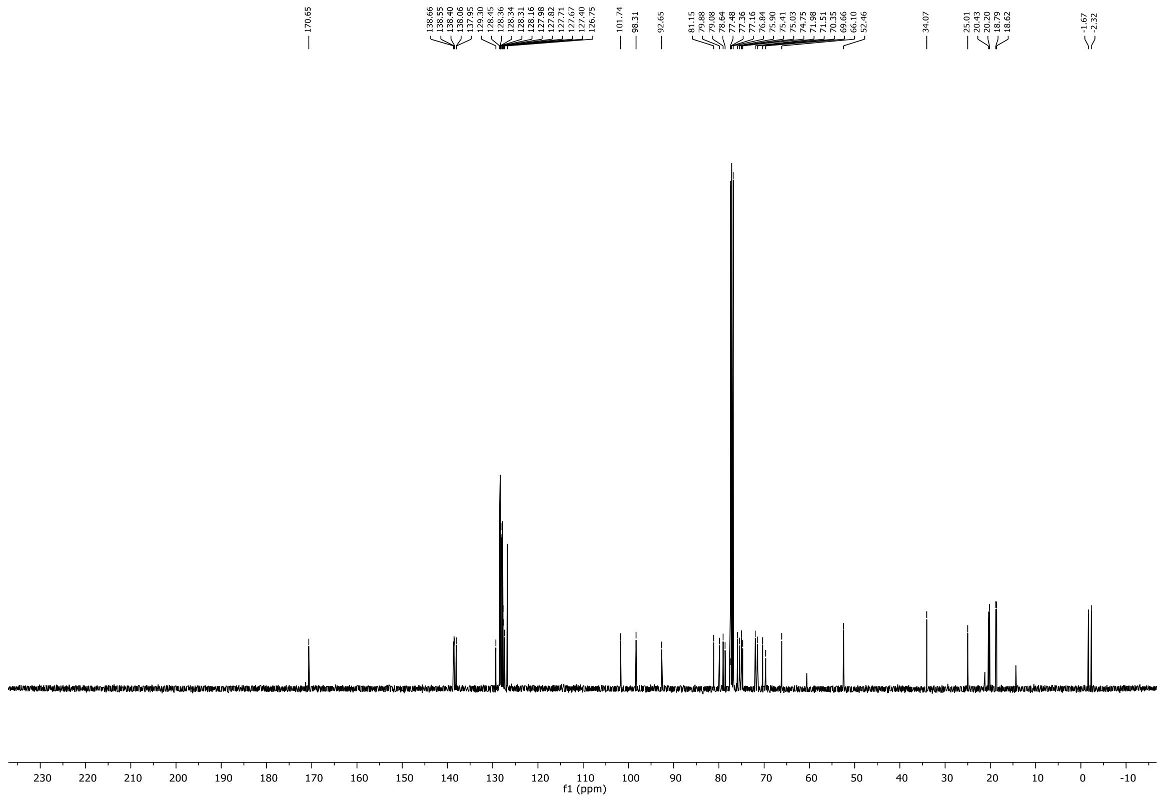
170.65
138.66
138.55
138.40
138.06
137.95
129.30
128.45
128.36
128.34
128.31
128.16
127.98
127.82
127.71
127.67
127.40
126.75
101.74
98.31
92.65
81.15
79.88
79.08
78.64
77.48
77.36
77.16
76.84
75.90
75.41
75.03
74.75
71.98
71.51
70.35
69.66
66.10
52.46
34.07
25.01
20.43
20.20
18.79
18.62
-1.67
-2.32
f1 (ppm)

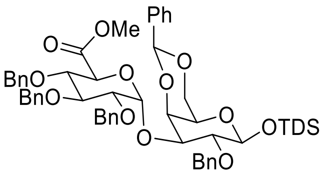

26α

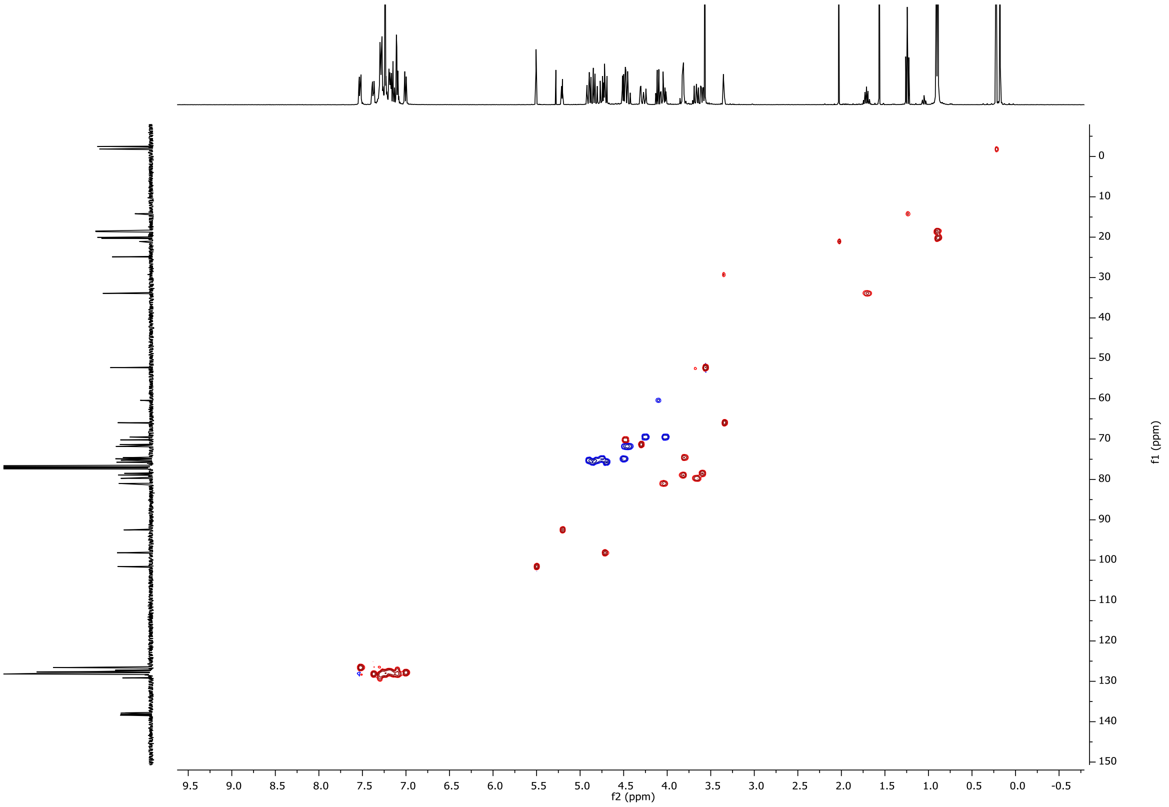

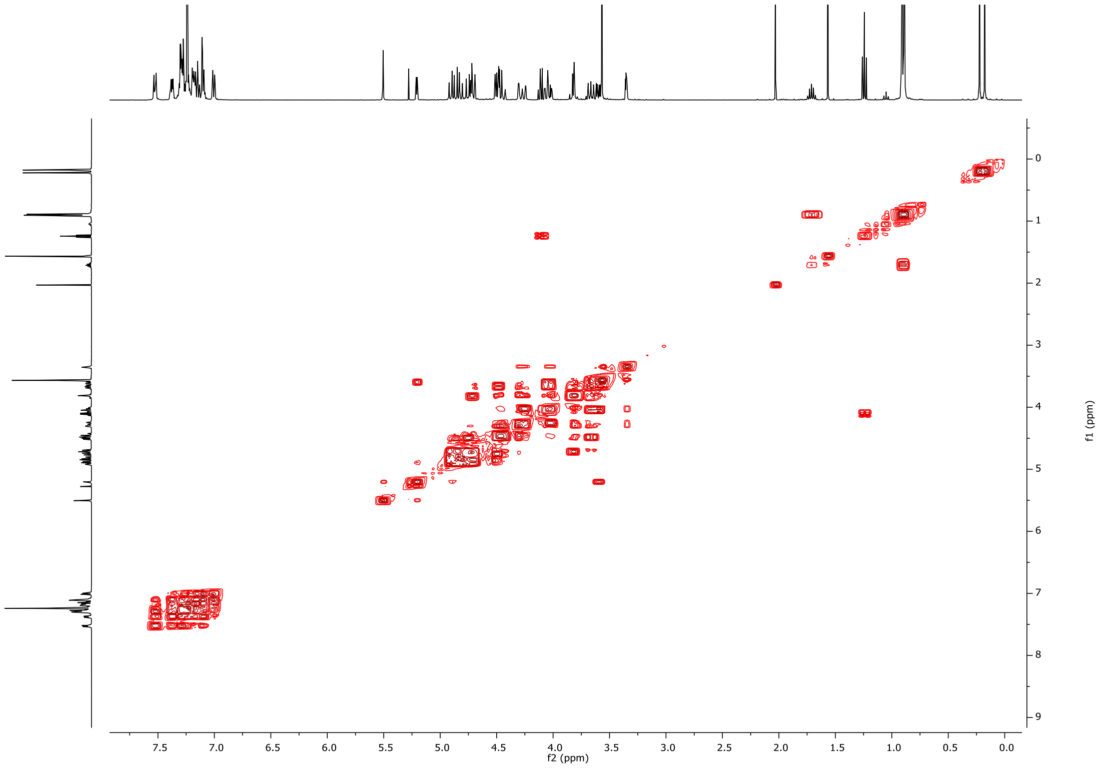

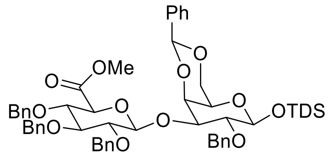

26β

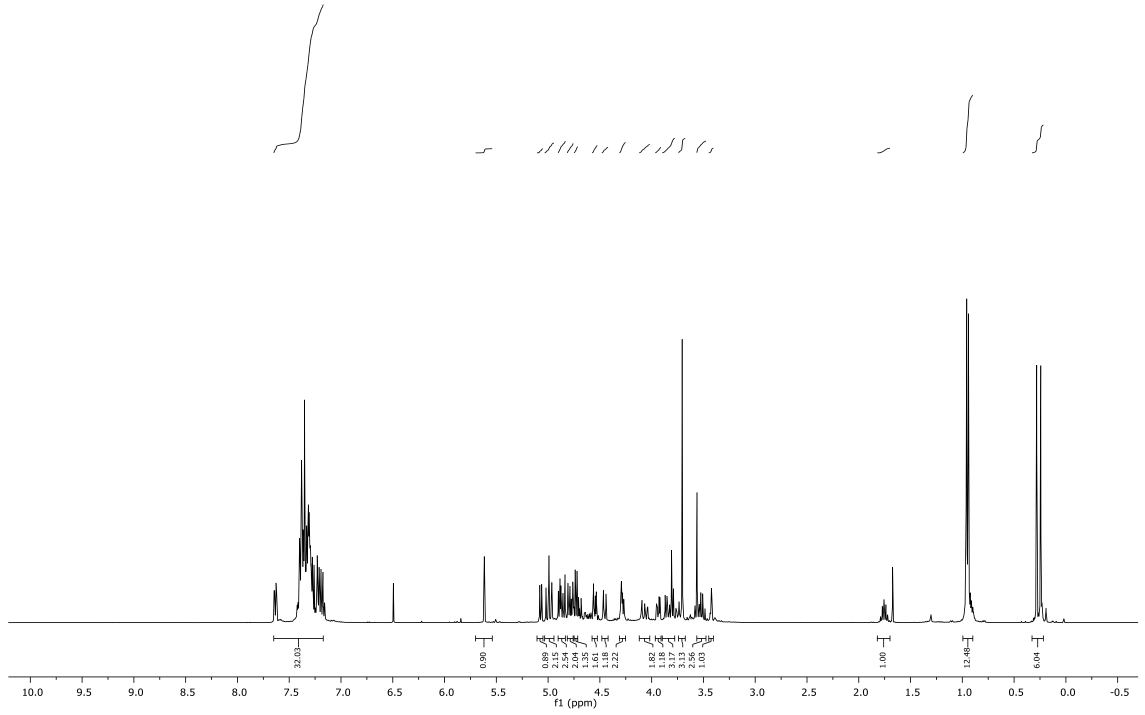

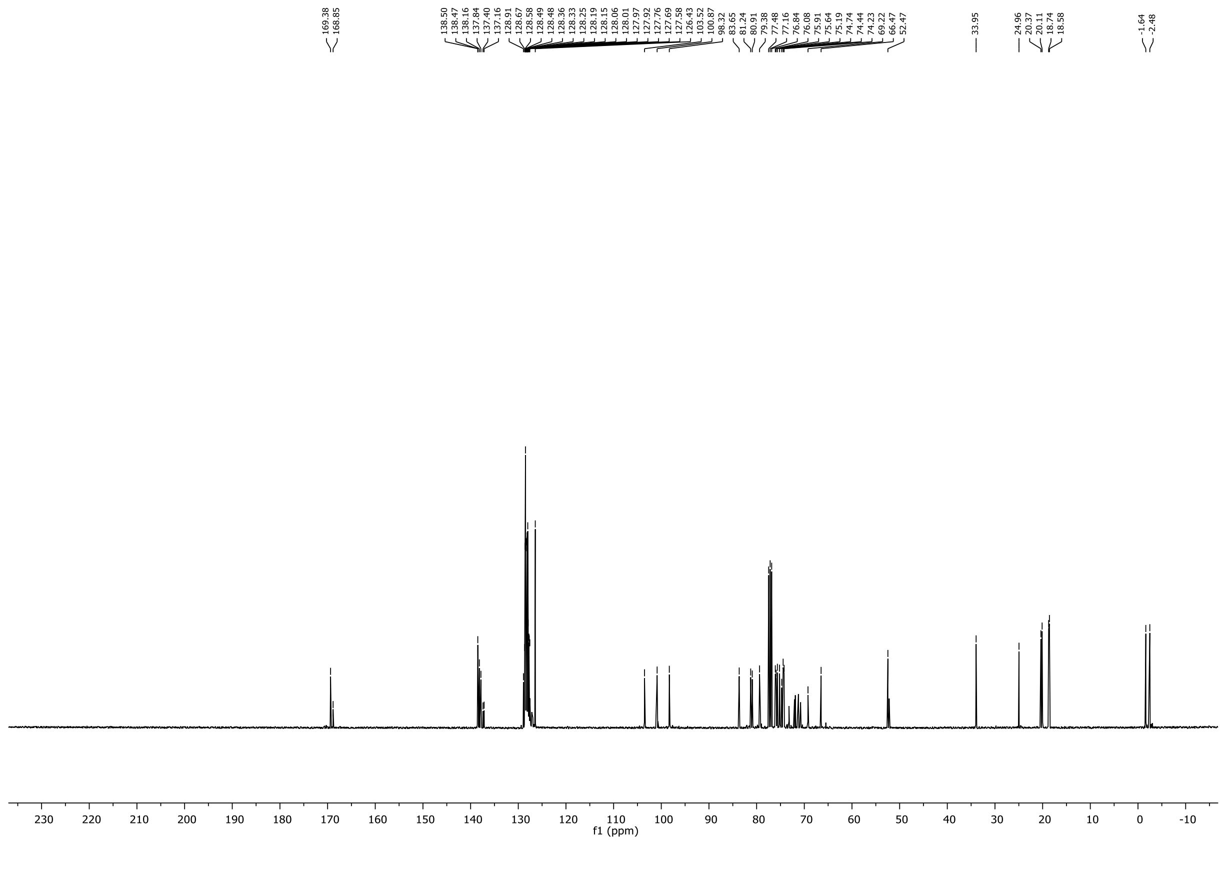

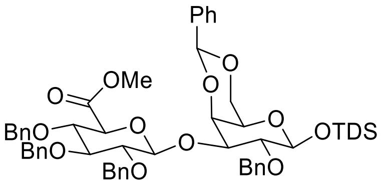

26β

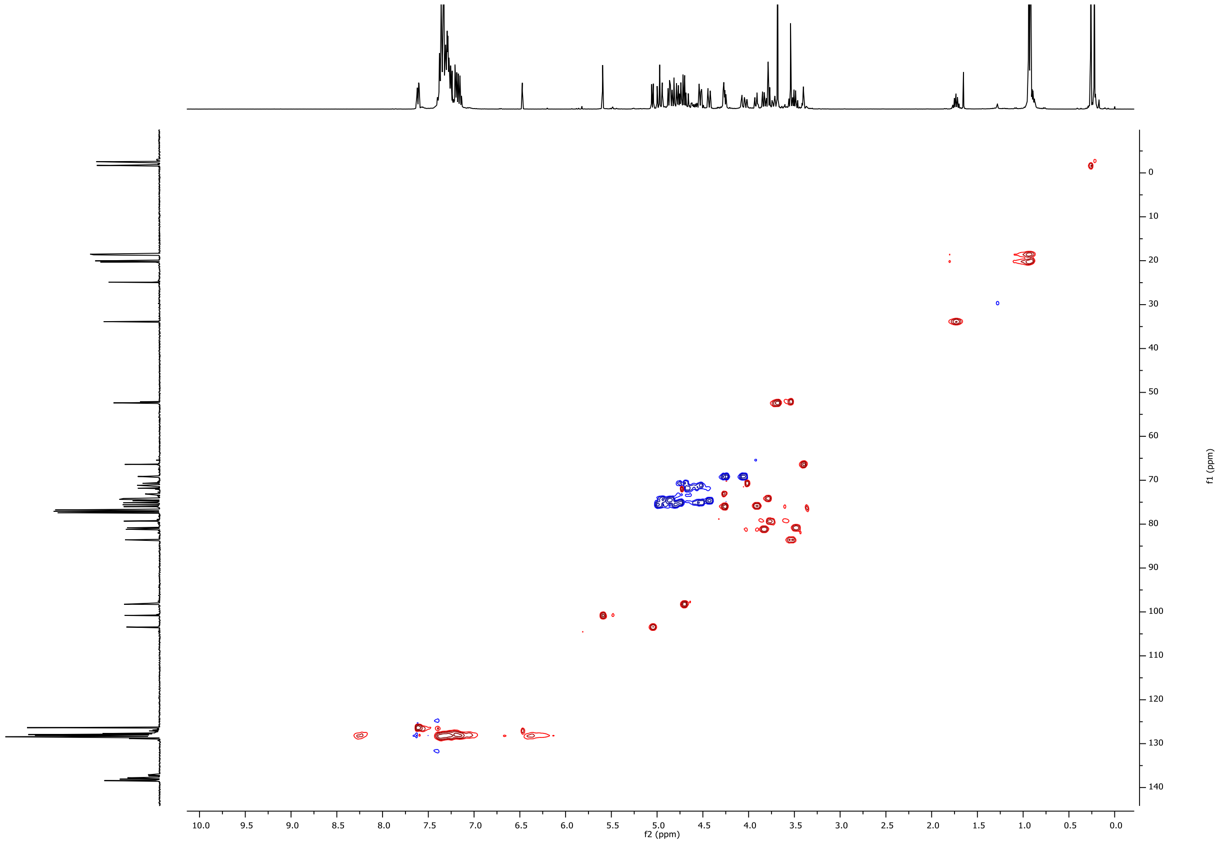

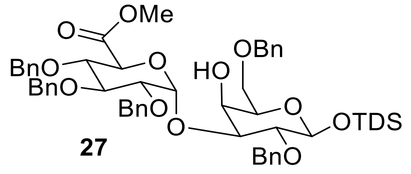
OMe
O
BnO
BnO
BnO
O
HO
OBn
O
OTDS
BnO

27

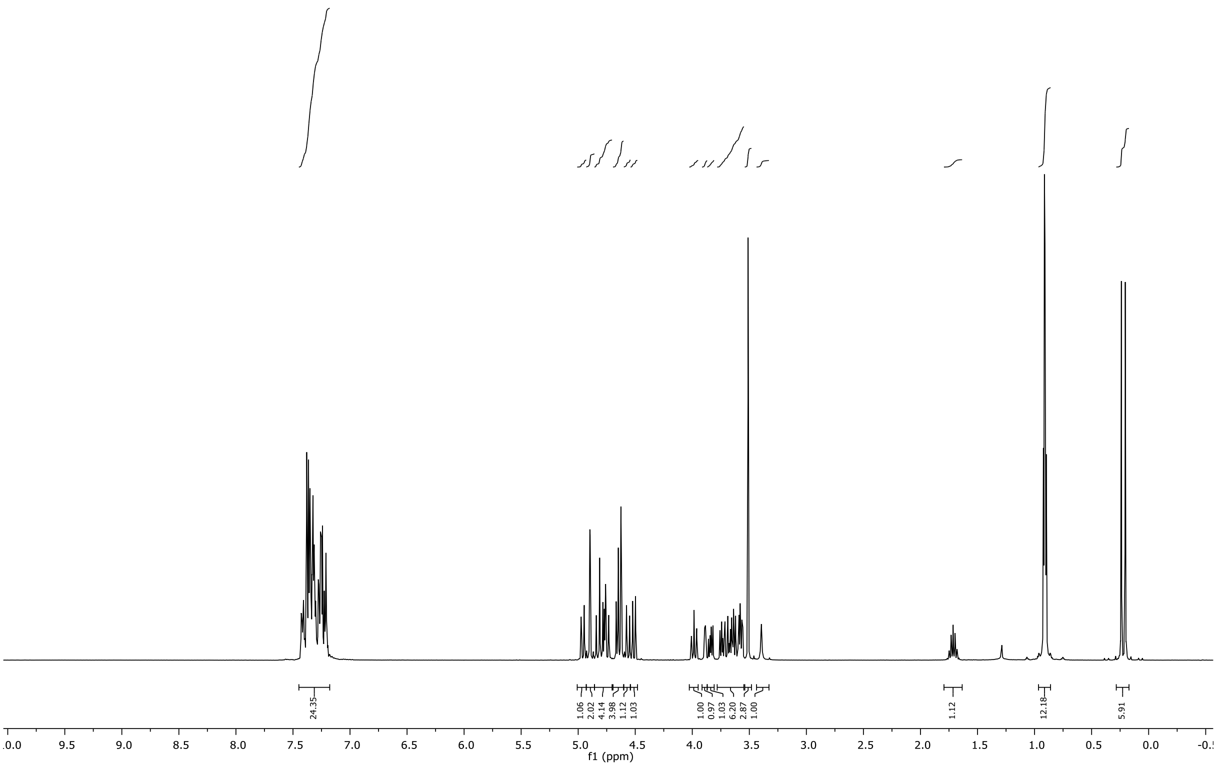
24.35
1.06
2.02
4.14
3.98
1.12
1.03
1.00
0.97
1.03
6.20
2.87
1.00
1.12
12.18
5.91
f1 (ppm)

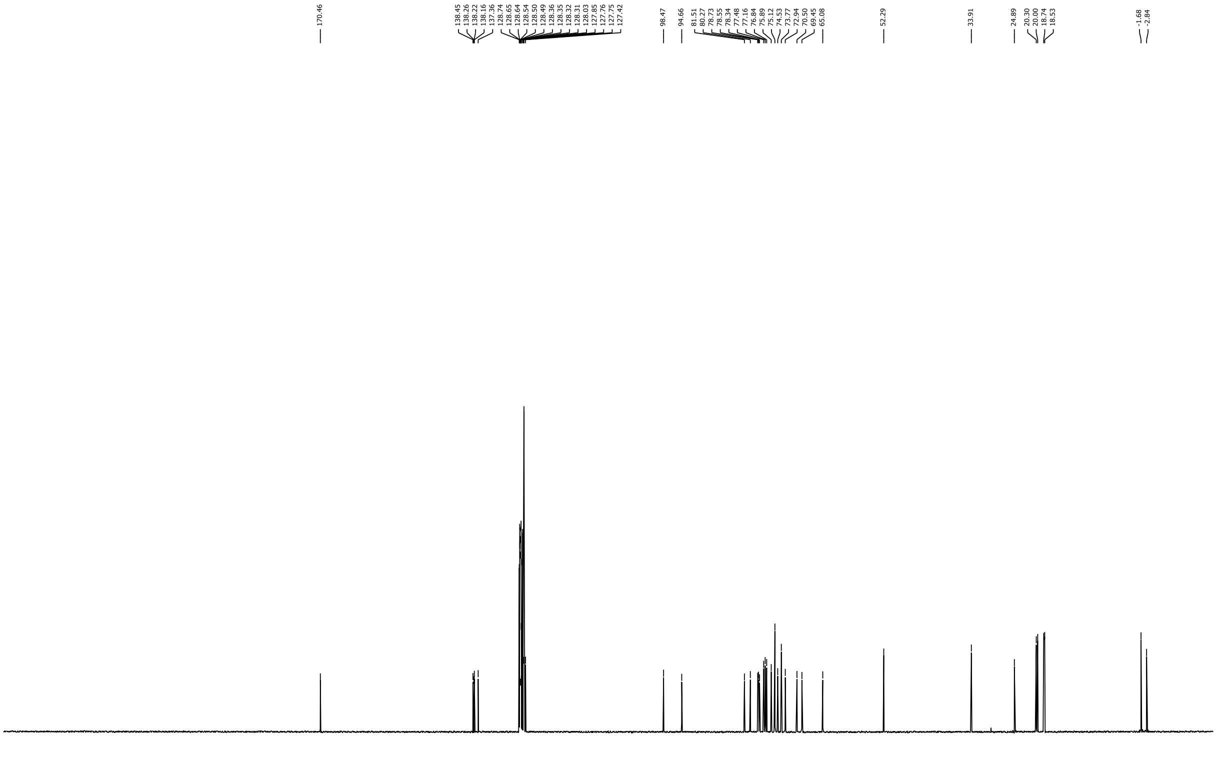
170.46
138.45
138.26
138.22
138.16
137.36
128.74
128.65
128.64
128.54
128.50
128.49
128.36
128.35
128.32
128.31
128.03
127.85
127.76
127.75
127.42
98.47
94.66
81.51
80.27
78.73
78.55
78.34
77.48
77.16
76.84
75.89
75.12
74.53
73.77
72.94
70.50
69.45
65.08
52.29
33.91
24.89
20.30
20.00
18.74
18.53
-1.68
-2.84
f1 (ppm)

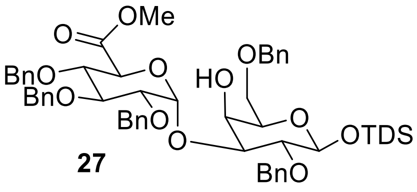

27

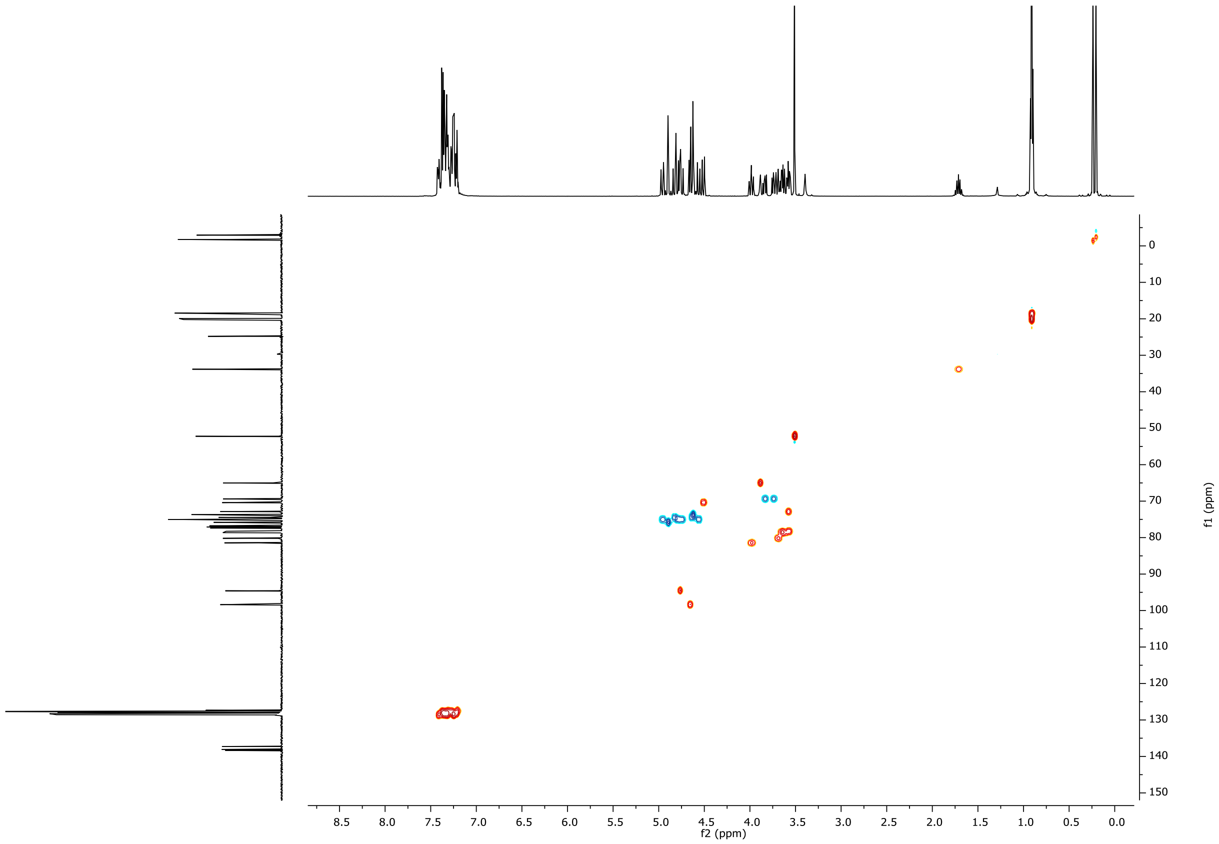

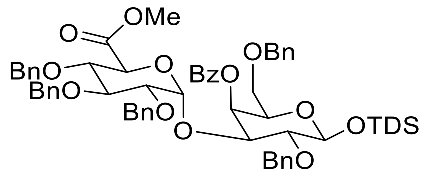

OMe
O
BnO
BnO
BnO
O
BzO
OBn
O
OTDS
BnO

6

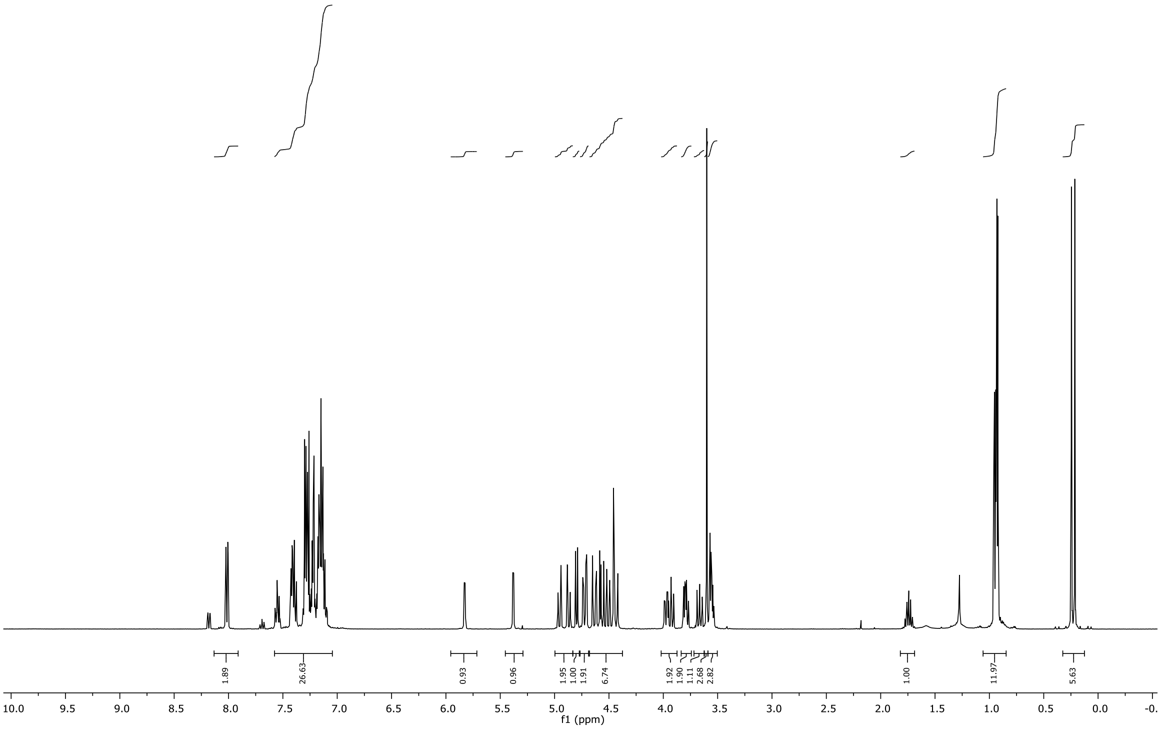

1.89
26.63
0.93
0.96
1.95
1.00
1.91
6.74
1.92
1.90
1.11
2.68
2.82
1.00
11.97
5.63
10.0 9.5 9.0 8.5 8.0 7.5 7.0 6.5 6.0 5.5 5.0 4.5 4.0 3.5 3.0 2.5 2.0 1.5 1.0 0.5 0.0 -0.
f1 (ppm)

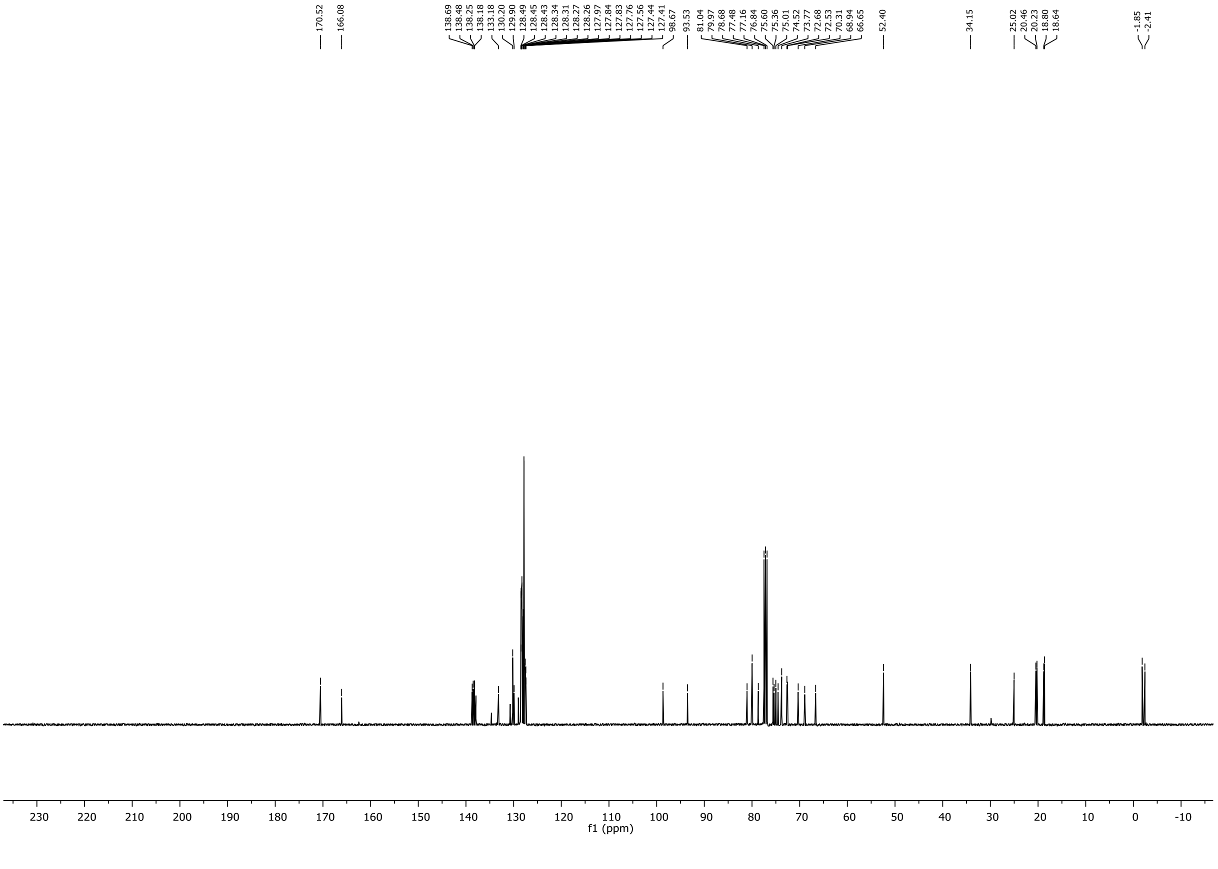

170.52
166.08
138.69
138.48
138.25
138.18
133.18
130.20
129.90
128.49
128.45
128.43
128.34
128.31
128.27
128.26
127.97
127.84
127.83
127.76
127.56
127.44
127.41
98.67
93.53
81.04
79.97
78.68
77.48
77.16
76.84
75.60
75.36
75.01
74.52
73.77
72.68
72.53
70.31
68.94
66.65
52.40
34.15
25.02
20.46
20.23
18.80
18.64
-1.85
-2.41
230 220 210 200 190 180 170 160 150 140 130 120 110 100 90 80 70 60 50 40 30 20 10 0 -10
f1 (ppm)

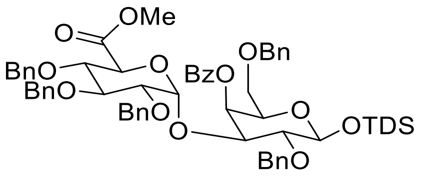
OMe
O
BnO
BnO
BnO
O
BzO
OBn
O
OTDS
BnO

6

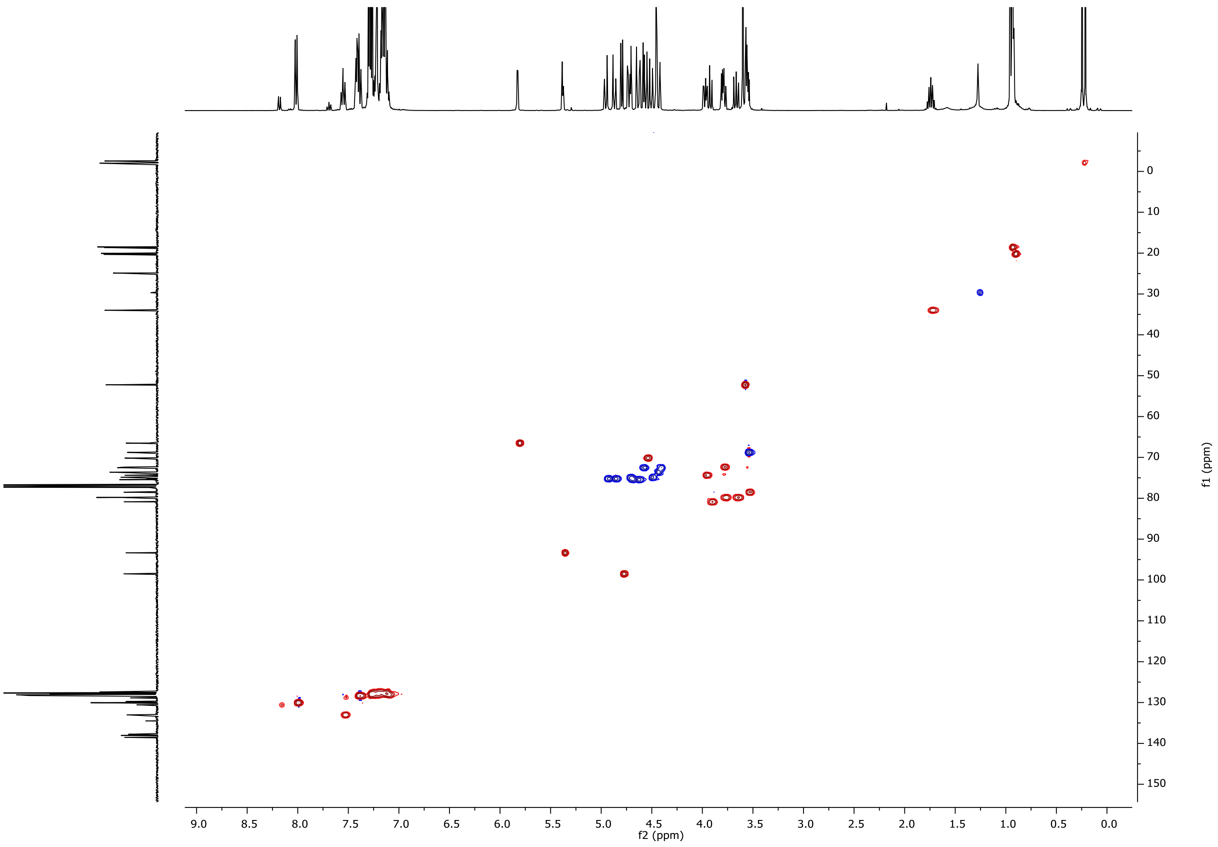
9.0 8.5 8.0 7.5 7.0 6.5 6.0 5.5 5.0 4.5 4.0 3.5 3.0 2.5 2.0 1.5 1.0 0.5 0.0
f2 (ppm)
0 10 20 30 40 50 60 70 80 90 100 110 120 130 140 150
f1 (ppm)

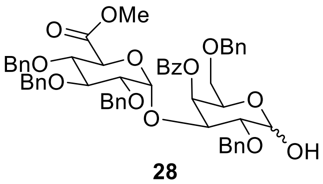

28

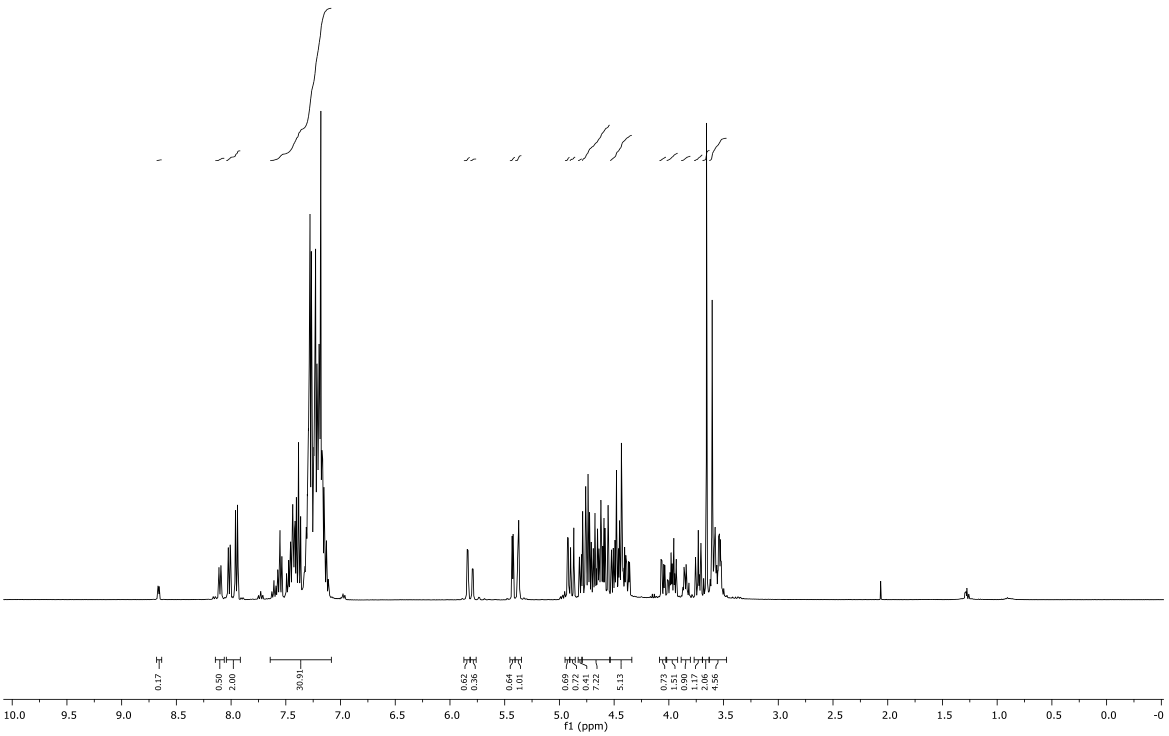

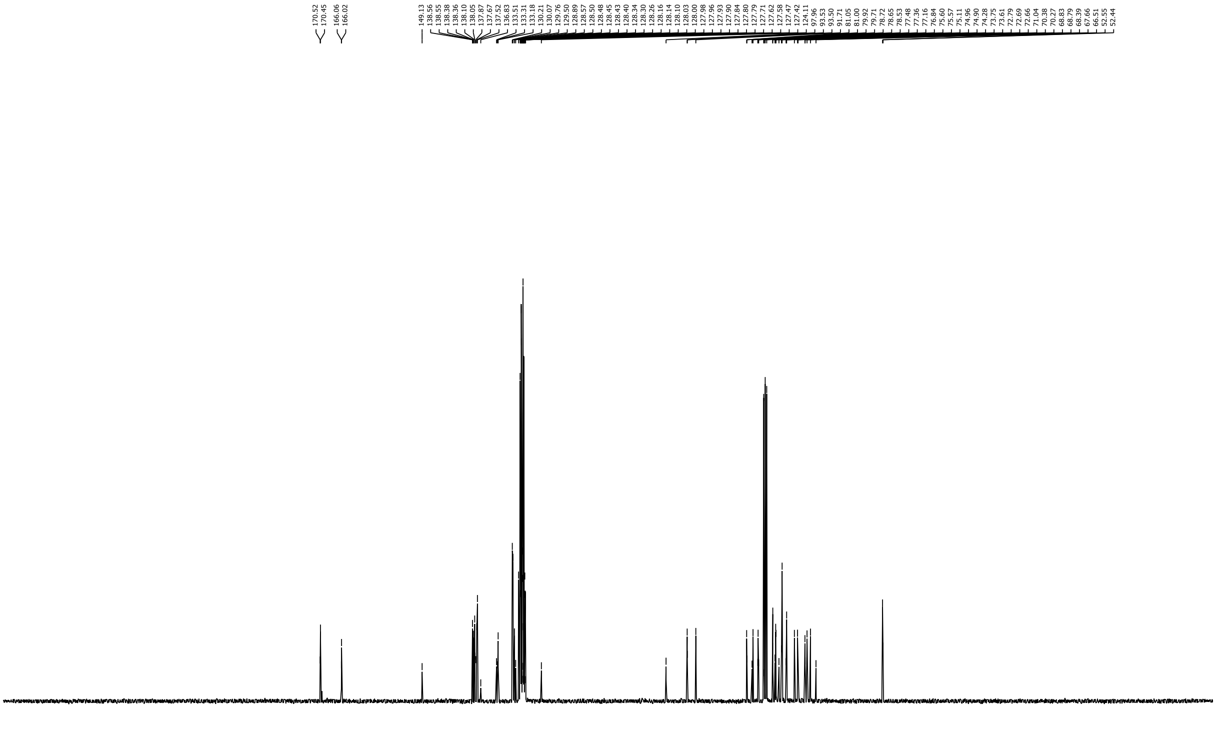

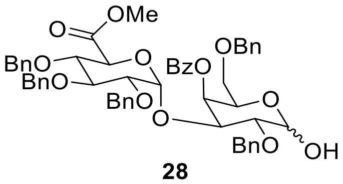

28

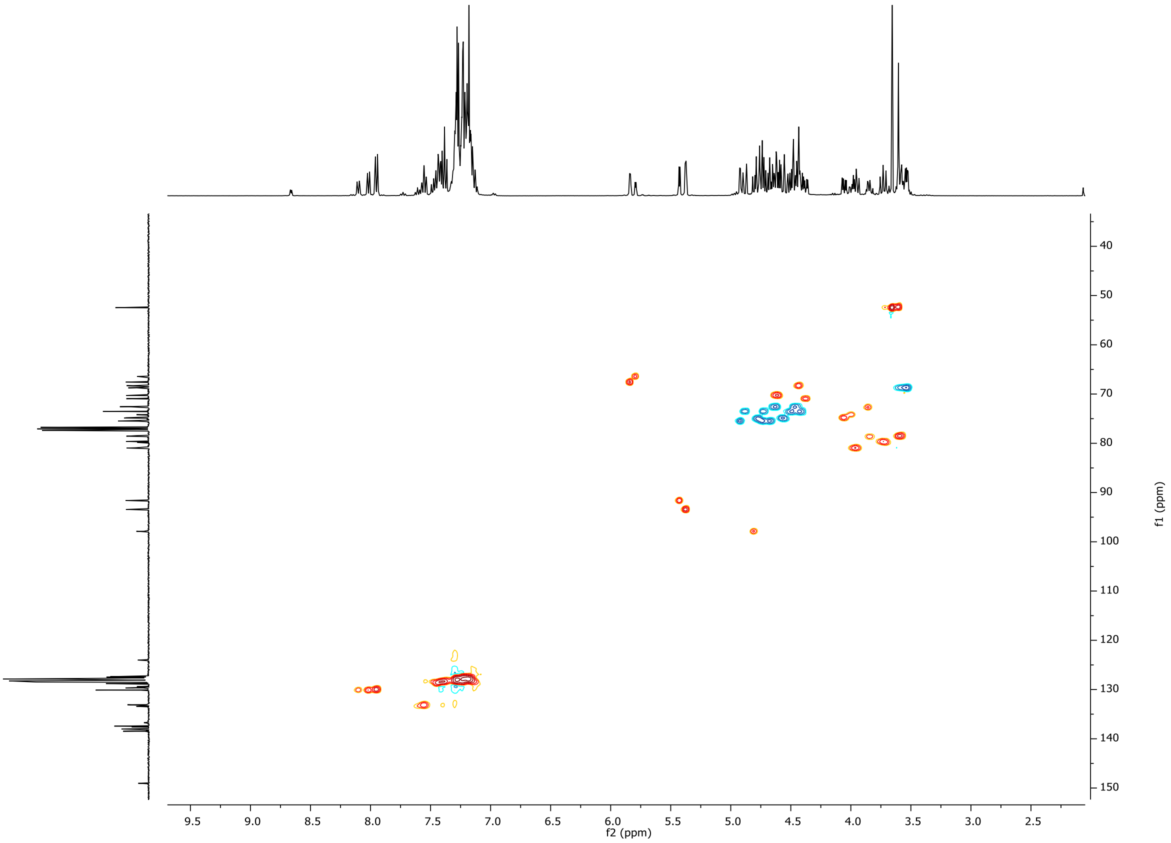

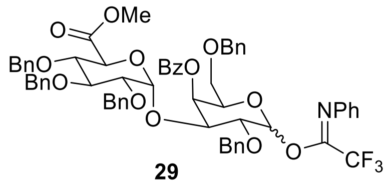

29

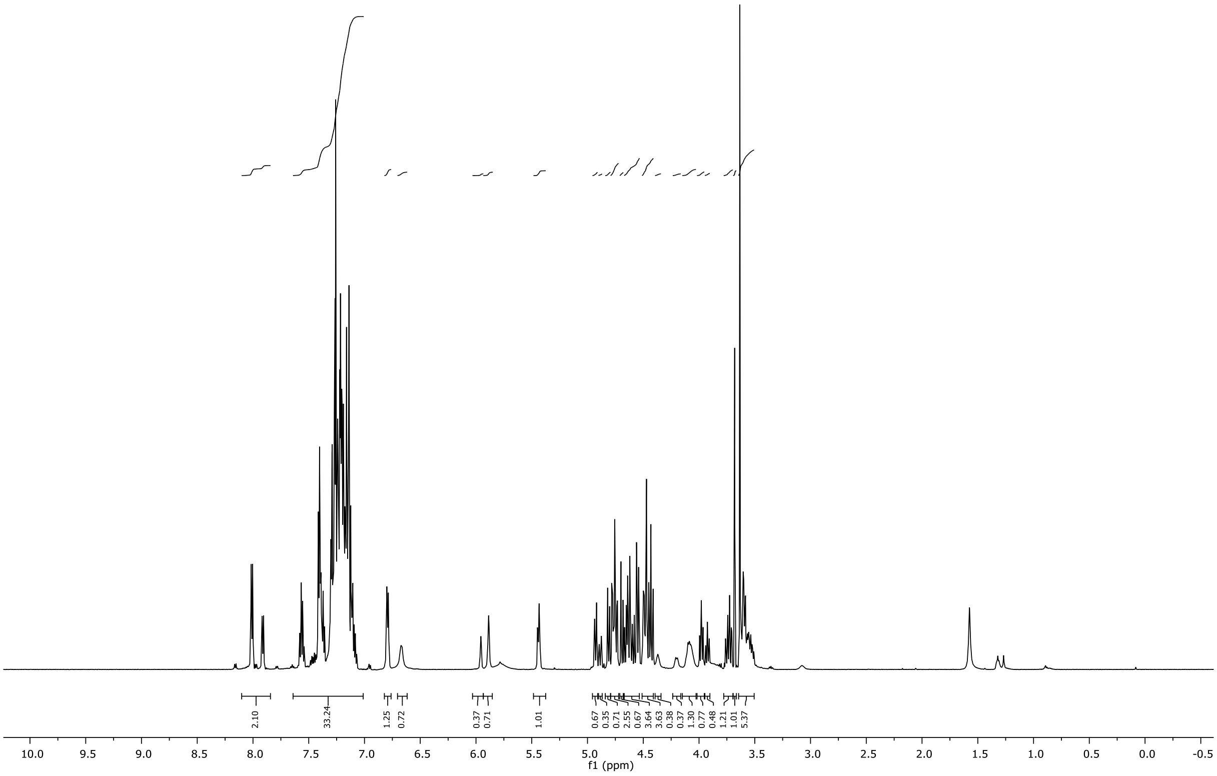

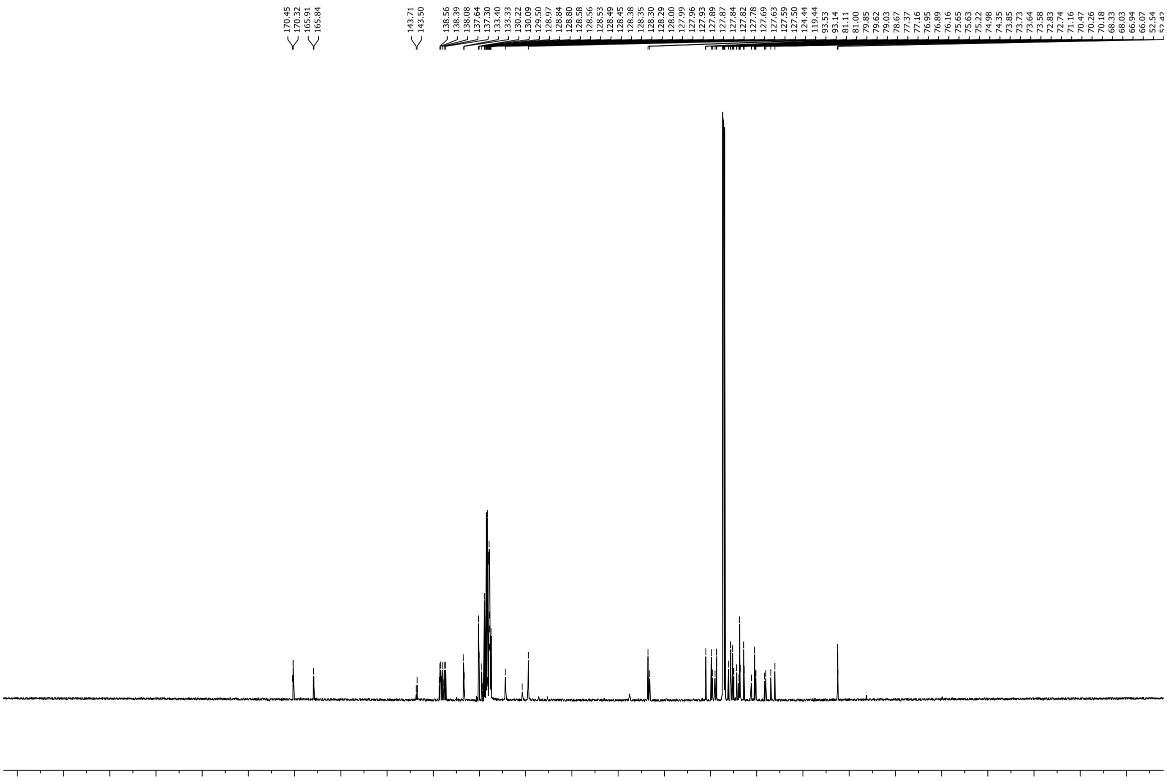

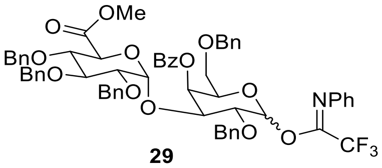

29

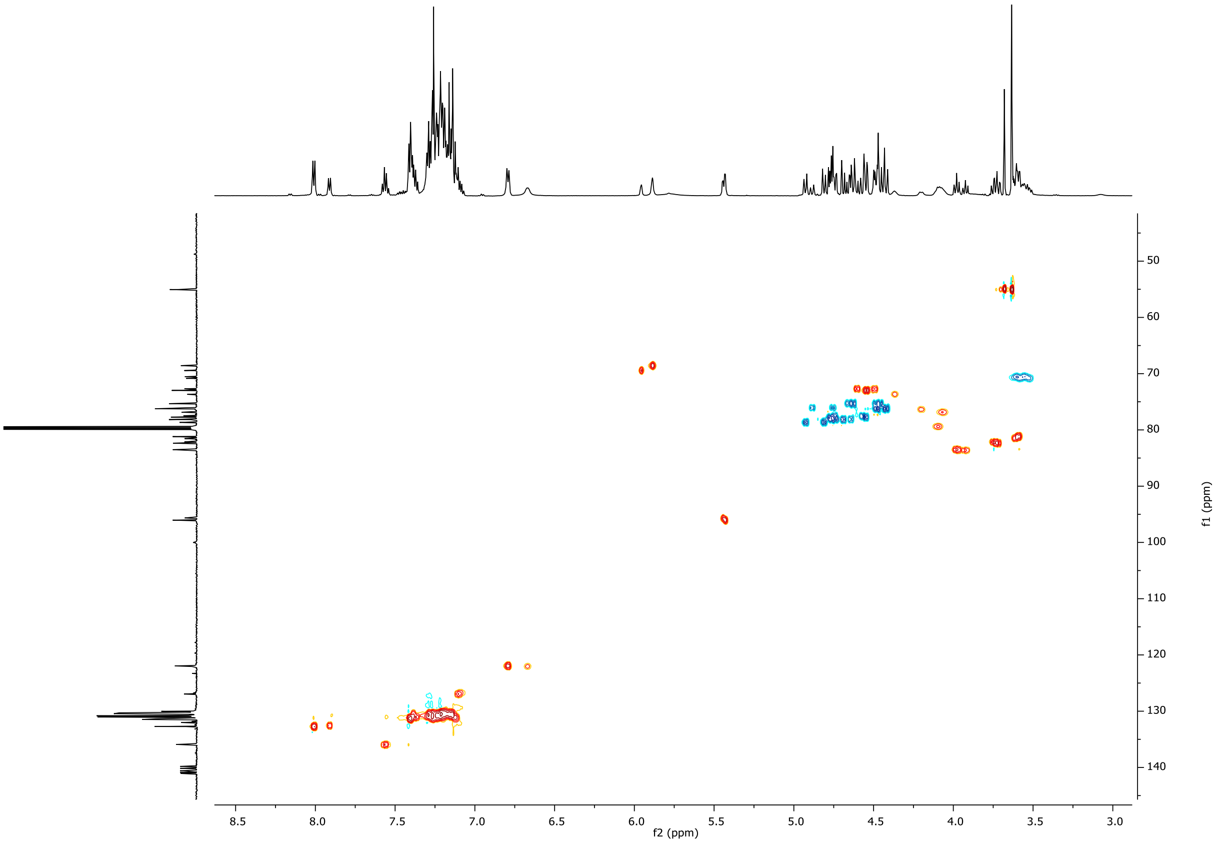

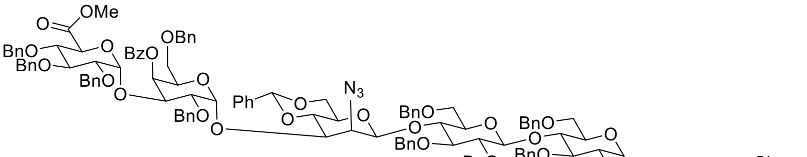

30

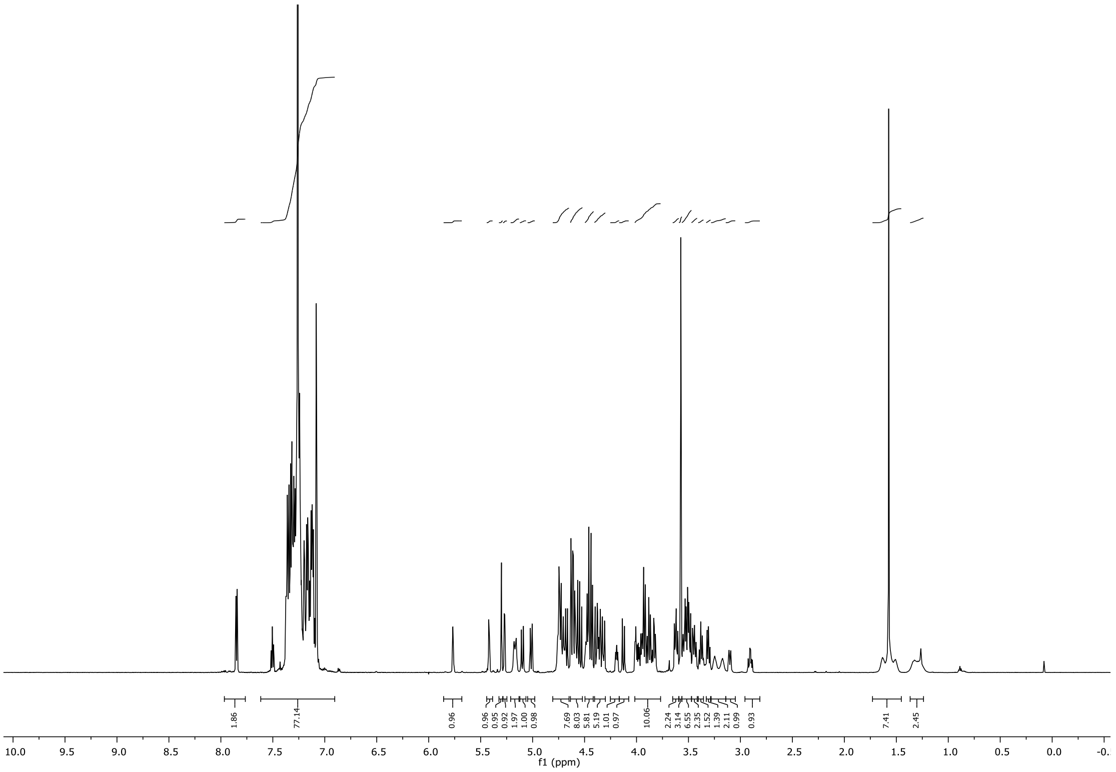

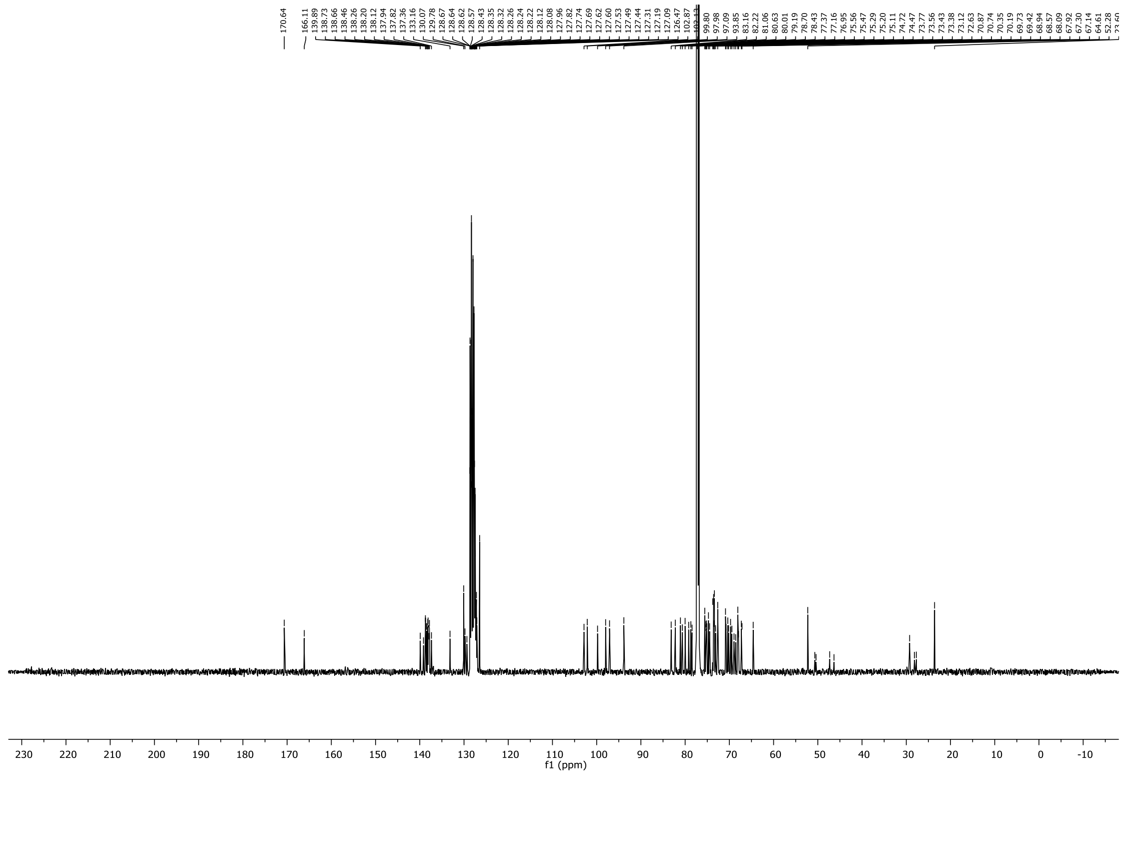

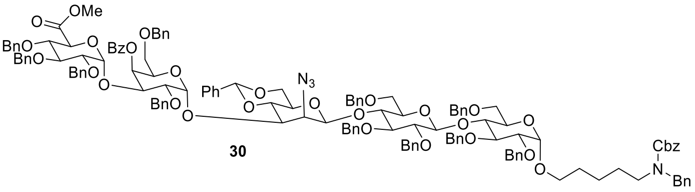
OMe
OBn
BnO
BnO
BzO
BnO
O
Ph
N3
BnO
BnO
BnO
BnO
BnO
BnO
Cbz
N
Bn

30

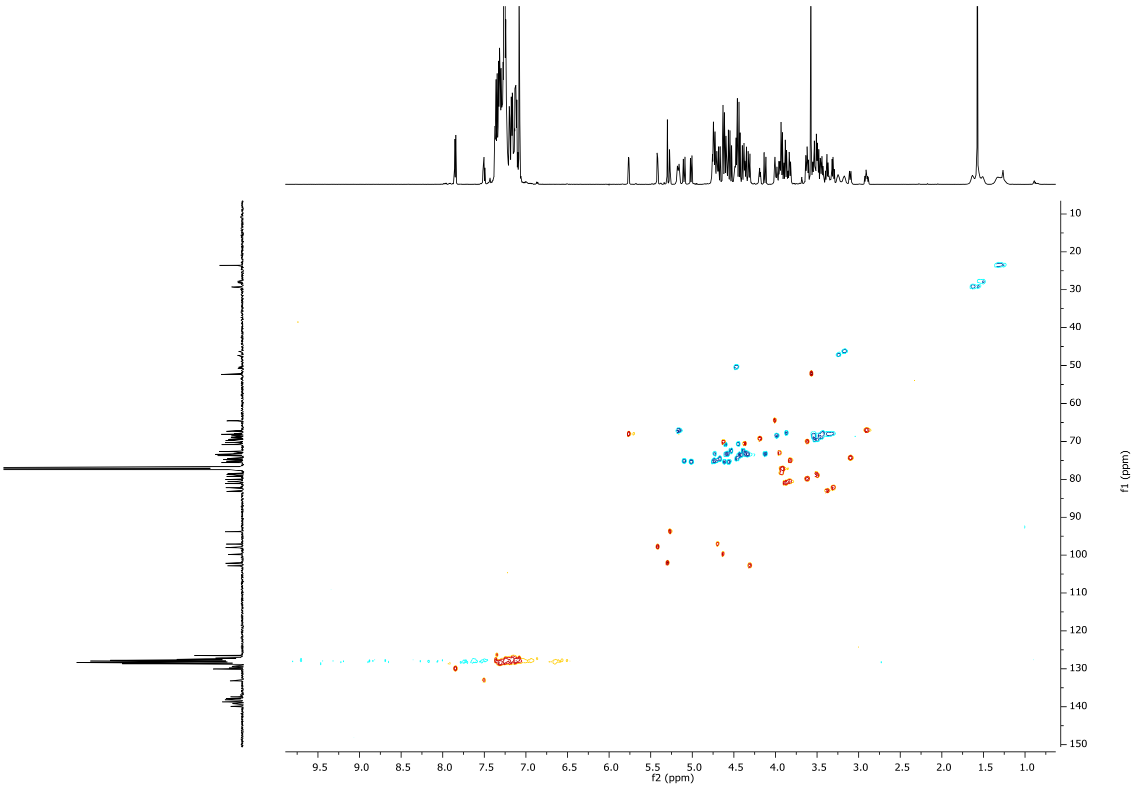
f2 (ppm)
f1 (ppm)

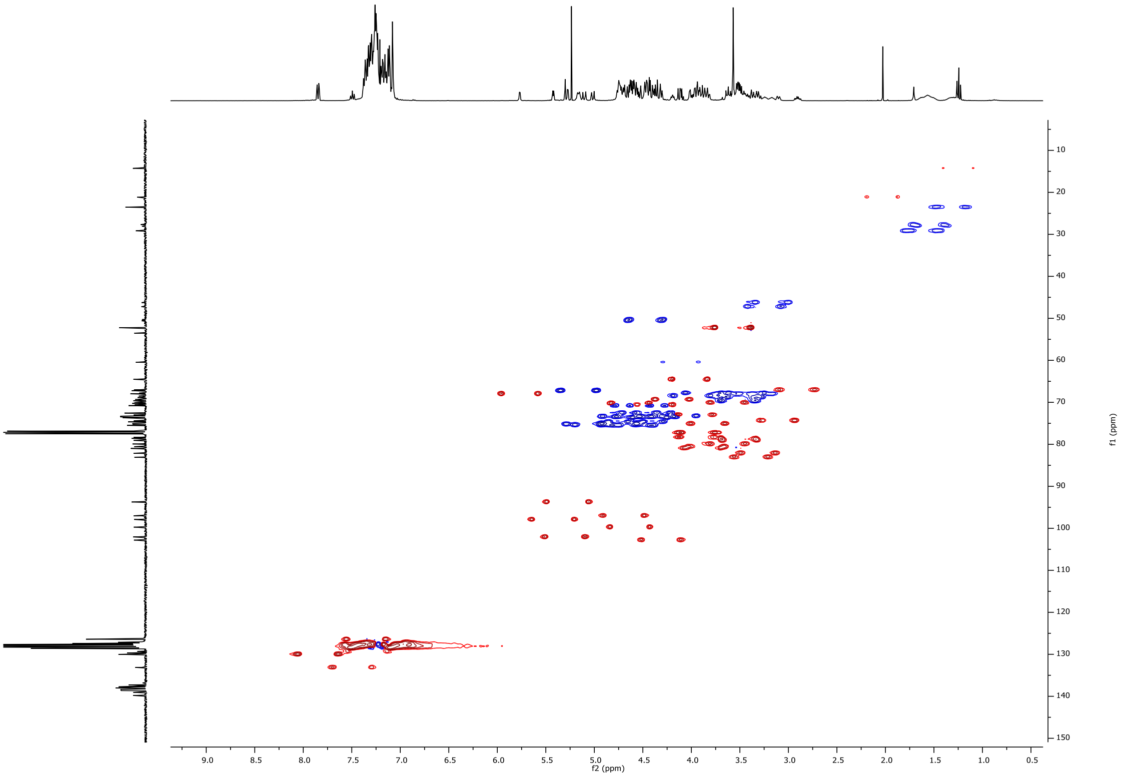
f2 (ppm)
f1 (ppm)

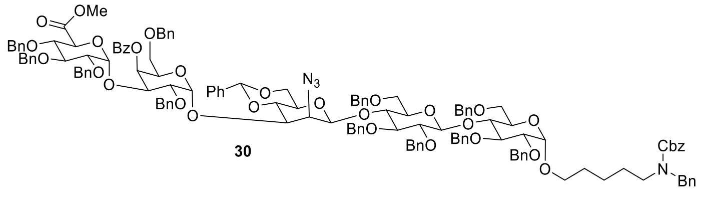
OMe
BnO
BnO
BnO
BzO
OBn
Ph
N3
BnO
Cbz
N
Bn

30

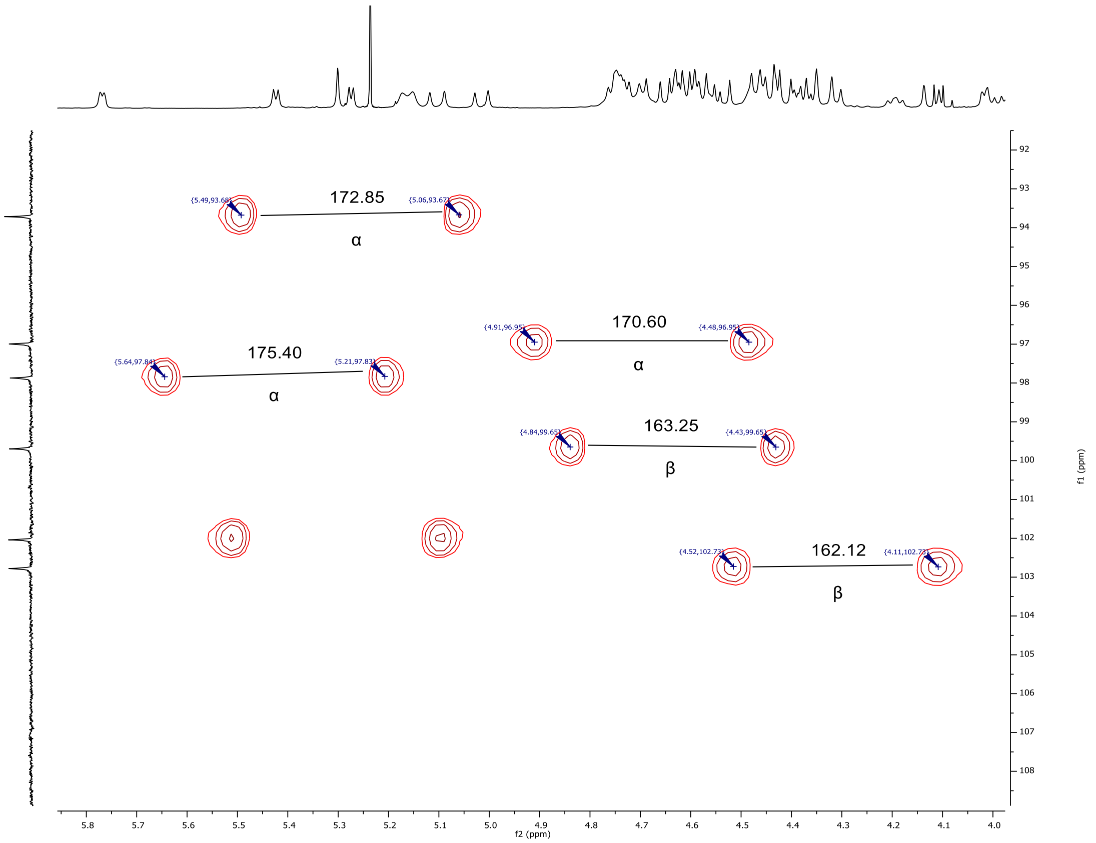
172.85
α
170.60
α
175.40
α
163.25
β
162.12
β
f2 (ppm)
f1 (ppm)

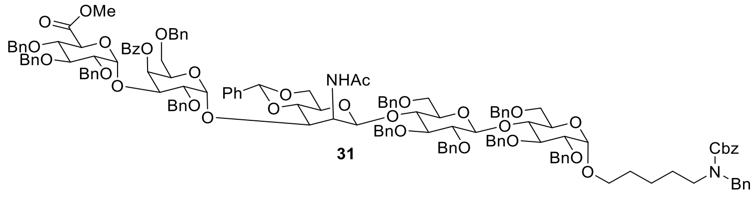
OMe
O
BnO
BnO
BnO
O
BzO
OBn
O
O
BnO
O
Ph
O
O
NHAc
O
BnO
O
BnO
BnO
BnO
O
BnO
O
BnO
O
Cbz
N
Bn
31

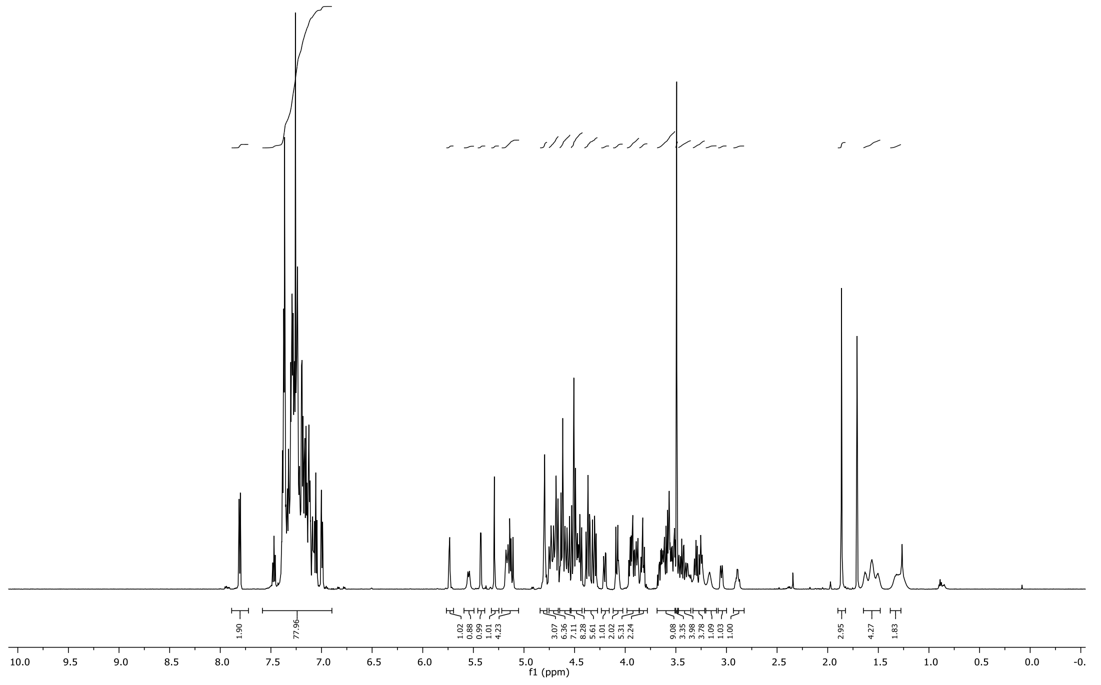
f1 (ppm)

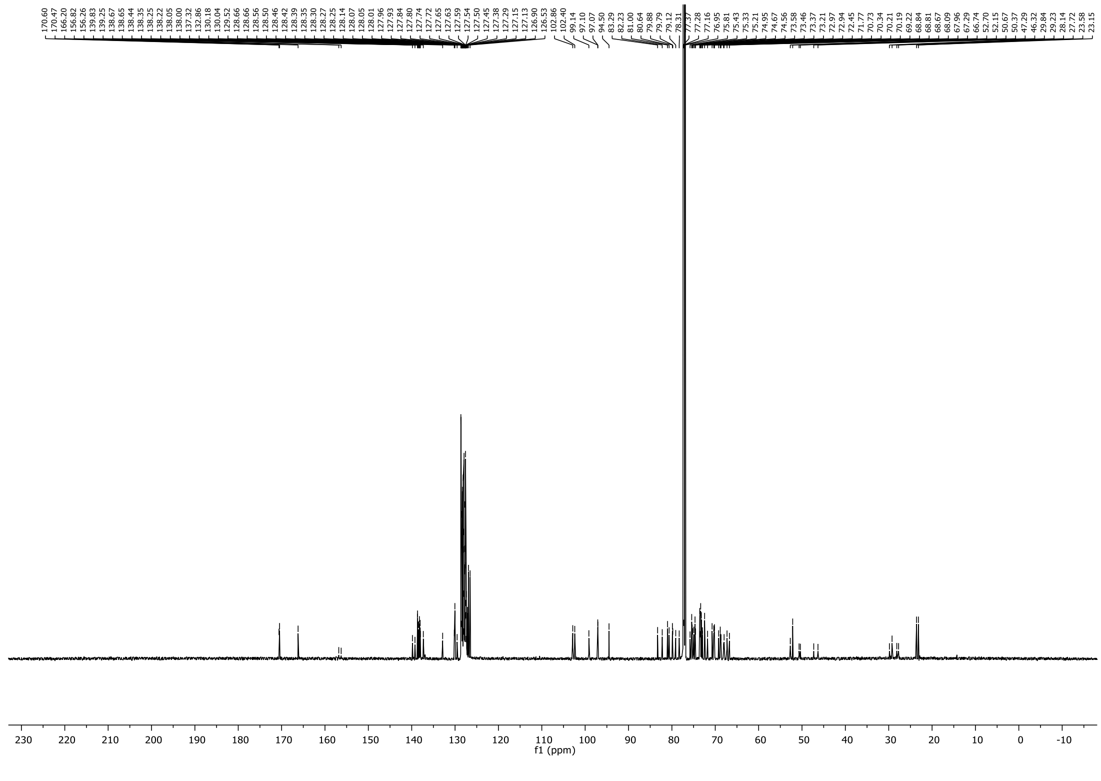
f1 (ppm)

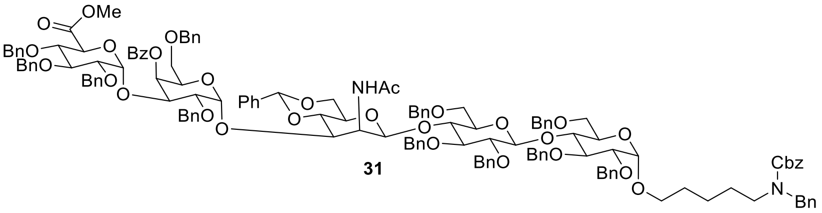

31

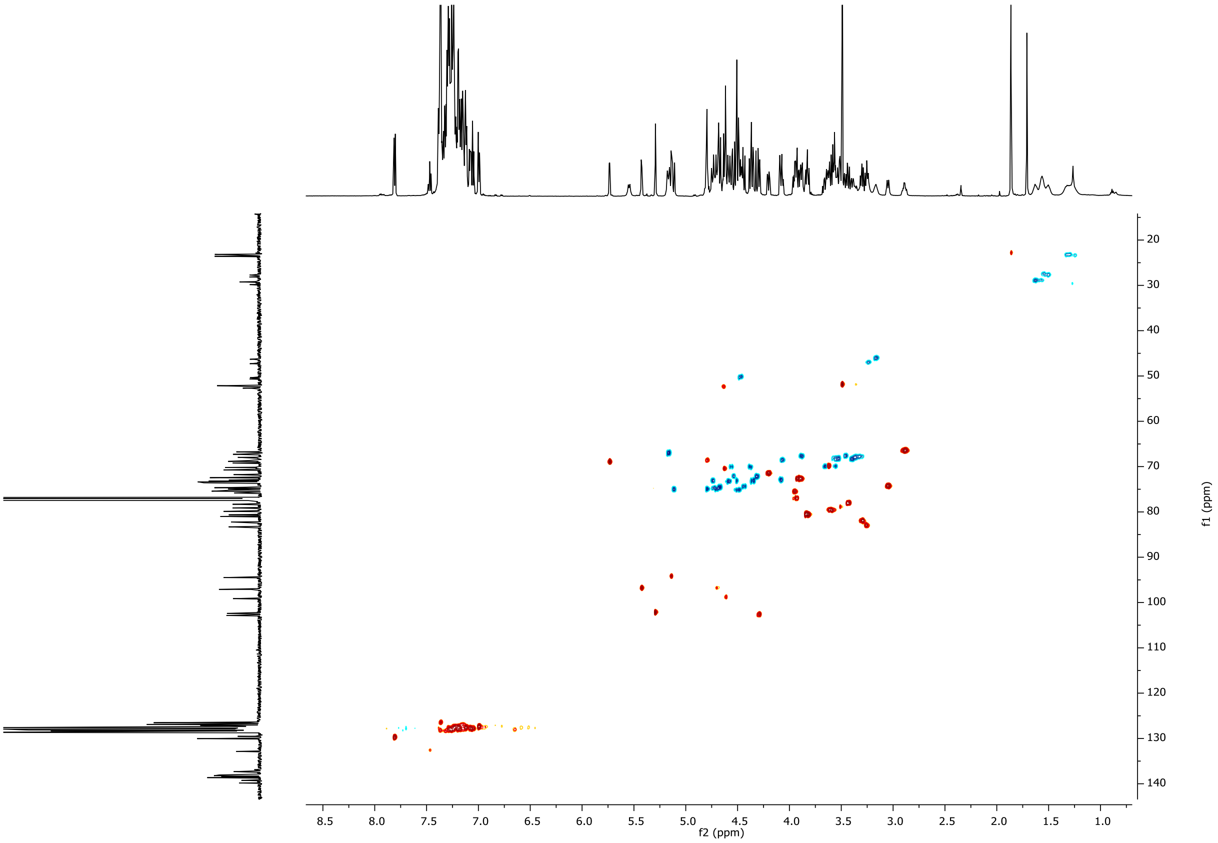

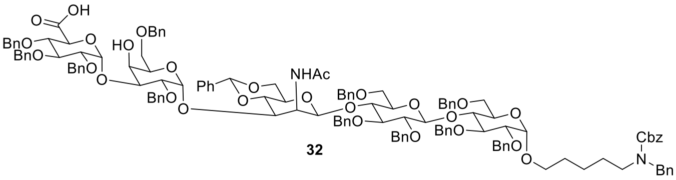

32

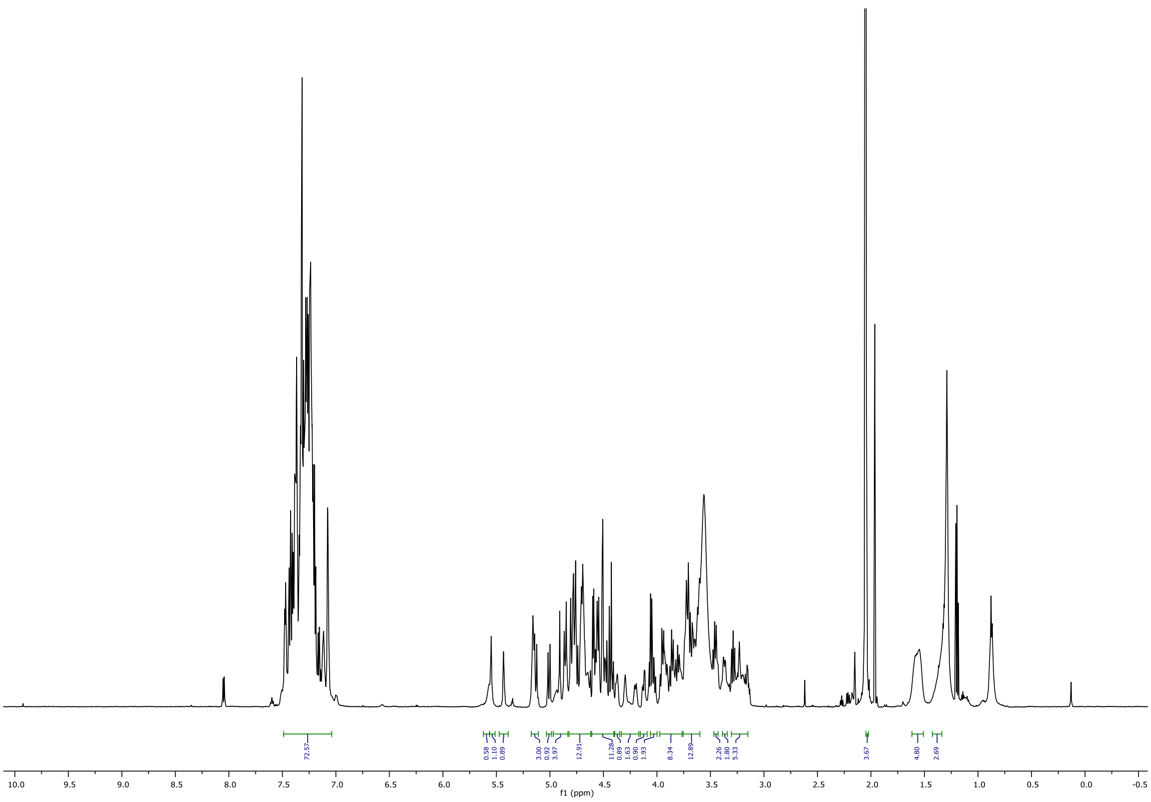
f1 (ppm)

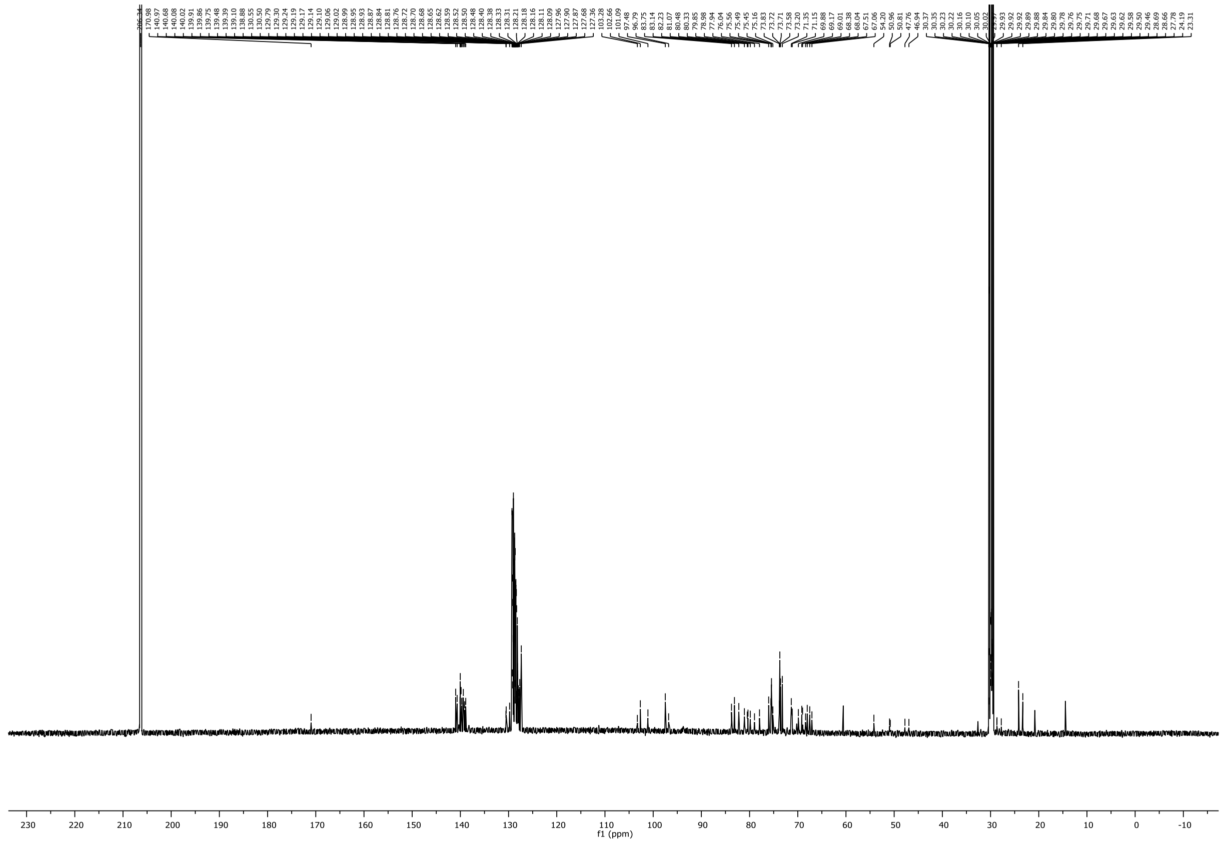
f1 (ppm)

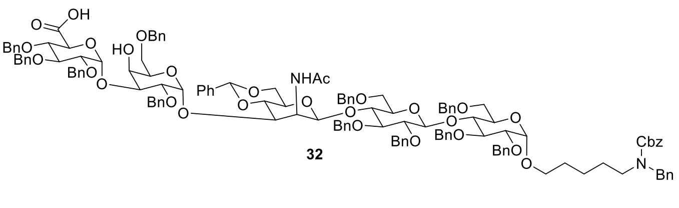
OH
OBn
HO
NHAc
Ph
Cbz
N
Bn
32

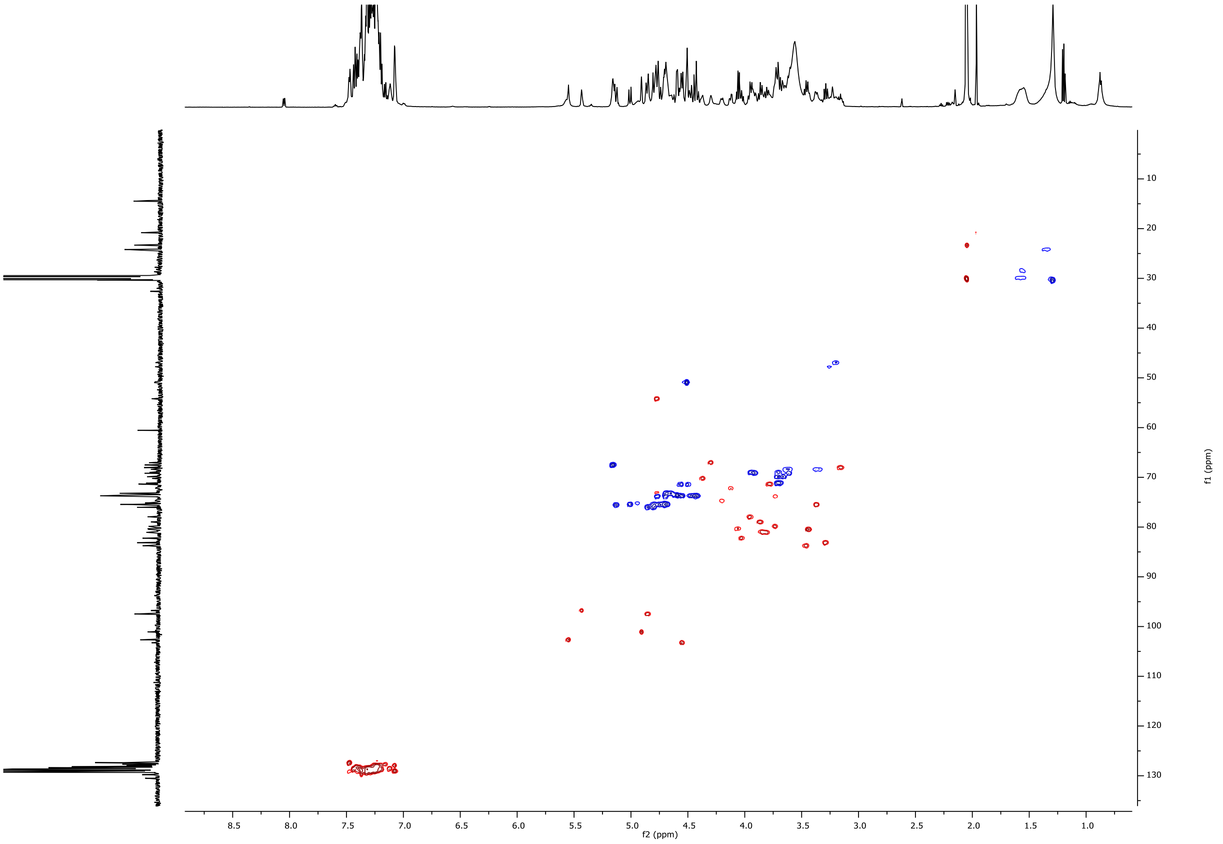

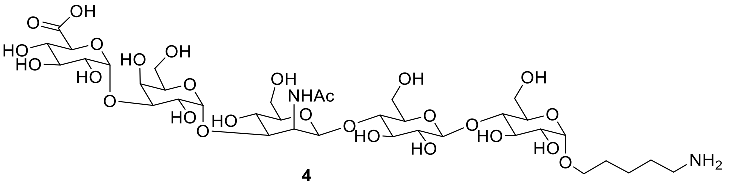

4

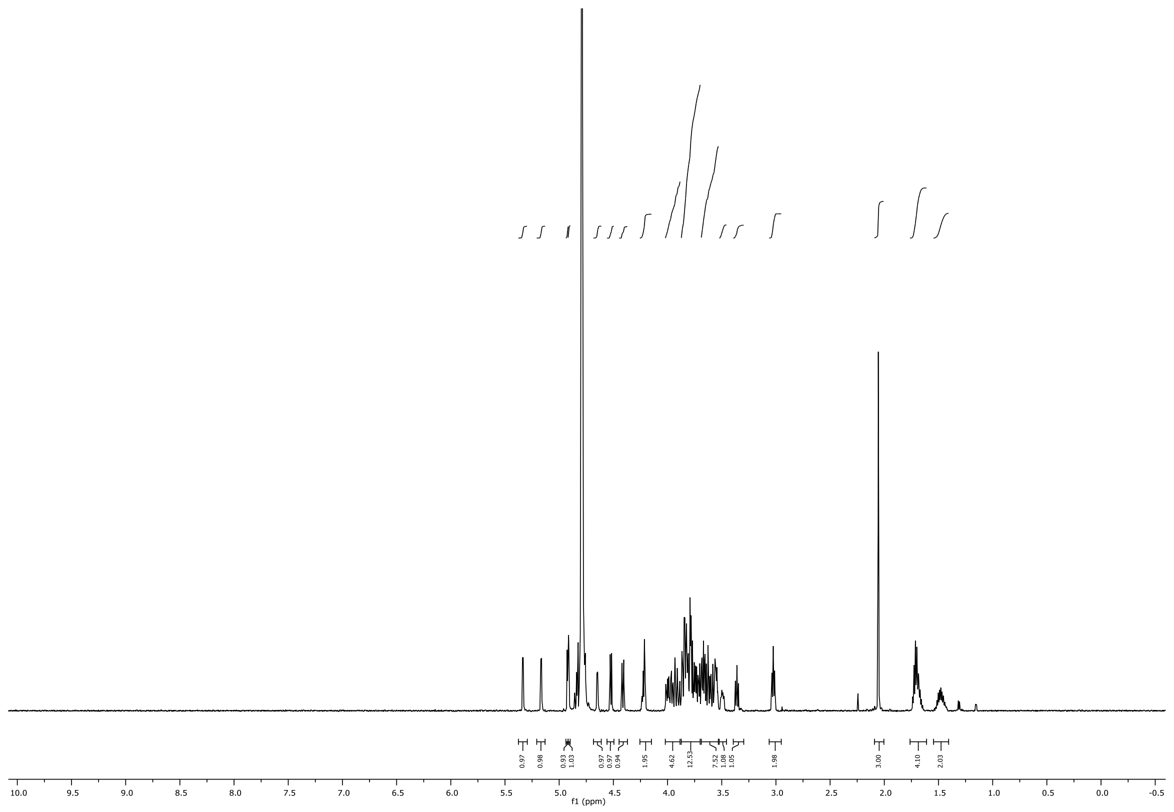

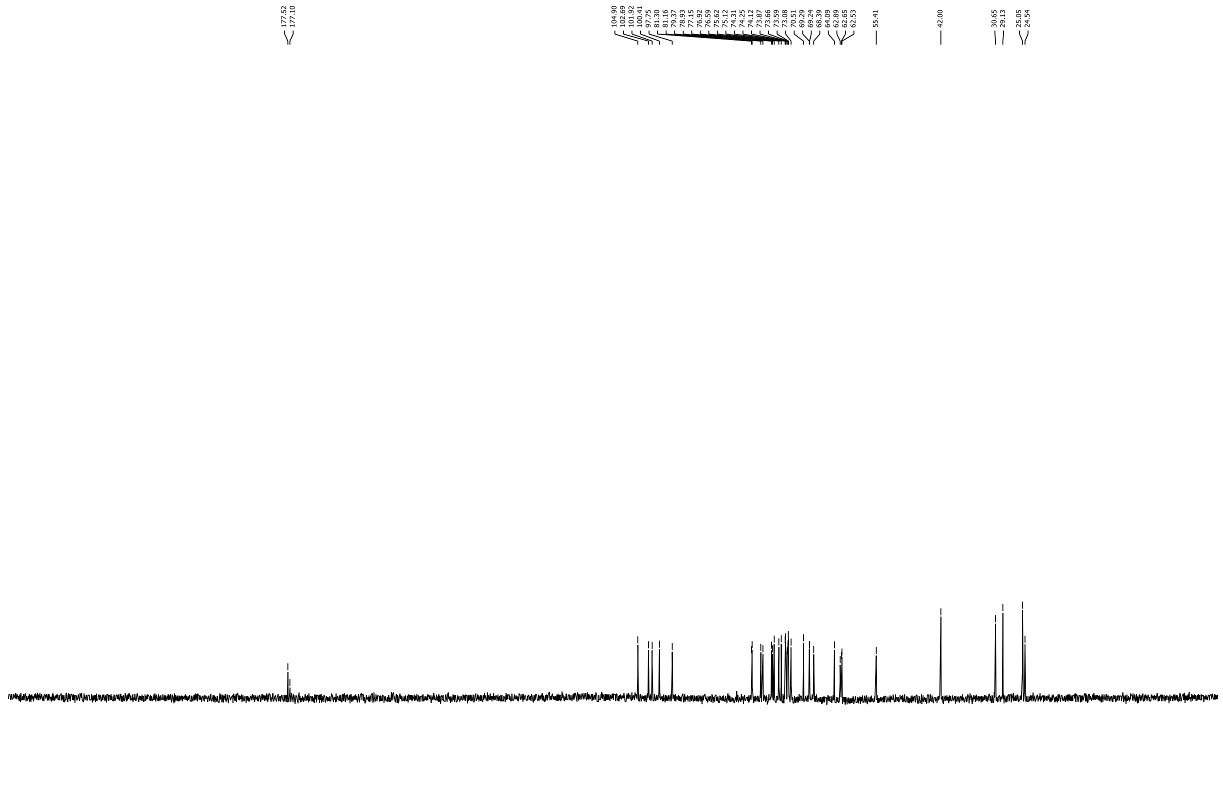

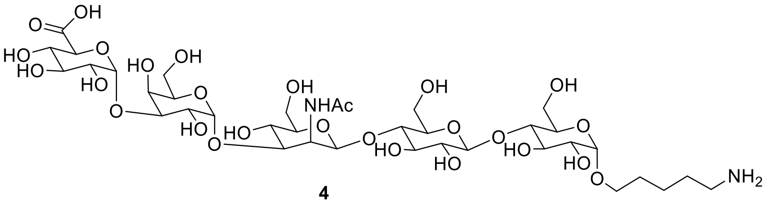

4

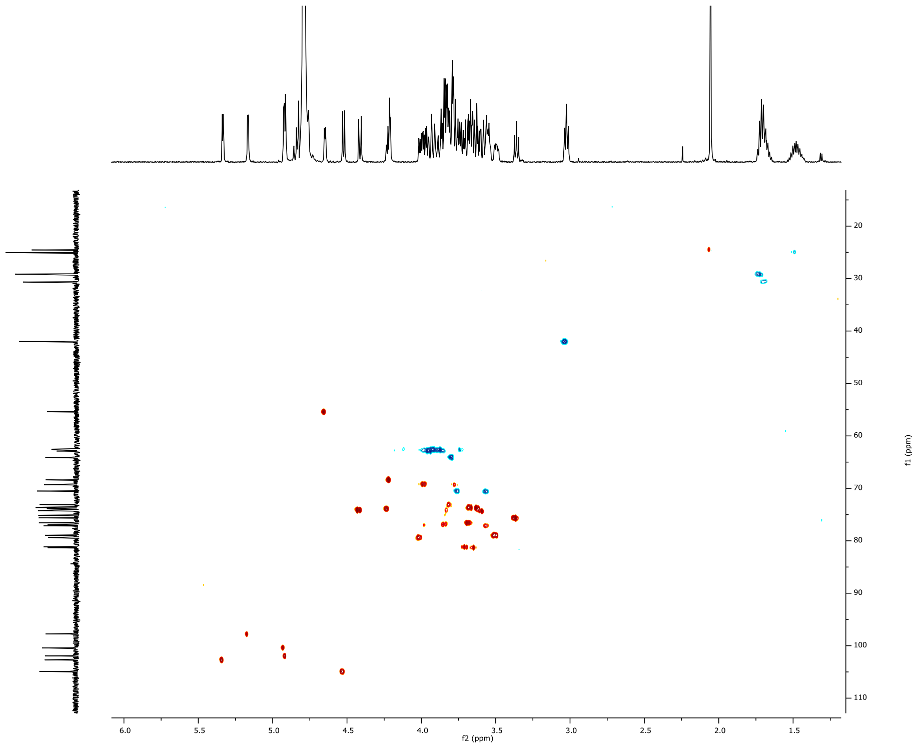

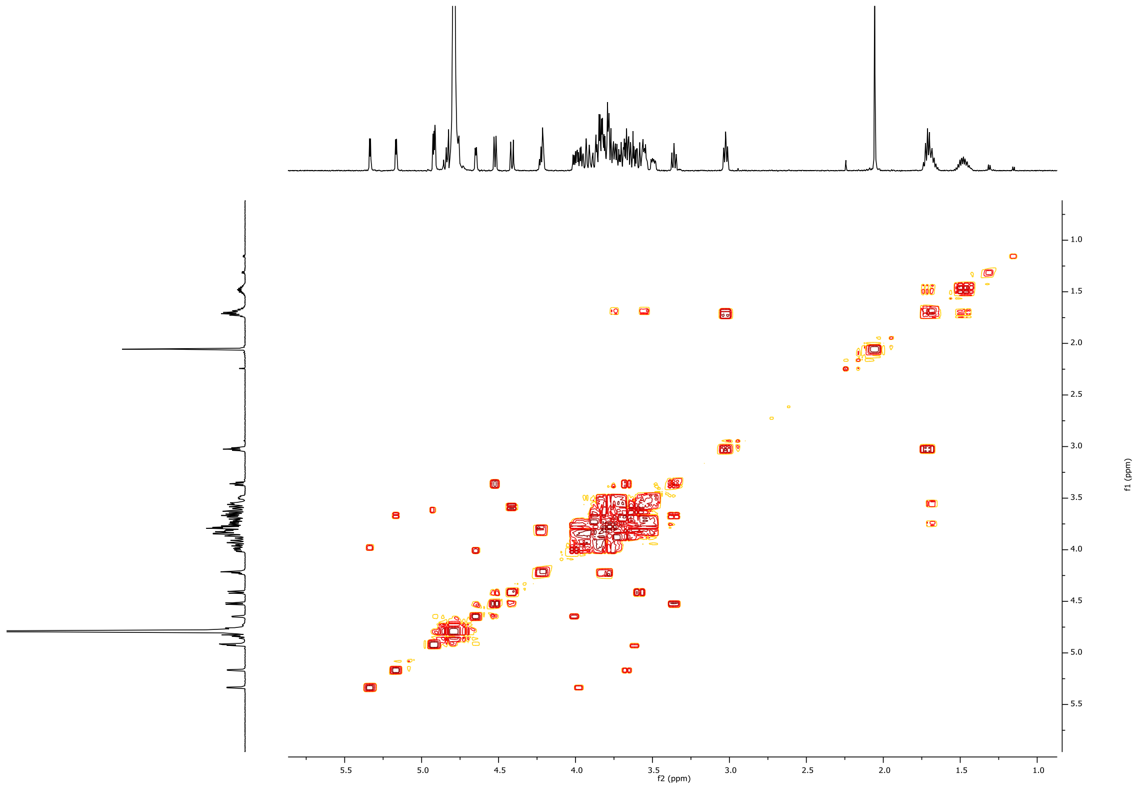

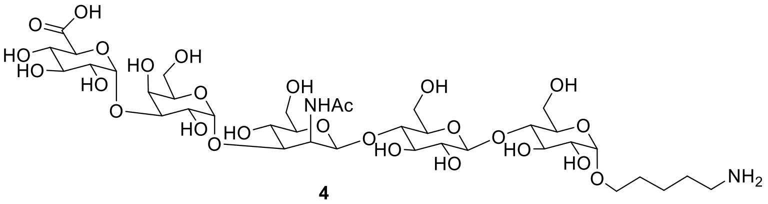

4

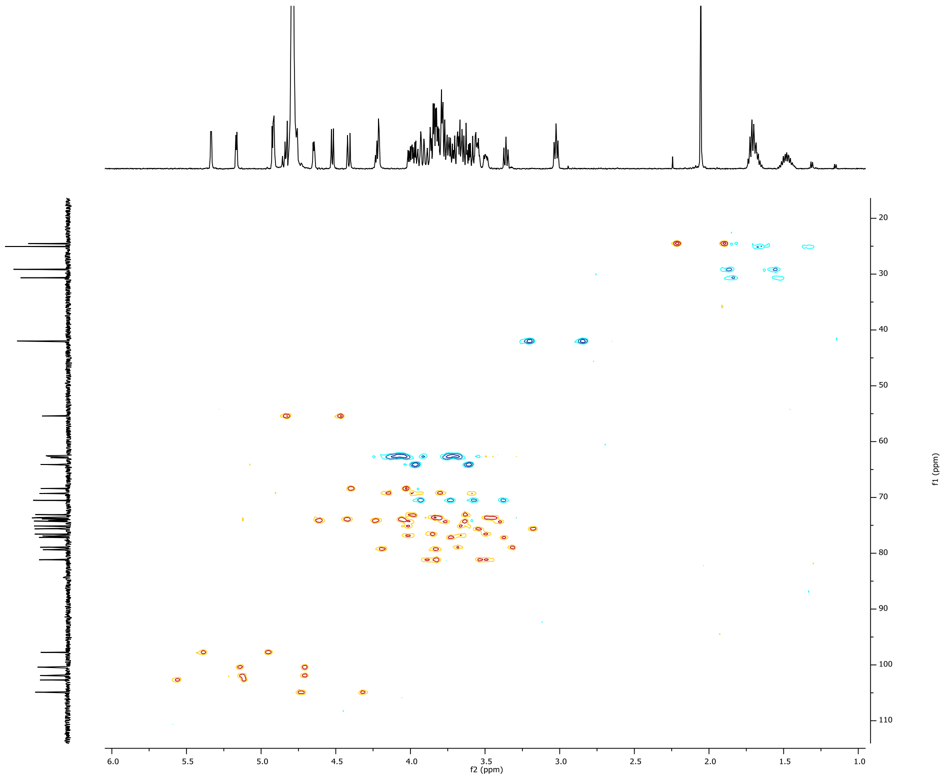

f2 (ppm)
f1 (ppm)

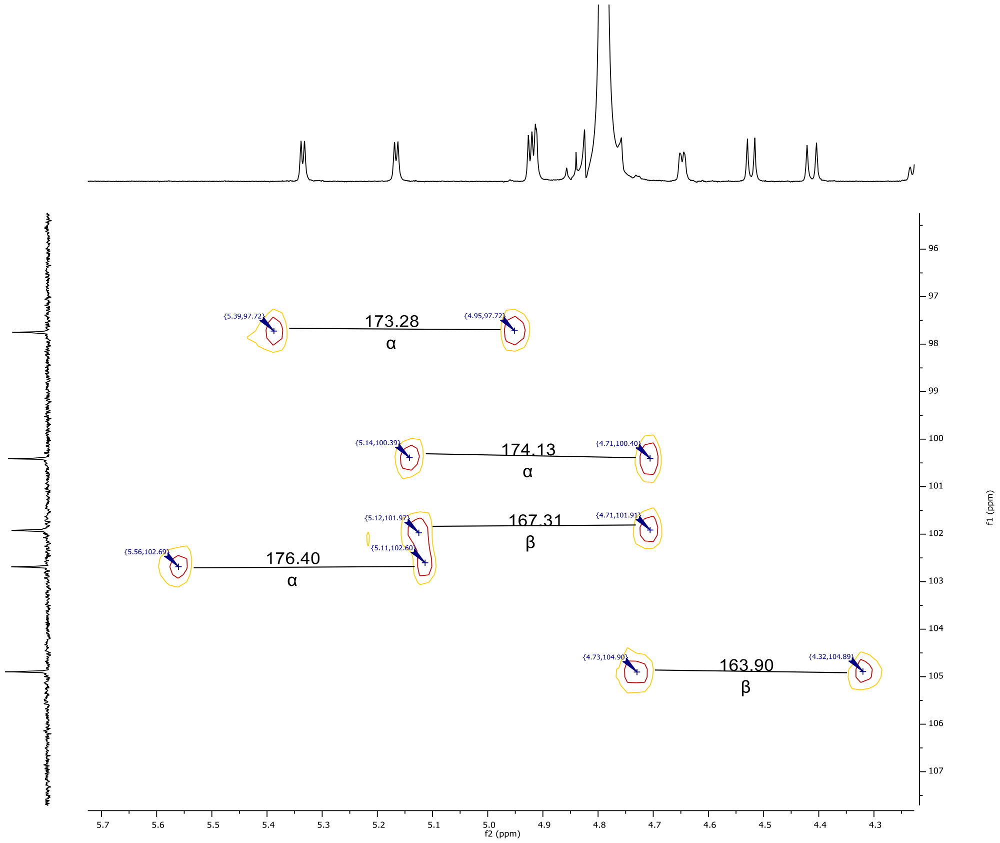

173.28
α
174.13
α
167.31
β
176.40
α
163.90
β
f2 (ppm)
f1 (ppm)

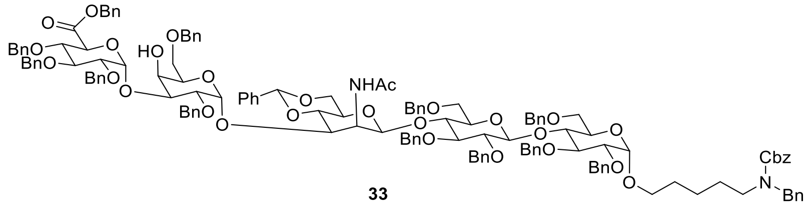
OBn
BnO
BnO
BnO
HO
OBn
BnO
Ph
NHAc
BnO
BnO
BnO
BnO
BnO
BnO
Cbz
N
Bn

33

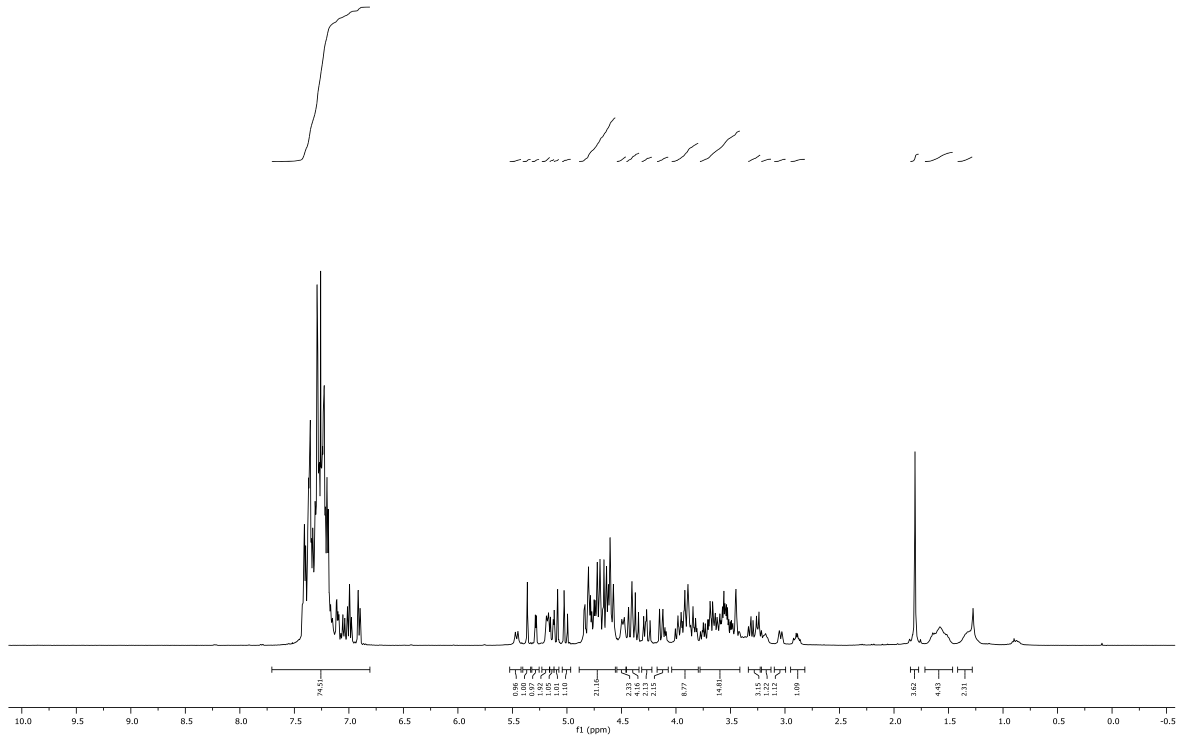
74.51
0.96
1.00
0.97
1.92
1.05
1.01
1.10
21.16
2.33
4.16
2.13
2.15
8.77
14.81
3.15
1.22
1.12
1.09
3.62
4.43
2.31
10.0 9.5 9.0 8.5 8.0 7.5 7.0 6.5 6.0 5.5 5.0 4.5 4.0 3.5 3.0 2.5 2.0 1.5 1.0 0.5 0.0 -0.5
f1 (ppm)

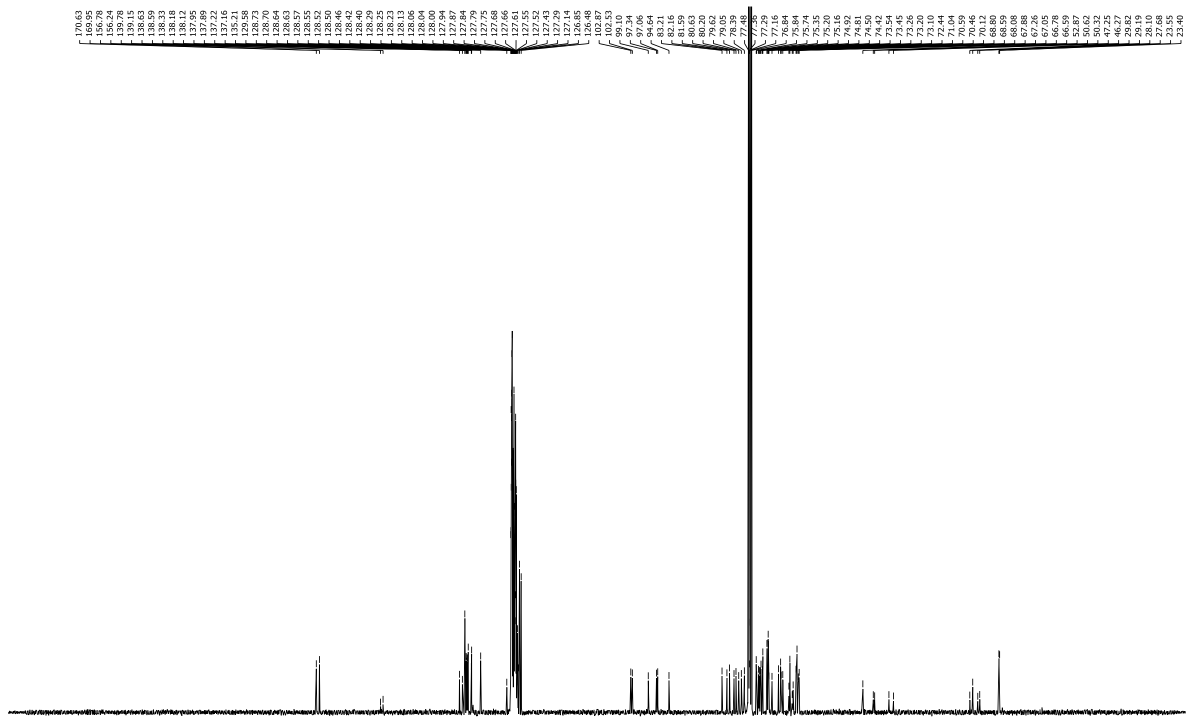
170.63 169.95 156.78 156.24 139.78 139.15 138.63 138.59 138.33 138.18 138.12 137.95 137.89 137.22 137.16 135.21 129.58 128.73 128.70 128.64 128.63 128.57 128.55 128.52 128.50 128.46 128.42 128.40 128.29 128.25 128.23 128.13 128.06 128.04 128.00 127.94 127.87 127.84 127.79 127.75 127.68 127.66 127.61 127.55 127.52 127.43 127.29 127.14 126.85 126.48 102.87 102.53 99.10 97.34 97.06 94.64 83.21 82.16 81.59 80.63 80.20 79.62 79.05 78.39 77.48 77.36 77.29 77.16 76.84 75.84 75.74 75.35 75.20 75.16 74.92 74.81 74.50 74.42 73.54 73.45 73.26 73.20 73.10 72.44 71.04 70.59 70.46 70.12 68.80 68.59 68.08 67.88 67.26 67.05 66.78 66.59 52.87 50.62 50.32 47.25 46.27 29.82 29.19 28.10 27.68 23.55 23.40
230 220 210 200 190 180 170 160 150 140 130 120 110 100 90 80 70 60 50 40 30 20 10 0 -10
f1 (ppm)

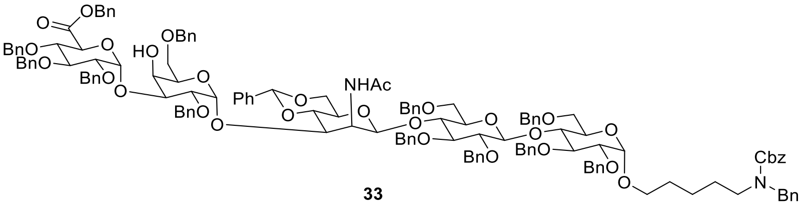
OBn
BnO
BnO
BnO
HO
OBn
BnO
Ph
NHAc
BnO
BnO
BnO
BnO
BnO
BnO
Cbz
N
Bn

33

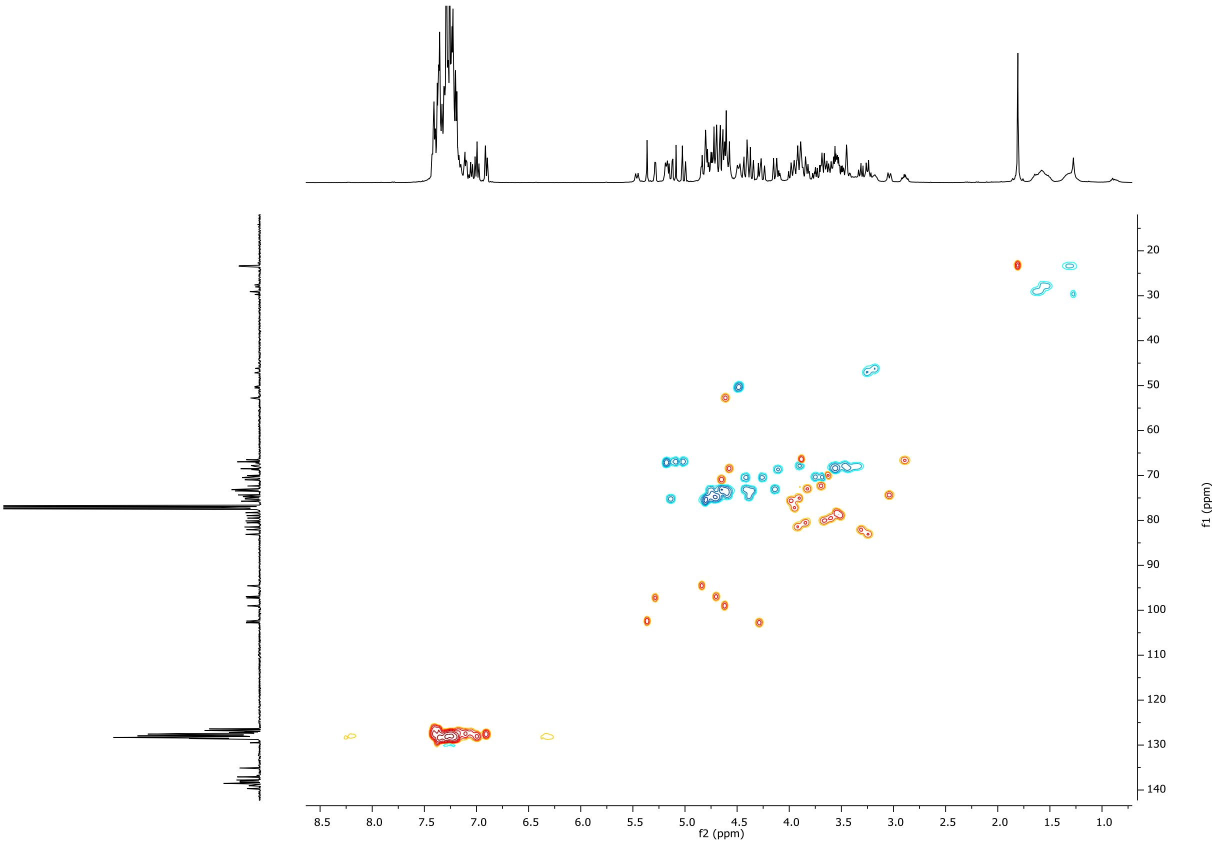
f1 (ppm)
f2 (ppm)

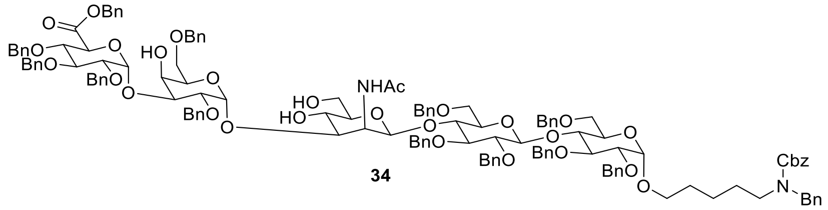

34

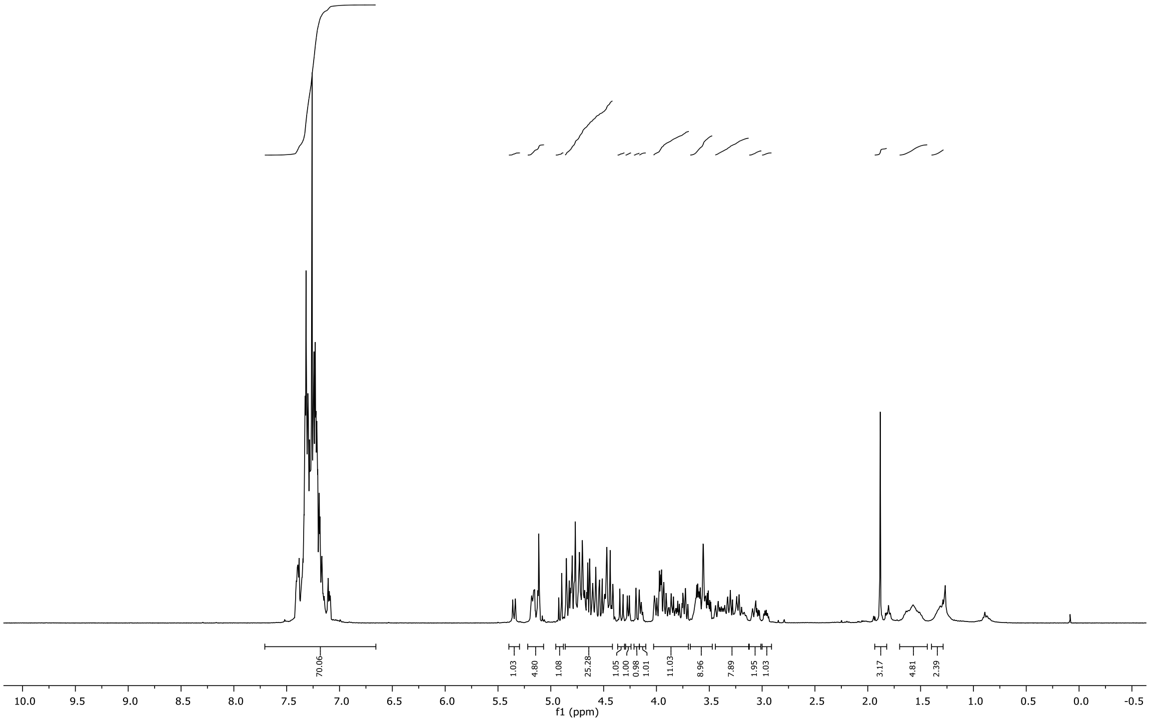

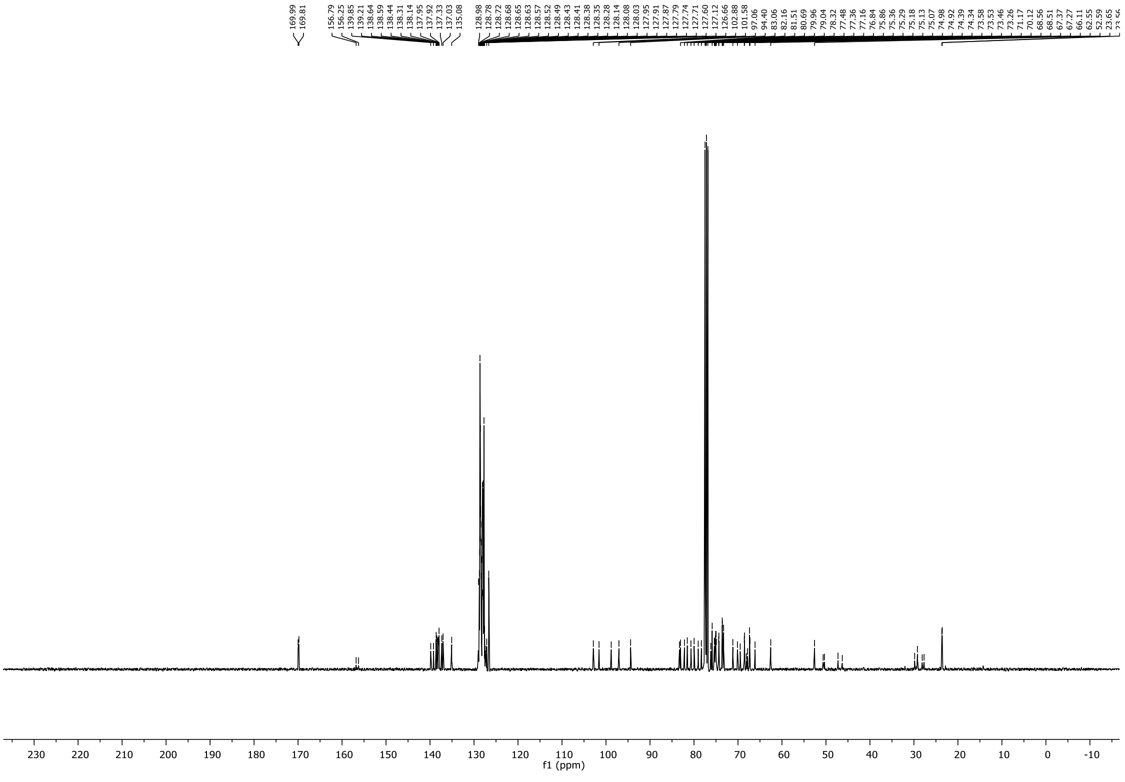

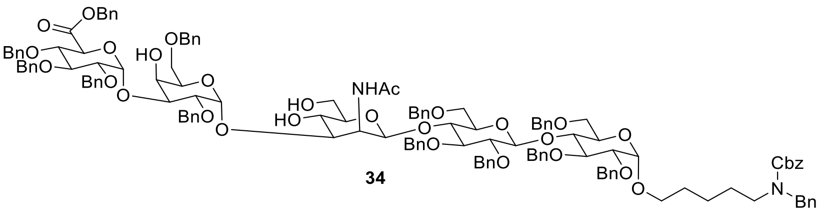
OBn
BnO
BnO
BnO
HO
OBn
BnO
HO
HO
NHAc
BnO
BnO
BnO
BnO
BnO
BnO
Cbz
N
Bn

34

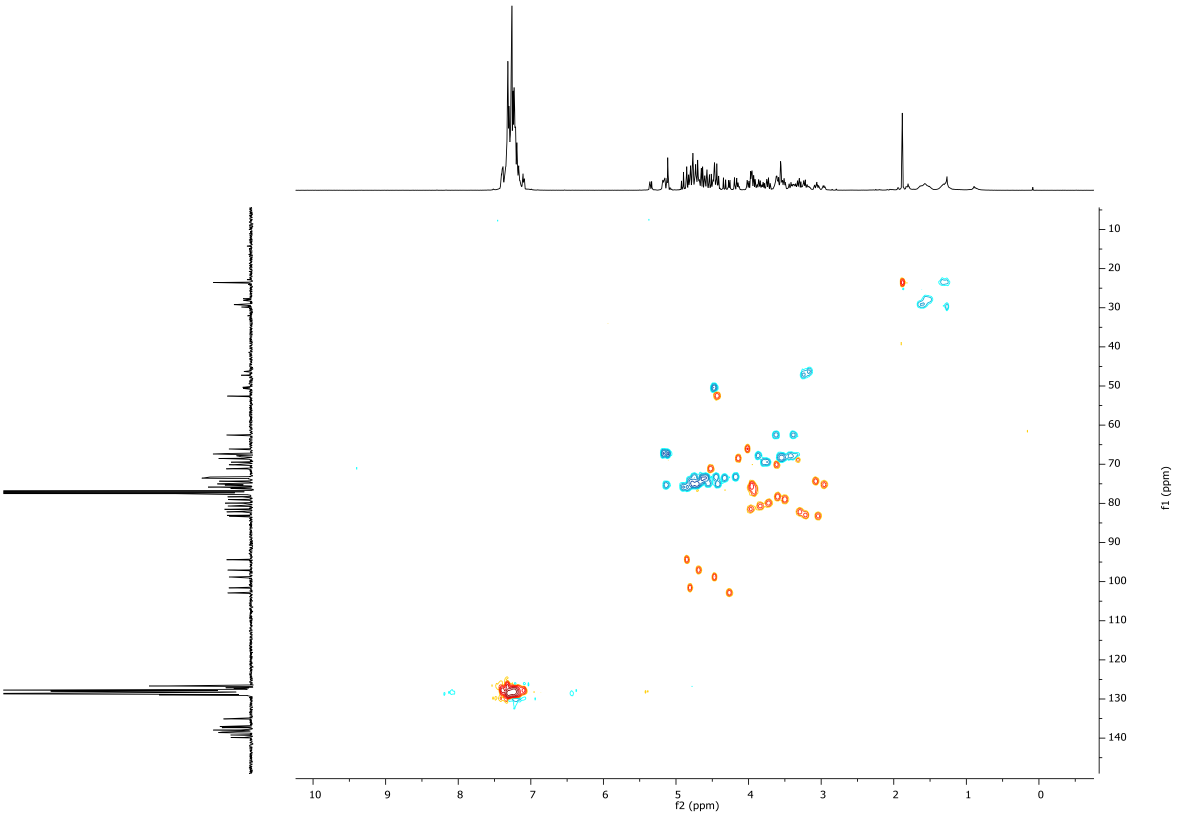
f2 (ppm)
f1 (ppm)

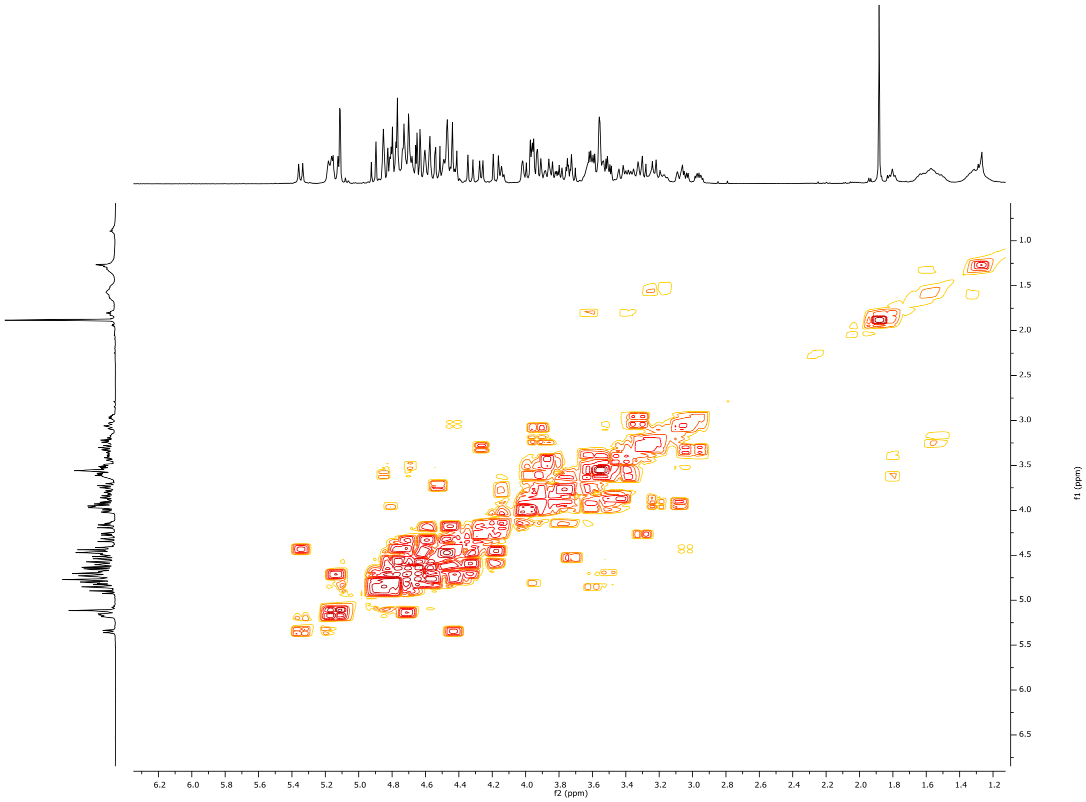
f2 (ppm)
f1 (ppm)

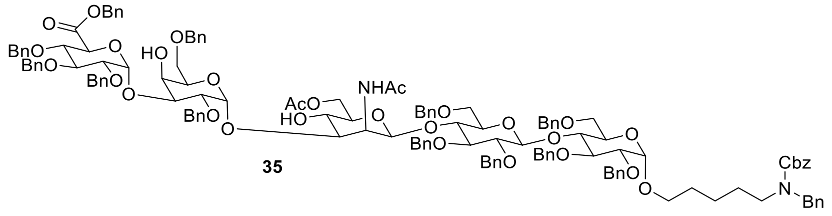
OBn
BnO
BnO
BnO
HO
OBn
BnO
AcO
HO
NHAc
BnO
BnO
BnO
BnO
BnO
BnO
Cbz
N
Bn

35

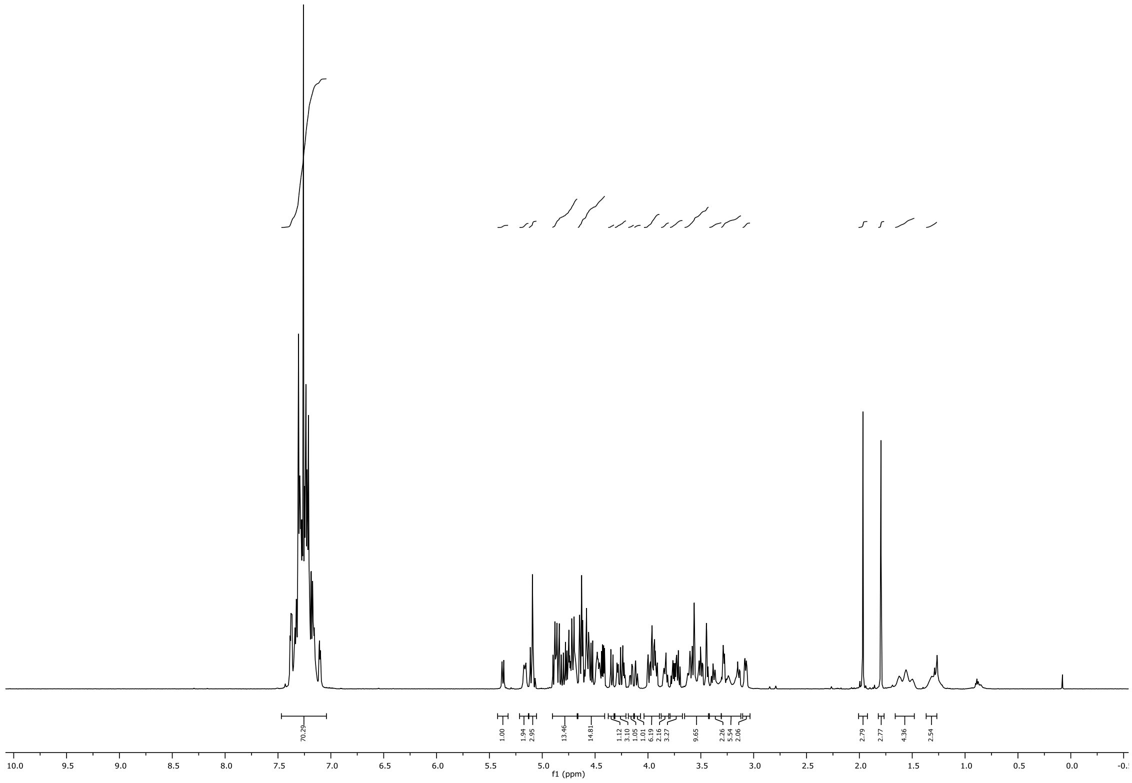
f1 (ppm)

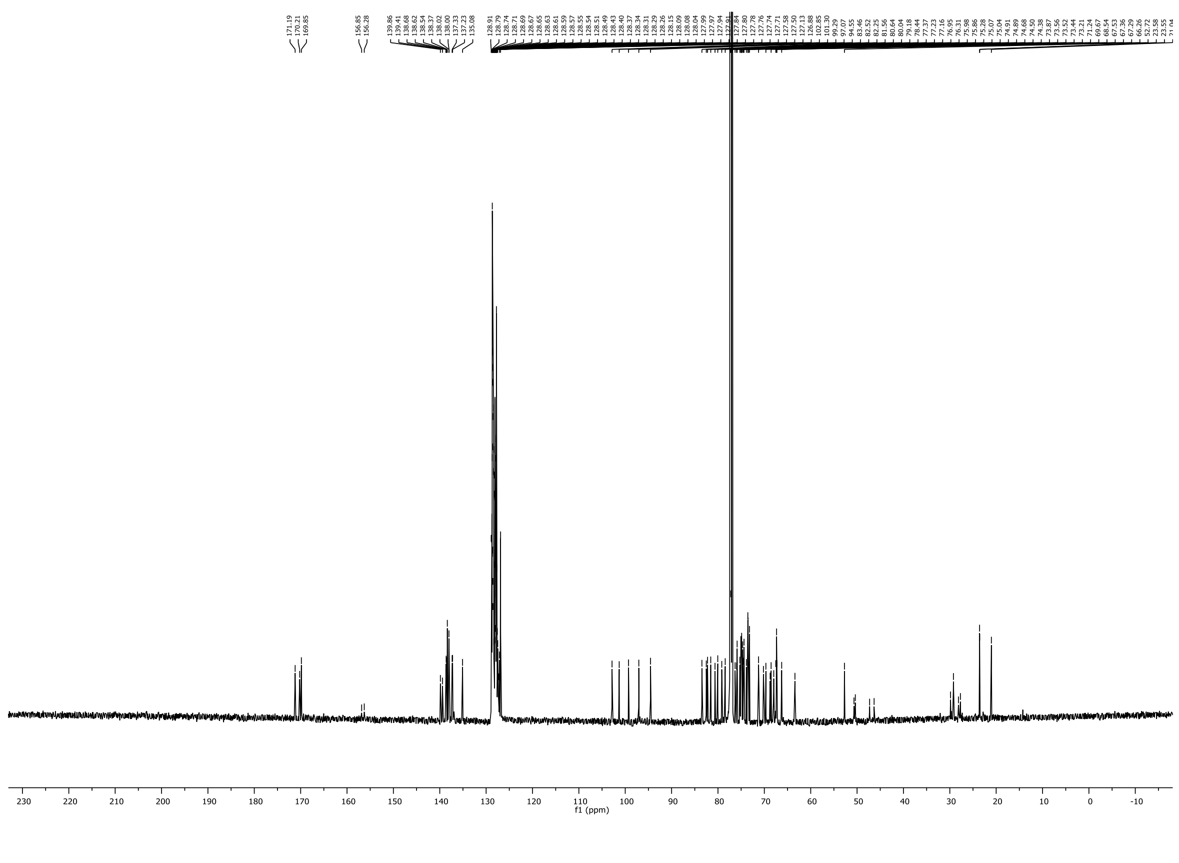
f1 (ppm)

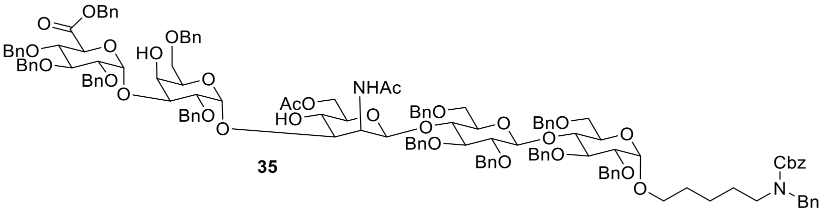

35

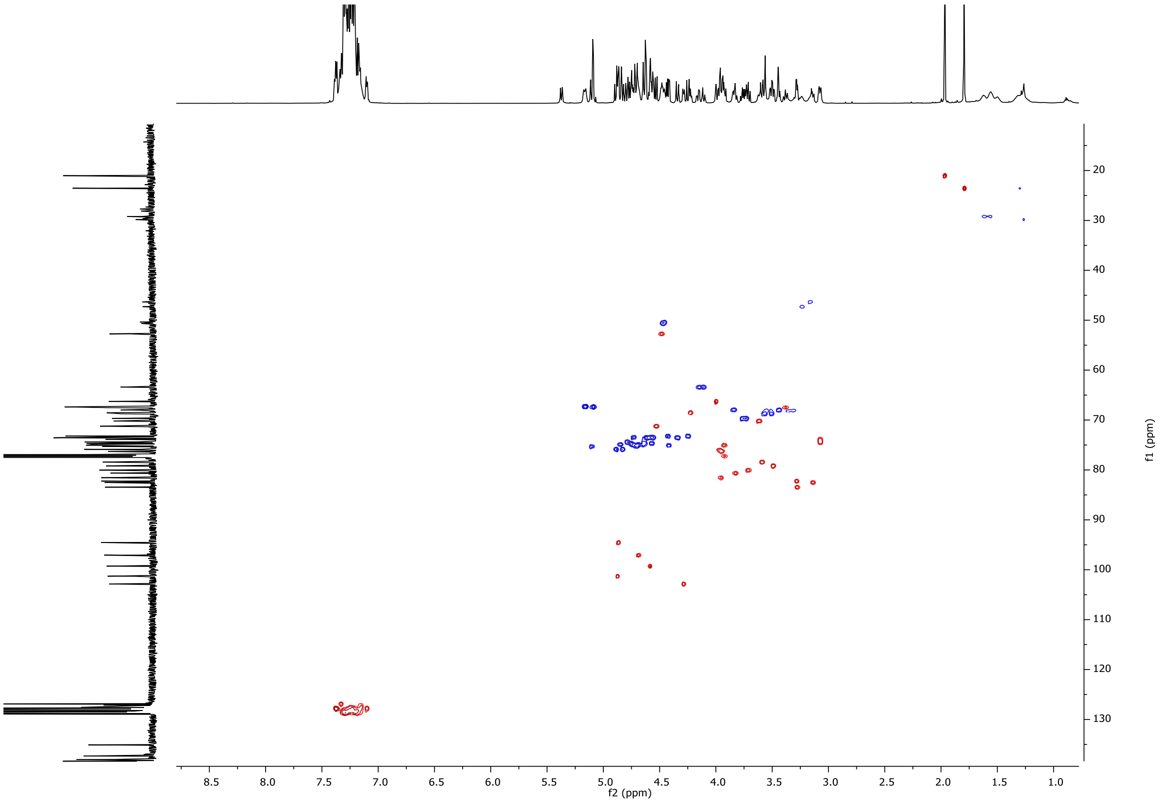

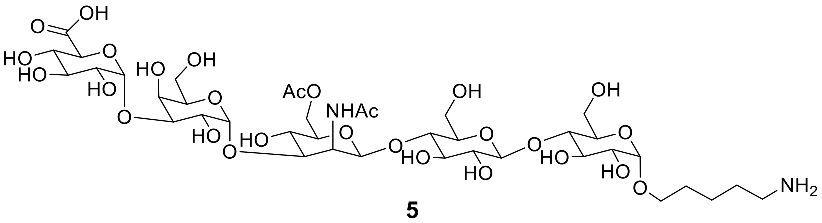

O
OH
HO
HO
HO
O
HO
OH
O
O
HO
O
AcO
NHAc
HO
O
O
OH
HO
HO
O
O
OH
HO
HO
O
O
NH2

5

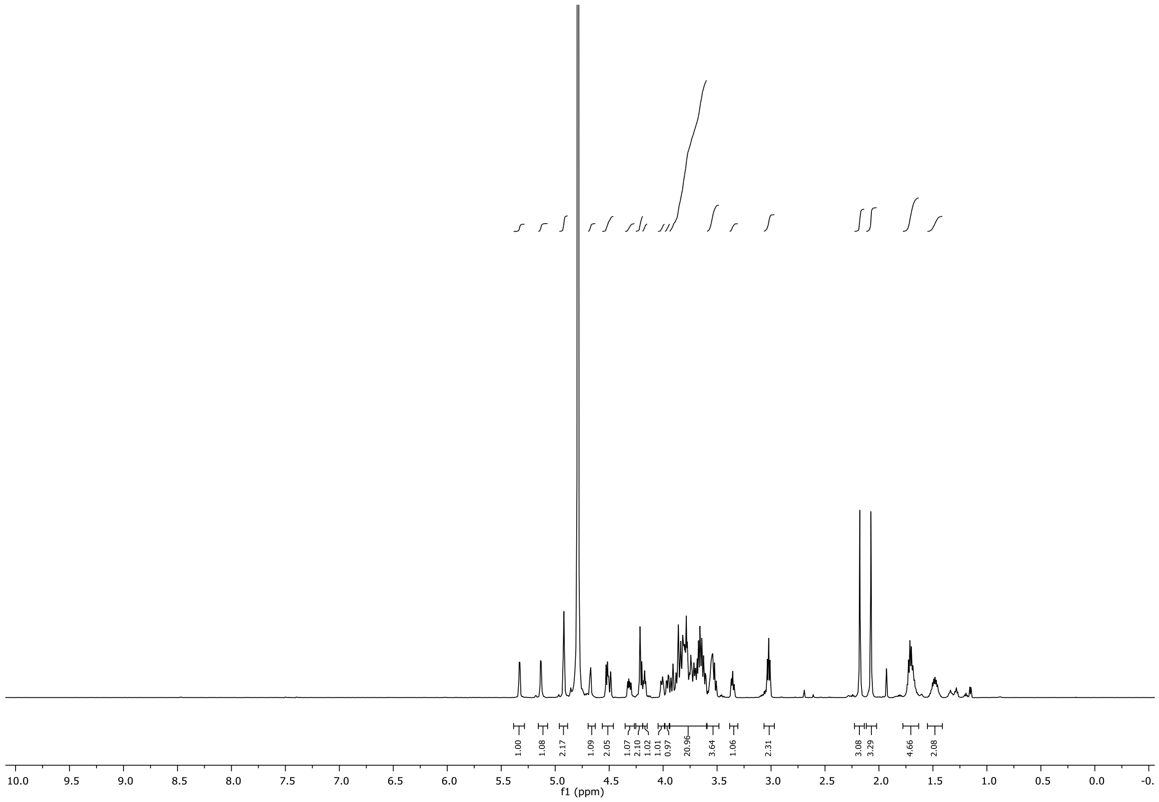

1.00
1.08
2.17
1.09
2.05
1.07
2.10
1.02
1.01
0.97
20.96
3.64
1.06
2.31
3.08
3.29
4.66
2.08
10.0 9.5 9.0 8.5 8.0 7.5 7.0 6.5 6.0 5.5 5.0 4.5 4.0 3.5 3.0 2.5 2.0 1.5 1.0 0.5 0.0 -0.
f1 (ppm)

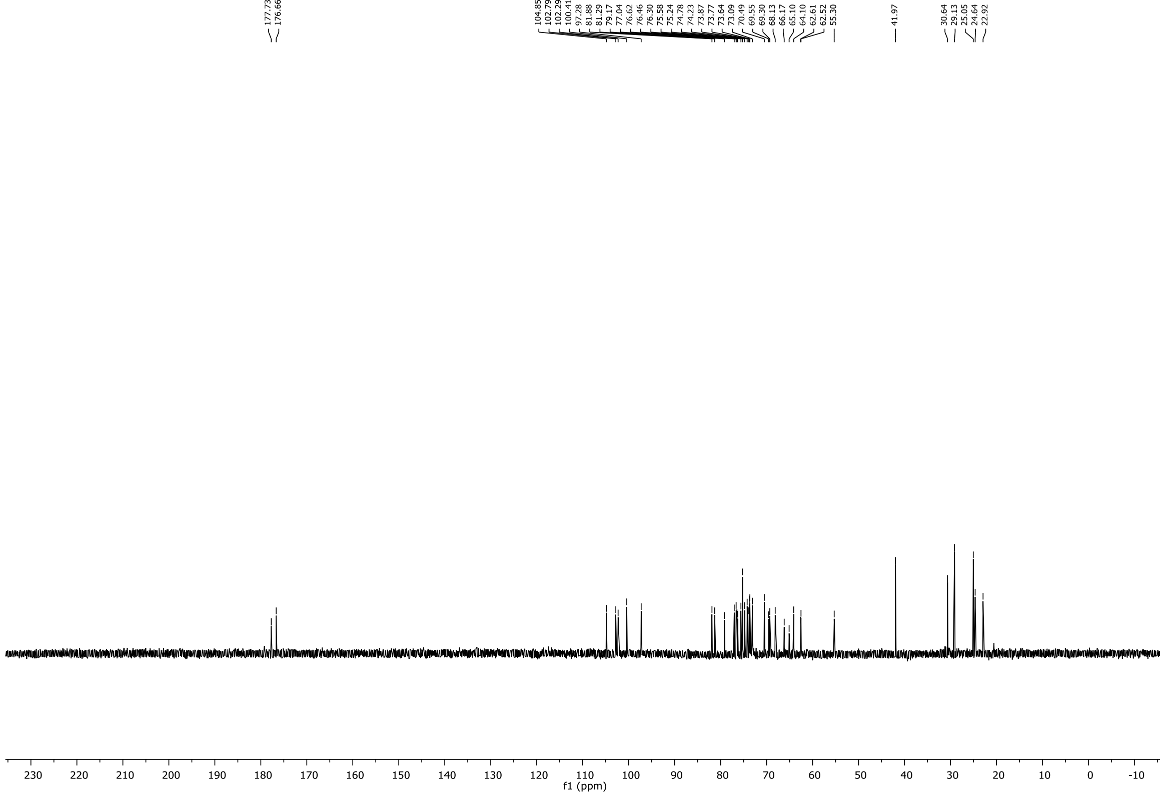

177.73
176.66
104.85
102.79
102.29
100.41
97.28
81.88
81.29
79.17
77.04
76.62
76.46
76.30
75.58
75.24
74.78
74.23
73.87
73.77
73.64
73.09
70.49
69.55
69.30
68.13
66.17
65.10
64.10
62.61
62.52
55.30
41.97
30.64
29.13
25.05
24.64
22.92
230 220 210 200 190 180 170 160 150 140 130 120 110 100 90 80 70 60 50 40 30 20 10 0 -10
f1 (ppm)

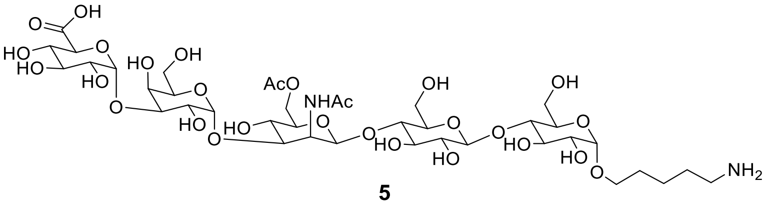

5

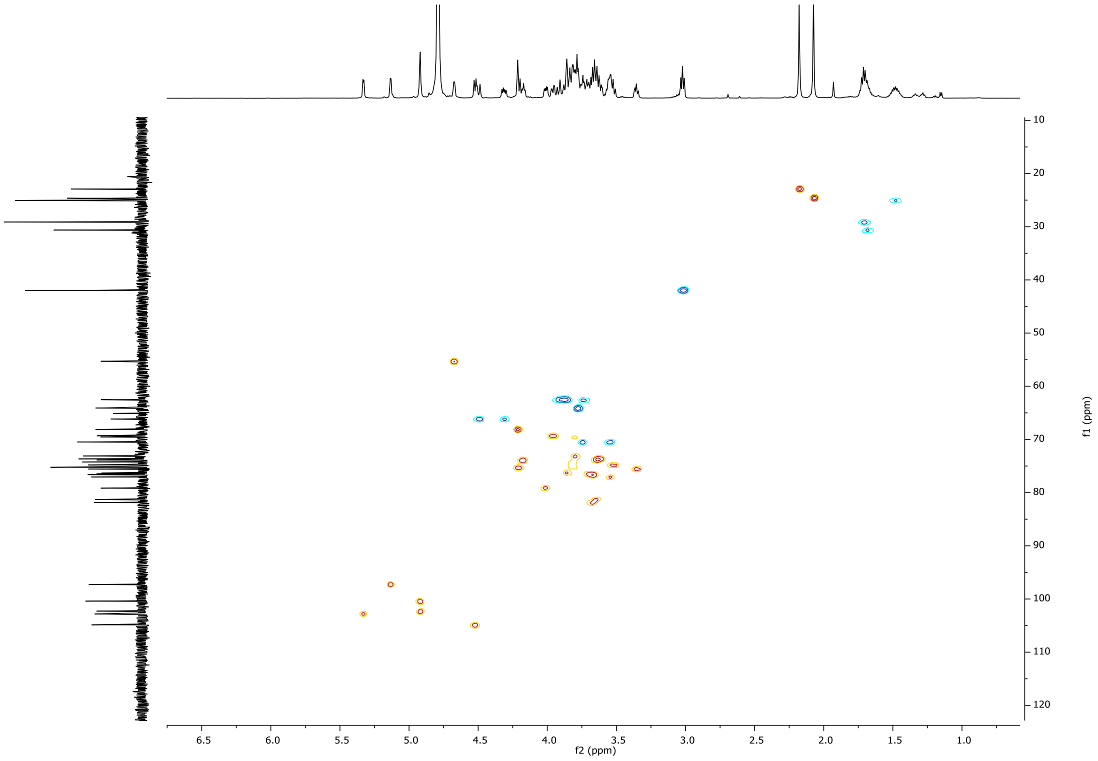

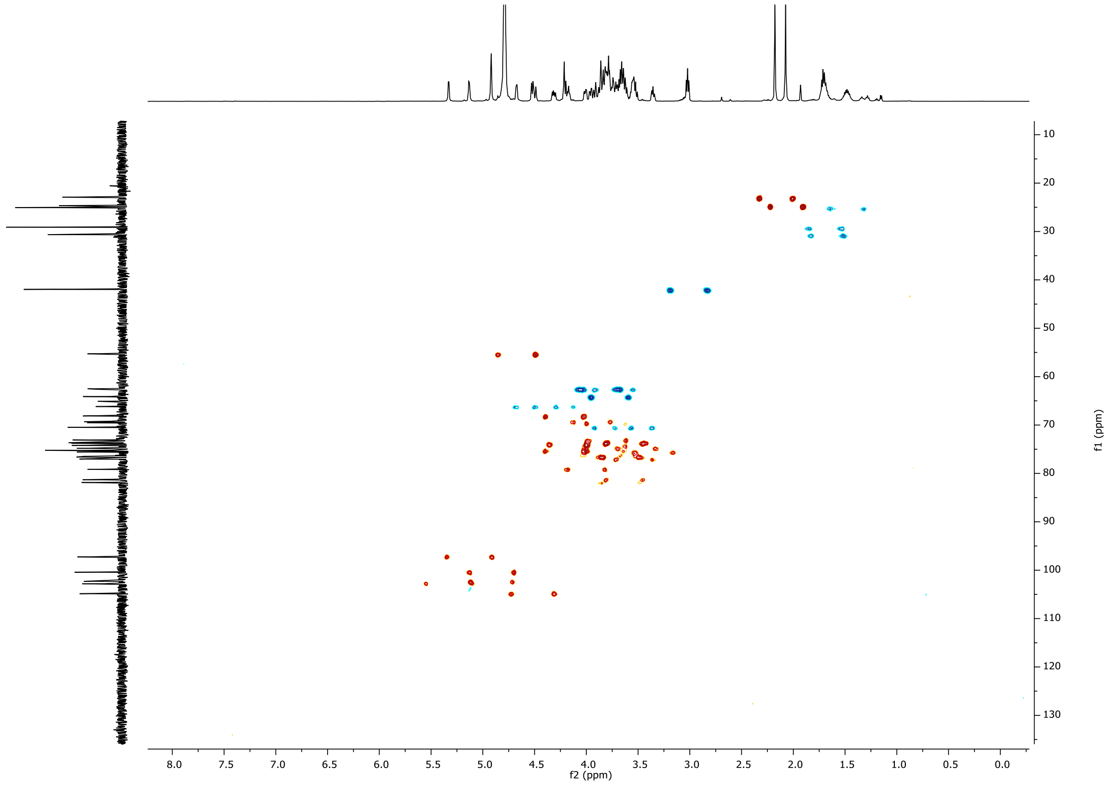

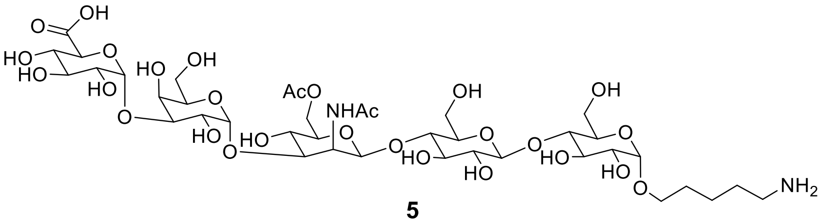

5

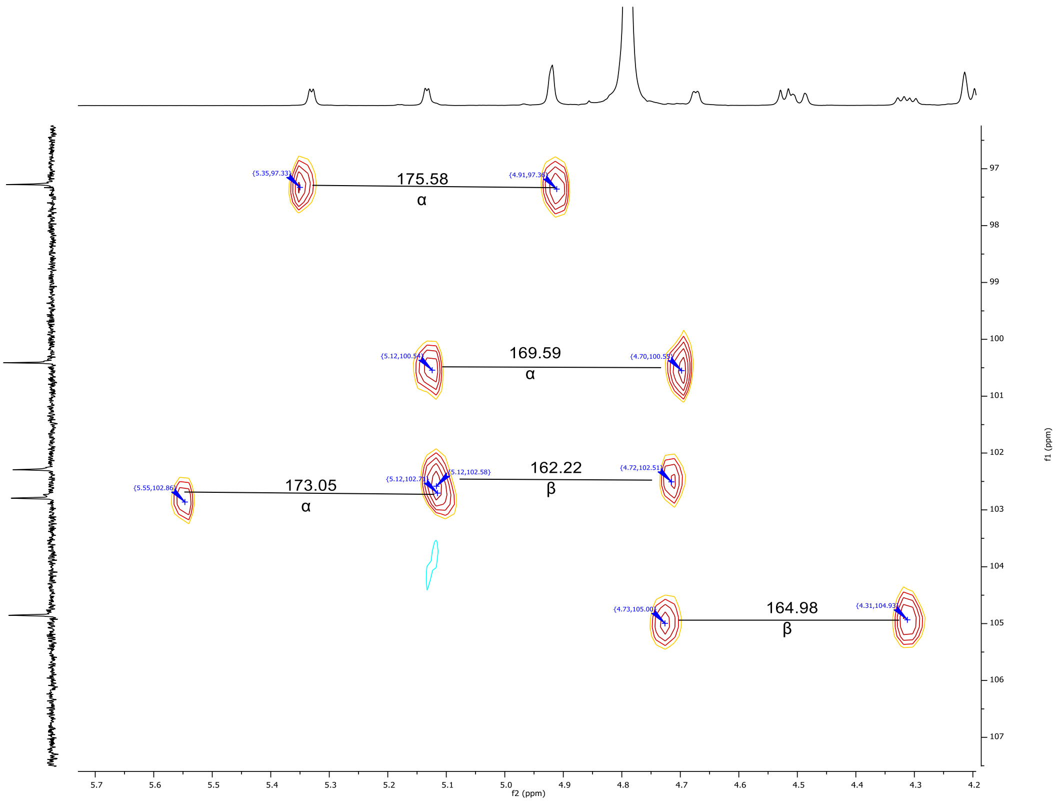

175.58
α
169.59
α
173.05
α
162.22
β
164.98
β
f2 (ppm)
f1 (ppm)

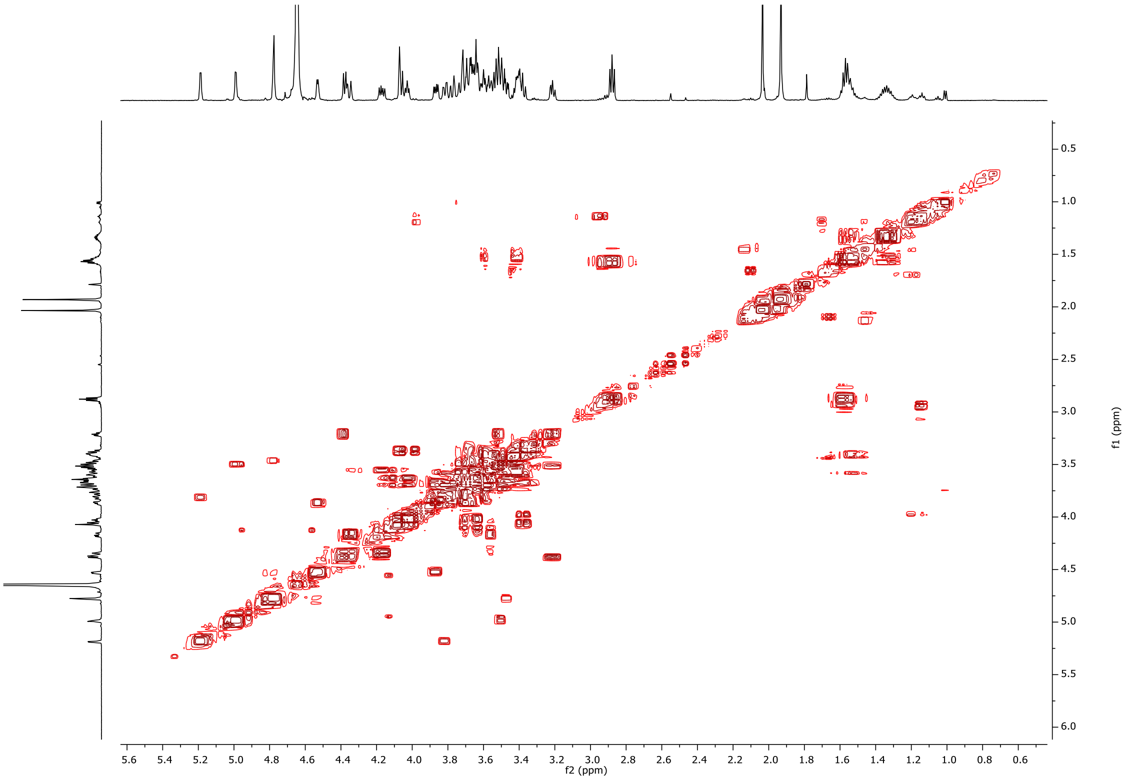

f2 (ppm)
f1 (ppm)

Confirmation of ManNAc-6-OAc:

The HSQC spectra clearly shows the presence of methylene C6 peaks at 4.49 and 4.31 ppm in the case of **5** (blue) which in the case of **4** are present between 3.86–3.94 ppm (orange). Also the new acetyl peak is seen at 2.18 ppm (an overlay of the HSQC's of **4** and **5** is shown below).

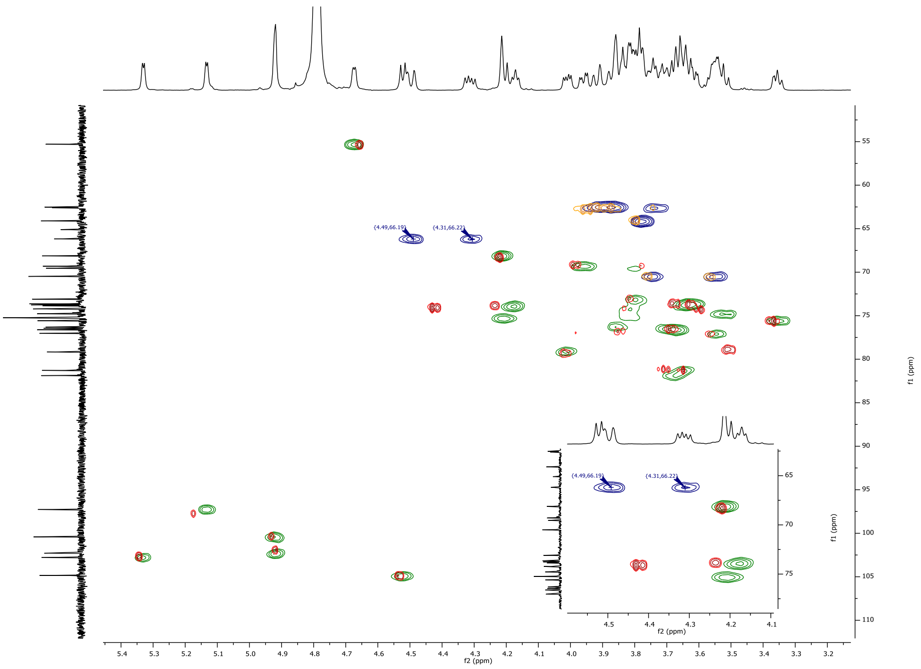

A comparison below of the ^{1}H NMR spectrum with the literature reported confirms the C6 acylation (denoted by # at 4.49 and 4.31 ppm). For further details, please refer to Moon H. Nahm, et. al. *J. Biol. Chem.* **2012,** *287*, 13996-14003 (Supporting Information Figure S3).

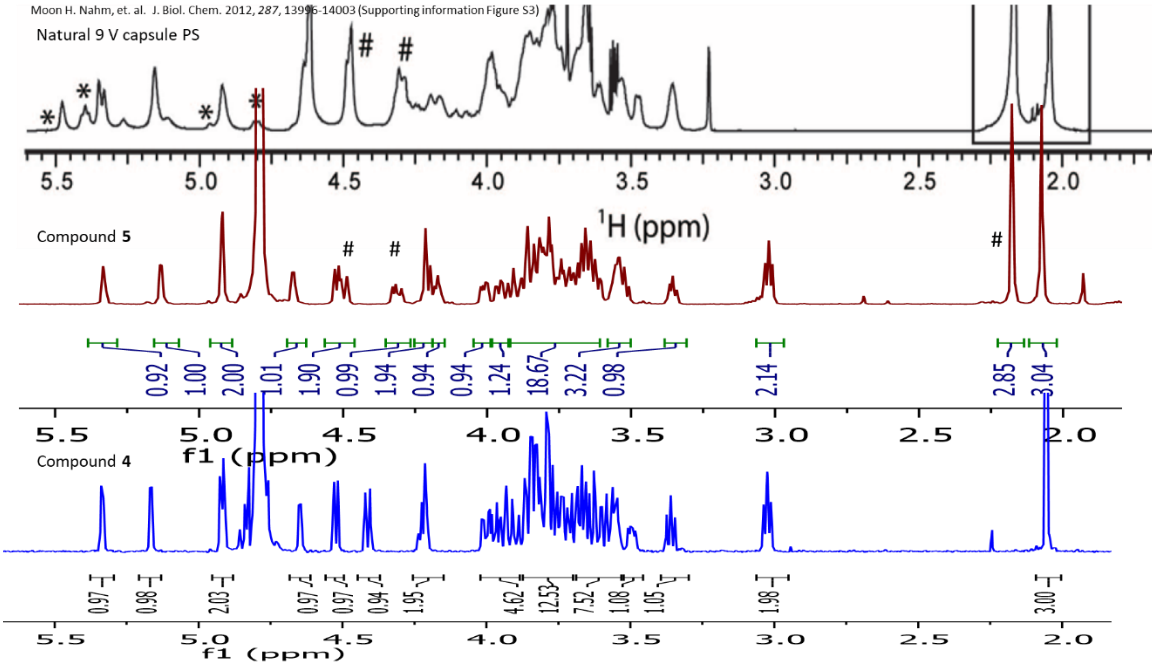

Supplement: File 2 — NMR Spectra. [file Beilstein_J_Org_Chem-16-1693-s002.pdf]
